# RapidMetalFF-AI: An AI-Assisted Web Server for Minute-Scale Bonded Force-Field Parameterization and Structural Validation of Metal-Containing Molecules with Validated AMBER and Converted GROMACS/CHARMM Outputs

**DOI:** 10.3390/molecules31203724

**Published:** 2026-10-17

**Authors:** Barış Kurt

**Affiliations:** Department of Chemistry, Faculty of Science and Literature, Muş Alparslan University, 49250 Muş, Türkiye; peacewolfus00@gmail.com

**Keywords:** force fields, AI-assisted parameterization, machine learning hessian, seminario method, ANS-SMILES, GAFF2, AMBER, OpenMM, web server, Orbmol-v2

## Abstract

Automated bonded-force-field generation for metal-containing molecular systems remains a practical bottleneck because it is expertise-intensive and computationally demanding. RapidMetalFF-AI converts this fragmented process into a minute-scale automated web pipeline integrating an Advanced Numbering System (ANS), machine learning-based geometry optimization and Hessian generation, rapid charge assignment, bonded-parameter generation, minimization, and structural validation. The platform produces bonded parameters, assembled AMBER topology/coordinates, converted GROMACS/CHARMM representations, structural-retention metrics, and ANS-SMILES identifiers. In a deliberately heterogeneous stress test of 90 unedited CSD-derived MOL2 structures, 82 completed the full workflow and 72 contained recognized metal-site subsets. Median native/Hybrid optimized-reference global RMSDs were 0.430/0.415 Å, and metal-site medians were 0.079/0.107 Å. Relative to experimental CSD geometries, final native RapidMetalFF global/metal-site medians were 0.626/0.176 Å; corresponding Hybrid values were 0.623/0.166 Å. Nine representative systems with completed independent HF/SDD optimizations gave median native heavy-atom and metal-site RMSDs of 0.333 and 0.101 Å, a median metal–ligand bond-length MAE of 0.074 Å, and a median coordination-angle MAE of 2.865°. Ten explicit-water, periodic boundary MD tests (5 ns production after heating and equilibration; 50 ns total production) gave median time-averaged global and metal-site RMSDs of 0.571 and 0.112 Å relative to their production-start structures. These additional tests assess local geometry and nanosecond-timescale dynamical stability; they do not establish free-energy, spectroscopic, or general production-MD accuracy. For the 82 completed benchmark structures, the mean and median end-to-end runtimes were 65.5 and 48.9 s per molecule on an NVIDIA GeForce RTX 4060 Laptop GPU (NVIDIA Corporation, Santa Clara, CA, USA).

## 1. Introduction

Metal-containing molecular systems are common targets for molecular dynamics (MD) studies, including metalloenzyme active sites, organometallic catalysts, metal–organic framework fragments, and small-molecule metal complexes deposited in the Cambridge Structural Database (CSD) [1]. For these systems, the practical barrier is frequently the force field rather than the simulation engine. General force fields such as GAFF/GAFF2 provide broad organic coverage [2,3], but coordination environments fall outside ordinary organic atom-type schemes and often require system-specific bonded treatment.

Three broad strategies are commonly used to represent metal centres in classical simulations: nonbonded electrostatic models, cationic dummy-atom models, and bonded covalent models. Bonded models are attractive when directional coordination geometry must be retained, but conventional development can require quantum-mechanical geometry optimization, Hessian evaluation, charge derivation, atom typing, parameter assembly, and application-specific torsional refinement. RapidMetalFF-AI is not presented as a conventional QM workflow made unusually fast, nor is its scientific contribution the standalone implementation of a Hessian-projection formula. The reference geometry and Hessian are generated by the OrbMol-v2 machine learning potential trained on OMol25 and OPoly26; its training domain and post-training evaluations are documented in the corresponding model and dataset reports [4,5,6]. The resulting ML Hessian is then projected to obtain bond and angle terms [7,8]. The scientific question addressed here is whether these AI-generated reference data can support an integrated workflow that constructs, assembles, checks, and preserves molecule-specific bonded models at scale.

Our earlier assign_v2 work introduced position-specific numbering of coordinating atoms for square-planar palladium systems [9]. Its central design principle was to preserve non-equivalent coordination-site identity so that chemically distinct donor positions were not collapsed into a single generic type, while primary metal geometry was controlled principally through explicit metal–ligand bond and ligand-metal–ligand angle terms rather than by mandatory fitting of every metal-centred torsion. In RapidMetalFF-AI, we further developed this concept into the Advanced Numbering System (ANS), as described below, and extended the resulting coordination-site information into the SMILES representation. This led to ANS-SMILES, a metal-aware molecular representation designed to retain coordination-site identity and distinguish geometrically relevant relationships such as cis and trans donor arrangements that can otherwise be lost or remain ambiguous in conventional molecular identifiers. RapidMetalFF-AI therefore extends the original assign_v2’s “numbering system” concept beyond force-field atom typing: the same coordination-aware identity framework is used both for parameter generation and for persistent molecular identification within the automated workflow.

Machine learning interatomic potentials (MLIPs) can remove several upstream bottlenecks simultaneously. RapidMetalFF-AI derives much of its computational efficiency and broad chemical applicability from the use of OrbMol-v2 as the central atomistic engine of the parameterization workflow [4,5]. OrbMol-v2 provides rapidly optimized molecular geometries and the force evaluations required for finite-difference Hessian construction. Together, these capabilities allow two of the most computationally demanding upstream stages of conventional bonded-force-field development to be handled within a single ML-based framework. This capability is particularly important for RapidMetalFF-AI because the quality and chemical coverage of the resulting bonded parameters are strongly influenced by the geometry and force information supplied at this stage. OrbMol-v2 belongs to the Orb family of atomistic models and is associated with training resources that include chemically and elementally diverse molecular data, including transition-metal chemistry [4,5,6]. Its combination of computational speed, force availability, and broad chemical coverage therefore provides a key foundation for the minute-scale automated parameterization of chemically diverse metal-containing systems. Importantly, the resulting MD potential remains a conventional classical force field: AI/ML is used to accelerate the generation of geometry and force information required for parameter construction, rather than to replace the classical force field used in subsequent molecular dynamics simulations.

The central advance of RapidMetalFF-AI is the integration of these methodological components into an operationally different mode of metal-force-field generation. ML-derived geometry and Hessian information alone would accelerate only one part of the conventional workflow, but neither automated atom typing, nor rapid charge assignment, nor web deployment alone would remove the serial bottlenecks that make metal parameterization difficult to scale. RapidMetalFF-AI combines these stages with coordination-aware assign_v2 atom identity, Seminario-derived bonded terms, a protected-metal/GAFF2 Hybrid representation, structural admission control, automated validation, and permanent result reuse. Charge handling is integrated into the same workflow: user-specified total charge and spin multiplicity take precedence when available, while the default electrostatic layer is generated by a rapid GFN1-xTB/CM5 calculation [10,11]. The consequence is a transition from bespoke molecule-by-molecule preparation toward automated processing of large collections of metal-containing structures.

RapidMetalFF-AI is deployed as a public web service at https://rapidmetalff-ai.biochemoinfo.com and maintains a searchable force-field library in which previously validated structures can be identified through conventional molecular identifiers and the coordination-aware ANS-SMILES representation. Exact verified matches can reuse an existing parameter package without repeating the GPU calculation. The broader significance of the platform is therefore its ability to make high-volume metal-complex parameterization operationally tractable on a minute-scale timescale while turning successful calculations into reusable community-facing resources.

## 2. Results

Before entering the parameterization pipeline, each CSD-derived MOL2 structure is first examined by a preflight screening system that evaluates whether the crystallographic MOL2 representation is suitable for direct molecular dynamics preparation. This screening identifies structural features such as problematic component organization, connectivity anomalies, periodic-image or duplicate representations, disorder-related geometry problems, and implausible bond-distance patterns that can prevent a crystal-derived MOL2 file from serving as a simulation-ready molecular input. Structures that pass this stage proceed through principal-component selection when required, OrbMol-v2 geometry optimization, Hessian-based bonded-parameter generation, charge assignment, force-field construction, and final OpenMM [12] minimization.

Two force-field realizations are generated and evaluated. In the native RapidMetalFF branch, the molecular bonded topology is described using the RapidMetalFF parameterization and atom-typing framework. In the Hybrid RapidMetalFF/GAFF2 branch, the metal centre and directly coordinated atoms retain the RapidMetalFF/ANS-derived description, whereas the remaining non-metal–ligand region is assigned GAFF2 bonded parameters where available. The Hybrid branch therefore tests whether established GAFF2 treatment of the ligand region can be combined with the RapidMetalFF description of the coordination environment while preserving the same underlying OrbMol-v2-derived metal-site information.

To evaluate how successfully the workflow preserves molecular and coordination-site geometry during parameterization, several RMSD comparisons were calculated between defined stages of the pipeline. Experimental Geometry RMSD measures the deviation between the CSD-derived starting geometry and the OrbMol-v2 optimized geometry and therefore describes the structural relaxation introduced before force-field construction. Optimized-reference global RMSD compares the final minimized native or Hybrid structure with the corresponding OrbMol-v2 optimized geometry over the complete molecular structure, whereas optimized-reference metal-site RMSD performs the same comparison only for the recognized coordination subset comprising the metal centre or centres and directly coordinated atoms. Experimental-reference global and metal-site RMSDs instead compare the final minimized native and Hybrid structures directly with the corresponding CSD-derived experimental geometry. Thus, optimized-reference RMSDs evaluate structural retention relative to the geometry used for parameter generation, whereas experimental-reference RMSDs evaluate how closely the final simulation-ready structures reproduce the original CSD-derived geometry. These metrics are therefore reported separately throughout the following sections. Where individual benchmark structures are discussed, their **CSD deposition numbers are provided** to enable unambiguous identification of the corresponding crystallographic records.

### 2.1. Benchmark Disposition and Completion Accounting

The benchmark comprised 90 manually selected CSD-derived MOL2 structures chosen to expose RapidMetalFF-AI to a broad range of metal-containing coordination environments and ligand chemistries rather than to reproduce the frequency distribution of the CSD. The structures were processed in their retrieved MOL2 form without manual geometry repair, removal of crystallization components, correction of periodic-image relationships, or molecule-specific editing before submission. This design therefore evaluated the complete automated workflow under realistic input conditions, including principal-component identification and handling of crystallographic representations that are not necessarily equivalent to immediately simulation-ready single molecules.

Of the 90 benchmark structures, 82 produced complete final validation records containing both native RapidMetalFF and Hybrid RapidMetalFF/GAFF2 global RMSD values and therefore formed the fixed quantitative analysis set. The other eight—1100369, 1107459, 1822165, 2083695, 241977, 244158, 2454589, and 2502801—did not yield complete final RMSD records. RapidMetalFF-AI applies an integrated preflight and completion-control layer before and during expensive stages to detect non-computable or unsafe conditions, including direct atom overlaps, duplicate atom identifiers, inconsistent bond geometry, disconnected or periodic-image components, incomplete hydrogen/valence patterns, and severe charge-state inconsistencies. The retained benchmark summary records the resulting admission decision and warning category rather than a molecule-specific terminal traceback. Six of the eight records carried ACCEPT status, whereas 2083695 and 2502801 carried non-fatal crystallographic periodic-image warnings; consequently, no unsupported molecule-specific failure mechanism was assigned. Supplementary Table S1 reports their screening, component accounting, warnings, and final benchmark disposition, making completion-conditioned selection explicit.

Among the 82 completed calculations, 72 contained a recognized metal-site subset comprising the metal centre or centres and directly coordinated atoms and therefore contributed both global and metal-site structural-retention metrics. The other 10 records (1493407, 1574551, 175478, 2075115, 2075193_as, 2076769, 2076771, 2150100, 2515638, and 876252) had no applicable recognized metal-site subset in the selected principal component. They were retained in the experimental-geometry and global force-field comparisons, while their local metal-site quantities were reported as N/A rather than assigned artificial values. Inspection of the original MOL2 connectivity distinguishes two groups. Seven inputs (1493407, 175478, 2075115, 2076769, 2076771, 2150100, and 876252) contained no metal centre recognized by the benchmark site definition; these include organic and main-group compounds containing B, Si, Se, or Te. In three multicomponent inputs, the selected principal component lacked a recognized metal centre although a discarded component contained a metal: 1574551 (Pu-containing component), 2075193_as (Fe-containing component), and 2515638 (Bi-containing component). Supplementary Table S1 records this distinction. The absence of a local metric therefore reflects the analyzed component and the operational site definition, rather than failure of the global RMSD calculation.

The 72 records with an applicable metal-site subset covered 50 distinct coordination-centre elements: Ag, Al, Au, Ba, Be, Bi, Ce, Cr, Cu, Er, Eu, Fe, Ga, Ge, Hg, Ho, In, Ir, K, La, Li, Mg, Mn, Mo, Nb, Os, Pb, Pd, Pt, Rb, Re, Rh, Ru, Sb, Sc, Sn, Sr, Ta, Tb, Tc, Th, Ti, Tl, Tm, U, W, Y, Yb, Zn, and Zr. Ten completed records contained lanthanides (La, Ce, Eu, Tb, Ho, Er, Tm, or Yb), all of which fall within the H–Bi elemental span documented for OMol25. Two records contained actinides—1563993 (Th) and 850911 (U)—which lie outside that documented training range and are therefore treated as extrapolative stress-test cases. Their native/Hybrid experimental-reference global RMSDs were 1.268/1.224 Å (Th) and 1.138/1.166 Å (U); native/Hybrid metal-site RMSDs were 0.331/0.370 Å (Th) and 1.036/1.064 Å (U). With only two actinide records, these values do not support a general accuracy claim for actinides. In RapidMetalFF-AI, “metal-site” is an operational coordination-domain definition rather than a transition-metal-only category.

### 2.2. Experimental CSD Geometry Versus OrbMol-v2 Optimization

For the 82 completed calculations, the RMSD between the CSD-derived experimental starting geometry and the OrbMol-v2 optimized geometry had a median of 0.278 Å (range 0.023–2.532 Å). Fifty-eight of 82 structures (70.7%) were within 0.5 Å of the optimized geometry and 76/82 (92.7%) were within 1.0 Å. This experimental-to-optimized RMSD quantifies the structural relaxation between the CSD-derived starting geometry and the OrbMol-v2 optimized geometry used for subsequent parameter generation. It is therefore distinct from the downstream structural-retention RMSDs, which evaluate how closely the final minimized structures preserve the OrbMol-v2 optimized reference or the experimental CSD geometry.

The largest experimental-to-OrbMol-v2 displacement in the completed set occurred for CSD deposition number 253279, with an Experimental Geometry RMSD of 2.532 Å. The corresponding post-parameterization deviations from the optimized reference were smaller: 0.965 Å for native global RMSD, 1.220 Å for Hybrid global RMSD, 0.501 Å for native metal-site RMSD, and 0.630 Å for Hybrid metal-site RMSD. Its four-component mean RMSD (Optimized Trust RMSD) was 0.829 Å. This case illustrates why a large crystal-to-optimized change should not be interpreted automatically as failure of the generated force field: the experimental-to-optimized and optimized-to-minimized comparisons describe different stages of the workflow.

Supplementary Table S2 aligns the final simulation-ready geometries directly to the CSD experimental structures. Across all 82 completed calculations, median experimental-reference global heavy-atom RMSDs were 0.626 Å (IQR 0.333–0.947 Å) for native RapidMetalFF and 0.623 Å (IQR 0.326–0.877 Å) for Hybrid, with 64/82 (78.0%) and 65/82 (79.3%) within 1.0 Å. For the 72 applicable metal sites, the corresponding medians were 0.176 Å (IQR 0.085–0.358 Å) and 0.166 Å (IQR 0.097–0.346 Å), with 65/72 (90.3%) and 63/72 (87.50%) within 1.0 Å. These four direct final-geometry RMSDs are the components used by Experimental Trust. These fixed historical statistics include the subsequently flagged inputs; the corrected 145192 follow-up, unsupported periodic representations of 850911/993277, and a separate exclusion sensitivity analysis are reported in Section 3.4 and Supplementary Tables S9a–S9d.

### 2.3. Global Structural Retention in the Native and Hybrid Force-Field Branches

Both force-field realizations were evaluated against the same OrbMol-v2 optimized-reference geometry. The native RapidMetalFF branch, in which the molecular topology is treated through the RapidMetalFF internal typing and parameter-completion framework, yielded a median global RMSD of 0.430 Å (IQR 0.221–0.795 Å). The Hybrid RapidMetalFF/GAFF2 branch, which preserves the RapidMetalFF/ANS description for the protected metal-coordination region while assigning the remaining ligand region from GAFF2, yielded a median global RMSD of 0.415 Å (IQR 0.228–0.697 Å). Molecule-resolved optimized-reference global RMSDs for both force-field branches are provided in Supplementary Table S3.

In each branch, 71/82 structures (86.6%) were within 1.0 Å of the common optimized reference. Native RapidMetalFF was below 0.5 Å for 48/82 structures (58.5%), and the Hybrid branch for 49/82 (59.8%). Hybrid global RMSD was lower for 36 structures, whereas native global RMSD was lower for 46. The two branches therefore did not show a uniform ordering across the dataset.

**Figure 1:**
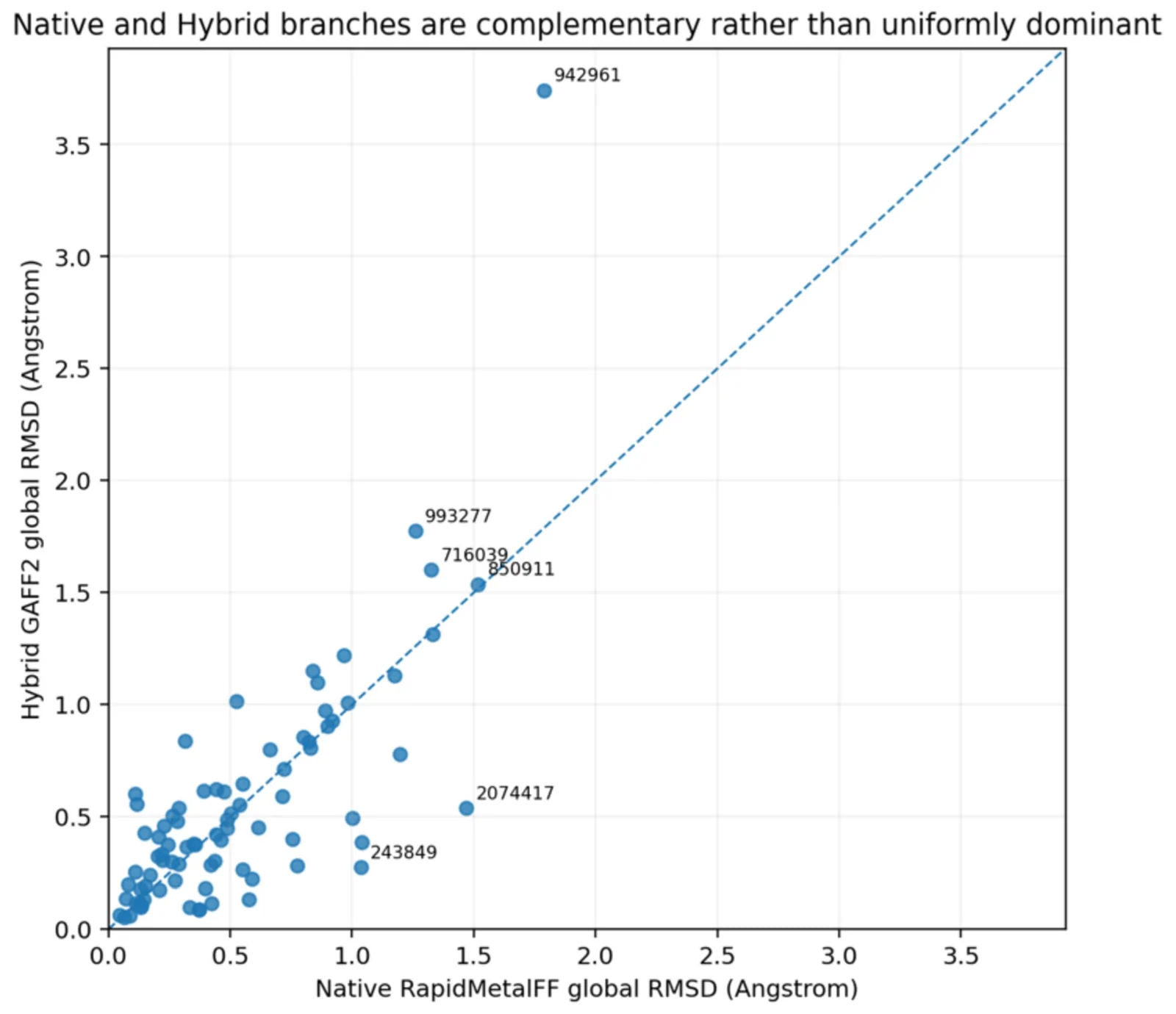
Native RapidMetalFF versus Hybrid RapidMetalFF/GAFF2 global RMSD for the 82 completed CSD-derived calculations. The diagonal marks equal global RMSD. Selected branch-sensitive and high-deviation cases are identified.

This comparison is best interpreted as a sensitivity analysis of the ligand-region force-field realization rather than as a competition between two fully independent models: the two branches share the same optimized geometry, charge-generation route, and RapidMetalFF description of the protected metal-coordination domain, but differ in how the unprotected ligand region is represented. Six completed structures, identified by their CSD deposition numbers, exceeded 1.0 Å in both global branches: 716039, 850911, 851092, 942961, 993277, and 2075193_as. The last of these had no applicable recognized metal-site subset in the selected principal component and therefore could not be evaluated with the local metal-site metrics. The molecule-by-molecule relationship between native and Hybrid global RMSD across the completed benchmark is shown in Figure 1.

### 2.4. Metal-Site Retention and the Four-Component Mean RMSD

Metal-site retention was available for 72 completed structures. The median native metal-site RMSD was 0.079 Å (IQR 0.046–0.267 Å), while the median Hybrid metal-site RMSD was 0.107 Å (IQR 0.049–0.249 Å). In both branches, 67/72 metal-site subsets (93.1%) were within 1.0 Å and 63/72 (87.5%) were within 0.5 Å of the corresponding OrbMol-v2 optimized metal-site geometry.

The substantially smaller local medians compared with the global medians show that whole-molecule motion and ligand reorganization can contribute strongly to global RMSD even when the immediate coordination environment remains close to the optimized reference. This is a structural-retention observation only; it does not by itself establish the accuracy of metal–ligand energetics, free-energy surfaces, or spectroscopic properties.

For records in which all four optimized-retention RMSDs were available, Supplementary Table S3 also reports OptimizedTrustRMSD_A, the equal-weight arithmetic mean of native global, Hybrid global, native metal-site, and Hybrid metal-site RMSDs. This quantity had a median of 0.284 Å (IQR 0.194–0.469 Å); 55/72 structures (76.4%) were at or below 0.5 Å and 67/72 (93.1%) were at or below 1.0 Å. OptimizedTrustRMSD_A remains an Å-scale descriptive average and should not be confused with the 0–100 RapidMetalFF Trust Score, which normalizes each component by the radius of gyration of the same atom subset before averaging component Trust values.

Five structures exceeded 1.0 Å in both native and Hybrid metal-site RMSD and also exceeded 1.0 Å in OptimizedTrustRMSD_A: 1100181 (1.058 Å), 716039 (1.578 Å), 850911 (1.477 Å), 942961 (3.149 Å), and 993277 (2.037 Å). These systems define the clearest local structural-retention outliers among the completed calculations and are considered separately below. The experimental-to-optimized, global, metal-site, and four-component optimized-retention statistics are summarized together in Table 1.

### 2.5. Representative Branch-Sensitive Cases, Outliers, and Trust Interpretation

Several completed calculations illustrate the value of reporting global and local metrics separately. CSD 232528 (Au1; five atoms in the metal-site subset) was a low-deviation case in both realizations, with native/Hybrid global RMSDs of 0.066/0.054 Å and native/Hybrid metal-site RMSDs of 0.048/0.029 Å. CSD 2104359 (Ru1; seven metal-site atoms) similarly showed low global and local deviations.

Other systems were more sensitive to ligand-region treatment. CSD 243849 had a native global RMSD of 1.036 Å but a Hybrid global RMSD of 0.274 Å, while its native/Hybrid metal-site RMSDs were only 0.032/0.023 Å. CSD 2074417 showed a related pattern, with global RMSDs of 1.469/0.539 Å but metal-site RMSDs of 0.032/0.069 Å. Because the local coordination environments remained close to the common optimized reference while the global responses differed, these cases are consistent with branch-sensitive ligand-region relaxation rather than with a simple collapse of the metal site. These branch-sensitive global responses are also apparent in Figure 1.

Five structures exceeded 1.0 Å in both native and Hybrid metal-site RMSD. Four of these—716039, 850911, 942961, and 993277—also exceeded 1.0 Å in both global branches, whereas CSD 1100181 lay close to the same global threshold (0.983/1.008 Å for native/Hybrid) while showing native/Hybrid metal-site RMSDs of 1.100/1.139 Å. The most pronounced case was CSD 942961, with native/Hybrid global RMSDs of 1.788/3.742 Å, native/Hybrid metal-site RMSDs of 2.227/4.838 Å, and an Optimized Trust RMSD of 3.149 Å. Representative low-deviation, branch-sensitive, and high-deviation benchmark cases are summarized in Table 2.

The web-based RapidMetalFF Trust Score described in Section 4.22 was not used for benchmark selection, ACCEPT/WARN status, or the 0.5/1.0 Å descriptive thresholds. It normalizes the four optimized-reference retention RMSDs by OrbMol-v2-reference Rg values. Experimental Trust independently normalizes the four direct experimental/CSD-reference final-geometry RMSDs—native global, Hybrid global, native metal-site, and Hybrid metal-site—using experimental-reference Rg values. Experimental-to-OrbMol-v2 RMSD is diagnostic only and is included in neither score.

### 2.6. Runtime Profile for the Exact 82 Completed Calculations

Runtime analysis used the exact same 82 calculations included in the structural-retention analysis. All timing rows were obtained from the local benchmark environment on an NVIDIA GeForce RTX 4060 Laptop GPU; the recorded queue time was 0 s for every run. The reported values therefore characterize computational workflow time in the benchmark environment and should not be interpreted as guaranteed public-server response times, which can additionally include deployment, network, and queueing delays.

The complete end-to-end workflow required a mean of 65.51 s per molecule and a median of 48.92 s (SD 46.85 s; IQR 38.37–77.69 s; range 27.18–266.07 s). The difference between mean and median reflects a right-skewed runtime distribution rather than the typical molecule requiring more than one minute.

The principal computational cost was associated with the OrbMol-v2 stage. Geometry optimization required a median of 23.51 s and finite-difference Hessian generation 9.89 s, while the complete Orb stage had a median runtime of 41.11 s. By comparison, native RapidMetalFF parameter construction required a median of 0.28 s, GFN1-xTB/CM5 charge assignment 0.76 s, force-field assembly plus OpenMM minimization 2.83 s, and multi-format export 0.52 s. The xTB/CM5 stage illustrates the importance of reporting medians as well as means: its mean was 5.40 s but its maximum was 172.86 s, producing a strongly skewed distribution despite a sub-second median.

Table 3 separates directly recorded stage times from orchestration overhead. The rows named “Complete Orb stage” and “Sum of listed stages” are aggregate quantities that overlap with constituent stage rows and must not be added to those components a second time. Molecule-resolved timings are provided in Supplementary Table S4 and the complete descriptive summary in Supplementary Table S5.

### 2.7. Searchable Library, ANS-SMILES, and Verified Result Reuse

The production deployment extends the workflow beyond one-off parameter generation. Completed records can be indexed with conventional molecular identifiers and ANS-SMILES, allowing users to search for previously parameterized structures while retaining coordination-site information that is relevant to the RapidMetalFF representation. ANS-SMILES is used to identify candidate matches, but equality of ANS-SMILES alone is not sufficient to authorize computational reuse.

Before a stored parameter package is returned, the candidate record is subjected to the stricter identity checks described in Section 4.16, including molecular-graph, metal–donor topology, three-dimensional agreement, charge, spin, and method-version consistency. If a verified match is present before a new calculation begins, the stored result can be returned without repeating the GPU parameterization. A second identity check after OrbMol-v2 optimization allows converged equivalent structures to reuse an existing result before Hessian generation. These library lookups are operational reuse events and were not included in the de novo 82-structure runtime benchmark.

At manuscript preparation, the public library had already expanded beyond the controlled benchmark set through deployment and external academic testing. Those additional records were not pooled into the RMSD or runtime statistics reported here. This separation keeps the benchmark numerically fixed while allowing the operational library to continue growing independently.

### 2.8. Independent QM Geometry and Local-Coordinate Validation

The independent-QM validation set comprised nine systems spanning Nb, Pt, Mo, Sc, Au, In, Ga, Bi, and Sn coordination chemistry, each with verified atom correspondence between the QM and RapidMetalFF-AI structures. All calculations used HF/SDD optimization followed by frequency analysis. Every job terminated normally and all nine optimized structures had no imaginary frequencies; their lowest frequencies ranged from 16.979 to 38.968 cm^−1^. Follow-up RapidMetalFF runs used reviewed charge/multiplicity states; for 902738 and 1100364 these differ from the original fixed exact-82 benchmark records, which were not retroactively altered.

Across the nine completed systems, final native RapidMetalFF structures gave median independent-QM heavy-atom and metal-site RMSDs of 0.333 and 0.101 Å, respectively. Median metal–ligand bond-length MAE was 0.074 Å and median coordination-angle MAE was 2.865°. Table 4 reports the native final-geometry values; Supplementary Table S7 provides OrbMol-v2, native, and Hybrid comparisons. The selected subset provides an independent diagnostic across nine elements rather than a universal accuracy ranking. The HF/SDD and MD benchmark sets were designed to address distinct questions and therefore differ in size: nine complexes were assessed against independent static HF/SDD geometries, whereas ten complexes were evaluated by explicit-solvent MD for short-timescale coordination-shell stability.

### 2.9. Short Explicit-Solvent Molecular Dynamics Tests

Ten systems were tested in explicit TIP3P water with periodic boundary conditions and PME electrostatics. After minimization, 50 ps heating and 100 ps NPT equilibration, each system underwent 5 ns NPT production at 300 K and 1 bar with a 0.5 fs unconstrained time step. Coordinates were sampled every 2 ps, yielding 2500 production frames per system and 50 ns of aggregate production sampling. Heavy-atom and metal-site RMSDs were aligned independently to the first production frame. For 1100364, the GFN1-xTB charge step failed; GFN2-xTB partial charges [13] were used for this follow-up trajectory while retaining the RapidMetalFF bonded, torsional, and Lennard-Jones terms.

Across the ten trajectories, mean metal-site RMSDs ranged from 0.083 to 0.191 Å and maxima from 0.178 to 0.350 Å (Table 5). Global mean RMSDs ranged from 0.371 to 1.761 Å, reflecting larger peripheral-ligand motion than coordination-site motion. The median of the ten time-averaged global and metal-site RMSDs was 0.571 and 0.112 Å. The corresponding last-1-ns medians were 0.634 and 0.113 Å, indicating that the local coordination domains remained stable through the final sampling interval. These trajectories assess nanosecond-scale structural stability and are not used to claim converged thermodynamic or kinetic observables.

## 3. Discussion

### 3.1. Structural Retention Across a Heterogeneous CSD-Derived Stress Test

The principal result of the revised benchmark is that a chemically heterogeneous collection of unedited CSD-derived inputs can be taken through a single automated route to generate complete, simulation-ready force-field packages for a substantial set of structurally diverse molecules, while retaining an explicit record of the inputs that do not yield complete validation outputs. The 82-record structural statistics should therefore be interpreted conditionally: they describe calculations that reached the defined completion criterion and may not estimate performance across all 90 inputs, because non-completion may be associated with molecular complexity or other unrecorded downstream factors. The complete 90-structure disposition and the 82/90 completion fraction are reported separately so that completion filtering remains visible rather than hidden.

Within the completed set, both force-field realizations generally remained close to the common OrbMol-v2 optimized reference after standardized OpenMM minimization. The native and Hybrid global medians were 0.430 and 0.415 Å, respectively, and the corresponding metal-site medians were 0.079 and 0.107 Å (Supplementary Table S3). The stronger local retention is important because global RMSD can be increased by conformational changes in peripheral ligand regions that do not necessarily disrupt the immediate coordination sphere. Reporting global and local quantities together therefore provides a more informative structural diagnostic than either quantity alone (Supplementary Table S3).

The experimental CSD geometry provides an additional and conceptually separate reference layer. Crystal-to-OrbMol-v2 RMSD measures the structural change introduced before force-field validation; optimized-reference RMSD then tests whether the assembled classical model reconstructs the same minimum used for parameter derivation. The latter is an internal structural-retention test and is partly self-referential. Direct final-geometry comparison with CSD and the independent QM subset described in Section 2.8 provide reference layers that do not use the OrbMol-v2 minimum as the target.

Direct final-geometry comparisons in Supplementary Table S2 use the experimental structure itself as the reference. Native and Hybrid branches gave median global deviations of 0.626 and 0.623 Å from CSD, and median metal-site deviations of 0.176 and 0.166 Å. The nine independently optimized HF/SDD cases further yielded a median native heavy-atom RMSD of 0.333 Å, a median native metal-site RMSD of 0.101 Å, a metal–ligand bond-length MAE of 0.074 Å, and a coordination-angle MAE of 2.865°. RMSD is an established MM-versus-QM geometry benchmark [14], and these global and local medians remain in the sub-angstrom structural-agreement regime. In particular, the 0.101 Å metal-site median lies within the 0.0492–0.2976 Å coordination-core RMSD range previously reported for assign_v2 palladium systems [9]. The ten 5 ns explicit-solvent trajectories provide an additional dynamical check, with a median time-averaged metal-site RMSD of 0.112 Å, supporting retention of the coordination domains throughout 50 ns of aggregate production sampling.

### 3.2. Native and Hybrid Realizations Probe Different Parts of the Same Parameterization Problem

The native and Hybrid branches are deliberately related but non-identical realizations. The native branch treats the molecular topology through the RapidMetalFF internal typing and completion framework, whereas the Hybrid branch retains the RapidMetalFF/ANS description at the metal and directly coordinated atoms but delegates the unprotected ligand region to GAFF2. Their comparison therefore probes the sensitivity of the minimized structure to ligand-region parameterization while holding the protected coordination domain and optimized reference fixed.

Neither branch was uniformly superior across the completed benchmark. Hybrid global RMSD was lower for 36/82 structures and native global RMSD for 46/82; at the metal-site level, Hybrid was lower for 28/72 and native for 44/72 (Supplementary Table S3). This absence of uniform dominance is consistent with the intended use of the Hybrid branch as an alternative ligand-region realization and cross-check rather than as a replacement that is expected to improve every molecule. Agreement between the branches increases confidence that the structural result is not strongly dependent on the ligand-region treatment, whereas disagreement identifies systems that deserve closer inspection.

The two-branch design exposes sensitivity to ligand-region parameterization that would be invisible in a single force-field realization. The limits of the structural comparisons are summarized in Section 3.9.

### 3.3. Input Screening, Development Chronology, and Avoidance of Circular Validation

Bond and angle terms are derived from the OrbMol-v2 Hessian through the Seminario projection, while charge and remaining force-field terms follow the procedures described in Section 4. The principal methodological consequence of screening is admission control: it determines whether an input is sufficiently well formed to enter a fully automated calculation, not the numerical force constants assigned after a calculation has been admitted.

### 3.4. Challenging Completed Cases Define the Practical Structural-Retention Boundary

The strongest completed outliers are scientifically useful because they demonstrate that workflow completion is not equated with structural success. CSD 1100181, 716039, 850911, 942961, and 993277 all exceeded 1.0 Å in both native and Hybrid metal-site RMSD, with 942961 showing the largest deviations in the set. Of these five cases, 716039, 850911, 942961, and 993277 also exceeded 1.0 Å in both global branches, whereas 1100181 remained close to the global threshold. These records remain visible in the component-level outputs and in the public Trust analysis rather than being hidden by an aggregate score.

A structure-level follow-up of the charge/spin flags identified distinct input and propagation failures (Supplementary Table S9a). The historical 145192 calculation used an incomplete 42-atom C18H23Yb fragment with 201 electrons at Q = 0; its recorded M = 1 is therefore inconsistent. The corrected 81-atom C32H46OYb2 component contains two Cp* anions, a C8H8 dianion, and neutral THF, supporting a neutral closed-shell assignment with two Yb(II) centres. It was rerun separately at Q = 0/M = 1. OrbMol-v2 optimization reached a maximum force of 0.009256 eV/Å in 214 steps, and GFN1-xTB CM5 charges summed to zero within numerical precision. Native and hybrid topologies preserve all 81 atoms and 106 source edges, with verified element identities, masses, and total charge. Their heavy-atom RMSDs are 0.5208 and 0.5803 Å relative to the Orb target and 0.5554 and 0.5856 Å relative to the cleaned crystallographic component. These follow-up values do not replace the historical 42-atom record in the fixed aggregate.

In contrast, 850911 and 993277 are periodic network fragments with repeated crystallographic images rather than complete isolated molecular entities. In 850911, four U records map to one translationally unique U site; completing its first-shell phosphate ligands still leaves connections to external U images. In 993277, eight Tb records form two translational equivalence classes, with additional repeated O sites and incomplete network boundaries. The stored Q = 0 values therefore do not establish a chemically meaningful isolated-molecule state for these selected fragments. Failed xTB attempts for 850911 and incomplete CM5 charge extraction for 993277 led to source-charge fallback; sums of −3 and −1 e were propagated into native and hybrid topologies. These supplied representations are classified as unsupported periodic inputs, and their historical fallback topologies are not accepted as chemically verified molecular parameter sets. No arbitrary capping, protonation, or multiplicity was introduced to manufacture isolated clusters.

The original 82-record distribution remains a historical, completion-conditioned description. Excluding the three flagged historical records in a separate sensitivity analysis gives 79 global and 69 applicable metal-site records. The experimental-reference global median RMSDs change from 0.6262 to 0.6165 Å for the native model and from 0.6227 to 0.6222 Å for the hybrid model. This sensitivity subset is not an electronically validated benchmark. A source-to-topology comparison of all 82 matched archives identified additional connectivity discrepancies in 158148, 232591, 251255, 716039, and 849384. Separate corrected native and hybrid minimizations were completed for these five records. All 15 native, hybrid, and hybrid-export AMBER topologies preserve atom identities, element numbers, masses, individual charges, and every source edge; all ten minimized geometries pass the structural-integrity checks. Their atom/edge counts are 102/113, 43/52, 67/72, 91/109, and 79/80, respectively. Native/hybrid heavy-atom RMSDs relative to their existing Orb targets are 0.6389/0.3540, 0.0597/0.0379, 0.4540/0.4964, 0.7786/0.8826, and 0.4034/0.2856 Å (Supplementary Table S9b). These topology corrections are reported separately from the historical aggregate and do not establish ground-state validity. High deviations or uncertain molecular boundaries still require case-specific review before production use.

### 3.5. Scope of the ML Hessian, Torsional Treatment, and Charge Model

RapidMetalFF-AI uses the ML-derived Hessian specifically for the portion of the classical force field for which local curvature information is directly exploited in the present workflow: bond and angle parameter generation. Proper torsions, impropers, masses, and nonbonded terms are completed through internal rules or inherited parameter tables, while the Hybrid realization introduces GAFF2 outside the protected metal-coordination region. The resulting molecular dynamics model is therefore a conventional classical force field informed by ML-derived geometry and curvature data rather than an ML potential used directly for production MD.

The zero-barrier treatment of metal-centred proper torsions is a deliberate bonded-model design choice rather than an assumption that metal-containing torsional energetics are universally unimportant. A comparable treatment has precedent in the Zinc AMBER Force Field (ZAFF), where the metal-site parameterization focused on bond, angle, and electrostatic terms while dihedral terms were omitted [15]. The same principle was explicitly adopted in assign_v2 [9], where metal-containing dihedral force constants were set to zero while square-planar coordination geometry was encoded principally through position-specific metal–ligand bond and ligand–metal–ligand angle parameters. Under that parameterization strategy, comparison of minimized structures with experimental crystallographic geometries for palladium and its four directly coordinated atoms yielded RMSDs of 0.0492–0.2976 Å, providing prior structural evidence that accurate local geometry can be retained without fitting an explicit non-zero torsional barrier for every metal-containing dihedral.

RapidMetalFF-AI generalizes this concept to a much broader set of coordination environments. ANS preserves non-equivalent coordination-site identity, while the OrbMol-v2 Hessian supplies the bond and angle curvature information used to construct the local bonded model. The present CSD-derived benchmark then evaluates the structural consequence of this approximation directly through both optimized-reference and experimental-reference global and metal-site RMSDs. Thus, the zero-torsion approximation is assessed here in terms of its intended purpose—preservation of coordination structure after force-field construction and minimization—rather than as a model of the full torsional free-energy surface.

This distinction defines an important boundary of the method. RapidMetalFF-AI omits routine molecule-specific metal-centred PES scans from the default automated workflow to achieve minute-scale parameterization. The 5 ns explicit-solvent trajectories show that the tested coordination sites remain locally stable over the sampled interval, but they do not determine rotational barriers or equilibrium conformer populations. Applications that depend on torsion-dependent free energies, metal–ligand rotation, or conformational kinetics should refine the relevant terms against a system-specific PES.

Similarly, GFN1-xTB/CM5 provides a rapid default electrostatic layer rather than a universal substitute for system-specific ESP/RESP or other higher-level charge models. The workflow is intentionally modular in this respect: the generated bonded parameters can be retained while users replace the charge model when the scientific application requires a more specialized electrostatic treatment.

### 3.6. Runtime, Computational Bottlenecks, and Comparison Context

The exact-82 timing analysis supports the practical claim that the complete scientific workflow operates on a minute-scale timescale in the tested local GPU environment. The median end-to-end runtime was 48.92 s, with the complete Orb stage accounting for most of the typical wall time. Parameter construction, OpenMM validation, and export were comparatively inexpensive once the optimized geometry and Hessian were available.

The timing distribution also shows why a single mean value would be misleading. A small number of long optimization, Hessian, or xTB calculations produced a pronounced right tail, increasing the mean end-to-end runtime to 65.51 s despite a sub-minute median. For the same reason, the xTB/CM5 stage had a median below one second but a much larger mean because of an isolated long calculation. Reporting molecule-resolved timings and full descriptive statistics therefore provides a more realistic picture of operational performance than a single headline number.

The reported times describe RapidMetalFF-AI on the stated RTX 4060 Laptop GPU and are not a hardware-normalized comparison with unrelated parameterization software. A cross-program wall-time ratio would mix differences in reference-data generation, charge treatment, chemical scope, hardware, user-supplied inputs, and output definitions. The manuscript therefore reports the measured minute-scale runtime of the present workflow without claiming a universal speed advantage. Public-server response time can additionally include queueing and deployment overhead absent from the local benchmark.

### 3.7. Trust Score as a Simplified User-Guidance Indicator

The Trust indicators were added primarily for usability rather than to introduce a new scientific validation endpoint. A public web server can be used by researchers with very different levels of familiarity with bonded-force-field validation, and a set of separate global and metal-site RMSDs may not provide an immediately intuitive overview to a non-specialist. The 0–100 summaries therefore serve as coarse, rapid orientation tools: they allow a user to glance at a completed record and obtain an initial impression of whether the final minimized structures remained close to the relevant reference geometry.

Experimental Trust is particularly useful in this context because, when a genuine experimental CSD structure has been verified, it places the final native and Hybrid geometries directly against the experimental reference. This provides a simple experimental-reference overview in the library without requiring the user to interpret four separate RMSD values at first sight. Its restricted activation is equally important: applying an ‘experimental’ score to arbitrary modelled or optimized inputs would give the indicator a meaning that the underlying reference structure does not support. For this reason, unverified records remain N/A rather than receiving an automatically generated Experimental Trust value.

The simplified presentation does not conceal the scientific data. The score itself functions as an entry point to the component-level analysis: clicking it exposes the individual RMSDs together with Rg, RMSD/Rg, component Trust, atom count, and reference geometry. Users who need the scientific structural evidence can therefore move directly from the coarse summary to the underlying measurements, while users seeking only a quick first look are not required to interpret several separate RMSD fields before deciding whether a result warrants closer inspection.

Accordingly, the scientific structural evidence reported by RapidMetalFF-AI remains the optimized-reference and experimental-reference global and metal-site RMSDs. RapidMetalFF Trust and Experimental Trust are interface-level aids constructed from those measurements for rapid user guidance, especially for non-specialist users. They are not probabilities, do not imply that a particular score corresponds to a percentage likelihood of force-field accuracy, and were not used as ACCEPT/WARN rules, benchmark inclusion criteria, or substitutes for the RMSD-based structural analysis.

### 3.8. Coordination-Aware Identity, Verified Reuse, and Reproducibility

ANS-SMILES links the force-field numbering concept to the searchable library by retaining canonicalized coordination-site numbers in a SMILES-compatible representation. Its role is deliberately limited to coordination-aware search and candidate matching. Exact computational reuse requires the stricter graph and three-dimensional checks described in Section 4.16 together with charge, spin, and method-version agreement. This separation reduces the risk that structures sharing a formula, conventional identifier, or superficially similar coordination pattern are treated as interchangeable parameterizations.

The reusable library also changes the operational value of successful calculations: a validated result can become a future cache hit rather than an isolated one-time output. At the same time, each downloadable result package retains the optimized geometry, Hessian, parameter files, atom mapping, native and Hybrid validation outputs, structural-integrity reports, molecular-identity records, exports, metadata, and stage-specific logs. These records provide an inspectable calculation trail even though the production web application and deployment code are not publicly distributed.

### 3.9. Intended Validation Domain

RapidMetalFF-AI is designed to automate bonded-force-field generation, assembly, and immediate structural validation for metal-containing molecular systems. The revised validation is layered: optimized-reference RMSD measures internal reconstruction of the OrbMol-v2-derived minimum; direct CSD comparisons provide an experimental structural reference; independent HF/SDD comparisons provide heavy-atom, metal-site, metal–ligand bond-length, and coordination-angle checks for nine representative systems; and ten explicit-solvent trajectories assess local stability over 5 ns of production per system.

Reaction energetics, ligand-field splittings, redox energetics, vibrational spectra, kinetic barriers, binding affinities, radial-distribution convergence, and solution-phase free energies are distinct theoretical targets requiring property-specific electronic-structure methods, sampling protocols, and validation criteria. The present structural and short-timescale dynamical tests do not establish those properties. RapidMetalFF-AI instead supplies a rapid, auditable first-line bonded model that can be refined and validated within the application-specific protocol chosen by the investigator.

Structural fidelity is a primary validation target for the bonded-force-field model developed here because the equilibrium bond lengths, coordination angles, and their associated force constants define the location and local curvature of the molecular-mechanics minimum. In RapidMetalFF-AI, the reference equilibrium geometry is obtained by OrbMol-v2 optimization, while bond and angle force constants are derived from the corresponding Hessian through the Seminario projection. Consequently, minimization of the independently assembled classical force field provides a direct test of whether this parameterization reconstructs and retains the intended molecular and coordination-site minimum. Substantial inconsistencies in the derived bonded terms would be expected to manifest as displacement from the reference geometry during minimization, whereas low global and, particularly, metal-site RMSDs indicate preservation of the targeted bonded structural environment. The use of optimized-geometry agreement as a force-field benchmark is well established in force-field development and parameterization studies [8,14,16].

Final native RapidMetalFF and Hybrid geometries were aligned directly to their CSD-derived experimental structures, and nine systems with completed independent HF/SDD calculations were additionally evaluated using whole-molecule RMSD, metal-site RMSD, metal–ligand bond-length MAE, and coordination-angle MAE. All nine frequency calculations terminated normally without imaginary modes. These tests reduce reliance on the OrbMol-v2 reference, although crystal packing and the deliberately selected QM subset limit broad quantitative interpretation.

The ten explicit-water trajectories complement the static comparisons by testing coordination-environment retention over 5 ns per system. Together, the reference layers support automated topology construction, local structural retention, and nanosecond-timescale stability for the tested systems.

### 3.10. Future Works

Although the ANS-based numbering system provides an effective solution for distinguishing non-equivalent metal–ligand interactions and coordination positions within the current RapidMetalFF-AI workflow, it is ultimately intended to serve as an intermediate representation layer rather than a permanent requirement of the force-field architecture. Preliminary internal tests indicate that bonded parameters can be reorganized and represented without dependence on the numbering layer. A longer-term objective is therefore to convert metal parameters into a general parameter infrastructure that can be used directly by standard AMBER tools, including **tleap and antechamber**, without generating molecule-specific numbered atom types. Conceptually, this would provide a GAFF2-like parameter database analogous to gaff2.dat, in which parameters for multiple metal centres could coexist within a common representation and be applied directly through standard AMBER tooling. The same general strategy may also extend beyond small metal-containing molecules. Given appropriate reference data, analogous approaches could be explored for identifying and correcting systematic bonded-parameter deviations in protein, DNA, and RNA force fields.

## 4. Materials and Methods

### 4.1. RapidMetalFF-AI Workflow Overview

**Figure 2:**
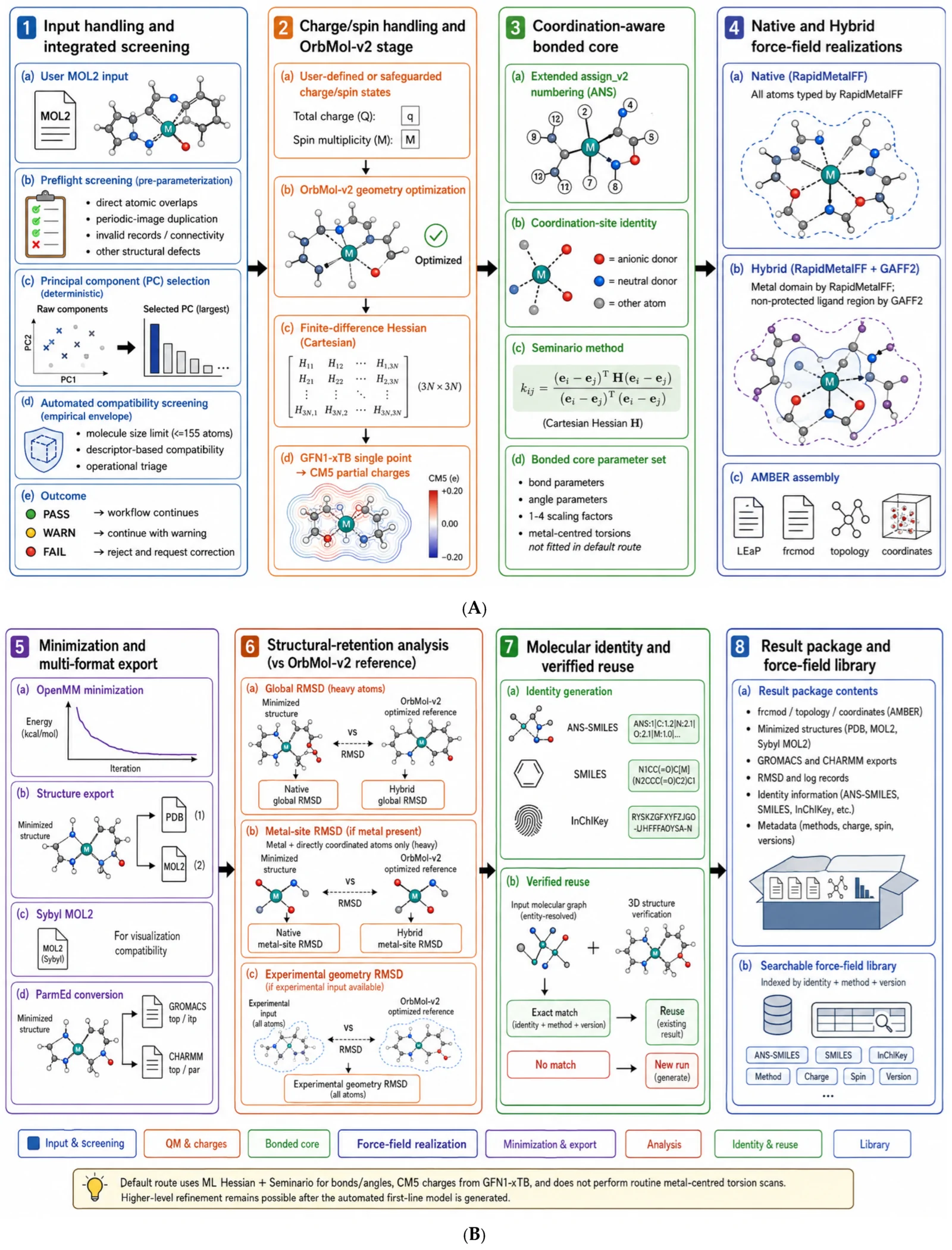
(**A**) RapidMetalFF-AI workflow, Part A (steps 1–4). Continued in (**B**). (**B**) RapidMetalFF-AI workflow, Part B (steps 5–8). Continuation of (**A**).

RapidMetalFF-AI was developed as a modular force-field generation framework for ligand-sized metal-containing molecular systems. The production route integrates MOL2 interpretation, deterministic principal-component selection, principal-component preflight screening, charge/spin safeguarding, OrbMol-v2 geometry optimization, finite-difference Hessian evaluation, extended assign_v2 coordination-site numbering, Hessian-guided bonded-parameter extraction, rapid semiempirical charge assignment, native and Hybrid force-field construction, AMBER assembly, OpenMM minimization, structural-retention analysis, molecular identity generation, verified result reuse, and multi-format export (Figure 2A,B).

The design objective is end-to-end parameter availability rather than acceleration of a single subcalculation. The ML Hessian provides curvature information for bond and angle terms, routine metal-centred torsional scans are omitted from the default automated route, and the default electrostatic layer is produced by a rapid GFN1-xTB/CM5 calculation rather than a system-specific DFT ESP/RESP calculation. Targeted higher-level refinement remains possible after the automated first-line model has been generated.

### 4.2. Input Structure Processing and Charge/Spin Safeguarding

Input structures are supplied as Tripos MOL2 files containing atom identifiers, Cartesian coordinates, atom-type annotations, partial charges, and bond connectivity. Total molecular charge (Q) and spin multiplicity (M) may be supplied explicitly by the user or handled automatically by the workflow. When no explicit molecular charge is provided, RapidMetalFF-AI obtains an integer total charge using Open Babel (version 3.2.1) charge perception [17]. Open Babel is an established open-source cheminformatics toolkit, and its molecular total-charge representation is derived from atomic formal-charge information when the total charge has not been explicitly set. Accordingly, this step is used as a rapid formal-charge perception procedure for automated input handling rather than as an electronic-structure calculation. Spin multiplicity is likewise handled automatically when not supplied by the user, while user-provided Q and M values always take precedence.

Before OrbMol-v2 and GFN1-xTB evaluation, automatically handled state information is subjected to an operational sanity check. Values that cannot be parsed or that fall outside the supported range of −3 ≤ *Q* ≤ +3 and 1 ≤ *M* ≤ 8 invoke a neutral-singlet safeguard (*Q* = 0, *M* = 1). This safeguard is intended only to prevent unusable automatically generated state information from propagating through the computational workflow and should not be interpreted as a chemically validated electronic-state assignment.

The historical exact-82 output set used 64 Q = 0/M = 1, 14 Q = 0/M = 2, and one each of Q/M = −2/1, +3/1, +1/2, and +1/1; 81 selections were automatic and one manual. Stored run metadata and Orb-stage Q/M agreed for all 82 records. The retrospective parity check flagged 145192, while final numbered-MOL2 and native/hybrid topology charge sums differed from stored Q for 850911 and 993277 by −3 and −1 e. These checks establish bookkeeping consistency only. The structure-level diagnoses, corrected 145192 follow-up, and periodic-input classifications are described in Section 3.4 and Supplementary Table S9a. The archived Orb optimization metadata also mark five records as not converged (1518810, 1563993, 2051215, 253279, and 993277); file completion is therefore distinguished from optimization convergence. The reviewed states used in the separate QM/MD follow-up for 902738 (Q = −3, M = 1) and 1100364 (Q = 0, M = 1) remain unchanged.

For the corrected 145192 follow-up, generic atom types were reserved to prevent a numbered carbon type from aliasing HC. Elements were explicitly assigned from the source atom mapping, and a local LEaP build with a maximum of 16 neighbours per atom was used to retain the complete haptic connectivity. The five additional topology corrections use numeric atom identifiers to distinguish duplicate names, and the local LEaP writer retains explicitly mapped element identities rather than replacing them with atom-type inference. Heavy-atom comparisons use source element identities, including Ho as a heavy atom. Atom counts, identities, element numbers, masses, source edges, and topology charge sums were checked after serialization, and both native and hybrid minimized geometries passed structural-integrity checks. The CM5 charge sum was inspected before roundoff correction. These are verified local follow-up safeguards; the historical server outputs are retained for provenance. The additional HF/SDD opt/freq attempt for corrected 145192 stopped at SCF convergence failure; it supplied no completed optimization or frequency result and is excluded from the follow-up metrics.

### 4.3. Principal-Component Preflight Screening and Input Admission Control

Before GPU-intensive parameterization, each submitted MOL2 file passes an integrated preflight screening layer. The same logic is used for public-server submissions to detect direct atom overlaps, duplicate atom identifiers, gross bond/coordinate inconsistencies, disconnected or crystallographic periodic-image components, incomplete hydrogen or valence patterns, severe charge/state inconsistencies, and other structurally unsafe inputs before they consume GPU resources. The layer records an admission decision and concise warning category; it is designed as automated calculation-readiness control rather than as a molecule-specific mechanistic failure analysis.

Crystallographic exports and user-provided MOL2 files can contain multiple molecular records, disconnected counterions, solvent molecules, symmetry-related images, disorder alternatives, or auxiliary fragments. The workflow therefore identifies the molecular entity that will actually enter parameterization. Connected components are ranked deterministically by heavy-atom count, followed by total atom count, bond count, metal-like atom count, and finally the lowest first atom identifier. Only the selected principal component is used for geometry-based gate decisions; non-selected components are excluded from calculation and retained only in the audit record.

High-confidence structural defects are treated as hard failures. These include direct Cartesian atom overlaps within the selected principal component, duplicate atom identifiers that make connectivity ambiguous, and gross inconsistencies between the submitted coordinates and explicitly defined MOL2 bonds. In the current production preflight, a direct overlap is defined by an interatomic distance of 0.30 Angstrom or less. Explicit bonds in the principal component must lie within the deliberately broad 0.50–6.00 Angstrom hard-sanity interval, and the mean explicit-bond distance must remain within the same interval. These limits are structural-sanity safeguards rather than element-specific chemical bond-length models.

Crystallographic periodic-image equivalence is handled separately because it can arise legitimately from crystal-derived structures and therefore is not, by itself, evidence that the molecular input is unusable. When unit-cell information is present, periodic-image repeats and related cell-boundary features are reported as diagnostic warnings rather than automatic failures. Other suspicious but non-definitive contacts are likewise retained in the audit record without necessarily blocking the calculation.

The public server also applies an upper size limit of 155 atoms to the molecular entity selected for calculation. For MOL2 inputs containing multiple molecules or disconnected components, this limit is applied only after deterministic principal-component selection, rather than to the combined atom count of all records in the uploaded file.

Accepted warnings remain attached to the calculation record. The preflight logic is also rechecked in the computational backend as a defence-in-depth safeguard, allowing malformed inputs to be rejected with an explicit explanation before expensive stages while permitting acceptable crystal-derived structures carrying non-fatal warnings to proceed through the normal RapidMetalFF-AI workflow.

### 4.4. Machine Learning Geometry Optimization

Geometry optimization is performed with the OrbMol-v2 machine learning interatomic potential implemented in orb-models (version 0.7.0) through the Atomic Simulation Environment (version 3.29.0) [18]. OrbMol-v2 is trained on OMol25 and OPoly26 [5]. OMol25 documents 83-element coverage from H through Bi (Z = 1–83), including the lanthanides within that atomic-number interval [4]. Actinides such as Th and U are outside this range. The two actinide benchmark records are therefore explicitly treated as extrapolative stress tests rather than evidence of trained-domain actinide accuracy. The selected total charge and spin multiplicity are supplied to the calculator. Structures are relaxed using BFGS optimization with a force-convergence threshold of 0.01 eV Å^−1^ and a maximum of 500 steps.

Device selection is automatic. GPU execution is used when available and compatible; if the GPU backend fails, the calculation can be restarted using a CPU calculator. This operational fallback is intended to preserve workflow completion across heterogeneous deployment environments.

### 4.5. Finite-Difference Hessian Evaluation

After geometry optimization, a Cartesian Hessian is generated by central finite differences in OrbMol-v2 analytical force predictions. Positive and negative displacements of 0.001 Angstrom are applied to each Cartesian coordinate. The resulting matrix is symmetrized before bonded-parameter extraction:

H_sym = (H + H^T^)/2

The maximum pre-symmetrization absolute deviation in H − H^T^ is retained as a numerical diagnostic. This supplies an explicit check on finite-difference consistency before the Hessian is projected into bonded terms.

### 4.6. Native RapidMetalFF Internal Atom Typing

The optimized topology is parsed into a native RapidMetalFF atom-typing layer without requiring antechamber or parmchk2. Sybyl/MOL2 annotations and local connectivity are mapped to chemically interpretable internal classes for common carbon, nitrogen, oxygen, sulfur, phosphorus, halogen, main-group, and metal environments. Compact AMBER-compatible atom types are then written for topology construction.

This internal typing layer defines the native RapidMetalFF representation and provides the parent-type dictionary used for numbered coordination-site types, parameter averaging, mass assignment, and nonbonded inheritance. The Hybrid branch described below deliberately reintroduces GAFF2 for the unprotected ligand region while preserving the native metal-coordination domain.

### 4.7. Extended assign_v2 Metal-Site Numbering

Metal-coordinating atoms are numbered using an extension of the assign_v2 scheme [9]. The key purpose is to retain position-specific identity for non-equivalent donor atoms around a metal centre. This is important in square-planar, octahedral, and multinuclear environments, where donors with the same element or parent chemical type may occupy chemically distinct cis, trans, terminal, or bridging positions.

Without this distinction, generic typing can force distinct metal–ligand bonds or ligand-metal–ligand angles to share averaged parameters. RapidMetalFF-AI evaluates the metal-centred topology first and assigns distinct numbered types to non-equivalent coordinating atoms so that Hessian-derived local terms remain associated with the correct coordination positions. A parent-type mapping preserves the underlying chemical class for mass and nonbonded inheritance.

### 4.8. Bond and Angle Parameter Derivation from the ML Hessian

Bond equilibrium values, angle equilibrium values, and their force constants are derived from the symmetrized ML Hessian using the Seminario projection formalism [7,8,19]. Bond parameters are obtained by projecting the relevant Hessian sub-block onto the bond vector, and angle force constants are obtained from local angular projections. Repeated atom-type patterns can be averaged to produce a compact FRCMOD representation.

Only bond and angle terms are interpreted as Hessian-derived in the present workflow. Proper torsions and impropers are not fitted from the Hessian. Near-linear or numerically degenerate angles that do not yield a stable projected value receive conservative internal fallback terms to preserve topological completeness and allow downstream AMBER assembly.

RapidMetalFF-AI therefore does not propose a new Hessian-to-force-constant formula. Its methodological contribution is the integration of rapid ML-derived curvature, coordination-aware site identity, established Hessian projection, automated force-field construction, and explicit structural validation into a high-throughput workflow.

### 4.9. Native Torsional, Improper, Mass, and Nonbonded Completion

Proper torsional and improper terms outside the metal-centred domain are completed from molecular topology using conservative internal rules. For proper torsions in which a metal–ligand bond forms the central rotational element, RapidMetalFF-AI assigns a zero torsional barrier. This zero-barrier treatment follows the bonded-model strategy adopted in assign_v2 [9] and is consistent with the ZAFF approach, in which the metal-site parameterization was constructed from bond, angle, and electrostatic terms while dihedral terms were omitted [15].

The purpose of the zero-barrier treatment is therefore not to assert that torsional energetics involving metals are physically negligible. Instead, the default RapidMetalFF representation transfers the principal structural description of the immediate coordination environment to explicit metal–ligand bond and ligand–metal–ligand angle terms. In the earlier assign_v2 implementation, the same general strategy was combined with position-specific atom numbering to distinguish cis and trans coordination relationships, and the resulting force fields reproduced the experimental palladium coordination geometries with metal-site RMSDs of 0.0492–0.2976 Å. RapidMetalFF-AI extends this structural principle beyond square-planar palladium systems by combining ANS-based coordination-site identity with bond and angle parameters derived from the OrbMol-v2 Hessian.

This treatment is intended as a rapid first-line bonded representation and does not replace targeted torsional potential-energy-surface fitting when torsional energetics are themselves a scientific observable. Systems whose conformational populations, rotational barriers, or free-energy profiles depend critically on a specific metal-centred torsion remain candidates for subsequent system-specific PES refinement.

Mass and van der Waals parameters are inherited from internal element/type tables. Numbered metal-neighbour types retain a parent internal type so that position-specific bonded identity does not disrupt chemically appropriate mass and nonbonded inheritance.

### 4.10. Hybrid GAFF2 Ligand-Region Completion

RapidMetalFF-AI also generates a complementary Hybrid force-field realization. The protected domain comprises each recognized metal atom and all atoms directly bonded to a metal. Protected atoms retain RapidMetalFF/assign_v2 atom types and metal-associated bonded parameters, whereas unprotected organic ligand atoms are typed and parameterized against GAFF2 [2,3]. Thus, the Hybrid design is explicitly partitioned: metal-coordination environment = RapidMetalFF; remaining organic ligand = GAFF2.

Purely unprotected organic bond and angle interactions are delegated to GAFF2 when a matching parameter is available. Interactions containing a protected atom remain under the RapidMetalFF description, and missing GAFF2 matches fall back to the corresponding RapidMetalFF term. Metal-centred proper torsions are preserved as direction-independent zero definitions, whereas torsions outside the protected metal-coordination domain may be assigned from GAFF2 using exact matches, reverse-order matches, or generic wildcard dihedral terms, i.e., patterns beginning and ending with the wildcard atom type X (just like X-C-C-X).

Atom-type identity is case-sensitive throughout the Hybrid merge. Uppercase RapidMetalFF-derived symbols and lowercase GAFF2 symbols intentionally denote different force-field regions and must not be collapsed. Direction-independent duplicate cleaning protects AMBER continuation terms and removes only genuinely redundant zero-force blocks without merging chemically distinct type identities.

### 4.11. Semiempirical CM5 Charge Assignment and Charge-Failure Handling

Partial atomic charges are assigned by a GFN1-xTB (xtb version 6.7.1) single-point calculation on the OrbMol-v2 optimized geometry using CM5 charges [10,11]. The same total Q and multiplicity M selected above are passed to xTB, with the unrestricted-electron input set as UHF = M − 1. The CM5 column is then written to the MOL2 charge field. Because printed CM5 values can show a small numerical sum drift, the residual Δq = Σi qi − Q is distributed uniformly over the N atoms, qi′ = qi − Δq/N, so that the final partial charges sum exactly to the selected molecular charge. This common protocol was used for the 82 completed benchmark calculations and avoids a separate default DFT electrostatic-potential calculation and RESP fitting step.

If the semiempirical charge calculation does not converge, charge assignment is treated as a separable layer rather than a reason to discard the bonded-parameter set. The workflow can retain the existing MOL2 charge column and records the charge source in the output. This fallback is a robustness mechanism, not a claim of equivalence between CM5 and input charges. Users may substitute system-specific ESP/RESP or other higher-level charges for production applications without changing the generated bonded FRCMOD terms [20].

### 4.12. AMBER Assembly and OpenMM Minimization

The numbered MOL2 and FRCMOD files are assembled with tleap using leaprc.gaff2 to generate Amber (version 23.6) topology and coordinate files. Native RapidMetalFF and Hybrid GAFF2 branches are then minimized in vacuum with OpenMM (version 8.5.2) [12]. The AMBER system uses NoCutoff nonbonded treatment, no bond constraints, no rigid-water constraint, and no centre-of-mass motion removal. Energy minimization uses a maximum of 1000 iterations and a convergence tolerance of 1.0 kJ mol^−1^ nm^−1^; no solvent model or periodic boundary condition is applied.

CUDA (version 12.x) mixed-precision execution is used when available, with CPU and Reference platforms available as operational fallbacks. Minimized coordinates are converted to the numbered MOL2/PDB representations used for validation and export. This short vacuum minimization is a standardized topology/parameter sanity check and should not be interpreted as a substitute for explicit-solvent MD validation.

### 4.13. RMSD Analysis, RapidMetalFF Trust Score, and Experimental Trust

RapidMetalFF-AI automatically evaluates how well the generated force fields retain the optimized molecular geometry after OpenMM minimization by means of RMSD analysis.

For the 82 historical records with complete RMSD outputs, molecule-by-molecule optimized-geometry retention RMSDs together with Experimental Geometry RMSD are reported in Supplementary Table S3. Experimental Geometry RMSD measures the structural change between the CSD-derived experimental starting geometry and the OrbMol-v2 optimized geometry. Orb Global RMSD measures the global deviation between the OrbMol-v2 optimized geometry and the structure obtained after OpenMM minimization with the Orb-derived RapidMetalFF force field, whereas Hybrid Global RMSD reports the same comparison for the Hybrid RapidMetalFF/GAFF2 realization.

When a metal-coordination environment can be identified, two additional RMSD values are calculated using the metal centre or centres together with the atoms directly coordinated to them. Orb Metal-Site RMSD measures structural retention in this local coordination environment for the Orb-derived force field, whereas Hybrid Metal-Site RMSD reports the corresponding value for the Hybrid RapidMetalFF/GAFF2 force field. This separation allows whole-molecule structural retention and direct metal-site retention to be evaluated independently. When a metal-site comparison is not applicable, the corresponding value is reported as N/A rather than being assigned an artificial numerical value.

All RMSD values reported by RapidMetalFF-AI were calculated after least-squares rigid-body alignment to remove differences arising from overall translation and rotation. For each comparison, reference and target coordinates were first centred on their respective geometric centroids, followed by Kabsch/Horn optimal rotational alignment using equal atom weights [21,22]. Global RMSD calculations included only non-hydrogen heavy atoms. Atom correspondence was based on the numbered atom order preserved throughout the RapidMetalFF workflow; structures with mismatched atom counts or order were not silently remapped, element-reordered, or subjected to symmetry-dependent alternative atom matching. Accordingly, each atom in the experimental starting geometry, OrbMol-v2-optimized geometry, and final OpenMM-minimized native RapidMetalFF or Hybrid RapidMetalFF/GAFF2 geometry was compared with its corresponding atom.

For metal-site RMSD calculations, the global heavy-atom set was replaced by the local coordination subset comprising only the defined metal centre or centres and the atoms directly coordinated to them, and the same rigid-body alignment procedure was applied independently to this subset. When a definable metal-coordination subset was unavailable, metal-site RMSD was reported as N/A rather than assigned an artificial numerical value. All 82 structures in the fixed completed benchmark set were included in the global comparisons, whereas the 72 structures satisfying the strict metal-site definition were included in the local coordination analyses.

Molecule-resolved comparisons using the experimental CSD geometry as the direct reference are reported in Supplementary Table S2. It contains experimental/CSD-to-OrbMol-v2 RMSD as a separate optimization diagnostic and the four direct final-force-field RMSDs used by Experimental Trust: experimental/CSD to final native global, final Hybrid global, final native metal-site, and final Hybrid metal-site geometries. The metal-site comparisons are reported for the 72 structures with an applicable coordination subset.

Supplementary Table S3 also reports the Optimized Trust RMSD value (OptimizedTrustRMSD_A). When all four optimized-retention RMSDs are available, this value is the equal-weight arithmetic mean of Orb Global RMSD, Hybrid Global RMSD, Orb Metal-Site RMSD, and Hybrid Metal-Site RMSD. It is therefore a summary RMSD quantity that remains on an Angstrom scale and is not the same quantity as the RapidMetalFF Trust Score reported on a 0–100 scale.

The RapidMetalFF Trust Score reported by the server is derived from the four optimized-geometry retention components: Orb Global, Hybrid Global, Orb Metal-Site, and Hybrid Metal-Site. Because the same absolute RMSD can have a different structural meaning for molecules of different spatial extent, the Trust Score does not use the raw RMSD directly. For each component, the RMSD is normalized by the radius of gyration (Rg) calculated from the exact same atom subset used in that RMSD comparison:

For a component containing N atoms, Rg is the unweighted coordinate radius of gyration of the RMSD reference structure: Rg = [(1/N) Σ_i ||r_i − r_c||^2^]^1/2^, where r_c is the geometric centroid. The atom subset is identical to that used for the corresponding RMSD: non-hydrogen heavy atoms globally or the defined metal-site subset locally. For the RapidMetalFF Trust Score the Rg reference is the OrbMol-v2 optimized geometry; for Experimental Trust it is the verified experimental/CSD input geometry.

*D_i_* = RMSD*_i_*/R*_g_*_,*i*_

The component Trust value is then calculated as:

*T_i_* = 100/(1 + D*_i_*) = 100/[1 + (RMSD*_i_*/R*_g_*_,*i*_)]

Here, T_i is the 0–100 Trust value for the corresponding RMSD component. As RMSD/Rg approaches zero, the Trust value approaches 100; increasing normalized structural deviation lowers the component Trust value.

The overall RapidMetalFF Trust Score is the equal-weight mean of the optimized-retention component Trust values that can be calculated for the molecular system:

*T_overall_* = (1/N) Σ_i=1_*^N^* T*_i_*

Here, N is the number of Trust components available for that molecule. A component that is not applicable is reported as N/A and is excluded from the mean rather than being assigned zero or another artificial value.

Experimental Trust uses the same RMSD/Rg transformation and equal-weight averaging rule but takes the verified experimental/CSD input geometry as the reference. Its four components are experimental/CSD to final native global, final Hybrid global, final native metal-site, and final Hybrid metal-site geometries. Rg is calculated from the experimental/CSD reference using the exact same atom subset as each RMSD, and the overall Experimental Trust is the mean of the available component Trust values; N/A components are excluded.

Experimental Geometry RMSD (experimental/CSD input to OrbMol-v2 optimized geometry) is retained only as a pre-parameterization relaxation diagnostic. It is not a component of either Trust score.

### 4.14. Batch Execution, Logging, and Export

Batch execution tracks each molecule through preflight screening, stage-specific status handling, timeout control, and completion checks. A calculation is counted as structurally completed in the present benchmark only when the supplied final report contains valid global native and Hybrid RMSD values. The preflight/completion-control layer is intended to identify non-computable conditions early and preserves concise admission or warning categories in the benchmark audit; it does not generate a detailed molecule-specific terminal traceback in the summary table. The eight incomplete records are therefore reported at the supported screening-category level without assigning an unsupported downstream mechanism.

Successful AMBER outputs are converted with ParmEd (version 4.3.1), a Python library for reading, modifying, and writing molecular-mechanics representations used by AMBER, GROMACS, CHARMM, and OpenMM [3,23]. Conversion begins from the assembled AMBER topology/coordinate files that completed standardized OpenMM minimization. ParmEd then translates the validated parameter set into the supported syntax, units, and topology conventions required by the corresponding output formats, providing consistent GROMACS- and CHARMM-compatible representations from the same validated AMBER model. These exports retain the originating AMBER-form parameter model; conversion does not constitute reparameterization with native GROMACS force fields or CHARMM/CGenFF conventions. Engine-to-engine energy-component and trajectory equivalence were not evaluated in this study.

### 4.15. Coordination-Aware Molecular Identity and ANS-SMILES

RapidMetalFF-AI generates ANS-SMILES (Advanced Numbering System SMILES), a coordination-aware canonical SMILES-derived line notation. The extended assign_v2 donor numbering is canonicalized to reduce dependence on input MOL2 atom order and is written into SMILES atom-map fields, while metal centres use a separate map-number range. The resulting string remains SMILES-readable while carrying the site identities used by parameterization.

ANS-SMILES is a searchable identifier rather than a stand-alone proof of molecular identity. The production identity layer independently builds topology, coordination, geometry, and calculation keys from the molecular graph, metal–donor environment, three-dimensional geometry, method version, charge, and spin. Reuse is accepted only after exact graph-isomorphism and three-dimensional alignment verification; equality of ANS-SMILES or a single hash is not sufficient by itself.

### 4.16. Web-Server Deployment, Library Identity Checks, and Verified Reuse

RapidMetalFF-AI is deployed at https://rapidmetalff-ai.biochemoinfo.com. The public interface supports submission of new MOL2 structures together with access to the server library, which users can browse at https://rapidmetalff-ai.biochemoinfo.com/search.php (accessed on 19 August 2026). After preflight admission and before a new expensive calculation is launched, the submitted molecular structure is checked against previously validated records in this library.

This identity check is deliberately stricter than a loose lookup based only on molecular formula or InChIKey. Standard molecular identifiers are used together with ANS-SMILES and metal-sensitive topology, coordination, and three-dimensional geometry descriptors. ANS-SMILES contributes coordination-site identity, while exact computational reuse is protected by stricter molecular-graph and three-dimensional verification. Thus, for example, the presence of cisplatin in the library does not cause a transplatin submission with the same molecular formula to be returned as the same calculation.

If no verified match is found in the first identity check, OrbMol-v2 geometry optimization is initiated. After optimization and before the Hessian calculation, the optimized structure is checked against the library again. This second identity check is intentional: different starting geometries can converge during OrbMol-v2 optimization to the same or an equivalent computed structure. When such a verified post-optimization match is found, the existing parameterization can be reused and unnecessary Hessian and force-field calculations are avoided. If no match is found, the calculation proceeds to Hessian generation and subsequent parameterization.

Result packages contain generated parameter files, optimized and numbered structures, AMBER validation outputs, RMSD and log records, and supported exports, providing a reproducible calculation record through the public service.

### 4.17. CSD-Derived Benchmark Selection and Statistical Analysis

The benchmark set was constructed by manually selecting 90 structures from the Cambridge Structural Database (CSD), the majority of which were metal-containing, while also including structures comprising organic ligands. The purpose of this selection was not to reproduce the statistical composition of the CSD, but to stress the automated workflow across as broad a range of metal-containing chemical space as practicable. Structures containing different metal elements and chemically diverse metal complexes were selected individually, while both very small species and excessively large molecular systems outside the intended operational scale of the public server were avoided. The resulting collection should therefore be interpreted as a deliberately heterogeneous stress-test set rather than as a random or frequency-weighted sample of the CSD.

Structural benchmark outcomes were summarized descriptively using medians, interquartile ranges (IQRs), ranges, and the proportions of structures below the 0.5 and 1.0 Angstrom RMSD thresholds. Runtime analysis used the exact same 82 structures that satisfied the completion criteria used for the structural benchmark. End-to-end runtime and stage-resolved timings were extracted from the calculation metadata for each completed run and summarized using mean, median, standard deviation (SD), IQR, and range. These timing measurements were obtained in the local benchmark environment using an NVIDIA GeForce RTX 4060 Laptop GPU. Molecule-resolved timing data and the corresponding descriptive summary are provided as Supplementary Tables S4 and S5, respectively.

### 4.18. Independent QM Comparison

The independent-QM set comprised 1101146 (Nb), 155447 (Pt), 902738 (Mo), 1100364 (Sc), 232528 (Au), 613741 (In), 163152 (Ga), 234071 (Bi), and 179921 (Sn). GAMESS-US (version July 15, 2024 R2 Patch 1; Iowa State University, Ames, IA, USA) [24] calculations were run independently of OrbMol-v2 using HF/SDD geometry optimization and frequency analysis. Normal termination and the absence of imaginary frequencies were verified for every reference. Atom counts, element order, connectivity, charge, and multiplicity correspondence were checked before comparison. Heavy-atom and metal-site RMSDs were calculated after independent least-squares alignment. Metal–ligand bond-length MAE was the mean absolute difference over directly bonded metal–donor pairs, and coordination-angle MAE was calculated over donor–metal–donor angles.

For follow-up comparisons, 902738 used Q = −3/M = 1 and 1100364 used Q = 0/M = 1 after review of their original automatically assigned doublet states (for 1100364, missing hydrogen atoms were added before the follow-up QM calculations). The fixed exact-82 tables and aggregates retain their original benchmark records; revised states are confined to Supplementary Tables S7 and S8 and the follow-up analyses.

### 4.19. Short Explicit-Solvent MD Protocol

Ten RapidMetalFF systems were solvated with TIP3P water using a 10 Å padding distance and periodic boundary conditions. Particle-mesh Ewald electrostatics were used; the Q = −3 902738 system was neutralized with three Na+ ions. OpenMM 8.1.1.dev-ec797ac was run on CUDA in mixed precision. After energy minimization, each system underwent 50 ps heating, 100 ps NPT equilibration, and 5 ns NPT production at 300 K and 1 bar using a 0.5 fs unconstrained time step. Coordinates were saved every 2 ps, yielding 2500 production frames per system. Heavy-atom and metal-site RMSDs were calculated after separate least-squares alignment to the first production frame.

Default RapidMetalFF GFN1-xTB/CM5 charges were used for 1101146, 155447, 902738, 1100319, 232528, 613741, 163152, 234071, and 179921. For 1100364, the GFN1-xTB charge parser failed and the raw output contained zero charges; GFN2-xTB partial charges [13] were therefore used locally while the RapidMetalFF bonded, torsional, and Lennard-Jones terms were retained. This substitution is stated explicitly because the 1100364 trajectory is not a test of the unmodified default electrostatic route. The short trajectories assess immediate structural stability only and were not used to estimate equilibrium free energies, binding affinities, spectra, or converged radial-distribution functions.

### 4.20. RapidMetalFF-AI Output Files

For this study, “simulation-ready” is an operational file-level definition: the generated MOL2/FRCMOD pair can be assembled by tleap into complete AMBER topology and coordinate files, and those files complete standardized OpenMM minimization without missing parameters or topology errors detected by the assembly/minimization checks. Successful assembly alone does not establish preservation of every source connectivity edge or chemical-state consistency; retrospective exceptions are reported in Section 3.4 and Section 4.2. As described in Section 4.14, the AMBER model validated by OpenMM minimization is then exported through ParmEd’s established AMBER/GROMACS/CHARMM conversion pathways, allowing the same parameter set to be distributed in the principal formats used by downstream simulation workflows. The downloadable ZIP preserves the processed structures, parameters, validation outputs, identity records, intermediate data, and stage-specific logs needed for further inspection.

A central feature of the output package is that the generated force-field parameters are not delivered as untested FRCMOD files alone. After the native RapidMetalFF and Hybrid RapidMetalFF/GAFF2 FRCMOD files have been constructed, each parameter set is assembled into an AMBER-compatible molecular system and the corresponding structure is subjected to OpenMM energy minimization. The resulting minimized geometries therefore provide the structures actually obtained after applying the generated force field, rather than only the reference geometry from which the parameters were derived.

For both the native and Hybrid branches, the minimized structure is retained in several complementary representations. Numbered MOL2 files preserve the RapidMetalFF/ANS atom identities used during parameterization and allow the final coordinates to be traced directly back to the generated bonded terms. Because MOL2 files containing the internal numbered atom types are not interpreted correctly by all molecular-visualization programs, RapidMetalFF-AI additionally generates visualization-oriented Sybyl MOL2 copies without the internal ANS numbering, together with conventional PDB representations of the minimized structures. AMBER topology and coordinate files are also retained for direct simulation use.

Structural validation results accompany these minimized geometries. RMSD values are calculated between the relevant reference structure and the final minimized native and Hybrid geometries, with separate global and metal-site comparisons where applicable. The corresponding RMSD records, structural-integrity reports, and atom-mapping information are included in the result package so that users can inspect not only the generated parameters but also the structural behaviour produced by those parameters after minimization.

The package additionally retains the OrbMol-v2 optimized geometry and Cartesian Hessian used for bonded-parameter derivation, the ANS-numbered input used by the force-field construction stage, molecular-identity files, calculation metadata, charge-assignment records, and the native and Hybrid force-field files. Successful parameterizations are further exported to supported AMBER-, GROMACS-, and CHARMM-compatible representations, while stage-specific logs provide an audit trail for preprocessing, OrbMol-v2 optimization and Hessian generation, parameter construction, charge assignment, AMBER/OpenMM processing, Hybrid generation, identity analysis, and final export.

A complete directory tree and file-by-file description of the RapidMetalFF-AI result package, including the individual intermediate, validation, visualization, conversion, and log files, are provided in Supplementary Table S6.

### 4.21. ANS, ANS-SMILES, and Standard SMILES Representation

The Advanced Numbering System (ANS) used in RapidMetalFF-AI is an extended form of the numbering approach previously introduced in the assign_v2 study [9]. Although the method was demonstrated in assign_v2 using square-planar palladium complexes, the fundamental principle of the numbering system is not specific to palladium. The purpose of the approach is to distinguish the positional identities of atoms bound to a metal centre that may otherwise belong to the same or similar chemical classes under conventional atom typing. In this way, distinct metal–ligand bonds and ligand–metal–ligand angles that cannot be differentiated solely on the basis of elemental identity or generic atom type can be represented by separate bonded parameters.

In assign_v2, this approach was used particularly to distinguish cis and trans positions in square-planar systems. By numbering the atoms around the metal in a defined sequence, adjacent and opposite positions could be distinguished, allowing the same conventional atom type to use different angle parameters in different geometric environments. The numbering procedure is therefore not merely a cosmetic renaming of atoms, but rather a positional-identity mechanism that transfers coordination geometry into the bonded-force-field representation. In the assign_v2 study, force constants for metal-containing dihedrals were set to zero, while preservation of the geometry around the metal was achieved primarily through position-specific metal–ligand bond and ligand–metal–ligand angle parameters.

ANS provides two major advantages over the previous numbering system. The first concerns numbering capacity. In the earlier implementation, the single-character numerical numbering scheme limited the system to a maximum of nine distinct numbered positions. ANS expands this numbering space while maintaining compatibility with two-character AMBER atom types. Whereas the original numbering system could describe up to nine atoms around metal centres, ANS extends this capacity to 35 by additionally using alphanumeric characters. Consequently, up to 35 distinct metal-neighbour atoms within a single molecular system can be represented by unique ANS identities.

This extension is particularly important for structures containing multiple metal centres, high coordination numbers, or numerous bridging ligands. RapidMetalFF-AI can evaluate multiple metal centres within the same molecular topology; when a donor atom bridges two or more metal centres, it retains the ANS identity already assigned to it and is not renumbered as an independent atom type for each additional metal. Similarly, in systems exhibiting haptic coordination, ring atoms associated with the metal are handled in a manner that preserves their unique positional identities. ANS therefore extends the same positional-identity principle from relatively simple mononuclear coordination geometries to substantially more complex multinuclear and highly bridged molecular systems.

This expanded positional resolution is also important for the treatment of metal-centred dihedrals in RapidMetalFF-AI. In the native RapidMetalFF model, no active torsional barrier is applied to metal-centred proper torsions in which the metal occupies the second or third position of the proper dihedral; these terms are defined with a zero-energy barrier. Preservation of the metal-coordination geometry is instead achieved through position-specific bonded parameters derived from the OrbMol-v2 Hessian for ANS-distinguished metal–ligand bonds and ligand–metal–ligand angles. By distinguishing as many as 35 different metal-neighbour positions, ANS prevents distinct local coordination relationships from collapsing into the same generic atom type even in highly complex systems containing numerous donors and bridging atoms. Consequently, a broad range of coordination topologies can be represented without requiring routine, separate metal-centred torsional potential-energy-surface scans merely to preserve geometric differentiation around the metal.

This does not mean that all dihedral terms in the molecule are removed. Metal-centred proper dihedrals are retained with zero barriers, whereas organic torsions outside the metal-coordination region are treated separately. In the native RapidMetalFF branch, ligand torsions are completed using internal rules, whereas in the Hybrid RapidMetalFF/GAFF2 branch, appropriate ligand torsions outside the protected metal region are obtained from GAFF2. Thus, the purpose of ANS is not to replace general organic conformational energetics with bond and angle terms, but rather to make preservation of metal-coordination geometry independent of mandatory metal-centred torsional fitting.

The second major advantage of ANS over the original numbering system is that its coordination-site numbering is designed to be independent of the local traversal direction. ANS assigns persistent and unique identities to metal-coordinating atoms, including donors shared between multiple metal centres. For molecular-identity generation, this provisional numbering is subsequently canonicalized using invariants derived from molecular topology and geometry, thereby reducing dependence on arbitrary MOL2 atom ordering and on the local traversal direction.

The primary objective of canonical ANS generation is to reduce dependence on arbitrary MOL2 atom ordering while preserving the coordination relationships represented by the numbering system. To achieve this, metal centres and their coordinating donor atoms are hierarchically reordered based on sequence-independent structural criteria, including atomic identity, chemical class, surrounding connectivity, and the three-dimensional coordination geometry of the metal. When symmetric or equivalent cases arise, standard graph-ranking rules resolve the ambiguity to establish a single, deterministic order.

Numbering is then assigned sequentially according to this standardized sequence. A donor atom bridging multiple metals retains the unique ANS identity assigned upon its first canonical encounter, rather than being renumbered for each metal centre. ANS numbering is standardized using topology- and geometry-based criteria so that coordination-site relationships, including adjacent/opposite and bridging relationships, are represented independently of the arbitrary traversal of the uploaded MOL2 structure.

The coordination-site identity generated by ANS is initially used within MOL2 and FRCMOD files for force-field parameterization. However, RapidMetalFF-AI also contains a persistent molecular library in which previously calculated force fields can be searched and verified results can be reused. Retaining this information only within force-field files would therefore be insufficient. For this purpose, ANS-SMILES was developed.

Standard SMILES [25] is a widely used and portable representation for compactly encoding molecular connectivity and chemical graphs. RapidMetalFF-AI continues to use conventional identifiers such as standard SMILES, canonical SMILES, and InChIKey [26]. However, these identifiers do not inherently contain the parameterization-specific positional identity defined by ANS. Standard SMILES is not intended to encode which donor atoms in a metal complex correspond to particular ANS positions, which atom serves as a bridge to another metal centre, or which numbered coordination-site identities are associated with particular bonded parameters.

ANS-SMILES adds this missing layer to the standard SMILES representation. RapidMetalFF-AI first canonicalizes the ANS numbering to reduce dependence on the arbitrary atom order present in the input MOL2 file. The canonical ANS donor numbers are then transferred to the atom-map fields of the SMILES representation, while metal centres are represented using a separate map-number range that does not overlap with the donor numbering. The resulting representation therefore remains SMILES-compatible while simultaneously retaining the coordination-site information used during force-field generation.

ANS-SMILES is not intended to replace canonical SMILES or InChIKey. Conventional identifiers are retained for general chemical identity, interoperability between software packages, and conventional molecular searching, whereas ANS-SMILES adds a coordination identity that is meaningful in the context of force-field parameterization. This makes it possible to search the RapidMetalFF-AI library not only for structures sharing the same general molecular graph, but also for coordination arrangements defined by ANS.

Nevertheless, ANS-SMILES alone is not treated as a definitive identity certificate that automatically permits reuse of a previously calculated force field. ANS-SMILES serves as a coordination-aware search layer for identifying candidate matches. Before a previous calculation can be reused, RapidMetalFF-AI additionally applies independent identity checks involving the molecular graph, metal–donor topology, three-dimensional geometric agreement, total charge, spin state, and method version. Accordingly, ANS-SMILES provides the searchable coordination identity, whereas the stricter graph-based and three-dimensional checks provide the verified computational-reuse layer.

From this perspective, ANS is not merely the assign_v2 numbering concept applied to different metals. The new system expands the number of distinguishable coordination positions from 9 to 35, handles multimetallic and bridged topologies within a single consistent numbering framework, propagates this identity into Hessian-derived bond and angle parameters, and finally incorporates the same information into searchable molecular identity through ANS-SMILES.

**Figure 3:**
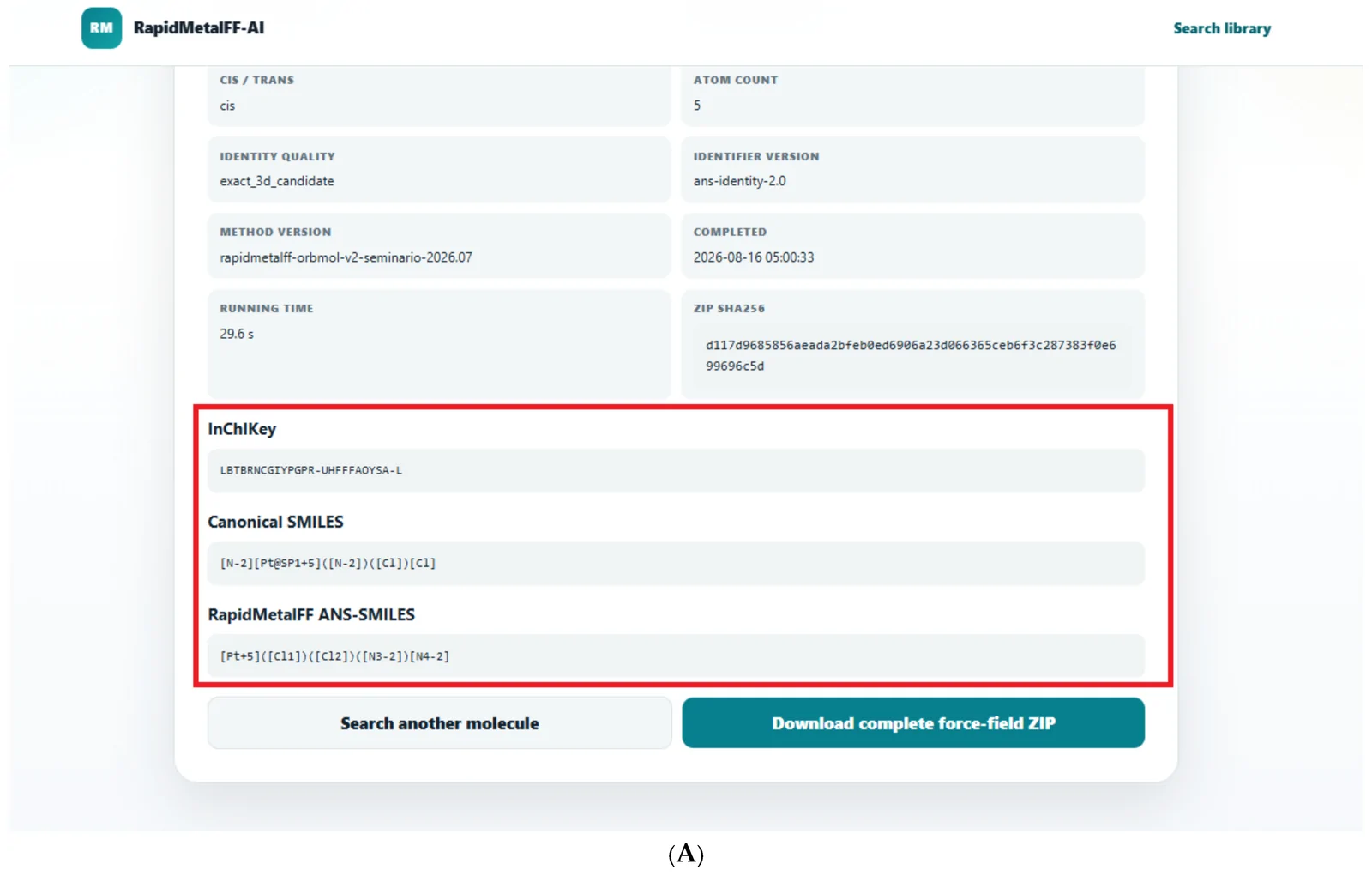

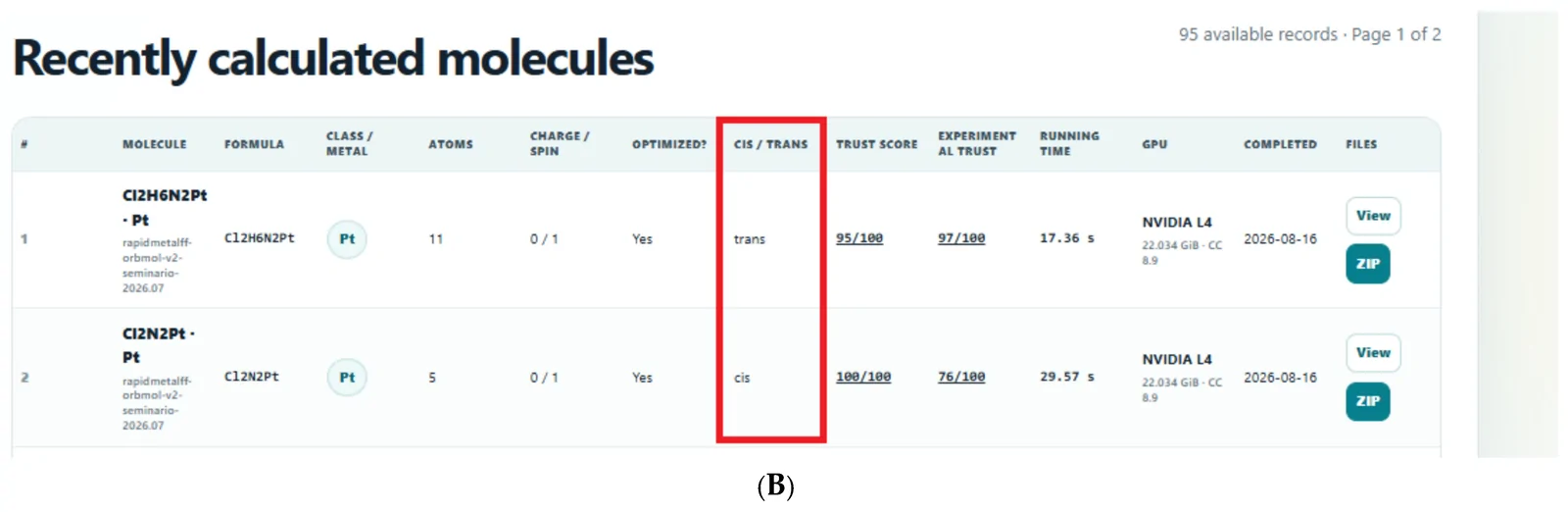
(**A**) The RapidMetalFF-AI server automatically assigns SMILES and ANS-SMILES identifiers to molecules. (**B**) Coordination-aware cis/trans representation in the RapidMetalFF-AI public server. The panels show the canonical SMILES and ANS-SMILES for CSD 1133046 (cisplatin) and CSD 1576669 (transplatin), together with the corresponding library cis/trans classification. Consecutive ANS donor positions (Cl1–Cl2) denote the cis arrangement, whereas opposite positions (Cl1–Cl3) denote the trans arrangement. The red box in panel (**A**) highlights the molecular identifiers (InChIKey, canonical SMILES, and ANS-SMILES), while the red box in panel (**B**) highlights the cis/trans classification column, showing “cis” for CSD 1133046 and “trans” for CSD 1576669.

The ability of ANS-SMILES to preserve and directly expose cis/trans coordination relationships can also be verified through the public RapidMetalFF-AI server. During manuscript preparation, two structures retrieved from the CSD by searching only for “cisplatin” and “transplatin”, CSD 1133046 and CSD 1576669, respectively, were submitted to the server without any manual modification or preprocessing (Figure 3). RapidMetalFF-AI automatically generated distinct coordination-aware representations for the two square-planar Pt complexes.

For the **cis** example, CSD 1133046, the canonical SMILES was [N-2][Pt@SP1+5]([N-2])([Cl])[Cl], whereas the corresponding RapidMetalFF ANS-SMILES was [Pt+5]([Cl1])([Cl2])([N3-2])[N4-2]. Although the canonical SMILES contains the square-planar stereochemical descriptor @SP1, recognition of the cis arrangement requires interpretation of this specialized stereochemical notation. In the ANS-SMILES representation, the relationship is directly visible from the coordination-site numbering itself: the two chlorine atoms coordinated to Pt occupy consecutive ANS positions, **Cl1 and Cl2**, indicating that they are adjacent around the square-planar metal centre and therefore correspond to the **cis** arrangement. The same pattern is seen for the two coordinated nitrogen atoms, which occupy the consecutive positions N3 and N4.

For the **trans** example, CSD 1576669, the canonical SMILES was [H][N+]([H])([H])[Pt@SP3+5]([Cl])([Cl])[N+]([H])([H])[H], whereas the corresponding RapidMetalFF ANS-SMILES was [H][N2+]([H])([H])[Pt+5]([Cl1])([Cl3])[N4+]([H])([H])[H]. In this case, the two chlorine atoms no longer occupy consecutive ANS positions. Instead, they occur at **Cl1 and Cl3**, with one coordination position separating them in the ANS sequence. Their placement at positions 1 and 3 therefore identifies them as opposite positions around the square-planar Pt centre, corresponding to the **trans** arrangement. Likewise, the coordinated nitrogen atoms occupy the opposite positions N2 and N4.

Thus, the distinction can be read directly from the ANS positional sequence: Cl1–Cl2 represents adjacent coordination positions and therefore the cis arrangement, whereas Cl1–Cl3 represents opposite coordination positions and therefore the trans arrangement. ANS-SMILES consequently does not merely preserve stereochemical information through a specialized stereochemical descriptor; it makes the coordination relationship explicitly readable through the same positional identities used during RapidMetalFF bonded-force-field parameterization. Both examples and their corresponding molecular-information records can be independently inspected and reproduced through the publicly accessible RapidMetalFF-AI server (Figure 3).

### 4.22. Web-Based Trust Score and Experimental Trust Reporting

RapidMetalFF-AI does not replace the underlying RMSDs with a single success score. The server additionally reports size-normalized Trust values as complementary diagnostics. The RapidMetalFF Trust Score uses the OrbMol-v2 optimized geometry as reference and summarizes final native and Hybrid retention after OpenMM minimization. Experimental Trust uses a verified experimental/CSD input geometry as reference and summarizes the direct deviations of those same final native and Hybrid geometries from the experimental input. In both cases, Trust is derived from RMSD/Rg and is not a probability of correctness.

Supplementary Table S3 reports the optimized-reference retention RMSDs, whereas Supplementary Table S2 reports the direct experimental-reference final-geometry RMSDs. The public library keeps these reference frames separate through the RapidMetalFF Trust Score and, for administrator-verified experimental inputs, Experimental Trust; otherwise Experimental Trust is N/A.

Selecting the Trust Score link opens a dedicated optimized-geometry Trust analysis page. Its four components are final native global, final Hybrid global, final native metal-site, and final Hybrid metal-site, all referenced to the OrbMol-v2 optimized geometry. Global components use non-hydrogen heavy atoms, whereas metal-site components use the metal centre(s) plus directly coordinated atoms.

For every available component, the detail page exposes the RMSD, the radius of gyration (Rg), the normalized RMSD/Rg ratio, the resulting component Trust value, the number of atoms used, and the reference geometry. Rg is calculated from the exact same atom subset used for the corresponding RMSD. Thus, global comparisons use the global atom subset for both RMSD and Rg, whereas metal-site comparisons use the identical restricted metal-site subset for both quantities. As defined in Section 4.13, this is a geometric, equal-atom-weight coordinate Rg and is not mass-weighted.

Experimental Trust is kept separate because it uses a different reference geometry. It is enabled only for records whose uploaded geometry has been administrator-verified as experimental. The dedicated Experimental Trust page reports the overall score, available components, calculation time, and calculator version.

Experimental Trust contains four direct comparisons from the verified experimental/CSD input to the final minimized force-field geometries: native global, Hybrid global, native metal-site, and Hybrid metal-site. Thus every component begins at the same experimental/CSD reference and ends at a final native or Hybrid geometry; OrbMol-v2 optimized geometry is never the reference of an Experimental Trust component.

For each Experimental Trust component, the page reports RMSD, experimental-reference Rg, RMSD/Rg, component Trust, atom count, and reference geometry. The normalization and aggregation procedure follows the definition in Section 4.13, with Rg calculated from the experimental/CSD reference. The overall Experimental Trust is the mean of available component Trust values. Experimental/CSD-to-OrbMol-v2 RMSD remains diagnostic only and is never included. Together, Supplementary Tables S2 and S3 preserve the experimental-reference and optimized-reference RMSDs, respectively, while the corresponding web-based Trust analyses expose the normalization, atom subsets, component availability, calculator version, and record-specific scores.

## 5. Conclusions

RapidMetalFF-AI makes three linked contributions: (i) minute-scale AI-assisted bonded-force-field generation from OrbMol-v2 geometry and finite-difference Hessian information; (ii) extended assign_v2/ANS coordination-aware identity that preserves non-equivalent donor positions in atom typing and ANS-SMILES; and (iii) integration of these components with charge handling, native and Hybrid force-field construction, AMBER assembly, structural validation, ParmEd-based multi-format export, public web delivery, and verified library reuse.

The deliberately heterogeneous benchmark began with 90 manually selected, unedited CSD-derived MOL2 structures and is a stress test rather than a frequency-weighted representation of the CSD. Eighty-two calculations produced complete final records; 72 had recognized metal-site subsets. Optimized-reference metrics test reconstruction of the OrbMol-v2-derived minimum and are partly self-referential, whereas the direct CSD comparisons use an experimental reference. Median native/Hybrid optimized-reference global RMSDs were 0.430/0.415 Å and metal-site medians were 0.079/0.107 Å; direct experimental-reference global medians were 0.626/0.623 Å and metal-site medians were 0.176/0.166 Å.

Nine representative systems with completed independent HF/SDD calculations provided a third structural layer: the native models gave median heavy-atom and metal-site RMSDs of 0.333 and 0.101 Å, a median metal–ligand bond-length MAE of 0.074 Å, and a median coordination-angle MAE of 2.865°. All nine reference optimizations were confirmed as true minima by frequency calculations without imaginary modes. Ten 5 ns explicit-water production trajectories provided a dynamical structural check, with a median time-averaged metal-site RMSD of 0.112 Å across 50 ns of aggregate sampling. Together, these results support local structural retention and nanosecond-timescale stability within the validation domain defined in Section 3.9.

From a computational-performance perspective, the principal gain of RapidMetalFF-AI is not merely its mean end-to-end runtime of 65.5 s and median runtime of 48.9 s, but its ability to integrate geometry optimization, Hessian generation, and subsequent parameter construction—steps that traditionally represent major time and computational costs in bonded-force-field parameterization—into a single automated workflow with a typical runtime below one minute. Although most of the total runtime is still associated with OrbMol-v2 geometry optimization and finite-difference Hessian evaluation, the short times required for parameter generation, force-field assembly, OpenMM minimization, and output generation indicate that these stages no longer constitute the principal bottlenecks of the workflow. In this way, a conventionally lengthy and multistep parameterization process for metal-containing molecules is transformed into a minute-scale workflow suitable for high-throughput calculations.

Future development will focus on removing the molecule-specific numbering requirement by moving toward a directly AMBER-compatible general parameter representation, with the same framework potentially extending to systematic force-field correction in proteins, DNA, and RNA.

## Figures and Tables

**Table 1:** Descriptive structural-retention statistics for the 82 completed CSD-derived calculations. Metal-site and Optimized Trust RMSD statistics use the 72 records with recognized metal-site subsets.

Metric	N	Mean (Å)	SD (Å)	Median (Å)	IQR (Å)	≤0.5 Å	≤1.0 Å	Range (Å)
CSD input → Orb optimized	82	0.412	0.421	0.278	0.137–0.534	58/82 (70.7%)	76/82 (92.7%)	0.023–2.532
Native global	82	0.530	0.395	0.430	0.221–0.795	48/82 (58.5%)	71/82 (86.6%)	0.046–1.788
Hybrid global	82	0.548	0.521	0.415	0.228–0.697	49/82 (59.8%)	71/82 (86.6%)	0.054–3.742
Native metal-site	72	0.247	0.426	0.079	0.046–0.267	63/72 (87.5%)	67/72 (93.1%)	0.006–2.227
Hybrid metal-site	72	0.316	0.718	0.107	0.049–0.249	63/72 (87.50%)	67/72 (93.1%)	0.013–4.838
Optimized Trust RMSD	72	0.419	0.476	0.284	0.194–0.469	55/72 (76.4%)	67/72 (93.1%)	0.049–3.149

Note: N/A metal-site values were excluded rather than treated as zero. Mean and SD are sample descriptive statistics across the applicable records. The 0.5 and 1.0 Å levels are descriptive reference thresholds, not benchmark acceptance criteria.

**Table 2:** Representative completed benchmark cases. OTR denotes the Å-scale Optimized Trust RMSD and is distinct from the 0–100 RapidMetalFF Trust Score.

CSD ID	ExpGeom	Native G	Hybrid G	Native M	Hybrid M	OTR	Screen
232528	0.047	0.066	0.054	0.048	0.029	0.049	PASS
2104359	0.055	0.072	0.135	0.054	0.091	0.088	PASS
243849	0.291	1.036	0.274	0.032	0.023	0.341	PASS
2074417	0.484	1.469	0.539	0.032	0.069	0.527	PASS
154784	1.738	0.840	1.152	0.394	0.350	0.684	WARN
716039	0.501	1.317	1.596	1.429	1.970	1.578	WARN
942961	0.237	1.788	3.742	2.227	4.838	3.149	WARN
993277	1.441	1.260	1.774	1.982	3.132	2.037	WARN
1100181	0.665	0.983	1.008	1.100	1.139	1.058	PASS
850911	1.131	1.517	1.536	1.418	1.439	1.477	WARN

**Table 3:** End-to-end and stage-resolved runtime statistics for the exact 82 completed benchmark calculations on an NVIDIA GeForce RTX 4060 Laptop GPU.

Metric	N	Mean (s)	Median (s)	SD (s)	IQR (s)	Range (s)
End-to-end runtime	82	65.51	48.92	46.85	38.37–77.69	27.18–266.07
MOL2 preflight	82	0.11	0.11	0.04	0.07–0.12	0.06–0.27
OrbMol-v2 optimization	82	29.11	23.51	18.46	17.06–34.10	11.89–118.77
Post-OrbMol structural integrity	82	0.20	0.19	0.05	0.16–0.21	0.13–0.43
Orb finite-difference Hessian	82	17.39	9.89	20.39	6.50–20.52	1.49–108.26
RapidMetalFF parameter construction	82	0.38	0.28	0.39	0.26–0.38	0.23–3.15
GFN1-xTB/CM5 charge	82	5.40	0.76	25.54	0.61–1.63	0.42–172.86
FF assembly + OpenMM	82	3.65	2.83	3.45	2.51–3.80	1.96–32.73
Multi-format export	82	0.63	0.52	0.28	0.49–0.57	0.32–1.90
Sum of listed stages	82	56.86	39.74	46.54	30.74–66.95	19.60–259.32
Unlisted/orchestration runtime	82	8.65	7.90	1.72	7.47–9.84	6.76–14.63
Complete Orb stage	82	53.00	41.11	35.70	32.11–62.44	21.14–219.42

**Table 4:** Native RapidMetalFF final geometries compared with independent QM-optimized references.

CSD ID	Metal	Q/M	QM Level	Native Heavy RMSD (Å)	Native Site RMSD (Å)	Bond MAE (Å)	Angle MAE (°)
1101146	Nb	0/1	HF/SDD	0.343	0.198	0.122	4.588
155447	Pt	0/1	HF/SDD	0.910	0.120	0.131	0.538
902738	Mo	−3/1	HF/SDD	0.610	0.177	0.149	2.865
1100364	Sc	0/1	HF/SDD	0.333	0.058	0.003	1.926
232528	Au	0/1	HF/SDD	0.098	0.091	0.098	0.933
613741	In	0/1	HF/SDD	0.178	0.094	0.043	3.529
163152	Ga	0/1	HF/SDD	0.278	0.105	0.074	3.769
234071	Bi	0/1	HF/SDD	0.120	0.045	0.031	1.303
179921	Sn	0/1	HF/SDD	0.851	0.101	0.011	4.509

**Table 5:** Explicit-water production-MD RMSD summary (5 ns; 2500 sampled frames per system).

CSD ID	Metal	Q/M	Global RMSD Mean ± SD (Å)	Global Max (Å)	Site RMSD Mean ± SD (Å)	Site Max (Å)
1101146	Nb	0/1	0.554 ± 0.168	0.929	0.191 ± 0.037	0.339
155447	Pt	0/1	0.570 ± 0.138	1.047	0.083 ± 0.019	0.181
902738	Mo	−3/1	0.775 ± 0.289	1.158	0.105 ± 0.021	0.178
1100319	Be	0/1	0.560 ± 0.139	1.083	0.089 ± 0.022	0.181
1100364	Sc	0/1	0.572 ± 0.127	0.984	0.114 ± 0.023	0.206
232528	Au	0/1	1.166 ± 0.598	2.047	0.138 ± 0.040	0.324
613741	In	0/1	0.371 ± 0.112	0.812	0.182 ± 0.040	0.350
163152	Ga	0/1	1.126 ± 0.353	1.855	0.109 ± 0.030	0.235
234071	Bi	0/1	0.523 ± 0.290	1.145	0.105 ± 0.028	0.212
179921	Sn	0/1	1.761 ± 0.376	2.704	0.117 ± 0.028	0.228

## Data Availability

Supplementary Tables S1–S6 provide the original 90-input disposition, exact-82 structural metrics, timing data, and package inventory. Supplementary Table S7 provides the nine-system independent HF/SDD comparison; Table S8 reports the ten 5 ns explicit-water MD tests; and Table S9 reports charge, multiplicity, propagation, parity, charge-sum, and fallback warnings for every benchmark record where completed metadata were available. Experimental Trust values and calculation-specific charge/spin metadata are also exposed through the corresponding public molecular-catalogue records. Raw CSD-derived structural files are not redistributed where CSD/CCDC licensing restrictions apply. Validated force-field packages indexed in the RapidMetalFF-AI library are accessible through the public server, and additional generated outputs can be supplied subject to licensing and repository constraints.

## Supplementary Materials

The following supporting information can be downloaded at: https://www.mdpi.com/article/10.3390/molecules31203724/s1. Table S1: Input screening results for all 90 CSD structures, including atom counts, selected molecular components, structural warnings, and final processing status. Table S2: Comparison of experimental CSD geometries with OrbMol-v2 optimized structures and final native RapidMetalFF and Hybrid force-field structures. Includes molecular identifiers, global and metal-site RMSDs, and metal-site atom information for the 82 completed calculations. Table S3: Molecular identifiers (SMILES and ANS-SMILES), experimental-to-OrbMol-v2 RMSDs, native RapidMetalFF and Hybrid optimized-reference global and metal-site RMSDs, Optimized Trust RMSDs, metal-site atom counts, and calculation status for the 82 completed structures. Table S4: Calculation times for each of the 82 completed structures, including total runtime, time spent at each processing stage, GPU information, and calculation metadata files. Table S5: Statistical summary of calculation times for the 82 completed structures, including mean, median, standard deviation, quartiles, and minimum and maximum runtimes for each processing stage. Table S6: List and explanation of the files and folders included in a RapidMetalFF-AI result package, covering input structures, optimized geometries, Hessians, force-field parameters, validation results, molecular identifiers, converted outputs, and calculation logs. Table S7: Comparison of OrbMol-v2, native RapidMetalFF, and Hybrid geometries with independent HF/SDD reference calculations for nine metal-containing structures, including global and metal-site RMSDs, metal–ligand bond-length errors, coordination-angle errors, and vibrational-frequency results. Table S8: Simulation settings and results for ten explicit-water molecular dynamics simulations, including charge and spin states, water model, simulation duration, global and metal-site RMSDs, temperature, and density. Table S9: Charge and spin multiplicity audit for all 90 benchmark structures, including the source of charge/spin assignments, electron-parity checks, final charge sums, recorded warnings, and calculation status. Table S9a: Investigation of three problematic structures (145192, 850911, and 993277), showing the identified input problems, corrective actions or classifications, and final assessment of each case. Table S9b: Results for five structures with corrected force-field topologies, including atom and bond counts, native and Hybrid RMSDs relative to OrbMol-v2, and structural-integrity check results. Table S9c: Additional consistency checks for the 82 archived calculations, including molecular composition, charge, spin multiplicity, electron counts, parity checks, OrbMol-v2 convergence, and charge sums in the generated force-field topologies. Table S9d: Comparison of the original benchmark RMSD statistics with those obtained after excluding the three flagged structures (145192, 850911, and 993277), including mean, median, and maximum RMSD values. Additional atom-mapping and crystallographic-boundary files are provided for the flagged structures.

## Abbreviations

The following abbreviations are used in this manuscript:

AI	artificial intelligence
ANS	Advanced Numbering System
CM5	Charge Model 5
CSD	Cambridge Structural Database
DFT	density functional theory
ESP	electrostatic potential
GAFF2	General AMBER Force Field 2
IQR	interquartile range
MD	molecular dynamics
ML	machine learning
MLIP	machine learning interatomic potential
PES	potential-energy surface
QM	quantum mechanics
RESP	restrained electrostatic potential
Rg	radius of gyration
RMSD	root-mean-square deviation
SD	standard deviation
SMILES	Simplified Molecular Input Line Entry System
