# Supplementary material for: RapidMetalFF-AI: An AI-Assisted Web Server for Minute-Scale Bonded Force-Field Parameterization and Structural Validation of Metal-Containing Molecules with Validated AMBER and Converted GROMACS/CHARMM Outputs
Source: Molecules. 2026 Oct 17;31(20):3724. doi: 10.3390/molecules31203724 (PMC13707471; doi:10.3390/molecules31203724)
Supplement: Supplementary file 1 [file molecules-31-03724-s001.zip › pdf_versions_of_data/Supplementary_Table_S1_CSD_input_screening_Exact90.pdf]

Sheet1

Molecule	ScreeningDecision	ScreeningReasonCategory	RawAtoms	PrincipalAtoms	ComponentCount
1012591	ACCEPT	ACCEPTED	142	89	4
1033814	ACCEPT	ACCEPTED	144	72	2
1100009	ACCEPT	ACCEPTED	25	20	2
1100100	ACCEPT	ACCEPTED	62	62	1
1100131	ACCEPT	ACCEPTED	38	38	1
1100134	ACCEPT	ACCEPTED	44	15	2
1100181	ACCEPT	ACCEPTED	224	112	2
1100271	ACCEPT	ACCEPTED	104	98	2
1100319	ACCEPT	ACCEPTED	58	29	2
1100364	ACCEPT	ACCEPTED	36	36	1
1100369	ACCEPT	ACCEPTED	29	29	1
1100416	ACCEPT	ACCEPTED	53	37	17
1101146	ACCEPT	ACCEPTED	11	11	1
1101247	ACCEPT	ACCEPTED	23	23	1
1107459	ACCEPT	ACCEPTED	75	75	1
145192	ACCEPT	ACCEPTED	106	81	26
1493407	ACCEPT	ACCEPTED	41	41	1
1506292	ACCEPT	ACCEPTED	123	123	1
1518810	ACCEPT	ACCEPTED	70	70	1
1519012	ACCEPT	ACCEPTED	41	41	1
152002	ACCEPT	ACCEPTED	57	56	2

Sheet1

1530857	ACCEPT	ACCEPTED	98	83	16
154031	ACCEPT	ACCEPTED	72	19	3
154784	WARN	ACCEPTED_WITH_CRYSTALLOGRAPHIC_PERIODIC_IMAGE_WARNING	33	33	1
155447	ACCEPT	ACCEPTED	40	35	2
156313	ACCEPT	ACCEPTED	92	46	2
1563993	ACCEPT	ACCEPTED	137	137	1
1574551	ACCEPT	ACCEPTED	52	45	2
158148	ACCEPT	ACCEPTED	135	102	20
1582689	ACCEPT	ACCEPTED	43	43	1
162486	ACCEPT	ACCEPTED	59	59	1
163152	ACCEPT	ACCEPTED	38	38	1
175478	ACCEPT	ACCEPTED	35	29	3
179921	ACCEPT	ACCEPTED	44	44	1
1822165	ACCEPT	ACCEPTED	92	83	10
182741	ACCEPT	ACCEPTED	52	52	1
183305	ACCEPT	ACCEPTED	54	47	2
2051215	ACCEPT	ACCEPTED	294	134	16
2074417	ACCEPT	ACCEPTED	149	115	3
2075115	ACCEPT	ACCEPTED	39	39	1
2075193_as	ACCEPT	ACCEPTED	115	64	4
2076769	ACCEPT	ACCEPTED	27	27	1
2076771	ACCEPT	ACCEPTED	54	27	2

Sheet1

2083565	ACCEPT	ACCEPTED	70	35	2
2083570	ACCEPT	ACCEPTED	42	42	1
2083695	WARN	ACCEPTED_WITH_CRYSTALLOGRAPHIC_PERIODIC_IMAGE_WARNING	51	51	1
2087213	ACCEPT	ACCEPTED	238	218	21
2087214	ACCEPT	ACCEPTED	129	119	11
2087215	ACCEPT	ACCEPTED	128	116	13
2091598	ACCEPT	ACCEPTED	83	83	1
2096425	ACCEPT	ACCEPTED	66	66	1
2104359	ACCEPT	ACCEPTED	73	59	3
2150100	ACCEPT	ACCEPTED	31	31	1
229783	ACCEPT	ACCEPTED	46	46	1
232528	ACCEPT	ACCEPTED	17	17	1
232536	ACCEPT	ACCEPTED	53	53	1
232591	ACCEPT	ACCEPTED	43	43	1
234033	WARN	ACCEPTED_WITH_CRYSTALLOGRAPHIC_PERIODIC_IMAGE_WARNING	28	28	1
234071	ACCEPT	ACCEPTED	42	42	1
236914	ACCEPT	ACCEPTED	27	27	1
2378332	ACCEPT	ACCEPTED	157	49	5
241977	ACCEPT	ACCEPTED	105	68	38
243849	ACCEPT	ACCEPTED	152	71	4
244158	ACCEPT	ACCEPTED	96	96	1

Sheet1

244175	ACCEPT	ACCEPTED	74	68	3
2454589	ACCEPT	ACCEPTED	69	58	6
2502801	WARN	ACCEPTED_WITH_CRYSTALLOGRAPHIC_PERIODIC_IMAGE_WARNING	87	79	9
251255	ACCEPT	ACCEPTED	101	67	5
2515638	ACCEPT	ACCEPTED	31	24	2
253279	ACCEPT	ACCEPTED	296	250	19
253288	ACCEPT	ACCEPTED	73	73	1
253291	ACCEPT	ACCEPTED	196	196	1
2535798	ACCEPT	ACCEPTED	222	111	2
602743	ACCEPT	ACCEPTED	52	52	1
613741	ACCEPT	ACCEPTED	32	32	1
716039	WARN	ACCEPTED_WITH_CRYSTALLOGRAPHIC_PERIODIC_IMAGE_WARNING	91	91	1
780531	ACCEPT	ACCEPTED	62	62	1
791724	ACCEPT	ACCEPTED	86	49	3
801553	ACCEPT	ACCEPTED	52	52	1
804893	ACCEPT	ACCEPTED	57	57	1
804894	ACCEPT	ACCEPTED	68	68	1
804896	ACCEPT	ACCEPTED	46	34	3
849384	ACCEPT	ACCEPTED	79	79	1
850911	WARN	ACCEPTED_WITH_CRYSTALLOGRAPHIC_PERIODIC_IMAGE_WARNING	37	18	7
850947	ACCEPT	ACCEPTED	79	79	1

Sheet1

851092	ACCEPT	ACCEPTED	455	63	25
876252	ACCEPT	ACCEPTED	67	67	1
902738	ACCEPT	ACCEPTED	30	23	8
942961	WARN	ACCEPTED_WITH_CRYSTALLOGRAPHIC_PERIODIC_IMAGE_WARNING	81	81	1
993277	WARN	ACCEPTED_WITH_CRYSTALLOGRAPHIC_PERIODIC_IMAGE_WARNING	105	103	3

Sheet1

DiscardedAtoms	PeriodicImageWarningCount	DirectOverlapCount	DuplicateAtomIDCount
53	0	0	0
72	0	0	0
5	0	0	0
0	0	0	0
0	0	0	0
29	0	0	0
112	0	0	0
6	0	0	0
29	0	0	0
0	0	0	0
0	0	0	0
16	0	0	0
0	0	0	0
0	0	0	0
0	0	0	0
25	0	0	0
0	0	0	0
0	0	0	0
0	0	0	0
0	0	0	0
1	0	0	0

Sheet1

15	0	0	0
53	0	0	0
0	1	0	0
5	0	0	0
46	0	0	0
0	0	0	0
7	0	0	0
33	0	0	0
0	0	0	0
0	0	0	0
0	0	0	0
6	0	0	0
0	0	0	0
9	0	0	0
0	0	0	0
7	0	0	0
160	0	0	0
34	0	0	0
0	0	0	0
51	0	0	0
0	0	0	0
27	0	0	0

Sheet1

35	0	0	0
0	0	0	0
0	1	0	0
20	0	0	0
10	0	0	0
12	0	0	0
0	0	0	0
0	0	0	0
14	0	0	0
0	0	0	0
0	0	0	0
0	0	0	0
0	0	0	0
0	0	0	0
0	6	0	0
0	0	0	0
0	0	0	0
108	0	0	0
37	0	0	0
81	0	0	0
0	0	0	0
6	0	0	0

Sheet1

11	0	0	0
8	6	0	0
34	0	0	0
7	0	0	0
46	0	0	0
0	0	0	0
0	0	0	0
111	0	0	0
0	0	0	0
0	0	0	0
0	3	0	0
0	0	0	0
37	0	0	0
0	0	0	0
0	0	0	0
0	0	0	0
12	0	0	0
0	0	0	0
19	10	0	0
0	0	0	0
392	0	0	0
0	0	0	0

Sheet1

7	0	0	0
0	1	0	0
2	15	0	0

Sheet1

FinalOutcome
COMPLETED_RMSD_REPORT
COMPLETED_RMSD_REPORT
COMPLETED_RMSD_REPORT
COMPLETED_RMSD_REPORT
COMPLETED_RMSD_REPORT
COMPLETED_RMSD_REPORT
COMPLETED_RMSD_REPORT
COMPLETED_RMSD_REPORT
COMPLETED_RMSD_REPORT
COMPLETED_RMSD_REPORT
SCREENED_IN_NO_COMPLETE_RMSD_IN_SUPPLIED_REPORT
COMPLETED_RMSD_REPORT
COMPLETED_RMSD_REPORT
COMPLETED_RMSD_REPORT
SCREENED_IN_NO_COMPLETE_RMSD_IN_SUPPLIED_REPORT
COMPLETED_RMSD_REPORT
COMPLETED_RMSD_REPORT
COMPLETED_RMSD_REPORT
COMPLETED_RMSD_REPORT
COMPLETED_RMSD_REPORT

Sheet1

COMPLETED_RMSD_REPORT
COMPLETED_RMSD_REPORT
COMPLETED_RMSD_REPORT
COMPLETED_RMSD_REPORT
COMPLETED_RMSD_REPORT
COMPLETED_RMSD_REPORT
COMPLETED_RMSD_REPORT
COMPLETED_RMSD_REPORT
COMPLETED_RMSD_REPORT
COMPLETED_RMSD_REPORT
COMPLETED_RMSD_REPORT
COMPLETED_RMSD_REPORT
COMPLETED_RMSD_REPORT
COMPLETED_RMSD_REPORT
SCREENED_IN_NO_COMPLETE_RMSD_IN_SUPPLIED_REPORT
COMPLETED_RMSD_REPORT
COMPLETED_RMSD_REPORT
COMPLETED_RMSD_REPORT
COMPLETED_RMSD_REPORT
COMPLETED_RMSD_REPORT
COMPLETED_RMSD_REPORT
COMPLETED_RMSD_REPORT

Sheet1

COMPLETED_RMSD_REPORT
COMPLETED_RMSD_REPORT
COMPLETED_RMSD_REPORT
SCREENED_IN_NO_COMPLETE_RMSD_IN_SUPPLIED_REPORT
COMPLETED_RMSD_REPORT
COMPLETED_RMSD_REPORT
COMPLETED_RMSD_REPORT
COMPLETED_RMSD_REPORT
COMPLETED_RMSD_REPORT
COMPLETED_RMSD_REPORT
COMPLETED_RMSD_REPORT
COMPLETED_RMSD_REPORT
COMPLETED_RMSD_REPORT
COMPLETED_RMSD_REPORT
COMPLETED_RMSD_REPORT
COMPLETED_RMSD_REPORT
COMPLETED_RMSD_REPORT
COMPLETED_RMSD_REPORT
COMPLETED_RMSD_REPORT
SCREENED_IN_NO_COMPLETE_RMSD_IN_SUPPLIED_REPORT
COMPLETED_RMSD_REPORT

Sheet1

SCREENED_IN_NO_COMPLETE_RMSD_IN_SUPPLIED_REPORT
COMPLETED_RMSD_REPORT
SCREENED_IN_NO_COMPLETE_RMSD_IN_SUPPLIED_REPORT
SCREENED_IN_NO_COMPLETE_RMSD_IN_SUPPLIED_REPORT
COMPLETED_RMSD_REPORT
COMPLETED_RMSD_REPORT
COMPLETED_RMSD_REPORT
COMPLETED_RMSD_REPORT
COMPLETED_RMSD_REPORT
COMPLETED_RMSD_REPORT
COMPLETED_RMSD_REPORT
COMPLETED_RMSD_REPORT
COMPLETED_RMSD_REPORT
COMPLETED_RMSD_REPORT
COMPLETED_RMSD_REPORT
COMPLETED_RMSD_REPORT
COMPLETED_RMSD_REPORT
COMPLETED_RMSD_REPORT
COMPLETED_RMSD_REPORT
COMPLETED_RMSD_REPORT
COMPLETED_RMSD_REPORT

Sheet1

COMPLETED_RMSD_REPORT
COMPLETED_RMSD_REPORT
COMPLETED_RMSD_REPORT
COMPLETED_RMSD_REPORT
COMPLETED_RMSD_REPORT
COMPLETED_RMSD_REPORT
