# Supplementary material for: RapidMetalFF-AI: An AI-Assisted Web Server for Minute-Scale Bonded Force-Field Parameterization and Structural Validation of Metal-Containing Molecules with Validated AMBER and Converted GROMACS/CHARMM Outputs
Source: Molecules. 2026 Oct 17;31(20):3724. doi: 10.3390/molecules31203724 (PMC13707471; doi:10.3390/molecules31203724)
Supplement: Supplementary file 1 [file molecules-31-03724-s001.zip › pdf_versions_of_data/Supplementary_Table_S2_Experimental_RMSD_Exact82_Strict72_CORRECTED.pdf]

Sheet1

Molecule	SMILES	ANS-SMILES
1012591	[H]C1=C(Cl)C([H])=C(Cl)C2=C1C([H])=[N+]1[C@@]3([H])C([H])([H])C([H])([H])C([H])([H])C([H])([H])[C@]3([H])[N+]3=C([H])C4=C([O][Er+9]13567([O]2)[O]C1=C(C([H])=C(Cl)C([H])=C1Cl)C([H])=[N+]5[C@@]1([H])C([H])([H])C([H])([H])C([H])([H])C([H])([H])[C@]1([H])[N+]6=C([H])C1=C([O]7)C(Cl)=C([H])C(Cl)=C1[H])C(Cl)=C([H])C(Cl)=C4[H]	[H]C1=C(Cl)C([H])=C(Cl)C2=C1C([H])=[N2+]1C3([H])C([H])([H])C([H])([H])C([H])([H])C([H])([H])C3([H])[N1+]3=C([H])C4=C(C(Cl)=C([H])C(Cl)=C4[H])[O6][Er+9]31456([N3+](=C([H])C1=C(C(Cl)=C([H])C(Cl)=C1[H])[O8]4)C1([H])C([H])([H])C([H])([H])C([H])([H])C([H])([H])C1([H])[N4+]5=C([H])C1=C(C(Cl)=C([H])C(Cl)=C1[H])[O7]6)[O5]2
1033814	[H]C1=C([H])C([H])=C([C@@]([H])([N+]2=C([H])C3=C([O][Ir@OH14+7]245([C]2=C([H])C(F)=C([H])C(F)=C2C2=C([H])C([H])=C([H])C([H])=[N+]24)[C]2=C(C(F)=C([H])C(F)=C2[H])C2=C([H])C([H])=C([H])C([H])=[N+]25)C([H])=C([H])C([H])=C3[H])C([H])([H])[H])C([H])=C1[H]	[H]C1=C([H])C([H])=C(C([H])(C([H])([H])[H])[N5+]2=C([H])C3=C(C([H])=C([H])C([H])=C3[H])[O6][Ir+7]234([C1]2=C(C(F)=C([H])C(F)=C2[H])C2=C([H])C([H])=C([H])C([H])=[N4+]23)[C2]2=C([H])C(F)=C([H])C(F)=C2C2=C([H])C([H])=C([H])C([H])=[N3+]24)C([H])=C1[H]
1100009	CC1[O][Ru@+7]234([O-])[O]C(C)[O][Ru@+7]2([O-])([O]1)([O]C(C)[O]3)[O]C(C)[O]4	CC1[O2][Ru+7]234([O5-])[Ru+7]([O10-])([O6]1)([O7]C(C)[O4]2)([O8]C(C)[O1]3)[O9]C(C)[O3]4
1100100	[H]C1=C([H])C([H])=C([N]2C([H])([H])C([H])([H])C([H])([H])[N](C3=C([H])C([H])=C([H])C([H])=C3[H])[Zr@OH15+7]2([Cl])([Cl])([O+]2C([H])([H])C([H])([H])C([H])([H])C2([H])[H])[O+]2C([H])([H])C([H])([H])C([H])([H])C2([H])[H])C([H])=C1[H]	[H]C1=C([H])C([H])=C([N3]2C([H])([H])C([H])([H])C([H])([H])[N4](C3=C([H])C([H])=C([H])C([H])=C3[H])[Zr+7]2([Cl1])([Cl2])([O5+]2C([H])([H])C([H])([H])C([H])([H])C2([H])[H])[O6+]2C([H])([H])C([H])([H])C([H])([H])C2([H])[H])C([H])=C1[H]
1100131	[H]C1=C([H])C([H])=C2C(=[N+](C([H])([H])C([H])([H])C([H])([H])[H])[Ge-]([N]=[N+]=[N-])[N]2C([H])([H])C([H])([H])C([H])([H])[H])C([H])=C1[H]	[H]C1=C([H])C([H])=C2C(=[N2+](C([H])([H])C([H])([H])C([H])([H])[H])[Ge-]([N3]=[N+]=[N-])[N1]2C([H])([H])C([H])([H])C([H])([H])[H])C([H])=C1[H]
1100134	[H]C([H])([H])C([H])([H])[O][Re@OH14+7]([Br])([Br])([Br])([Br])[N]=O	[H]C([H])([H])C([H])([H])[O6][Re+7]([Br1])([Br2])([Br3])([Br4])[N5]=O
1100181	[H]C[Si]([N-]1[Y-7]23([C]([H])([H])C4=C([H])C([H])=C([H])C([H])=C4[H])([C]([H])([H])C4=C([H])C([H])=C([H])C([H])=C4[H])[N-]([Si](C([H])([H])[H])(C([H])([H])[H])C([H])([H])[H])~C(C4=C([H])C([H])=C([H])C([H])=C4[H])~[N]24[Li-3]2([N]3(C([H])([H])C([H])([H])[N+]2(C([H])([H])[H])C([H])([H])[H])~C~1C1=C([H])C([H])=C([H])C([H])=C1[H])[N+](C([H])([H])[H])(C([H])([H])[H])C([H])([H])C4([H])[H])(C([H])([H])[H])C([H])([H])[H]	[H]C1=C([H])C([H])=C(C2~[N1]34[Li-3]5([N2]6(C([H])([H])C([H])([H])[N4+]5(C([H])([H])[H])C([H])([H])[H])[Y-7]3([C13]([H])([H])C3=C([H])C([H])=C([H])C([H])=C3[H])([C14]([H])([H])C3=C([H])C([H])=C([H])C([H])=C3[H])([N8-]~2[Si]([C5][H])([C6]([H])([H])[H])[C7]([H])([H])[H])[N12-]([Si]([C9]([H])([H])[H])([C10]([H])([H])[H])[C11]([H])([H])[H])~C~6C2=C([H])C([H])=C([H])C([H])=C2[H])[N3+](C([H])([H])[H])(C([H])([H])[H])C([H])([H])C4([H])[H])C([H])=C1[H]
1100271	[H]C1=C([H])C([H])=C([P@@+]2(C(C([H])([H])[H])(C([H])([H])[H])C([H])([H])[H])C3=C([H])C([H])=C([H])C([H])=C3[C]([H])([H])[Pd@+5]23[O]C(C([H])([H])[H])[O][Pd@+5]2([O]C(C([H])([H])[H])[O]3)[C]([H])([H])C3=C([H])C([H])=C([H])C([H])=C3[P@@+]2(C2=C(C([H])([H])[H])C([H])=C([H])C([H])=C2[H])C(C([H])([H])[H])(C([H])([H])[H])C([H])([H])[H])C(C([H])([H])[H])=C1[H]	[H]C1=C([H])C([H])=C([P4+]2(C(C([H])([H])[H])(C([H])([H])[H])C([H])([H])[H])C3=C([H])C([H])=C([H])C([H])=C3[C1]([H])([H])[Pd+5]23[O2]C(C([H])([H])[H])[O7][Pd+5]2([C5]([H])([H])C4=C([H])C([H])=C([H])C([H])=C4[P8+]2(C2=C(C([H])([H])[H])C([H])=C([H])C([H])=C2[H])C(C([H])([H])[H])(C([H])([H])[H])C([H])([H])[H])[O6]C(C([H])([H])[H])[O3]3)C(C([H])([H])[H])=C1[H]

Sheet1

1100319	[H]C1~C(C([H])([H])[H])~[O-][Be-2]2([O-]~C~1C([H])([H])[H])[O-]~C(C([H])([H])[H])~C([H])~C(C([H])([H])[H])~[O-]2	[H]C1~C(C([H])([H])[H])~[O1-][Be-2]2([O2-]~C(C([H])([H])[H])~C([H])~C(C([H])([H])[H])~[O4-]2)[O3-]~C~1C([H])([H])[H]
1100364	[H]C1~C(C)~[O-][Sc@OH13-7]23([O-]~C(C)~C([H])~C(C([H])([H])[H])~[O-]2)([O-]~C(C([H])[H])~C([H])~C(C([H])([H])[H])~[O-]3)[O-]~C~1C([H])([H])[H]	[H]C1~C(C([H])[H])~[O1-][Sc-7]23([O2-]~C~1C([H])([H])[H])([O3-]~C(C)~C([H])~C(C([H])([H])[H])~[O6-]2)[O4-]~C(C)~C([H])~C(C([H])([H])[H])~[O5-]3
1100416	[H][N+](=C([O-])C(C([H])([H])[H])(C([H])([H])[H])C([H])([H])[H])[Pt@SP3+5]([Cl])([Cl])[N+]([H])=C([O-])C(C([H])([H])[H])(C([H])([H])[H])C([H])([H])[H]	[H]C([H])([H])C(C([O-])=[N2+]([H])[Pt+5]([Cl1])([Cl3])[N4+]([H])=C([O-])C(C([H])([H])[H])(C([H])([H])[H])C([H])([H])[H])(C([H])([H])[H])C([H])([H])[H]
1101146	CC#[N+][Nb@OH23+8](=[O])([Cl])([Cl])([Cl])[N+]#CC	CC#[N4+][Nb+8]([Cl1])([Cl2])([Cl3])([N5+]#CC)=[O6]
1101247	[H]C1([H])[O+]([Ta@OH14+7]([Cl])([Cl])([Cl])([Cl])[N]=P(Cl)(Cl)Cl)C([H])([H])C([H])([H])C1([H])[H]	[H]C1([H])C([H])([H])C([H])([H])[O6+]([Ta+7]([Cl1])([Cl2])([Cl3])([Cl4])[N5]=P(Cl)(Cl)Cl)C1([H])[H]
145192	[H]C([H])([H])[C@]12=[C@]3(C([H])([H])[H])=[C@]4(C([H])([H])[H])=[C@]5(C([H])([H])[H])=[C@@]=1(C([H])([H])[H])[Yb+14]234516789%10%11[C@]2([H])=[C@@]1([H])=[C@@]6([H])=[C@@]7([H])=[C@@]8([H])=[C@@]9([H])=[C@@]%10([H])=[C@@]=2%11[H]	[H]C([H])([H])[C1]12=[C2]3(C([H])([H])[H])=[C5]4(C([H])([H])[H])=[C3]5(C([H])([H])[H])=[C4]=1(C([H])([H])[H])[Yb+14]234516789%10%11[C6]2([H])=[C11]1([H])=[C10]6([H])=[C9]7([H])=[C7]8([H])=[C8]9([H])=[C13]%10([H])=[C12]=2%11[H]
1493407	[H]C1=C([H])C2=C(C([H])=C1[H])C1=C(O2)C([Se]C([H])([H])C([H])([H])C([H])([H])C([H])([H])[H])=C(C2=C([H])C([H])=C(Cl)C([H])=C2[H])[Se]1	[H]C1=C([H])C2=C(C([H])=C1[H])[C2]1=C(O2)[C3]([Se][C4]([H])([H])C([H])([H])C([H])([H])C([H])([H])[H])=[C1](C2=C([H])C([H])=C(Cl)C([H])=C2[H])[Se]1
1506292	[H]C1=C([H])C(C([H])(C([H])([H])[H])C([H])([H])[H])=C([N]2C(=[N+](C([H])(C([H])([H])[H])C([H])([H])[H])C([H])(C([H])([H])[H])C([H])([H])[H])[N](C3=C(C([H])(C([H])([H])[H])C([H])([H])[H])C([H])=C([H])C([H])=C3C([H])(C([H])([H])[H])C([H])([H])[H])[Mg-2]2([N]([Si](C([H])([H])[H])(C([H])([H])[H])C([H])([H])[H])[Si](C([H])([H])[H])(C([H])([H])[H])C([H])([H])[H])[O+]2C([H])([H])C([H])([H])C([H])([H])C2([H])[H])C(C([H])(C([H])([H])[H])C([H])([H])[H])=C1[H]	[H]C1=C([H])C(C([H])(C([H])([H])[H])C([H])([H])[H])=C([N1]2C(=[N+](C([H])(C([H])([H])[H])C([H])([H])[H])C([H])(C([H])([H])[H])C([H])([H])[H])[N2](C3=C(C([H])(C([H])([H])[H])C([H])([H])[H])C([H])=C([H])C([H])=C3C([H])(C([H])([H])[H])C([H])([H])[H])[Mg-2]2([N3]([Si]([C5]([H])([H])[H])([C6]([H])([H])[H])[C7]([H])([H])[H])[Si]([C8]([H])([H])[H])([C9]([H])([H])[H])[C10]([H])([H])[H])[O4+]2C([H])([H])C([H])([H])C([H])([H])C2([H])[H])C(C([H])(C([H])([H])[H])C([H])([H])[H])=C1[H]
1518810	[H]C1=C([H])C(S(=O)(=O)[N]([Ag@SP2+4]([N+]2=C(C([H])([H])[H])C([H])=C(C([H])([H])[H])C([H])=C2C([H])([H])[H])[N+]2=C(C([H])([H])[H])C([H])=C(C([H])([H])[H])C([H])=C2C([H])([H])[H])S(=O)(=O)C2=C([H])C([H])=C(Br)C([H])=C2[H])=C([H])C([H])=C1Br	[H]C1=C([H])C(S(=O)(=O)[N3]([Ag+4]([N1+]2=C(C([H])([H])[H])C([H])=C(C([H])([H])[H])C([H])=C2C([H])([H])[H])[N2+]2=C(C([H])([H])[H])C([H])=C(C([H])([H])[H])C([H])=C2C([H])([H])[H])S(=O)(=O)C2=C([H])C([H])=C(Br)C([H])=C2[H])=C([H])C([H])=C1Br
1519012	[H]C([H])([H])N(C1~[S-][Sc@OH26-7]([Cl])([Cl])([O+]2C([H])([H])C([H])([H])C([H])([H])C2([H])[H])([O+]2C([H])([H])C([H])([H])C([H])([H])C2([H])[H])[S-]~1)C([H])([H])[H]	[H]C([H])([H])N(C1~[S5-][Sc-7]([Cl1])([Cl2])([O3+]2C([H])([H])C([H])([H])C([H])([H])C2([H])[H])([O4+]2C([H])([H])C([H])([H])C([H])([H])C2([H])[H])[S6-]~1)C([H])([H])[H]

Sheet1

152002	[H]C1=C([H])C([S][Tc+7]2(=[O])([S]C3=C([H])C([H])=C(OC([H])([H])[H])C([H])=C3[H])[S]C([H])([H])C([H])([H])[N+]2(C([H])([H])C([H])([H])[H])C([H])([H])C([H])([H])[H])=C([H])C([H])=C1OC([H])([H])[H]	[H]C1=C([H])C([S3][Tc+7]2(=[O2])([S4]C3=C([H])C([H])=C(OC([H])([H])[H])C([H])=C3[H])[N1+](C([H])([H])C([H])([H])[H])(C([H])([H])C([H])([H])[H])C([H])([H])C([H])([H])[S5]2)=C([H])C([H])=C1OC([H])([H])[H]
1530857	[H]C1~C(C2=C([H])C([H])=C([H])S2)~[O-][Tm+9]234([O-]~C(C5=C([H])C([H])=C([H])S5)~C([H])~C(C(F)(F)F)~[O-]2)([O-]~C(C2=C([H])C([H])=C([H])S2)~C([H])~C(C(F)(F)F)~[O-]3)([O-]~C~1C(F)(F)F)[N+]1=C(C([H])=C([H])C(C2=C([H])C([H])=C([H])C([H])=N2)=N1)C1=[N+]4C([H])=C([H])C([H])=C1[H]	[H]C1~C(C2=C([H])C([H])=C([H])S2)~[O5-][Tm+9]234([N1+]5=C(C([H])=C([H])C([H])=C5[H])C5=[N2+]2N=C(C2=C([H])C([H])=C([H])C([H])=N2)C([H])=C5[H])([O3-]~C(C2=C([H])C([H])=C([H])S2)~C([H])~C(C(F)(F)F)~[O8-]3)([O4-]~C(C2=C([H])C([H])=C([H])S2)~C([H])~C(C(F)(F)F)~[O7-]4)[O6-]~C~1C(F)(F)F
154031	N#C[Se][Pt@OH12+7]12([Se]C#N)([O]C(=O)C(=O)[O]1)[O]C(=O)C(=O)[O]2	N#[C5][Se][Pt+7]12([Se][C6]#N)([O1]C(=O)C(=O)[O2]1)[O3]C(=O)C(=O)[O4]2
154784	[H]C1=C([H])C2=[N+](C([H])=C1[H])[Pb+2]134([S][Pb][S@@+]1C#N)[S][Pb][S@@+]3C(N([H])C([H])([H])[H])=N[N+]4=C2C([H])([H])[H]	[H]C1=C([H])C2=[N1+](C([H])=C1[H])[Pb+2]134([N2+](=C2C([H])([H])[H])N=C(N([H])C([H])([H])[H])[S3+]1[Pb][S5]3)[S4+](C#N)[Pb][S6]4
155447	[H][N+](=C(N(C([H])([H])[H])C([H])([H])[H])C([H])([H])[H])[Pt@SP2+5]([Cl])([Cl])[N+]([H])=C(N(C([H])([H])[H])C([H])([H])[H])C([H])([H])[H]	[H]C([H])([H])C(N(C([H])([H])[H])C([H])([H])[H])=[N3+]([H])[Pt+5]([Cl1])([Cl2])[N4+]([H])=C(N(C([H])([H])[H])C([H])([H])[H])C([H])([H])[H]
156313	[H]C1=C([H])C([H])=C([P+]2(C3=C([H])C([H])=C([H])C([H])=C3[H])C([H])([H])C([H])([H])[S][Tc@TB13+6]234[S]C([H])([H])C([H])([H])[S+]3C([H])([H])C([H])([H])[S]4)C([H])=C1[H]	[H]C1=C([H])C([H])=C([P1+]2(C3=C([H])C([H])=C([H])C([H])=C3[H])C([H])([H])C([H])([H])[S5][Tc+6]234[S2]C([H])([H])C([H])([H])[S4+]3C([H])([H])C([H])([H])[S3]4)C([H])=C1[H]
1563993	[H]C1=C([H])C([H])=C([P](C2=C([H])C([H])=C([H])C([H])=C2[H])[Th+7]234([C]5N(C6=C(C([H])([H])[H])C([H])=C(C([H])([H])[H])C([H])=C6C([H])([H])[H])C([H])=C([H])N5[B-]([H])([H])N5[C]2N(C2=C(C([H])([H])[H])C([H])=C(C([H])([H])[H])C([H])=C2C([H])([H])[H])C([H])=C5[H])[C]2N(C5=C(C([H])([H])[H])C([H])=C(C([H])([H])[H])C([H])=C5C([H])([H])[H])C([H])=C([H])N2[B-]([H])([H])N2[C]3N(C([H])=C2[H])C2=C(C([H])([H])[H])C([H])=C(C([H])([H])[H])C([H])=C2[C]4([H])[H])C([H])=C1[H]	[H]C1=C([H])C([H])=C([P10](C2=C([H])C([H])=C([H])C([H])=C2[H])[Th+7]234([C5]5N(C([H])=C([H])[N2]5[B-]([H])([H])[N1]5C([H])=C([H])N(C6=C(C([H])([H])[H])C([H])=C(C([H])([H])[H])C([H])=C6C([H])([H])[H])[C9]25)C2=C(C([H])([H])[H])C([H])=C(C([H])([H])[H])C([H])=C2[C8]3([H])[H])[C6]2N(C3=C(C([H])([H])[H])C([H])=C(C([H])([H])[H])C([H])=C3C([H])([H])[H])C([H])=C([H])[N3]2[B-]([H])([H])[N4]2C([H])=C([H])N(C3=C(C([H])([H])[H])C([H])=C(C([H])([H])[H])C([H])=C3C([H])([H])[H])[C7]42)C([H])=C1[H]
1574551	[H]C1=C([H])C([H])=C([P+](C2=C([H])C([H])=C([H])C([H])=C2[H])(C2=C([H])C([H])=C([H])C([H])=C2[H])C2=C([H])C([H])=C([H])C([H])=C2[H])C([H])=C1[H]	[H]C1=C([H])C([H])=C([P+](C2=C([H])C([H])=C([H])C([H])=C2[H])(C2=C([H])C([H])=C([H])C([H])=C2[H])C2=C([H])C([H])=C([H])C([H])=C2[H])C([H])=C1[H]

Sheet1

158148	[H]C1=C([H])C([H])=C(N([H])C2=[O+][Eu+10]345678([O-])[O+]=C(N([H])C9=C([H])C([H])=C([H])C([H])=C9[H])C([H])([H])[N@+]39C([H])([H])C([H])([H])[N@@+]4(C2([H])[H])C([H])([H])C([H])([H])[N@@+]5(C([H])([H])C(N([H])C2=C([H])C([H])=C([H])C([H])=C2[H])=[O+]6)C([H])([H])C([H])([H])[N@@+]7(C([H])([H])C(N([H])C2=C([H])C([H])=C([H])C([H])=C2[H])=[O+]8)C([H])([H])C9([H])[H])C([H])=C1[H]	[H]C1=C([H])C([H])=C(N([H])C2=[O5+][Eu+10]345678([O9-])[N1+]9(C([H])([H])C(N([H])C%10=C([H])C([H])=C([H])C([H])=C%10[H])=[O7+]3)C([H])([H])C([H])([H])[N3+]4(C2([H])[H])C([H])([H])C([H])([H])[N2+]5(C([H])([H])C(N([H])C2=C([H])C([H])=C([H])C([H])=C2[H])=[O8+]6)C([H])([H])C([H])([H])[N4+]7(C([H])([H])C(N([H])C2=C([H])C([H])=C([H])C([H])=C2[H])=[O6+]8)C([H])([H])C9([H])[H])C([H])=C1[H]
1582689	[H]C1~C(C([H])([H])[H])~[O-][Ga@OH10-3]23([O-]~C~1C([H])([H])[H])([O-]~C(C([H])([H])[H])~C([H])~C(C([H])([H])[H])~[O-]2)[O-]~C(C([H])([H])[H])~C([H])~C(C([H])([H])[H])~[O-]3	[H]C1~C(C([H])([H])[H])~[O2-][Ga-3]23([O1-]~C(C([H])([H])[H])~C([H])~C(C([H])([H])[H])~[O5-]2)([O3-]~C(C([H])([H])[H])~C([H])~C(C([H])([H])[H])~[O6-]3)[O4-]~C~1C([H])([H])[H]
162486	[H]C([H])([H])C([O+]1[Sn+]2[O+](C(C([H])([H])[H])(C([H])([H])[H])C([H])([H])[H])[In@OH22-3]1([Cl])([Cl])([O+]1C([H])([H])C([H])([H])C([H])([H])C1([H])[H])[O+]2C(C([H])([H])[H])(C([H])([H])[H])C([H])([H])[H])(C([H])([H])[H])C([H])([H])[H]	[H]C([H])([H])C(C([H])([H])[H])(C([H])([H])[H])[O4+]1[Sn+]2[O5+](C(C([H])([H])[H])(C([H])([H])[H])C([H])([H])[H])[In-3]1([Cl1])([Cl2])([O3+]1C([H])([H])C([H])([H])C([H])([H])C1([H])[H])[O6+]2C(C([H])([H])[H])(C([H])([H])[H])C([H])([H])[H]
163152	[H]C([H])([H])C([N]1C([H])([H])C([H])([H])[N@@+]([H])(C(C([H])([H])[H])(C([H])([H])[H])C([H])([H])[H])[Ga-]1([Cl])[Cl])(C([H])([H])[H])C([H])([H])[H]	[H]C([H])([H])C(C([H])([H])[H])(C([H])([H])[H])[N3]1C([H])([H])C([H])([H])[N4+]([H])(C(C([H])([H])[H])(C([H])([H])[H])C([H])([H])[H])[Ga-]1([Cl1])[Cl2]
175478	[H]C1=C2C([H])=C([H])C([H])=C([H])C2=C([H])C(C(C([H])([H])[H])(C([H])([H])[H])C([H])([H])[H])[Te]1	[H]C1=C([H])C2=C([H])[C2](C(C([H])([H])[H])(C([H])([H])[H])C([H])([H])[H])[Te][C1]([H])=C2C([H])=C1[H]
179921	[H]C1=C([H])SC(C(=O)[O][Sn]([C]2=C([H])C([H])=C([H])C([H])=C2[H])([C]2=C([H])C([H])=C([H])C([H])=C2[H])[C]2=C([H])C([H])=C([H])C([H])=C2[H])=N1	[H]C1=C([H])SC(C(=O)[O4][Sn]([C1]2=C([H])C([H])=C([H])C([H])=C2[H])([C2]2=C([H])C([H])=C([H])C([H])=C2[H])[C3]2=C([H])C([H])=C([H])C([H])=C2[H])=N1
182741	[H]C1=C([H])C([H])=C([N-]2[Tl-3][S-]~[P-]~23N(C(C([H])([H])[H])(C([H])([H])[H])C([H])([H])[H])[Si](C([H])([H])[H])(C([H])([H])[H])N3C(C([H])([H])[H])(C([H])([H])[H])C([H])([H])[H])C([H])=C1[H]	[H]C1=C([H])C([H])=C([N5-]2[Tl-3][S6-]~[P-]~23[N3](C(C([H])([H])[H])(C([H])([H])[H])C([H])([H])[H])[Si]([C1]([H])([H])[H])([C2]([H])([H])[H])[N4]3C(C([H])([H])[H])(C([H])([H])[H])C([H])([H])[H])C([H])=C1[H]
183305	[H]C1=C(C2=C([H])C([H])=[N+]([Cu@OH8+7]([N-2])([N-2])([O-])([O-])[N+]3=C([H])C([H])=C(C4=C([H])C([H])=[N+]([Cu+2])C([H])=C4[H])C([H])=C3[H])C([H])=C2[H])C([H])=C([H])[N+]([Cu+2])=C1[H]	[H]C1=C(C2=C([H])C([H])=[N3+]([Cu+7]([N4+]3=C([H])C([H])=C(C4=C([H])C([H])=[N2+]([Cu+2])C([H])=C4[H])C([H])=C3[H])([N5-2])([N6-2])([O7-])[O8-])C([H])=C2[H])C([H])=C([H])[N1+]([Cu+2])=C1[H]

Sheet1

2051215	[H]C1=C([H])C([H])=[C]([Sn]([C]2=C([H])C([H])=C([H])C([H])=C2[H])([C]2=C([H])C([H])=C([H])C([H])=C2[H])[Ba-5]([O+]2C([H])([H])C([H])([H])C([H])([H])C2([H])[H])([O+]2C([H])([H])C([H])([H])C([H])([H])C2([H])[H])([O+]2C([H])([H])C([H])([H])C([H])([H])C2([H])[H])([O+]2C([H])([H])C([H])([H])C([H])([H])C2([H])[H])([O+]2C([H])([H])C([H])([H])C([H])([H])C2([H])[H])[Sn]([C]2=C([H])C([H])=C([H])C([H])=C2[H])([C]2=C([H])C([H])=C([H])C([H])=C2[H])[C]2=C([H])C([H])=C([H])C([H])=C2[H])C([H])=C1[H]	[H]C1=C([H])C([H])=[C6]([Sn]([Ba-5]([Sn]([C9]2=C([H])C([H])=C([H])C([H])=C2[H])([C10]2=C([H])C([H])=C([H])C([H])=C2[H])[C11]2=C([H])C([H])=C([H])C([H])=C2[H])([O1+]2C([H])([H])C([H])([H])C([H])([H])C2([H])[H])([O2+]2C([H])([H])C([H])([H])C([H])([H])C2([H])[H])([O3+]2C([H])([H])C([H])([H])C([H])([H])C2([H])[H])([O4+]2C([H])([H])C([H])([H])C([H])([H])C2([H])[H])[O5+]2C([H])([H])C([H])([H])C([H])([H])C2([H])[H])([C7]2=C([H])C([H])=C([H])C([H])=C2[H])[C8]2=C([H])C([H])=C([H])C([H])=C2[H])C([H])=C1[H]
2074417	[H]C1~C(C(C([H])([H])[H])(C([H])([H])[H])C([H])([H])[H])~[N-](C2=C(C([H])(C([H])([H])[H])C([H])([H])[H])C([H])=C([H])C([H])=C2C([H])(C([H])([H])[H])C([H])([H])[H])[Fe+5]([C]#CC2=C([H])C([H])=C([H])C([H])=C2[H])([N+]2=C([H])C([H])=C([H])C([H])=C2[H])[N-](C2=C(C([H])(C([H])([H])[H])C([H])([H])[H])C([H])=C([H])C([H])=C2C([H])(C([H])([H])[H])C([H])([H])[H])~C~1C(C([H])([H])[H])(C([H])([H])[H])C([H])([H])[H]	[H]C1~C(C(C([H])([H])[H])(C([H])([H])[H])C([H])([H])[H])~[N2-](C2=C(C([H])(C([H])([H])[H])C([H])([H])[H])C([H])=C([H])C([H])=C2C([H])(C([H])([H])[H])C([H])([H])[H])[Fe+5]([C1]#CC2=C([H])C([H])=C([H])C([H])=C2[H])([N4+]2=C([H])C([H])=C([H])C([H])=C2[H])[N3-](C2=C(C([H])(C([H])([H])[H])C([H])([H])[H])C([H])=C([H])C([H])=C2C([H])(C([H])([H])[H])C([H])([H])[H])~C~1C(C([H])([H])[H])(C([H])([H])[H])C([H])([H])[H]
2075115	[H]C([H])([H])C1(C([H])([H])[H])O[Si+]23(C([H])([H])C([H])([H])C([H])([H])Cl)OC([H])([H])C([H])([H])[N+]2(C([H])([H])C([H])([H])O3)C1([H])[H]	[H]C([H])([H])C1(C([H])([H])[H])C([H])([H])[N2+]23C([H])([H])C([H])([H])[O4][Si+]2([C1]([H])([H])C([H])([H])C([H])([H])Cl)([O3]1)[O5]C([H])([H])C3([H])[H]
2075193_as	[H]C1=C([H])C([H])=C2OC([H])([H])C([H])([H])OC([H])([H])C([H])([H])OC([H])([H])C([H])([H])OC3=C(OC([H])([H])C([H])([H])OC([H])([H])C([H])([H])OC([H])([H])C([H])([H])OC2=C1[H])C([H])=C([H])C([H])=C3[H]	[H]C1=C([H])C([H])=C2OC([H])([H])C([H])([H])OC([H])([H])C([H])([H])OC([H])([H])C([H])([H])OC3=C(OC([H])([H])C([H])([H])OC([H])([H])C([H])([H])OC([H])([H])C([H])([H])OC2=C1[H])C([H])=C([H])C([H])=C3[H]
2076769	[H]C1=C([H])C2=C(C([H])=C1[H])[N+]1=C(C([H])=C2[H])C2=C([H])C([H])=C(Cl)N2[B-]1(F)F	[H]C1=C([H])C2=C(C([H])=C1[H])[N4+]1=C(C([H])=C2[H])C2=C([H])C([H])=C(Cl)[N3]2[B-]1([F1])[F2]
2076771	[H]C1=C([H])C2=C(C([H])=C1[H])[N+]1=C(C([H])=C2[H])C2=C([H])C([H])=C(I)N2[B-]1(F)F	[H]C1=C([H])C2=C(C([H])=C1[H])[N4+]1=C(C([H])=C2[H])C2=C([H])C([H])=C(I)[N3]2[B-]1([F1])[F2]
2083565	[H]C1=C([H])C([H])=C(S[P]([W@OH6+7]([C]#[O+])([C]#[O+])([C]#[O+])([C]#[O+])[C]#[O+])[W@OH3+7]([C]#[O+])([C]#[O+])([C]#[O+])([C]#[O+])[C]#[O+])C([H])=C1[H]	[H]C1=C([H])C([H])=C(S[P6]([W+7]([C1]#[O+])([C2]#[O+])([C3]#[O+])([C4]#[O+])[C5]#[O+])[W+7]([C7]#[O+])([C8]#[O+])([C9]#[O+])([C10]#[O+])[C11]#[O+])C([H])=C1[H]
2083570	[H]C1=C([H])C([H])=C([Se@+]2[W+8]3([C]#[O+])([C]#[O+])([C]#[O+])([C]#[O+])[Se@@+](C4=C([H])C([H])=C([H])C([H])=C4[H])[W+8]23([C]#[O+])([C]#[O+])([C]#[O+])[C]#[O+])C([H])=C1[H]	[H]C1=C([H])C([H])=[C1]([Se+]2[W+8]3([C3]#[O+])([C4]#[O+])([C5]#[O+])([C6]#[O+])[Se+]([C2]4=C([H])C([H])=C([H])C([H])=C4[H])[W+8]23([C7]#[O+])([C8]#[O+])([C9]#[O+])[C10]#[O+])C([H])=C1[H]

Sheet1

2087213	[H]C(=NC1=C(C([H])(C([H])([H])[H])C([H])([H])[H])C([H])=C([H])C([H])=C1C([H])(C([H])([H])[H])C([H])([H])[H])C1=C2C(=C([H])C(C(C([H])([H])[H])(C([H])([H])[H])C([H])([H])[H])=C1[H])C1=C([H])C(C(C([H])([H])[H])(C([H])([H])[H])C([H])([H])[H])=C([H])C3=C1[N]2[Ga@-]1([Se][Ga@-]2([Se]1)[N]1C4=C(C([H])=NC5=C(C([H])(C([H])([H])[H])C([H])([H])[H])C([H])=C([H])C([H])=C5C([H])(C([H])([H])[H])C([H])([H])[H])C([H])=C(C(C([H])([H])[H])(C([H])([H])[H])C([H])([H])[H])C([H])=C4C4=C([H])C(C(C([H])([H])[H])(C([H])([H])[H])C([H])([H])[H])=C([H])C(=C41)C([H])=[N+]2C1=C(C([H])(C([H])([H])[H])C([H])([H])[H])C([H])=C([H])C([H])=C1C([H])(C([H])([H])[H])C([H])([H])[H])[N+](C1=C(C([H])(C([H])([H])[H])C([H])([H])[H])C([H])=C([H])C([H])=C1C([H])(C([H])([H])[H])C([H])([H])[H])=C3[H]	[H]C(=NC1=C(C([H])(C([H])([H])[H])C([H])([H])[H])C([H])=C([H])C([H])=C1C([H])(C([H])([H])[H])C([H])([H])[H])C1=C2C(=C([H])C(C(C([H])([H])[H])(C([H])([H])[H])C([H])([H])[H])=C1[H])C1=C([H])C(C(C([H])([H])[H])(C([H])([H])[H])C([H])([H])[H])=C([H])C3=C1[N1]2[Ga-]1([Se][Ga-]2([Se]1)[N3]1C4=C(C([H])=NC5=C(C([H])(C([H])([H])[H])C([H])([H])[H])C([H])=C([H])C([H])=C5C([H])(C([H])([H])[H])C([H])([H])[H])C([H])=C(C(C([H])([H])[H])(C([H])([H])[H])C([H])([H])[H])C([H])=C4C4=C([H])C(C(C([H])([H])[H])(C([H])([H])[H])C([H])([H])[H])=C([H])C(=C41)C([H])=[N4+]2C1=C(C([H])(C([H])([H])[H])C([H])([H])[H])C([H])=C([H])C([H])=C1C([H])(C([H])([H])[H])C([H])([H])[H])[N2+](C1=C(C([H])(C([H])([H])[H])C([H])([H])[H])C([H])=C([H])C([H])=C1C([H])(C([H])([H])[H])C([H])([H])[H])=C3[H]
2087214	[H]C(=NC1=C(C([H])(C([H])([H])[H])C([H])([H])[H])C([H])=C([H])C([H])=C1C([H])(C([H])([H])[H])C([H])([H])[H])C1=C2C(=C([H])C(C(C([H])([H])[H])(C([H])([H])[H])C([H])([H])[H])=C1[H])C1=C([H])C(C(C([H])([H])[H])(C([H])([H])[H])C([H])([H])[H])=C([H])C3=C1[N]2[Ga]([Cr@OH30+7]([C]#[O+])([C]#[O+])([C]#[O+])([C]#[O+])[C]#[O+])[N+](C1=C(C([H])(C([H])([H])[H])C([H])([H])[H])C([H])=C([H])C([H])=C1C([H])(C([H])([H])[H])C([H])([H])[H])=C3[H]	[H]C(=NC1=C(C([H])(C([H])([H])[H])C([H])([H])[H])C([H])=C([H])C([H])=C1C([H])(C([H])([H])[H])C([H])([H])[H])C1=C2C(=C([H])C(C(C([H])([H])[H])(C([H])([H])[H])C([H])([H])[H])=C1[H])C1=C([H])C(C(C([H])([H])[H])(C([H])([H])[H])C([H])([H])[H])=C([H])C3=C1[N7]2[Ga]([Cr+7]([C1]#[O+])([C2]#[O+])([C3]#[O+])([C4]#[O+])[C5]#[O+])[N6+](C1=C(C([H])(C([H])([H])[H])C([H])([H])[H])C([H])=C([H])C([H])=C1C([H])(C([H])([H])[H])C([H])([H])[H])=C3[H]
2087215	[H]C1=C([H])C(C([H])(C([H])([H])[H])C([H])([H])[H])=C([N+]2=C([H])C3=C4C(=C([H])C(C(C([H])([H])[H])(C([H])([H])[H])C([H])([H])[H])=C3[H])C3=C([H])C(C(C([H])([H])[H])(C([H])([H])[H])C([H])([H])[H])=C([H])C5=C3[N]4[Al-2]2([C]([H])([H])[H])([C]([H])([H])[H])[N+](C2=C(C([H])(C([H])([H])[H])C([H])([H])[H])C([H])=C([H])C([H])=C2C([H])(C([H])([H])[H])C([H])([H])[H])=C5[H])C(C([H])(C([H])([H])[H])C([H])([H])[H])=C1[H]	[H]C1=C([H])C(C([H])(C([H])([H])[H])C([H])([H])[H])=C([N4+]2=C([H])C3=C4C(=C([H])C(C(C([H])([H])[H])(C([H])([H])[H])C([H])([H])[H])=C3[H])C3=C([H])C(C(C([H])([H])[H])(C([H])([H])[H])C([H])([H])[H])=C([H])C5=C3[N3]4[Al-2]2([C1]([H])([H])[H])([C2]([H])([H])[H])[N5+](C2=C(C([H])(C([H])([H])[H])C([H])([H])[H])C([H])=C([H])C([H])=C2C([H])(C([H])([H])[H])C([H])([H])[H])=C5[H])C(C([H])(C([H])([H])[H])C([H])([H])[H])=C1[H]
2091598	[H]C([H])([H])[O+]1C([H])([H])C([H])([H])[O+](C([H])([H])[H])[Mg-4]123([O+](C([H])([H])C(F)(F)F)[B-](OC([H])([H])C(F)(F)F)(OC([H])([H])C(F)(F)F)[O+]2C([H])([H])C(F)(F)F)[O+](C([H])([H])C(F)(F)F)[B-](OC([H])([H])C(F)(F)F)(OC([H])([H])C(F)(F)F)[O+]3C([H])([H])C(F)(F)F	[H]C([H])(C(F)(F)F)[O1][B-]1([O2]C([H])([H])C(F)(F)F)[O3+](C([H])([H])C(F)(F)F)[Mg-4]23([O4+]1C([H])([H])C(F)(F)F)([O7+](C([H])([H])C(F)(F)F)[B-]([O5]C([H])([H])C(F)(F)F)([O6]C([H])([H])C(F)(F)F)[O8+]2C([H])([H])C(F)(F)F)[O9+](C([H])([H])[H])C([H])([H])C([H])([H])[O10+]3C([H])([H])[H]
2096425	[H]C1=C([H])C2=C3[C](=C1[H])[Al-]1([C]([H])([H])C([H])([H])[H])[C]4=C([H])C([H])=C([H])C5=C4[N+](=C(C([H])([H])[H])C([H])=C5[H])[Li-2]([N+]3=C(C([H])([H])[H])C([H])=C2[H])[N+]2=C(C([H])([H])[H])C([H])=C([H])C3=C2[C]1=C([H])C([H])=C3[H]	[H]C1=C([H])C([H])=[C2]2C3=C1C([H])=C([H])C(C([H])([H])[H])=[N5+]3[Li-2]1[N6+]3=C(C([H])([H])[H])C([H])=C([H])C4=C3[C1](=C([H])C([H])=C4[H])[Al-]2([C4]([H])([H])C([H])([H])[H])[C3]2=C([H])C([H])=C([H])C3=C2[N7+]1=C(C([H])([H])[H])C([H])=C3[H]

Sheet1

2104359	[H]C1=C([H])C([H])=[N+]2C(=C1[H])C1=C([H])C([H])=C([H])C3=[N+]1[Ru@OH23+7]214([N+]2=C([H])C([H])=C([H])C([H])=C2C2=C([H])C([H])=C([H])C(=[N+]21)C1=C([H])C([H])=C([H])C([H])=[N+]14)[N+]1=C([H])C([H])=C([H])C([H])=C31	[H]C1=C([H])C([H])=[N1+]2C(=C1[H])C1=[N5+]3C(=C([H])C([H])=C1[H])C1=C([H])C([H])=C([H])C([H])=[N3+]1[Ru+7]2314[N2+]2=C([H])C([H])=C([H])C([H])=C2C2=[N6+]1C(=C([H])C([H])=C2[H])C1=C([H])C([H])=C([H])C([H])=[N4+]14
2150100	[H]C1=C([H])C([H])=C2C(=C1[H])C(=O)C([H])([H])C1=C(C([H])=C([H])C([H])=C1[H])N2C(=O)N([H])[H]	[H]C1=C([H])C([H])=C2C(=C1[H])C(=O)C([H])([H])C1=C(C([H])=C([H])C([H])=C1[H])N2C(=O)N([H])[H]
229783	[H]C1=C(C([H])([H])[H])C([H])=C(C([H])([H])[H])C(N2[C]3N(C([H])=C2[H])C2=[N+](C(C([H])([H])[H])(C([H])([H])[H])C([H])([H])O2)[Rh@SP3+5]3([Br])[C]#[O+])=C1C([H])([H])[H]	[H]C1=C(C([H])([H])[H])C([H])=C(C([H])([H])[H])C(N2C([H])=C([H])N3C4=[N4+](C(C([H])([H])[H])(C([H])([H])[H])C([H])([H])O4)[Rh+5]([Br1])([C2]#[O+])[C3]23)=C1C([H])([H])[H]
232528	[H]C([H])([H])[P+](C([H])([H])[H])(C([H])([H])[H])[Au@SP3+5]([I])([I])[I]	[H]C([H])([H])[P4+](C([H])([H])[H])(C([H])([H])[H])[Au+5]([I1])([I2])[I3]
232536	[H]C1=C([H])C([H])=C([P+]([Au+3][Br])(C2=C([H])C([H])=C([H])C([H])=C2[H])C([H])([H])[P+]([Au+3][Br])(C2=C([H])C([H])=C([H])C([H])=C2[H])C2=C([H])C([H])=C([H])C([H])=C2[H])C([H])=C1[H]	[H]C1=C([H])C([H])=C([P2+]([Au+3][Br1])(C2=C([H])C([H])=C([H])C([H])=C2[H])C([H])([H])[P4+]([Au+3][Br3])(C2=C([H])C([H])=C([H])C([H])=C2[H])C2=C([H])C([H])=C([H])C([H])=C2[H])C([H])=C1[H]
232591	[H]C([H])([H])[C]12=[P+]3=[C]4(C([H])([H])[H])=[C]5(C([H])([H])[H])=[C]=1(C([H])([H])[H])~[Fe+]~3~2~4~51678~[P+]2=[C]~1(C([H])([H])[H])=[C]~6(C([H])([H])[H])=[C]~7(C([H])([H])[H])=[C]=2~8C([H])([H])[H]	[H]C([H])([H])[C1]12=[C5]3(C([H])([H])[H])=[C7]4(C([H])([H])[H])=[C3]5(C([H])([H])[H])=[P9+]=1~[Fe+]~2~5~3~41678~[C2]2(C([H])([H])[H])=[C6]~1(C([H])([H])[H])=[C8]~6(C([H])([H])[H])=[C4]~7(C([H])([H])[H])=[P10+]=2~8
234033	[H]C1=C(C2=C([H])C([H])=[N+]([Pb+2]3([N-2])([Cl])([Cl])[Cl+][Pb][Cl+]3)C([H])=C2[H])C([H])=C([H])[N+]([Pb-])=C1[H]	[H]C1=C(C2=C([H])C([H])=[N6+]([Pb+2]3([Cl4])([Cl5])([N7-2])[Cl2+][Pb][Cl3+]3)C([H])=C2[H])C([H])=C([H])[N1+]([Pb-])=C1[H]
234071	[H]C1=C([H])C([H])=[C]2C(=C1[H])C([H])([H])[N@+]1(C(C([H])([H])[H])(C([H])([H])[H])C([H])([H])[H])C([H])([H])C3=C([H])C([H])=C([H])C([H])=[C]3[Bi@+]21[Cl]	[H]C1=C([H])C([H])=[C1]2C(=C1[H])C([H])([H])[N4+]1(C(C([H])([H])[H])(C([H])([H])[H])C([H])([H])[H])C([H])([H])C3=C([H])C([H])=C([H])C([H])=[C2]3[Bi+]21[Cl3]
236914	[H]C([H])([H])C([H])([H])[S@+]1C([H])([H])C([H])([H])[S@@+](C([H])([H])C([H])([H])[H])[Mo@OH22+9]1(=[O])(=[O])([Cl])[Cl]	[H]C([H])([H])C([H])([H])[S5+]1C([H])([H])C([H])([H])[S6+](C([H])([H])C([H])([H])[H])[Mo+9]1([Cl1])([Cl2])(=[O3])=[O4]
2378332	[H]C([H])([H])C([H])([H])C([H])([H])N(C1~[S-][Au@SP3+5]2([S-]~1)[S-]~C(N(C([H])([H])C([H])([H])C([H])([H])[H])C([H])([H])C([H])([H])C([H])([H])[H])~[S-]2)C([H])([H])C([H])([H])C([H])([H])[H]	[H]C([H])([H])C([H])([H])C([H])([H])N(C1~[S1-][Au+5]2([S2-]~1)[S3-]~C(N(C([H])([H])C([H])([H])C([H])([H])[H])C([H])([H])C([H])([H])C([H])([H])[H])~[S4-]2)C([H])([H])C([H])([H])C([H])([H])[H]
243849	[H]C1=C([H])C2=C([H])C3=C(C([H])=C([H])C([H])=C3[H])C(C([H])([H])[N@@+]3(C([H])([H])[H])C([H])([H])C([H])([H])[N@@+](C([H])([H])[H])(C([H])([H])C4=C5C([H])=C([H])C([H])=C([H])C5=C([H])C5=C4C([H])=C([H])C([H])=C5[H])[Pd@SP1+5]3([Cl])[Cl])=C2C([H])=C1[H]	[H]C1=C([H])C2=C([H])C3=C(C([H])=C([H])C([H])=C3[H])C(C([H])([H])[N3+]3(C([H])([H])[H])C([H])([H])C([H])([H])[N4+](C([H])([H])[H])(C([H])([H])C4=C5C([H])=C([H])C([H])=C([H])C5=C([H])C5=C4C([H])=C([H])C([H])=C5[H])[Pd+5]3([Cl1])[Cl2])=C2C([H])=C1[H]

Sheet1

244175	[H]C([H])OC1=C([H])C([H])=C([H])C([H])=C1C([H])([H])[N@@+]1([H])[C@]([H])(C2=C([H])C([H])=C([H])C([H])=C2[H])[C@@]([H])(C2=C([H])C([H])=C([H])C([H])=C2[H])[N@+]([H])(C([H])([H])C2=C([H])C([H])=C([H])C([H])=C2OC([H])([H])[H])[Zn-5]1([Cl])[Cl]	[H]C([H])OC1=C([H])C([H])=C([H])C([H])=C1C([H])([H])[N3+]1([H])C([H])(C2=C([H])C([H])=C([H])C([H])=C2[H])C([H])(C2=C([H])C([H])=C([H])C([H])=C2[H])[N4+]([H])(C([H])([H])C2=C([H])C([H])=C([H])C([H])=C2OC([H])([H])[H])[Zn-5]1([Cl1])[Cl2]
251255	[H]C1=NC(C([H])([H])N2[C]([Hg-3][C]3N(C([H])([H])C4=NC([H])=C([H])C([H])=C4[H])C([H])=C([H])N3C([H])([H])C3=NC([H])=C([H])C([H])=C3[H])N(C([H])([H])C3=C([H])C([H])=C([H])C([H])=N3)C([H])=C2[H])=C([H])C([H])=C1[H]	[H]C1=NC(C([H])([H])N2C([H])=C([H])N(C([H])([H])C3=C([H])C([H])=C([H])C([H])=N3)[C1]2[Hg-3][C2]2N(C([H])([H])C3=NC([H])=C([H])C([H])=C3[H])C([H])=C([H])N2C([H])([H])C2=NC([H])=C([H])C([H])=C2[H])=C([H])C([H])=C1[H]
2515638	[H]C1([H])C([H])([H])C([H])([H])C([H])([N+]([H])([H])C([H])([H])[H])C([H])([H])C1([H])[H]	[H]C1([H])C([H])([H])C([H])([H])C([H])([N+]([H])([H])C([H])([H])[H])C([H])([H])C1([H])[H]
253279	[H]C1=C([H])C([H])=C([P+](C2=C([H])C([H])=C([H])C([H])=C2[H])(C2=C([H])C([H])=C([H])C([H])=C2[H])C2=C([H])C([H])=[C]([Pd@SP2+5]([N+]#CC3=C([H])C([H])=C(C4=C([H])C([H])=C(C#[N+][Pd@SP2+5]([C]5=C([H])C([H])=C([P+](C6=C([H])C([H])=C([H])C([H])=C6[H])(C6=C([H])C([H])=C([H])C([H])=C6[H])C6=C([H])C([H])=C([H])C([H])=C6[H])C([H])=C5[H])([P+](C5=C([H])C([H])=C([H])C([H])=C5[H])(C5=C([H])C([H])=C([H])C([H])=C5[H])C5=C([H])C([H])=C([H])C([H])=C5[H])[P+](C5=C([H])C([H])=C([H])C([H])=C5[H])(C5=C([H])C([H])=C([H])C([H])=C5[H])C5=C([H])C([H])=C([H])C([H])=C5[H])C([H])=C4[H])C([H])=C3[H])([P+](C3=C([H])C([H])=C([H])C([H])=C3[H])(C3=C([H])C([H])=C([H])C([H])=C3[H])C3=C([H])C([H])=C([H])C([H])=C3[H])[P+](C3=C([H])C([H])=C([H])C([H])=C3[H])(C3=C([H])C([H])=C([H])C([H])=C3[H])C3=C([H])C([H])=C([H])C([H])=C3[H])C([H])=C2[H])C([H])=C1[H]	[H]C1=C([H])C([H])=C([P+](C2=C([H])C([H])=C([H])C([H])=C2[H])(C2=C([H])C([H])=C([H])C([H])=C2[H])C2=C([H])C([H])=[C1]([Pd+5]([P2+](C3=C([H])C([H])=C([H])C([H])=C3[H])(C3=C([H])C([H])=C([H])C([H])=C3[H])C3=C([H])C([H])=C([H])C([H])=C3[H])([N3+]#CC3=C([H])C([H])=C(C4=C([H])C([H])=C(C#[N7+][Pd+5]([C5]5=C([H])C([H])=C([P+](C6=C([H])C([H])=C([H])C([H])=C6[H])(C6=C([H])C([H])=C([H])C([H])=C6[H])C6=C([H])C([H])=C([H])C([H])=C6[H])C([H])=C5[H])([P6+](C5=C([H])C([H])=C([H])C([H])=C5[H])(C5=C([H])C([H])=C([H])C([H])=C5[H])C5=C([H])C([H])=C([H])C([H])=C5[H])[P8+](C5=C([H])C([H])=C([H])C([H])=C5[H])(C5=C([H])C([H])=C([H])C([H])=C5[H])C5=C([H])C([H])=C([H])C([H])=C5[H])C([H])=C4[H])C([H])=C3[H])[P4+](C3=C([H])C([H])=C([H])C([H])=C3[H])(C3=C([H])C([H])=C([H])C([H])=C3[H])C3=C([H])C([H])=C([H])C([H])=C3[H])C([H])=C2[H])C([H])=C1[H]
253288	[H]C1=C([H])C(C([H])([H])[H])=C([O][Ti+5]([O]C2=C(C([H])([H])[H])C([H])=C([H])C([H])=C2C([H])([H])[H])([O]C2=C(C([H])([H])[H])C([H])=C([H])C([H])=C2C([H])([H])[H])[O]C2=C(C([H])([H])[H])C([H])=C([H])C([H])=C2C([H])([H])[H])C(C([H])([H])[H])=C1[H]	[H]C1=C([H])C(C([H])([H])[H])=C([O1][Ti+5]([O2]C2=C(C([H])([H])[H])C([H])=C([H])C([H])=C2C([H])([H])[H])([O3]C2=C(C([H])([H])[H])C([H])=C([H])C([H])=C2C([H])([H])[H])[O4]C2=C(C([H])([H])[H])C([H])=C([H])C([H])=C2C([H])([H])[H])C(C([H])([H])[H])=C1[H]

Sheet1

253291	[H]C1=C([H])C([H])=[N+]([Rb-4]234[O+](C([H])([H])C(C([H])([H])[H])(C([H])([H])[H])C([H])([H])[H])[Ti@+6]5([O]C([H])([H])C(C([H])([H])[H])(C([H])([H])[H])C([H])([H])[H])([O+]2C([H])([H])C(C([H])([H])[H])(C([H])([H])[H])C([H])([H])[H])[O+](C([H])([H])C(C([H])([H])[H])(C([H])([H])[H])C([H])([H])[H])[Rb-4]2([N+]6=C([H])C([H])=C([H])C([H])=C6[H])([O+]5C([H])([H])C(C([H])([H])[H])(C([H])([H])[H])C([H])([H])[H])[O+](C([H])([H])C(C([H])([H])[H])(C([H])([H])[H])C([H])([H])[H])[Ti@+6]([O]C([H])([H])C(C([H])([H])[H])(C([H])([H])[H])C([H])([H])[H])([O+]3C([H])([H])C(C([H])([H])[H])(C([H])([H])[H])C([H])([H])[H])([O+]4C([H])([H])C(C([H])([H])[H])(C([H])([H])[H])C([H])([H])[H])[O+]2C([H])([H])C(C([H])([H])[H])(C([H])([H])[H])C([H])([H])[H])C([H])=C1[H]	[H]C1=C([H])C([H])=[N1+]([Rb-4]234[O2+](C([H])([H])C(C([H])([H])[H])(C([H])([H])[H])C([H])([H])[H])[Ti+6]5([O12]C([H])([H])C(C([H])([H])[H])(C([H])([H])[H])C([H])([H])[H])([O4+]2C([H])([H])C(C([H])([H])[H])(C([H])([H])[H])C([H])([H])[H])[O8+](C([H])([H])C(C([H])([H])[H])(C([H])([H])[H])C([H])([H])[H])[Rb-4]2([N6+]6=C([H])C([H])=C([H])C([H])=C6[H])([O7+](C([H])([H])C(C([H])([H])[H])(C([H])([H])[H])C([H])([H])[H])[Ti+6]([O11]C([H])([H])C(C([H])([H])[H])(C([H])([H])[H])C([H])([H])[H])([O3+]3C([H])([H])C(C([H])([H])[H])(C([H])([H])[H])C([H])([H])[H])([O5+]4C([H])([H])C(C([H])([H])[H])(C([H])([H])[H])C([H])([H])[H])[O9+]2C([H])([H])C(C([H])([H])[H])(C([H])([H])[H])C([H])([H])[H])[O10+]5C([H])([H])C(C([H])([H])[H])(C([H])([H])[H])C([H])([H])[H])C([H])=C1[H]
2535798	[H]C1=C([H])C([H])=C(P(C2=C([H])C([H])=C([H])C([H])=C2[H])C([H])([H])C([H])([H])[P+](C2=C([H])C([H])=C([H])C([H])=C2[H])(C2=C([H])C([H])=C([H])C([H])=C2[H])[Mo@OH12+7]2([C]#[O+])([C]#[O+])([C]#[O+])[P+](C3=C([H])C([H])=C([H])C([H])=C3[H])(C3=C([H])C([H])=C([H])C([H])=C3[H])C([H])([H])C([H])([H])[P+]2(C2=C([H])C([H])=C([H])C([H])=C2[H])C2=C([H])C([H])=C([H])C([H])=C2[H])C([H])=C1[H]	[H]C1=C([H])C([H])=C(P(C2=C([H])C([H])=C([H])C([H])=C2[H])C([H])([H])C([H])([H])[P4+](C2=C([H])C([H])=C([H])C([H])=C2[H])(C2=C([H])C([H])=C([H])C([H])=C2[H])[Mo+7]2([C1]#[O+])([C2]#[O+])([C3]#[O+])[P5+](C3=C([H])C([H])=C([H])C([H])=C3[H])(C3=C([H])C([H])=C([H])C([H])=C3[H])C([H])([H])C([H])([H])[P6+]2(C2=C([H])C([H])=C([H])C([H])=C2[H])C2=C([H])C([H])=C([H])C([H])=C2[H])C([H])=C1[H]
602743	[H]C1=C([H])C(C([H])([H])[H])=C([H])C([H])=C1C(=O)[S][Sb@TB18]12([O+]=C(C3=C([H])C([H])=C(C([H])([H])[H])C([H])=C3[H])[S]1)[O+]=C(C1=C([H])C([H])=C(C([H])([H])[H])C([H])=C1[H])[S]2	[H]C1=C([H])C(C([H])([H])[H])=C([H])C([H])=C1C(=O)[S5][Sb]12([O1+]=C(C3=C([H])C([H])=C(C([H])([H])[H])C([H])=C3[H])[S3]1)[O2+]=C(C1=C([H])C([H])=C(C([H])([H])[H])C([H])=C1[H])[S4]2
613741	[H]C1=C([H])C2=[N+](C([H])=C1[H])[In@OH13-3]1([Br])([Br])([Br])[N+]3=C(C([H])=C([H])C([H])=C3[H])C([H])([H])[N+]1([H])C2([H])[H]	[H]C1=C([H])C2=[N4+](C([H])=C1[H])[In-3]1([Br1])([Br2])([Br3])[N5+]3=C(C([H])=C([H])C([H])=C3[H])C([H])([H])[N6+]1([H])C2([H])[H]
716039	[H]C1=C([H])C([H])=C2C(=C1[H])N([H])C1=[N+]2[Ho+8]23([N-2])([N-2])([S]1)[N+]1=C(N([H])C4=C1C([H])=C([H])C([H])=C4[H])[S@@+]2[Ho+8]124([S]C5=[N+]1C1=C([H])C([H])=C([H])C([H])=C1N5[H])([N]1C5=C([H])C([H])=C([H])C([H])=C5[N+]5=C1C1=[N+]([Ho+3]5)C5=C([H])C([H])=C([H])C([H])=C5[N]12)[N+]1=C(N([H])C2=C1C([H])=C([H])C([H])=C2[H])[S@+]34	[H]C1=C([H])C([H])=C2C(=C1[H])N([H])C1=[N3+]2[Ho+8]234([N4+]5=C(N([H])C6=C5C([H])=C([H])C([H])=C6[H])[S8+]2[Ho+8]25([N12-2])([N13-2])([S7+]3C3=[N10+]2C2=C(C([H])=C([H])C([H])=C2[H])N3[H])[N11+]2=C(N([H])C3=C([H])C([H])=C([H])C([H])=C32)[S14]5)([N5]2C3=C([H])C([H])=C([H])C([H])=C3[N2+]3=C2C2=[N1+]([Ho+3]3)C3=C([H])C([H])=C([H])C([H])=C3[N6]24)[S9]1
780531	[H]C1=C([H])C([H])=C(C2=N[N+](=C(C([H])([H])[H])C([H])([H])[H])[Re@OH20+8](=[O])([Cl])([Cl])([P+](C3=C([H])C([H])=C([H])C([H])=C3[H])(C3=C([H])C([H])=C([H])C([H])=C3[H])C3=C([H])C([H])=C([H])C([H])=C3[H])[O]2)C([H])=C1[H]	[H]C1=C([H])C([H])=C(C2=N[N3+](=C(C([H])([H])[H])C([H])([H])[H])[Re+8]([Cl1])([Cl2])(=[O4])([P6+](C3=C([H])C([H])=C([H])C([H])=C3[H])(C3=C([H])C([H])=C([H])C([H])=C3[H])C3=C([H])C([H])=C([H])C([H])=C3[H])[O5]2)C([H])=C1[H]

Sheet1

791724	[H]C1=C([H])C2=C(C([H])=C1[H])[N+]([Mn@OH2+7]([N]=C=S)([N]=C=S)([N]=C=S)([N]=C=S)[N+]1=C(C([H])([H])[H])N([H])C3=C1C([H])=C([H])C([H])=C3[H])=C(C([H])([H])[H])N2[H]	[H]C1=C([H])C2=C(C([H])=C1[H])[N1+]([Mn+7]([N2+]1=C(C([H])([H])[H])N([H])C3=C1C([H])=C([H])C([H])=C3[H])([N3]=C=S)([N4]=C=S)([N5]=C=S)[N6]=C=S)=C(C([H])([H])[H])N2[H]
801553	[H][C]([H])([H])[Al-]1([C]([H])([H])[H])[C]([H])([H])([H])[Tm@OH9+7]23([C]1([H])([H])[H])([C]([H])([H])([H])[Al-]([C]([H])([H])[H])([C]([H])([H])[H])[C]2([H])([H])[H])[C]([H])([H])([H])[Al-]([C]([H])([H])[H])([C]([H])([H])[H])[C]3([H])([H])[H]	[H][C1]([H])([H])[Al-]1([C2]([H])([H])[H])[C3]([H])([H])([H])[Tm+7]23([C4]1([H])([H])[H])([C7]([H])([H])([H])[Al-]([C5]([H])([H])[H])([C6]([H])([H])[H])[C8]2([H])([H])[H])[C11]([H])([H])([H])[Al-]([C9]([H])([H])[H])([C10]([H])([H])[H])[C12]3([H])([H])[H]
804893	[H]C1=C([H])C([H])=[N+]([Ce+9]2([Br])([Br])([Br])([N+]3=C([H])C([H])=C([H])C([H])=C3[H])([N+]3=C([H])C([H])=C([H])C([H])=C3[H])[N+]3=C(C([H])=C([H])C([H])=C3[H])C3=[N+]2C([H])=C([H])C([H])=C3[H])C([H])=C1[H]	[H]C1=C([H])C2=[N4+](C([H])=C1[H])[Ce+9]([Br1])([Br2])([Br3])([N6+]1=C([H])C([H])=C([H])C([H])=C1[H])([N7+]1=C([H])C([H])=C([H])C([H])=C1[H])([N8+]1=C([H])C([H])=C([H])C([H])=C1[H])[N5+]1=C2C([H])=C([H])C([H])=C1[H]
804894	[H]C1([H])[O+]([Ce+8]([Br])([Br])([Br])([O+]2C([H])([H])C([H])([H])C([H])([H])[C@]2([H])C([H])([H])[H])([O+]2C([H])([H])C([H])([H])C([H])([H])[C@@]2([H])C([H])([H])[H])[O+]2C([H])([H])C([H])([H])C([H])([H])[C@@]2([H])C([H])([H])[H])[C@@]([H])(C([H])([H])[H])C([H])([H])C1([H])[H]	[H]C([H])([H])C1([H])C([H])([H])C([H])([H])C([H])([H])[O4+]1[Ce+8]([Br1])([Br2])([Br3])([O5+]1C([H])([H])C([H])([H])C([H])([H])C1([H])C([H])([H])[H])([O6+]1C([H])([H])C([H])([H])C([H])([H])C1([H])C([H])([H])[H])[O7+]1C([H])([H])C([H])([H])C([H])([H])C1([H])C([H])([H])[H]
804896	[H]C([H])([H])C#[N+][La-9]([Br])([Br])([Br])([N+]#CC([H])([H])[H])([N+]#CC([H])([H])[H])([N+]#CC([H])([H])[H])[N+]#CC([H])([H])[H]	[H]C([H])([H])C#[N4+][La-9]([Br1])([Br2])([Br3])([N5+]#CC([H])([H])[H])([N6+]#CC([H])([H])[H])([N7+]#CC([H])([H])[H])[N8+]#CC([H])([H])[H]
849384	[H]C1=C([H])C2=C(C([H])=C1[H])[N]([H])[Os+7]([H])([H])([P+](C([H])(C([H])([H])[H])C([H])([H])[H])(C([H])(C([H])([H])[H])C([H])([H])[H])C([H])(C([H])([H])[H])C([H])([H])[H])([P+](C([H])(C([H])([H])[H])C([H])([H])[H])(C([H])(C([H])([H])[H])C([H])([H])[H])C([H])(C([H])([H])[H])C([H])([H])[H])[N]2[H]	[H]C1=C([H])C2=C(C([H])=C1[H])[N2]([H])[Os+7]([H])([H])([P3+](C([H])(C([H])([H])[H])C([H])([H])[H])(C([H])(C([H])([H])[H])C([H])([H])[H])C([H])(C([H])([H])[H])C([H])([H])[H])([P4+](C([H])(C([H])([H])[H])C([H])([H])[H])(C([H])(C([H])([H])[H])C([H])([H])[H])C([H])(C([H])([H])[H])C([H])([H])[H])[N1]2[H]
850911	O=[P@@]1([O][U+2])[O][U+3][O][P@@](=O)([O][U+2])[O][U@OH23+7]([O-])([O-])([O-])([O-])[O]1	O=P1([O2][U+2])[O4][U+3][O3]P(=O)([O1][U+2])[O6][U+7]([O7-])([O8-])([O9-])([O10-])[O5]1
850947	[H]C1=C([H])C([H])=C([Te][Cu+5]([P+](C([H])([H])C([H])([H])[H])(C([H])([H])C([H])([H])[H])C([H])([H])C([H])([H])[H])([P+](C([H])([H])C([H])([H])[H])(C([H])([H])C([H])([H])[H])C([H])([H])C([H])([H])[H])[P+](C([H])([H])C([H])([H])[H])(C([H])([H])C([H])([H])[H])C([H])([H])C([H])([H])[H])C([H])=C1[H]	[H]C1=C([H])C([H])=[C4]([Te][Cu+5]([P1+](C([H])([H])C([H])([H])[H])(C([H])([H])C([H])([H])[H])C([H])([H])C([H])([H])[H])([P2+](C([H])([H])C([H])([H])[H])(C([H])([H])C([H])([H])[H])C([H])([H])C([H])([H])[H])[P3+](C([H])([H])C([H])([H])[H])(C([H])([H])C([H])([H])[H])C([H])([H])C([H])([H])[H])C([H])=C1[H]
851092	[H]C1([H])[O+]2C([H])([H])C([H])([H])[N+]34C([H])([H])C([H])([H])[O+]5C([H])([H])C([H])([H])[O+]6C([H])([H])C([H])([H])[N+]78C([H])([H])C([H])([H])[O+](C1([H])[H])[K-7]256371[O+](C([H])([H])C([H])([H])[O+]1C([H])([H])C8([H])[H])C([H])([H])C4([H])[H]	[H]C1([H])C([H])([H])[O3+]2C([H])([H])C([H])([H])[O8+]3C([H])([H])C([H])([H])[N2+]45C([H])([H])C([H])([H])[O6+]6C([H])([H])C([H])([H])[O4+]7C([H])([H])C([H])([H])[N1+]18C([H])([H])C([H])([H])[O5+]1C([H])([H])C([H])([H])[O7+](C([H])([H])C4([H])[H])[K-7]8527163

Sheet1

876252	[H]C1=C([H])C([H])=C(C2=C(OC([H])([H])[H])O[Si+2]345(OC(OC([H])([H])[H])=C(C6=C([H])C([H])=C([H])C([H])=C6[H])[N+]3=C([H])C3=C(O4)C([H])=C([H])C([H])=C3[H])OC3=C(C([H])=C([H])C([H])=C3[H])C([H])=[N+]25)C([H])=C1[H]	[H]C1=C([H])C([H])=C(C2=C(OC([H])([H])[H])[O4][Si+2]345([N1+]2=C([H])C2=C(C([H])=C([H])C([H])=C2[H])[O5]3)[N2+](=C([H])C2=C(C([H])=C([H])C([H])=C2[H])[O6]4)C(C2=C([H])C([H])=C([H])C([H])=C2[H])=C(OC([H])([H])[H])[O3]5)C([H])=C1[H]
902738	[H]C([H])(C(=O)[O-])[N+]12C([H])([H])C(=O)[O][Mo@OH16+10]1(=[O])(=[O])(=[O])[O]C(=O)C2([H])[H]	[H]C([H])(C(=O)[O-])[N1+]12C([H])([H])C(=O)[O5][Mo+10]1(=[O2])(=[O3])(=[O4])[O6]C(=O)C2([H])[H]
942961	[H]C1=C([H])C(C2=NC3=C(C4=C5C6=C3C([H])=C([H])C([H])=[N+]6[Sr-6]3([O-])([O-])([O-])([O-])([N+]6=C([H])C([H])=C([H])C7=C6C6=C(C([H])=C([H])C([H])=[N+]63)C3=C7N=C(C6=C([H])C([H])=C(C7[O][Sr][O]7)C([H])=C6[H])N3[H])[N+]5=C([H])C([H])=C4[H])N2[H])=C([H])C([H])=C1C1[O][Sr][O]1	[H]C1=C([H])C(C2=NC3=C(C4=C5C6=C3C([H])=C([H])C([H])=[N5+]6[Sr-6]3([O9-])([O10-])([O11-])([O12-])([N6+]6=C([H])C([H])=C([H])C7=C6C6=C(C([H])=C([H])C([H])=[N8+]63)C3=C7N=C(C6=C([H])C([H])=C(C7[O3][Sr][O4]7)C([H])=C6[H])N3[H])[N7+]5=C([H])C([H])=C4[H])N2[H])=C([H])C([H])=C1C1[O1][Sr][O2]1
993277	[H]C1=C([H])C2=C(C3=C([H])C([H])=C([S+](=O)(~[O-][Tb+2])~[O-][Tb+2])C([H])=C3[H])C3=C([H])C([H])=C([N-]3)C(C3=C([H])C([H])=C([S+](=O)(~[O-][Tb+2])~[O-][Tb+9]([O-])([O-])([O-])([O-])([O-])([O-])[O-])C([H])=C3[H])=C3N=C(C([H])=C3[H])C(C3=C([H])C([H])=C([S+](=O)(~[O-][Tb+2])~[O-][Tb+2])C([H])=C3[H])=C3[N-]C(=C(C4=C([H])C([H])=C([S+](=O)(~[O-][Tb+2])~[O-][Tb+2])C([H])=C4[H])C1=N2)C([H])=C3[H]	[H]C1=C([H])C2=C(C3=C([H])C([H])=C([S+](=O)(~[O3-][Tb+2])~[O4-][Tb+2])C([H])=C3[H])C3=C([H])C([H])=C([N-]3)C(C3=C([H])C([H])=C([S+](=O)(~[O7-][Tb+2])~[O8-][Tb+9]([O9-])([O10-])([O11-])([O12-])([O13-])([O14-])[O15-])C([H])=C3[H])=C3N=C(C([H])=C3[H])C(C3=C([H])C([H])=C([S+](=O)(~[O2-][Tb+2])~[O6-][Tb+2])C([H])=C3[H])=C3[N-]C(=C(C4=C([H])C([H])=C([S+](=O)(~[O1-][Tb+2])~[O5-][Tb+2])C([H])=C4[H])C1=N2)C([H])=C3[H]

Sheet1

ExperimentalToOrbOptimizedRMSD_A	ExperimentalToRapidMetalFFMinimizedRMSD_A	ExperimentalToHybridMinimizedRMSD_A
0.332313	0.422366	0.342602
0.206043	0.362327	0.296419
0.530887	0.616513	0.622216
0.218842	0.223647	0.289666
0.120043	0.458242	0.435864
0.280381	0.446258	0.265846
0.664983	0.955362	0.987024
0.163164	0.68974	0.858608
0.07177	0.404162	0.427996
0.26622	0.549477	0.315969
0.696691	0.738993	1.061406
0.333231	0.435207	0.432148
0.17713	0.576611	0.66502
0.081824	0.444611	0.23155
0.260788	1.026553	0.90773
0.080441	0.328847	0.370086
0.93042	0.922141	0.670394
0.164434	0.703715	0.698344
0.169229	0.823942	1.054958
0.488653	0.870084	0.782107
0.044384	0.589456	0.228046

Sheet1

1.738289	1.258126	1.176232
0.555221	1.011059	1.123012
0.1319	0.184326	0.398386
0.775586	1.267776	1.223849
0.225711	0.267677	0.713101
1.461666	1.085073	1.395856
0.073143	0.425686	0.302628
0.269799	0.226355	0.190301
0.073081	0.164591	0.163366
0.027396	0.041388	0.065943
0.68036	0.863422	0.642081
0.295054	0.635907	0.323992
0.797842	0.615944	0.682516
0.629172	0.968946	0.53185
0.484199	1.749823	0.849716
0.130157	0.150857	0.123254
0.162695	1.441354	1.249985
0.052945	0.337827	0.105005
0.071182	0.33107	0.11362
0.668999	1.21202	0.781368
0.301003	0.700681	0.701293
0.431174	0.826088	0.656077

Sheet1

0.535425	0.748285	0.612836
0.733834	0.787397	0.803707
0.134286	0.725784	0.399245
0.139588	0.285691	0.365385
0.055472	0.103369	0.150548
0.266783	0.253873	0.361818
0.178697	0.954828	0.605133
0.04741	0.085305	0.078187
0.710424	0.85309	1.164103
0.022815	0.139227	0.123508
0.646602	0.536133	0.465468
0.112779	0.152758	0.126103
0.058951	0.101963	0.083228
0.151653	0.84553	0.840457
0.291088	1.163838	0.427407
0.991014	1.130825	1.059431
0.692237	0.918952	1.283732
0.052291	0.15336	0.218788
2.531577	2.580833	1.961484
0.347849	0.329574	0.631809
0.509626	0.395176	0.692462
0.303471	0.453137	0.90208

Sheet1

0.395392	1.036093	0.64435
0.13586	0.164511	0.165554
0.500802	1.480031	1.69009
0.31981	0.642248	0.546849
0.354161	0.538264	0.572024
0.101246	0.330577	0.330057
0.432575	0.373208	0.382109
0.217225	0.275499	0.43494
1.116587	1.060857	1.05102
0.27553	0.827135	0.88293
1.131383	1.138386	1.165948
0.498314	0.639175	0.623135
0.242127	1.358062	1.309495
0.237206	0.453833	0.67061
0.332113	0.654538	0.701756
0.236912	1.834999	3.792733
1.441013	1.871178	1.561315

Sheet1

ExperimentalToRapidMetalFFMinimizedMetalSiteRMSD_A	ExperimentalToHybridMinimizedMetalSiteRMSD_A	Metal(s)
0.265832	0.2631	Er1
0.071323	0.078899	Ir1
0.341213	0.34343	Ru1,Ru2
0.122569	0.145791	Zr1
0.037095	0.054672	Ge
0.143709	0.142787	Re1
1.227161	1.253294	Y1,Li1
0.496627	0.298242	Pd1,Pd2
0.085049	0.096674	Be1
0.073635	0.075217	Sc1
0.091753	0.105123	Pt2
0.196824	0.189832	Nb1
0.029348	0.033639	Ta1
0.347996	0.207225	Yb1
N/A	N/A	n/a
0.123114	0.141781	Mg1
0.181992	0.153232	Ag
0.068766	0.065028	Sc1
0.034765	0.033574	Tc1
0.323661	0.34449	Tm1
0.067931	0.081912	Pt1

Sheet1

1.393813	1.419135	Pb1,Pb1,Pb1
0.083722	0.083823	Pt1
0.038243	0.03161	Tc1
0.33112	0.370471	Th1
N/A	N/A	n/a
0.252905	0.274517	Eu1
0.096476	0.124907	Ga01
0.069867	0.098181	Sn1,In1
0.107124	0.104527	Ga1
N/A	N/A	n/a
0.065232	0.064181	Sn1
0.410478	0.140081	Tl1
0.836558	0.841718	Cu1,Cu1C,Cu1C
0.379925	0.293699	Sn1,Sn2,Ba1
0.240791	0.265766	Fe1
N/A	N/A	n/a
N/A	N/A	n/a
N/A	N/A	n/a
N/A	N/A	n/a
0.350363	0.351853	W1,W2
0.564829	0.563655	W1,W1
0.144302	0.123397	Ga1,Ga2

Sheet1

0.305329	0.281296	Cr1,Ga1
0.151768	0.160163	Al1
0.208683	0.207887	Mg1
0.17939	0.178542	Al1,Li1
0.067267	0.10247	Ru1
N/A	N/A	n/a
0.102326	0.077593	Rh1
0.056236	0.04611	Au1
0.896094	1.276509	Au1,Au1F
0.015673	0.027188	Fe1
0.634801	0.540199	Pb1,Pb1,Pb1
0.057935	0.065589	Bi1
0.076368	0.078664	Mo1
0.132321	0.134946	Au1
0.130817	0.121209	Pd1
0.807906	0.824972	Zn1
0.327138	0.375629	Hg1
N/A	N/A	n/a
1.344141	1.157997	Pd1,Pd1B
0.01936	0.032418	Ti1
0.186929	0.273236	Ti1,Rb1,Rb1B,Ti1B
0.08646	0.118684	Mo1

Sheet1

0.170487	0.171376	Sb
0.10633	0.128041	In1
1.541312	2.077495	Ho1,Ho1A,Ho1A
0.051579	0.051328	Re1
0.400434	0.406	Mn1
0.330577	0.330057	Tm1,Al1,Al2,Al1A
0.12521	0.121572	Ce1
0.172664	0.195774	Ce1
0.090906	0.096431	La1
0.395981	0.527812	Os1
1.036238	1.063549	U1,U1,U1,U1
0.230368	0.235003	Cu1
0.651141	0.570214	K1
N/A	N/A	n/a
0.233325	0.235999	Mo1
2.301505	4.913271	Sr1,Sr1,Sr1
2.764377	2.432722	Tb1,Tb1,Tb1,Tb1,Tb1,Tb1,Tb1,Tb1

Sheet1

MetalSiteAtomCount	MetalSiteSubsetSource	MetalSiteAtomIDs	Status
9	hybrid_typing_protected_atoms	81;82;83;84;85;86;87;88;89	OK
7	hybrid_typing_protected_atoms	46;63;68;69;70;71;72	OK
12	hybrid_typing_protected_atoms	1;2;3;4;5;6;7;8;9;10;11;12	OK
7	hybrid_typing_protected_atoms	1;2;3;4;5;6;7	OK
4	hybrid_typing_protected_atoms	1;2;3;6	OK
7	hybrid_typing_protected_atoms	1;2;3;4;12;14;15	OK
18	hybrid_typing_protected_atoms	1;2;3;4;5;6;7;8;9;10;17;24;25;26;38;39;40;50	OK
10	hybrid_typing_protected_atoms	1;21;91;92;93;94;95;96;97;98	OK
5	hybrid_typing_protected_atoms	1;26;27;28;29	OK
7	hybrid_typing_protected_atoms	30;31;32;33;34;35;36	OK
5	hybrid_typing_protected_atoms	1;2;3;6;7	OK
7	hybrid_typing_protected_atoms	1;2;3;4;5;6;7	OK
7	hybrid_typing_protected_atoms	1;2;3;4;5;10;11	OK
14	hybrid_typing_protected_atoms	1;2;3;4;5;6;27;29;31;33;35;37;39;41	OK
N/A	n/a	n/a	OK_NO_METAL
13	hybrid_typing_protected_atoms	1;2;3;4;6;9;12;89;93;104;108;112;116	OK
4	hybrid_typing_protected_atoms	1;30;31;51	OK
7	hybrid_typing_protected_atoms	1;2;3;4;5;16;17	OK
6	hybrid_typing_protected_atoms	1;2;3;4;5;6	OK
9	hybrid_typing_protected_atoms	1;5;6;9;10;13;14;15;23	OK
9	hybrid_typing_protected_atoms	1;2;3;4;8;11;12;14;18	OK

Sheet1

9	hybrid_typing_protected_atoms	1;12;13;17;18;30;31;32;33	OK
5	hybrid_typing_protected_atoms	1;2;3;4;6	OK
6	hybrid_typing_protected_atoms	1;2;3;4;5;6	OK
17	hybrid_typing_protected_atoms	1;2;3;4;97;120;121;123;124;127;128;130;131;132;133;134;135	OK
N/A	n/a	n/a	OK_NO_METAL
10	hybrid_typing_protected_atoms	1;2;9;16;23;34;52;70;88;102	OK
7	hybrid_typing_protected_atoms	1;2;3;4;5;6;7	OK
8	hybrid_typing_protected_atoms	1;2;3;4;5;6;7;8	OK
5	hybrid_typing_protected_atoms	1;2;3;4;6	OK
N/A	n/a	n/a	OK_NO_METAL
5	hybrid_typing_protected_atoms	1;3;10;16;22	OK
8	hybrid_typing_protected_atoms	1;2;4;5;6;7;8;12	OK
11	hybrid_typing_protected_atoms	1;2;3;16;17;18;19;26;27;40;41	OK
14	hybrid_typing_protected_atoms	1;2;13;24;35;36;47;58;69;70;83;96;109;122	OK
5	hybrid_typing_protected_atoms	1;2;3;4;103	OK
N/A	n/a	n/a	OK_NO_METAL
N/A	n/a	n/a	OK_NO_METAL
N/A	n/a	n/a	OK_NO_METAL
N/A	n/a	n/a	OK_NO_METAL
13	hybrid_typing_protected_atoms	1;2;3;15;16;17;18;19;20;21;22;24;25	OK
14	hybrid_typing_protected_atoms	1;2;7;8;11;12;15;22;23;28;29;32;33;36	OK
8	hybrid_typing_protected_atoms	209;210;211;212;214;215;217;218	OK

Sheet1

9	hybrid_typing_protected_atoms	1;2;3;4;105;106;107;108;109	OK
6	hybrid_typing_protected_atoms	1;106;110;114;115;116	OK
13	hybrid_typing_protected_atoms	1;2;3;5;6;11;41;43;51;52;54;55;82	OK
9	hybrid_typing_protected_atoms	1;2;3;4;5;6;14;32;50	OK
7	hybrid_typing_protected_atoms	1;2;3;5;6;7;22	OK
N/A	n/a	n/a	OK_NO_METAL
5	hybrid_typing_protected_atoms	1;2;3;5;16	OK
5	hybrid_typing_protected_atoms	1;2;3;4;5	OK
6	hybrid_typing_protected_atoms	1;2;3;28;29;30	OK
11	hybrid_typing_protected_atoms	1;2;3;4;13;18;23;24;25;26;27	OK
10	hybrid_typing_protected_atoms	1;2;3;22;23;24;25;26;27;28	OK
5	hybrid_typing_protected_atoms	1;2;3;4;17	OK
7	hybrid_typing_protected_atoms	1;2;3;10;15;17;18	OK
5	hybrid_typing_protected_atoms	1;24;25;48;49	OK
5	hybrid_typing_protected_atoms	1;2;3;5;8	OK
5	hybrid_typing_protected_atoms	1;2;3;6;7	OK
3	hybrid_typing_protected_atoms	1;10;39	OK
N/A	n/a	n/a	OK_NO_METAL
10	hybrid_typing_protected_atoms	1;2;36;81;82;135;136;137;171;205	OK
5	hybrid_typing_protected_atoms	1;2;3;4;5	OK
16	hybrid_typing_protected_atoms	1;2;3;4;5;6;7;8;99;100;101;102;103;120;121;132	OK
7	hybrid_typing_protected_atoms	1;2;3;4;9;10;11	OK

Sheet1

6	hybrid_typing_protected_atoms	1;2;3;4;5;6	OK
7	hybrid_typing_protected_atoms	1;2;3;4;6;19;20	OK
17	hybrid_typing_protected_atoms	1;2;3;4;7;10;11;45;46;47;48;49;52;55;56;57;58	OK
7	hybrid_typing_protected_atoms	1;2;3;4;5;6;7	OK
7	hybrid_typing_protected_atoms	1;4;7;8;28;31;32	OK
16	hybrid_typing_protected_atoms	1;2;3;4;8;12;16;20;24;28;29;33;37;41;45;49	OK
9	hybrid_typing_protected_atoms	1;2;3;4;5;6;7;8;9	OK
8	hybrid_typing_protected_atoms	1;2;3;4;5;36;37;38	OK
9	hybrid_typing_protected_atoms	1;2;3;4;5;6;7;8;9	OK
7	hybrid_typing_protected_atoms	1;2;3;4;5;6;8	OK
14	hybrid_typing_protected_atoms	1;4;5;6;7;8;9;12;13;14;15;16;17;18	OK
6	hybrid_typing_protected_atoms	1;2;3;4;5;69	OK
9	hybrid_typing_protected_atoms	1;2;3;4;5;6;7;8;9	OK
N/A	n/a	n/a	OK_NO_METAL
7	hybrid_typing_protected_atoms	7;8;9;10;11;12;17	OK
15	hybrid_typing_protected_atoms	1;2;3;7;8;39;40;44;45;76;77;78;79;80;81	OK
23	hybrid_typing_protected_atoms	1;17;34;51;57;62;67;83;89;90;91;92;93;94;95;96;97;98;99;100;101;102;103	OK
