# Supplementary material for: RapidMetalFF-AI: An AI-Assisted Web Server for Minute-Scale Bonded Force-Field Parameterization and Structural Validation of Metal-Containing Molecules with Validated AMBER and Converted GROMACS/CHARMM Outputs
Source: Molecules. 2026 Oct 17;31(20):3724. doi: 10.3390/molecules31203724 (PMC13707471; doi:10.3390/molecules31203724)
Supplement: Supplementary file 1 [file molecules-31-03724-s001.zip › pdf_versions_of_data/Supplementary_Table_S3_AI_Supplementary_Table_S1_Exact82_CORRECTED.pdf]

Sheet1

Molecule	SMILES	ANS-SMILES	ExperimentalGeometryRMSD_A
1012591	[H]C1=C(Cl)C([H])=C(Cl)C2=C1C([H])=[N+]1[C@@]3([H])C([H])([H])C([H])([H])C([H])([H])C([H])([H])[C@]3([H])[N+]3=C([H])C4=C([O][Er+9]13567([O]2)[O]C1=C(C([H])=C(Cl)C([H])=C1Cl)C([H])=[N+]5[C@@]1([H])C([H])([H])C([H])([H])C([H])([H])C([H])([H])[C@]1([H])[N+]6=C([H])C1=C([O]7)C(Cl)=C([H])C(Cl)=C1[H])C(Cl)=C([H])C(Cl)=C4[H]	[H]C1=C(Cl)C([H])=C(Cl)C2=C1C([H])=[N2+]1C3([H])C([H])([H])C([H])([H])C([H])([H])C([H])([H])C3([H])[N1+]3=C([H])C4=C(C(Cl)=C([H])C(Cl)=C4[H])[O6][Er+9]31456([N3+](=C([H])C1=C(C(Cl)=C([H])C(Cl)=C1[H])[O8]4)C1([H])C([H])([H])C([H])([H])C([H])([H])C([H])([H])C1([H])[N4+]5=C([H])C1=C(C(Cl)=C([H])C(Cl)=C1[H])[O7]6)[O5]2	0.332313
1033814	[H]C1=C([H])C([H])=C([C@@]([H])([N+]2=C([H])C3=C([O][Ir@OH14+7]245([C]2=C([H])C(F)=C([H])C(F)=C2C2=C([H])C([H])=C([H])C([H])=[N+]24)[C]2=C(C(F)=C([H])C(F)=C2[H])C2=C([H])C([H])=C([H])C([H])=[N+]25)C([H])=C([H])C([H])=C3[H])C([H])([H])[H])C([H])=C1[H]	[H]C1=C([H])C([H])=C(C([H])(C([H])([H])[H])[N5+]2=C([H])C3=C(C([H])=C([H])C([H])=C3[H])[O6][Ir+7]234([C1]2=C(C(F)=C([H])C(F)=C2[H])C2=C([H])C([H])=C([H])C([H])=[N4+]23)[C2]2=C([H])C(F)=C([H])C(F)=C2C2=C([H])C([H])=C([H])C([H])=[N3+]24)C([H])=C1[H]	0.206043
1100009	CC1[O][Ru@+7]234([O-])[O]C(C)[O][Ru@+7]2([O-])([O]1)([O]C(C)[O]3)[O]C(C)[O]4	CC1[O2][Ru+7]234([O5-])[Ru+7]([O10-])([O6]1)([O7]C(C)[O4]2)([O8]C(C)[O1]3)[O9]C(C)[O3]4	0.530887
1100100	[H]C1=C([H])C([H])=C([N]2C([H])([H])C([H])([H])C([H])([H])[N](C3=C([H])C([H])=C([H])C([H])=C3[H])[Zr@OH15+7]2([Cl])([Cl])([O+]2C([H])([H])C([H])([H])C([H])([H])C2([H])[H])[O+]2C([H])([H])C([H])([H])C([H])([H])C2([H])[H])C([H])=C1[H]	[H]C1=C([H])C([H])=C([N3]2C([H])([H])C([H])([H])C([H])([H])[N4](C3=C([H])C([H])=C([H])C([H])=C3[H])[Zr+7]2([Cl1])([Cl2])([O5+]2C([H])([H])C([H])([H])C([H])([H])C2([H])[H])[O6+]2C([H])([H])C([H])([H])C([H])([H])C2([H])[H])C([H])=C1[H]	0.218842
1100131	[H]C1=C([H])C([H])=C2C(=[N+](C([H])([H])C([H])([H])C([H])([H])[H])[Ge-]([N]=[N+]=[N-])[N]2C([H])([H])C([H])([H])C([H])([H])[H])C([H])=C1[H]	[H]C1=C([H])C([H])=C2C(=[N2+](C([H])([H])C([H])([H])C([H])([H])[H])[Ge-]([N3]=[N+]=[N-])[N1]2C([H])([H])C([H])([H])C([H])([H])[H])C([H])=C1[H]	0.120043
1100134	[H]C([H])([H])C([H])([H])[O][Re@OH14+7]([Br])([Br])([Br])([Br])[N]=O	[H]C([H])([H])C([H])([H])[O6][Re+7]([Br1])([Br2])([Br3])([Br4])[N5]=O	0.280381
1100181	[H]C[Si]([N-]1[Y-7]23([C]([H])([H])C4=C([H])C([H])=C([H])C([H])=C4[H])([C]([H])([H])C4=C([H])C([H])=C([H])C([H])=C4[H])[N-]([Si](C([H])([H])[H])(C([H])([H])[H])C([H])([H])[H])~C(C4=C([H])C([H])=C([H])C([H])=C4[H])~[N]24[Li-3]2([N]3(C([H])([H])C([H])([H])[N+]2(C([H])([H])[H])C([H])([H])[H])~C~1C1=C([H])C([H])=C([H])C([H])=C1[H])[N+](C([H])([H])[H])(C([H])([H])[H])C([H])([H])C4([H])[H])(C([H])([H])[H])C([H])([H])[H]	[H]C1=C([H])C([H])=C(C2~[N1]34[Li-3]5([N2]6(C([H])([H])C([H])([H])[N4+]5(C([H])([H])[H])C([H])([H])[H])[Y-7]3([C13]([H])([H])C3=C([H])C([H])=C([H])C([H])=C3[H])([C14]([H])([H])C3=C([H])C([H])=C([H])C([H])=C3[H])([N8-]~2[Si]([C5][H])([C6]([H])([H])[H])[C7]([H])([H])[H])[N12-]([Si]([C9]([H])([H])[H])([C10]([H])([H])[H])[C11]([H])([H])[H])~C~6C2=C([H])C([H])=C([H])C([H])=C2[H])[N3+](C([H])([H])[H])(C([H])([H])[H])C([H])([H])C4([H])[H])C([H])=C1[H]	0.664983
1100271	[H]C1=C([H])C([H])=C([P@@+]2(C(C([H])([H])[H])(C([H])([H])[H])C([H])([H])[H])C3=C([H])C([H])=C([H])C([H])=C3[C]([H])([H])[Pd@+5]23[O]C(C([H])([H])[H])[O][Pd@+5]2([O]C(C([H])([H])[H])[O]3)[C]([H])([H])C3=C([H])C([H])=C([H])C([H])=C3[P@@+]2(C2=C(C([H])([H])[H])C([H])=C([H])C([H])=C2[H])C(C([H])([H])[H])(C([H])([H])[H])C([H])([H])[H])C(C([H])([H])[H])=C1[H]	[H]C1=C([H])C([H])=C([P4+]2(C(C([H])([H])[H])(C([H])([H])[H])C([H])([H])[H])C3=C([H])C([H])=C([H])C([H])=C3[C1]([H])([H])[Pd+5]23[O2]C(C([H])([H])[H])[O7][Pd+5]2([C5]([H])([H])C4=C([H])C([H])=C([H])C([H])=C4[P8+]2(C2=C(C([H])([H])[H])C([H])=C([H])C([H])=C2[H])C(C([H])([H])[H])(C([H])([H])[H])C([H])([H])[H])[O6]C(C([H])([H])[H])[O3]3)C(C([H])([H])[H])=C1[H]	0.163164

Sheet1

1100319	[H]C1~C(C([H])([H])[H])~[O-][Be-2]2([O-]~C~1C([H])([H])[H])[O-]~C(C([H])([H])[H])~C([H])~C(C([H])([H])[H])~[O-]2	[H]C1~C(C([H])([H])[H])~[O1-][Be-2]2([O2-]~C(C([H])([H])[H])~C([H])~C(C([H])([H])[H])~[O4-]2)[O3-]~C~1C([H])([H])[H]	0.07177
1100364	[H]C1~C(C)~[O-][Sc@OH13-7]23([O-]~C(C)~C([H])~C(C([H])([H])[H])~[O-]2)([O-]~C(C([H])[H])~C([H])~C(C([H])([H])[H])~[O-]3)[O-]~C~1C([H])([H])[H]	[H]C1~C(C([H])[H])~[O1-][Sc-7]23([O2-]~C~1C([H])([H])[H])([O3-]~C(C)~C([H])~C(C([H])([H])[H])~[O6-]2)[O4-]~C(C)~C([H])~C(C([H])([H])[H])~[O5-]3	0.26622
1100416	[H][N+](=C([O-])C(C([H])([H])[H])(C([H])([H])[H])C([H])([H])[H])[Pt@SP3+5]([Cl])([Cl])[N+]([H])=C([O-])C(C([H])([H])[H])(C([H])([H])[H])C([H])([H])[H]	[H]C([H])([H])C(C([O-])=[N2+]([H])[Pt+5]([Cl1])([Cl3])[N4+]([H])=C([O-])C(C([H])([H])[H])(C([H])([H])[H])C([H])([H])[H])(C([H])([H])[H])C([H])([H])[H]	0.696691
1101146	CC#[N+][Nb@OH23+8](=[O])([Cl])([Cl])([Cl])[N+]#CC	CC#[N4+][Nb+8]([Cl1])([Cl2])([Cl3])([N5+]#CC)=[O6]	0.333231
1101247	[H]C1([H])[O+]([Ta@OH14+7]([Cl])([Cl])([Cl])([Cl])[N]=P(Cl)(Cl)Cl)C([H])([H])C([H])([H])C1([H])[H]	[H]C1([H])C([H])([H])C([H])([H])[O6+]([Ta+7]([Cl1])([Cl2])([Cl3])([Cl4])[N5]=P(Cl)(Cl)Cl)C1([H])[H]	0.17713
145192	[H]C([H])([H])[C@]12=[C@]3(C([H])([H])[H])=[C@]4(C([H])([H])[H])=[C@]5(C([H])([H])[H])=[C@@]=1(C([H])([H])[H])[Yb+14]234516789%10%11[C@]2([H])=[C@@]1([H])=[C@@]6([H])=[C@@]7([H])=[C@@]8([H])=[C@@]9([H])=[C@@]%10([H])=[C@@]=2%11[H]	[H]C([H])([H])[C1]12=[C2]3(C([H])([H])[H])=[C5]4(C([H])([H])[H])=[C3]5(C([H])([H])[H])=[C4]=1(C([H])([H])[H])[Yb+14]235416789%10%11[C6]2([H])=[C11]1([H])=[C10]6([H])=[C9]7([H])=[C7]8([H])=[C8]9([H])=[C13]%10([H])=[C12]=2%11[H]	0.081824
1493407	[H]C1=C([H])C2=C(C([H])=C1[H])C1=C(O2)C([Se]C([H])([H])C([H])([H])C([H])([H])C([H])([H])[H])=C(C2=C([H])C([H])=C(Cl)C([H])=C2[H])[Se]1	[H]C1=C([H])C2=C(C([H])=C1[H])[C2]1=C(O2)[C3]([Se][C4]([H])([H])C([H])([H])C([H])([H])C([H])([H])[H])=[C1](C2=C([H])C([H])=C(Cl)C([H])=C2[H])[Se]1	0.260788
1506292	[H]C1=C([H])C(C([H])(C([H])([H])[H])C([H])([H])[H])=C([N]2C(=[N+](C([H])(C([H])([H])[H])C([H])([H])[H])C([H])(C([H])([H])[H])C([H])([H])[H])[N](C3=C(C([H])(C([H])([H])[H])C([H])([H])[H])C([H])=C([H])C([H])=C3C([H])(C([H])([H])[H])C([H])([H])[H])[Mg-2]2([N]([Si](C([H])([H])[H])(C([H])([H])[H])C([H])([H])[H])[Si](C([H])([H])[H])(C([H])([H])[H])C([H])([H])[H])[O+]2C([H])([H])C([H])([H])C([H])([H])C2([H])[H])C(C([H])(C([H])([H])[H])C([H])([H])[H])=C1[H]	[H]C1=C([H])C(C([H])(C([H])([H])[H])C([H])([H])[H])=C([N1]2C(=[N+](C([H])(C([H])([H])[H])C([H])([H])[H])C([H])(C([H])([H])[H])C([H])([H])[H])[N2](C3=C(C([H])(C([H])([H])[H])C([H])([H])[H])C([H])=C([H])C([H])=C3C([H])(C([H])([H])[H])C([H])([H])[H])[Mg-2]2([N3]([Si]([C5]([H])([H])[H])([C6]([H])([H])[H])[C7]([H])([H])[H])[Si]([C8]([H])([H])[H])([C9]([H])([H])[H])[C10]([H])([H])[H])[O4+]2C([H])([H])C([H])([H])C([H])([H])C2([H])[H])C(C([H])(C([H])([H])[H])C([H])([H])[H])=C1[H]	0.080441
1518810	[H]C1=C([H])C(S(=O)(=O)[N]([Ag@SP2+4]([N+]2=C(C([H])([H])[H])C([H])=C(C([H])([H])[H])C([H])=C2C([H])([H])[H])[N+]2=C(C([H])([H])[H])C([H])=C(C([H])([H])[H])C([H])=C2C([H])([H])[H])S(=O)(=O)C2=C([H])C([H])=C(Br)C([H])=C2[H])=C([H])C([H])=C1Br	[H]C1=C([H])C(S(=O)(=O)[N3]([Ag+4]([N1+]2=C(C([H])([H])[H])C([H])=C(C([H])([H])[H])C([H])=C2C([H])([H])[H])[N2+]2=C(C([H])([H])[H])C([H])=C(C([H])([H])[H])C([H])=C2C([H])([H])[H])S(=O)(=O)C2=C([H])C([H])=C(Br)C([H])=C2[H])=C([H])C([H])=C1Br	0.93042
1519012	[H]C([H])([H])N(C1~[S-][Sc@OH26-7]([Cl])([Cl])([O+]2C([H])([H])C([H])([H])C([H])([H])C2([H])[H])([O+]2C([H])([H])C([H])([H])C([H])([H])C2([H])[H])[S-]~1)C([H])([H])[H]	[H]C([H])([H])N(C1~[S5-][Sc-7]([Cl1])([Cl2])([O3+]2C([H])([H])C([H])([H])C([H])([H])C2([H])[H])([O4+]2C([H])([H])C([H])([H])C([H])([H])C2([H])[H])[S6-]~1)C([H])([H])[H]	0.164434

Sheet1

152002	[H]C1=C([H])C([S][Tc+7]2(=[O])([S]C3=C([H])C([H])=C(OC([H])([H])[H])C([H])=C3[H])[S]C([H])([H])C([H])([H])[N+]2(C([H])([H])C([H])([H])[H])C([H])([H])C([H])([H])[H])=C([H])C([H])=C1OC([H])([H])[H]	[H]C1=C([H])C([S3][Tc+7]2(=[O2])([S4]C3=C([H])C([H])=C(OC([H])([H])[H])C([H])=C3[H])[N1+](C([H])([H])C([H])([H])[H])(C([H])([H])C([H])([H])[H])C([H])([H])C([H])([H])[S5]2)=C([H])C([H])=C1OC([H])([H])[H]	0.169229
1530857	[H]C1~C(C2=C([H])C([H])=C([H])S2)~[O-][Tm+9]234([O-]~C(C5=C([H])C([H])=C([H])S5)~C([H])~C(C(F)(F)F)~[O-]2)([O-]~C(C2=C([H])C([H])=C([H])S2)~C([H])~C(C(F)(F)F)~[O-]3)([O-]~C~1C(F)(F)F)[N+]1=C(C([H])=C([H])C(C2=C([H])C([H])=C([H])C([H])=N2)=N1)C1=[N+]4C([H])=C([H])C([H])=C1[H]	[H]C1~C(C2=C([H])C([H])=C([H])S2)~[O5-][Tm+9]234([N1+]5=C(C([H])=C([H])C([H])=C5[H])C5=[N2+]2N=C(C2=C([H])C([H])=C([H])C([H])=N2)C([H])=C5[H])([O3-]~C(C2=C([H])C([H])=C([H])S2)~C([H])~C(C(F)(F)F)~[O8-]3)([O4-]~C(C2=C([H])C([H])=C([H])S2)~C([H])~C(C(F)(F)F)~[O7-]4)[O6-]~C~1C(F)(F)F	0.488653
154031	N#C[Se][Pt@OH12+7]12([Se]C#N)([O]C(=O)C(=O)[O]1)[O]C(=O)C(=O)[O]2	N#[C5][Se][Pt+7]12([Se][C6]#N)([O1]C(=O)C(=O)[O2]1)[O3]C(=O)C(=O)[O4]2	0.044384
154784	[H]C1=C([H])C2=[N+](C([H])=C1[H])[Pb+2]134([S][Pb][S@@+]1C#N)[S][Pb][S@@+]3C(N([H])C([H])([H])[H])=N[N+]4=C2C([H])([H])[H]	[H]C1=C([H])C2=[N1+](C([H])=C1[H])[Pb+2]134([N2+](=C2C([H])([H])[H])N=C(N([H])C([H])([H])[H])[S3+]1[Pb][S5]3)[S4+](C#N)[Pb][S6]4	1.738289
155447	[H][N+](=C(N(C([H])([H])[H])C([H])([H])[H])C([H])([H])[H])[Pt@SP2+5]([Cl])([Cl])[N+]([H])=C(N(C([H])([H])[H])C([H])([H])[H])C([H])([H])[H]	[H]C([H])([H])C(N(C([H])([H])[H])C([H])([H])[H])=[N3+]([H])[Pt+5]([Cl1])([Cl2])[N4+]([H])=C(N(C([H])([H])[H])C([H])([H])[H])C([H])([H])[H]	0.555221
156313	[H]C1=C([H])C([H])=C([P+]2(C3=C([H])C([H])=C([H])C([H])=C3[H])C([H])([H])C([H])([H])[S][Tc@TB13+6]234[S]C([H])([H])C([H])([H])[S+]3C([H])([H])C([H])([H])[S]4)C([H])=C1[H]	[H]C1=C([H])C([H])=C([P1+]2(C3=C([H])C([H])=C([H])C([H])=C3[H])C([H])([H])C([H])([H])[S5][Tc+6]234[S2]C([H])([H])C([H])([H])[S4+]3C([H])([H])C([H])([H])[S3]4)C([H])=C1[H]	0.1319
1563993	[H]C1=C([H])C([H])=C([P](C2=C([H])C([H])=C([H])C([H])=C2[H])[Th+7]234([C]5N(C6=C(C([H])([H])[H])C([H])=C(C([H])([H])[H])C([H])=C6C([H])([H])[H])C([H])=C([H])N5[B-]([H])([H])N5[C]2N(C2=C(C([H])([H])[H])C([H])=C(C([H])([H])[H])C([H])=C2C([H])([H])[H])C([H])=C5[H])[C]2N(C5=C(C([H])([H])[H])C([H])=C(C([H])([H])[H])C([H])=C5C([H])([H])[H])C([H])=C([H])N2[B-]([H])([H])N2[C]3N(C([H])=C2[H])C2=C(C([H])([H])[H])C([H])=C(C([H])([H])[H])C([H])=C2[C]4([H])[H])C([H])=C1[H]	[H]C1=C([H])C([H])=C([P10](C2=C([H])C([H])=C([H])C([H])=C2[H])[Th+7]234([C5]5N(C([H])=C([H])[N2]5[B-]([H])([H])[N1]5C([H])=C([H])N(C6=C(C([H])([H])[H])C([H])=C(C([H])([H])[H])C([H])=C6C([H])([H])[H])[C9]25)C2=C(C([H])([H])[H])C([H])=C(C([H])([H])[H])C([H])=C2[C8]3([H])[H])[C6]2N(C3=C(C([H])([H])[H])C([H])=C(C([H])([H])[H])C([H])=C3C([H])([H])[H])C([H])=C([H])[N3]2[B-]([H])([H])[N4]2C([H])=C([H])N(C3=C(C([H])([H])[H])C([H])=C(C([H])([H])[H])C([H])=C3C([H])([H])[H])[C7]42)C([H])=C1[H]	0.775586
1574551	[H]C1=C([H])C([H])=C([P+](C2=C([H])C([H])=C([H])C([H])=C2[H])(C2=C([H])C([H])=C([H])C([H])=C2[H])C2=C([H])C([H])=C([H])C([H])=C2[H])C([H])=C1[H]	[H]C1=C([H])C([H])=C([P+](C2=C([H])C([H])=C([H])C([H])=C2[H])(C2=C([H])C([H])=C([H])C([H])=C2[H])C2=C([H])C([H])=C([H])C([H])=C2[H])C([H])=C1[H]	0.225711

Sheet1

158148	[H]C1=C([H])C([H])=C(N([H])C2=[O+][Eu+10]345678([O-])[O+]=C(N([H])C9=C([H])C([H])=C([H])C([H])=C9[H])C([H])([H])[N@+]39C([H])([H])C([H])([H])[N@@+]4(C2([H])[H])C([H])([H])C([H])([H])[N@@+]5(C([H])([H])C(N([H])C2=C([H])C([H])=C([H])C([H])=C2[H])=[O+]6)C([H])([H])C([H])([H])[N@@+]7(C([H])([H])C(N([H])C2=C([H])C([H])=C([H])C([H])=C2[H])=[O+]8)C([H])([H])C9([H])[H])C([H])=C1[H]	[H]C1=C([H])C([H])=C(N([H])C2=[O5+][Eu+10]345678([O9-])[N1+]9(C([H])([H])C(N([H])C%10=C([H])C([H])=C([H])C([H])=C%10[H])=[O7+]3)C([H])([H])C([H])([H])[N3+]4(C2([H])[H])C([H])([H])C([H])([H])[N2+]5(C([H])([H])C(N([H])C2=C([H])C([H])=C([H])C([H])=C2[H])=[O8+]6)C([H])([H])C([H])([H])[N4+]7(C([H])([H])C(N([H])C2=C([H])C([H])=C([H])C([H])=C2[H])=[O6+]8)C([H])([H])C9([H])[H])C([H])=C1[H]	1.461666
1582689	[H]C1~C(C([H])([H])[H])~[O-][Ga@OH10-3]23([O-]~C~1C([H])([H])[H])([O-]~C(C([H])([H])[H])~C([H])~C(C([H])([H])[H])~[O-]2)[O-]~C(C([H])([H])[H])~C([H])~C(C([H])([H])[H])~[O-]3	[H]C1~C(C([H])([H])[H])~[O2-][Ga-3]23([O1-]~C(C([H])([H])[H])~C([H])~C(C([H])([H])[H])~[O5-]2)([O3-]~C(C([H])([H])[H])~C([H])~C(C([H])([H])[H])~[O6-]3)[O4-]~C~1C([H])([H])[H]	0.073143
162486	[H]C([H])([H])C([O+]1[Sn+]2[O+](C(C([H])([H])[H])(C([H])([H])[H])C([H])([H])[H])[In@OH22-3]1([Cl])([Cl])([O+]1C([H])([H])C([H])([H])C([H])([H])C1([H])[H])[O+]2C(C([H])([H])[H])(C([H])([H])[H])C([H])([H])[H])(C([H])([H])[H])C([H])([H])[H]	[H]C([H])([H])C(C([H])([H])[H])(C([H])([H])[H])[O4+]1[Sn+]2[O5+](C(C([H])([H])[H])(C([H])([H])[H])C([H])([H])[H])[In-3]1([Cl1])([Cl2])([O3+]1C([H])([H])C([H])([H])C([H])([H])C1([H])[H])[O6+]2C(C([H])([H])[H])(C([H])([H])[H])C([H])([H])[H]	0.269799
163152	[H]C([H])([H])C([N]1C([H])([H])C([H])([H])[N@@+]([H])(C(C([H])([H])[H])(C([H])([H])[H])C([H])([H])[H])[Ga-]1([Cl])[Cl])(C([H])([H])[H])C([H])([H])[H]	[H]C([H])([H])C(C([H])([H])[H])(C([H])([H])[H])[N3]1C([H])([H])C([H])([H])[N4+]([H])(C(C([H])([H])[H])(C([H])([H])[H])C([H])([H])[H])[Ga-]1([Cl1])[Cl2]	0.073081
175478	[H]C1=C2C([H])=C([H])C([H])=C([H])C2=C([H])C(C(C([H])([H])[H])(C([H])([H])[H])C([H])([H])[H])[Te]1	[H]C1=C([H])C2=C([H])[C2](C(C([H])([H])[H])(C([H])([H])[H])C([H])([H])[H])[Te][C1]([H])=C2C([H])=C1[H]	0.027396
179921	[H]C1=C([H])SC(C(=O)[O][Sn]([C]2=C([H])C([H])=C([H])C([H])=C2[H])([C]2=C([H])C([H])=C([H])C([H])=C2[H])[C]2=C([H])C([H])=C([H])C([H])=C2[H])=N1	[H]C1=C([H])SC(C(=O)[O4][Sn]([C1]2=C([H])C([H])=C([H])C([H])=C2[H])([C2]2=C([H])C([H])=C([H])C([H])=C2[H])[C3]2=C([H])C([H])=C([H])C([H])=C2[H])=N1	0.68036
182741	[H]C1=C([H])C([H])=C([N-]2[Tl-3][S-]~[P-]~23N(C(C([H])([H])[H])(C([H])([H])[H])C([H])([H])[H])[Si](C([H])([H])[H])(C([H])([H])[H])N3C(C([H])([H])[H])(C([H])([H])[H])C([H])([H])[H])C([H])=C1[H]	[H]C1=C([H])C([H])=C([N5-]2[Tl-3][S6-]~[P-]~23[N3](C(C([H])([H])[H])(C([H])([H])[H])C([H])([H])[H])[Si]([C1]([H])([H])[H])([C2]([H])([H])[H])[N4]3C(C([H])([H])[H])(C([H])([H])[H])C([H])([H])[H])C([H])=C1[H]	0.295054
183305	[H]C1=C(C2=C([H])C([H])=[N+]([Cu@OH8+7]([N-2])([N-2])([O-])([O-])[N+]3=C([H])C([H])=C(C4=C([H])C([H])=[N+]([Cu+2])C([H])=C4[H])C([H])=C3[H])C([H])=C2[H])C([H])=C([H])[N+]([Cu+2])=C1[H]	[H]C1=C(C2=C([H])C([H])=[N3+]([Cu+7]([N4+]3=C([H])C([H])=C(C4=C([H])C([H])=[N2+]([Cu+2])C([H])=C4[H])C([H])=C3[H])([N5-2])([N6-2])([O7-])[O8-])C([H])=C2[H])C([H])=C([H])[N1+]([Cu+2])=C1[H]	0.797842

Sheet1

2051215	[H]C1=C([H])C([H])=[C]([Sn]([C]2=C([H])C([H])=C([H])C([H])=C2[H])([C]2=C([H])C([H])=C([H])C([H])=C2[H])[Ba-5]([O+]2C([H])([H])C([H])([H])C([H])([H])C2([H])[H])([O+]2C([H])([H])C([H])([H])C([H])([H])C2([H])[H])([O+]2C([H])([H])C([H])([H])C([H])([H])C2([H])[H])([O+]2C([H])([H])C([H])([H])C([H])([H])C2([H])[H])[Sn]([C]2=C([H])C([H])=C([H])C([H])=C2[H])([C]2=C([H])C([H])=C([H])C([H])=C2[H])[C]2=C([H])C([H])=C([H])C([H])=C2[H])C([H])=C1[H]	[H]C1=C([H])C([H])=[C6]([Sn]([Ba-5]([Sn]([C9]2=C([H])C([H])=C([H])C([H])=C2[H])([C10]2=C([H])C([H])=C([H])C([H])=C2[H])[C11]2=C([H])C([H])=C([H])C([H])=C2[H])([O1+]2C([H])([H])C([H])([H])C([H])([H])C2([H])[H])([O2+]2C([H])([H])C([H])([H])C([H])([H])C2([H])[H])([O3+]2C([H])([H])C([H])([H])C([H])([H])C2([H])[H])([O4+]2C([H])([H])C([H])([H])C([H])([H])C2([H])[H])[O5+]2C([H])([H])C([H])([H])C([H])([H])C2([H])[H])([C7]2=C([H])C([H])=C([H])C([H])=C2[H])[C8]2=C([H])C([H])=C([H])C([H])=C2[H])C([H])=C1[H]	0.629172
2074417	[H]C1~C(C(C([H])([H])[H])(C([H])([H])[H])C([H])([H])[H])~[N-](C2=C(C([H])(C([H])([H])[H])C([H])([H])[H])C([H])=C([H])C([H])=C2C([H])(C([H])([H])[H])C([H])([H])[H])[Fe+5]([C]#CC2=C([H])C([H])=C([H])C([H])=C2[H])([N+]2=C([H])C([H])=C([H])C([H])=C2[H])[N-](C2=C(C([H])(C([H])([H])[H])C([H])([H])[H])C([H])=C([H])C([H])=C2C([H])(C([H])([H])[H])C([H])([H])[H])~C~1C(C([H])([H])[H])(C([H])([H])[H])C([H])([H])[H]	[H]C1~C(C(C([H])([H])[H])(C([H])([H])[H])C([H])([H])[H])~[N2-](C2=C(C([H])(C([H])([H])[H])C([H])([H])[H])C([H])=C([H])C([H])=C2C([H])(C([H])([H])[H])C([H])([H])[H])[Fe+5]([C1]#CC2=C([H])C([H])=C([H])C([H])=C2[H])([N4+]2=C([H])C([H])=C([H])C([H])=C2[H])[N3-](C2=C(C([H])(C([H])([H])[H])C([H])([H])[H])C([H])=C([H])C([H])=C2C([H])(C([H])([H])[H])C([H])([H])[H])~C~1C(C([H])([H])[H])(C([H])([H])[H])C([H])([H])[H]	0.484199
2075115	[H]C([H])([H])C1(C([H])([H])[H])O[Si+]23(C([H])([H])C([H])([H])C([H])([H])Cl)OC([H])([H])C([H])([H])[N+]2(C([H])([H])C([H])([H])O3)C1([H])[H]	[H]C([H])([H])C1(C([H])([H])[H])C([H])([H])[N2+]23C([H])([H])C([H])([H])[O4][Si+]2([C1]([H])([H])C([H])([H])C([H])([H])Cl)([O3]1)[O5]C([H])([H])C3([H])[H]	0.130157
2075193_as	[H]C1=C([H])C([H])=C2OC([H])([H])C([H])([H])OC([H])([H])C([H])([H])OC([H])([H])C([H])([H])OC3=C(OC([H])([H])C([H])([H])OC([H])([H])C([H])([H])OC([H])([H])C([H])([H])OC2=C1[H])C([H])=C([H])C([H])=C3[H]	[H]C1=C([H])C([H])=C2OC([H])([H])C([H])([H])OC([H])([H])C([H])([H])OC([H])([H])C([H])([H])OC3=C(OC([H])([H])C([H])([H])OC([H])([H])C([H])([H])OC([H])([H])C([H])([H])OC2=C1[H])C([H])=C([H])C([H])=C3[H]	0.162695
2076769	[H]C1=C([H])C2=C(C([H])=C1[H])[N+]1=C(C([H])=C2[H])C2=C([H])C([H])=C(Cl)N2[B-]1(F)F	[H]C1=C([H])C2=C(C([H])=C1[H])[N4+]1=C(C([H])=C2[H])C2=C([H])C([H])=C(Cl)[N3]2[B-]1([F1])[F2]	0.052945
2076771	[H]C1=C([H])C2=C(C([H])=C1[H])[N+]1=C(C([H])=C2[H])C2=C([H])C([H])=C(I)N2[B-]1(F)F	[H]C1=C([H])C2=C(C([H])=C1[H])[N4+]1=C(C([H])=C2[H])C2=C([H])C([H])=C(I)[N3]2[B-]1([F1])[F2]	0.071182
2083565	[H]C1=C([H])C([H])=C(S[P]([W@OH6+7]([C]#[O+])([C]#[O+])([C]#[O+])([C]#[O+])[C]#[O+])[W@OH3+7]([C]#[O+])([C]#[O+])([C]#[O+])([C]#[O+])[C]#[O+])C([H])=C1[H]	[H]C1=C([H])C([H])=C(S[P6]([W+7]([C1]#[O+])([C2]#[O+])([C3]#[O+])([C4]#[O+])[C5]#[O+])[W+7]([C7]#[O+])([C8]#[O+])([C9]#[O+])([C10]#[O+])[C11]#[O+])C([H])=C1[H]	0.668999
2083570	[H]C1=C([H])C([H])=C([Se@+]2[W+8]3([C]#[O+])([C]#[O+])([C]#[O+])([C]#[O+])[Se@@+](C4=C([H])C([H])=C([H])C([H])=C4[H])[W+8]23([C]#[O+])([C]#[O+])([C]#[O+])[C]#[O+])C([H])=C1[H]	[H]C1=C([H])C([H])=[C1]([Se+]2[W+8]3([C3]#[O+])([C4]#[O+])([C5]#[O+])([C6]#[O+])[Se+]([C2]4=C([H])C([H])=C([H])C([H])=C4[H])[W+8]23([C7]#[O+])([C8]#[O+])([C9]#[O+])[C10]#[O+])C([H])=C1[H]	0.301003

Sheet1

2087213	[H]C(=NC1=C(C([H])(C([H])([H])[H])C([H])([H])[H])C([H])=C([H])C([H])=C1C([H])(C([H])([H])[H])C([H])([H])[H])C1=C2C(=C([H])C(C(C([H])([H])[H])(C([H])([H])[H])C([H])([H])[H])=C1[H])C1=C([H])C(C(C([H])([H])[H])(C([H])([H])[H])C([H])([H])[H])=C([H])C3=C1[N]2[Ga@-]1([Se][Ga@-]2([Se]1)[N]1C4=C(C([H])=NC5=C(C([H])(C([H])([H])[H])C([H])([H])[H])C([H])=C([H])C([H])=C5C([H])(C([H])([H])[H])C([H])([H])[H])C([H])=C(C(C([H])([H])[H])(C([H])([H])[H])C([H])([H])[H])C([H])=C4C4=C([H])C(C(C([H])([H])[H])(C([H])([H])[H])C([H])([H])[H])=C([H])C(=C41)C([H])=[N+]2C1=C(C([H])(C([H])([H])[H])C([H])([H])[H])C([H])=C([H])C([H])=C1C([H])(C([H])([H])[H])C([H])([H])[H])[N+](C1=C(C([H])(C([H])([H])[H])C([H])([H])[H])C([H])=C([H])C([H])=C1C([H])(C([H])([H])[H])C([H])([H])[H])=C3[H]	[H]C(=NC1=C(C([H])(C([H])([H])[H])C([H])([H])[H])C([H])=C([H])C([H])=C1C([H])(C([H])([H])[H])C([H])([H])[H])C1=C2C(=C([H])C(C(C([H])([H])[H])(C([H])([H])[H])C([H])([H])[H])=C1[H])C1=C([H])C(C(C([H])([H])[H])(C([H])([H])[H])C([H])([H])[H])=C([H])C3=C1[N1]2[Ga-]1([Se][Ga-]2([Se]1)[N3]1C4=C(C([H])=NC5=C(C([H])(C([H])([H])[H])C([H])([H])[H])C([H])=C([H])C([H])=C5C([H])(C([H])([H])[H])C([H])([H])[H])C([H])=C(C(C([H])([H])[H])(C([H])([H])[H])C([H])([H])[H])C([H])=C4C4=C([H])C(C(C([H])([H])[H])(C([H])([H])[H])C([H])([H])[H])=C([H])C(=C41)C([H])=[N4+]2C1=C(C([H])(C([H])([H])[H])C([H])([H])[H])C([H])=C([H])C([H])=C1C([H])(C([H])([H])[H])C([H])([H])[H])[N2+](C1=C(C([H])(C([H])([H])[H])C([H])([H])[H])C([H])=C([H])C([H])=C1C([H])(C([H])([H])[H])C([H])([H])[H])=C3[H]	0.431174
2087214	[H]C(=NC1=C(C([H])(C([H])([H])[H])C([H])([H])[H])C([H])=C([H])C([H])=C1C([H])(C([H])([H])[H])C([H])([H])[H])C1=C2C(=C([H])C(C(C([H])([H])[H])(C([H])([H])[H])C([H])([H])[H])=C1[H])C1=C([H])C(C(C([H])([H])[H])(C([H])([H])[H])C([H])([H])[H])=C([H])C3=C1[N]2[Ga]([Cr@OH30+7]([C]#[O+])([C]#[O+])([C]#[O+])([C]#[O+])[C]#[O+])[N+](C1=C(C([H])(C([H])([H])[H])C([H])([H])[H])C([H])=C([H])C([H])=C1C([H])(C([H])([H])[H])C([H])([H])[H])=C3[H]	[H]C(=NC1=C(C([H])(C([H])([H])[H])C([H])([H])[H])C([H])=C([H])C([H])=C1C([H])(C([H])([H])[H])C([H])([H])[H])C1=C2C(=C([H])C(C(C([H])([H])[H])(C([H])([H])[H])C([H])([H])[H])=C1[H])C1=C([H])C(C(C([H])([H])[H])(C([H])([H])[H])C([H])([H])[H])=C([H])C3=C1[N7]2[Ga]([Cr+7]([C1]#[O+])([C2]#[O+])([C3]#[O+])([C4]#[O+])[C5]#[O+])[N6+](C1=C(C([H])(C([H])([H])[H])C([H])([H])[H])C([H])=C([H])C([H])=C1C([H])(C([H])([H])[H])C([H])([H])[H])=C3[H]	0.535425
2087215	[H]C1=C([H])C(C([H])(C([H])([H])[H])C([H])([H])[H])=C([N+]2=C([H])C3=C4C(=C([H])C(C(C([H])([H])[H])(C([H])([H])[H])C([H])([H])[H])=C3[H])C3=C([H])C(C(C([H])([H])[H])(C([H])([H])[H])C([H])([H])[H])=C([H])C5=C3[N]4[Al-2]2([C]([H])([H])[H])([C]([H])([H])[H])[N+](C2=C(C([H])(C([H])([H])[H])C([H])([H])[H])C([H])=C([H])C([H])=C2C([H])(C([H])([H])[H])C([H])([H])[H])=C5[H])C(C([H])(C([H])([H])[H])C([H])([H])[H])=C1[H]	[H]C1=C([H])C(C([H])(C([H])([H])[H])C([H])([H])[H])=C([N4+]2=C([H])C3=C4C(=C([H])C(C(C([H])([H])[H])(C([H])([H])[H])C([H])([H])[H])=C3[H])C3=C([H])C(C(C([H])([H])[H])(C([H])([H])[H])C([H])([H])[H])=C([H])C5=C3[N3]4[Al-2]2([C1]([H])([H])[H])([C2]([H])([H])[H])[N5+](C2=C(C([H])(C([H])([H])[H])C([H])([H])[H])C([H])=C([H])C([H])=C2C([H])(C([H])([H])[H])C([H])([H])[H])=C5[H])C(C([H])(C([H])([H])[H])C([H])([H])[H])=C1[H]	0.733834
2091598	[H]C([H])([H])[O+]1C([H])([H])C([H])([H])[O+](C([H])([H])[H])[Mg-4]123([O+](C([H])([H])C(F)(F)F)[B-](OC([H])([H])C(F)(F)F)(OC([H])([H])C(F)(F)F)[O+]2C([H])([H])C(F)(F)F)[O+](C([H])([H])C(F)(F)F)[B-](OC([H])([H])C(F)(F)F)(OC([H])([H])C(F)(F)F)[O+]3C([H])([H])C(F)(F)F	[H]C([H])(C(F)(F)F)[O1][B-]1([O2]C([H])([H])C(F)(F)F)[O3+](C([H])([H])C(F)(F)F)[Mg-4]23([O4+]1C([H])([H])C(F)(F)F)([O7+](C([H])([H])C(F)(F)F)[B-]([O5]C([H])([H])C(F)(F)F)([O6]C([H])([H])C(F)(F)F)[O8+]2C([H])([H])C(F)(F)F)[O9+](C([H])([H])[H])C([H])([H])C([H])([H])[O10+]3C([H])([H])[H]	0.134286
2096425	[H]C1=C([H])C2=C3[C](=C1[H])[Al-]1([C]([H])([H])C([H])([H])[H])[C]4=C([H])C([H])=C([H])C5=C4[N+](=C(C([H])([H])[H])C([H])=C5[H])[Li-2]([N+]3=C(C([H])([H])[H])C([H])=C2[H])[N+]2=C(C([H])([H])[H])C([H])=C([H])C3=C2[C]1=C([H])C([H])=C3[H]	[H]C1=C([H])C([H])=[C2]2C3=C1C([H])=C([H])C(C([H])([H])[H])=[N5+]3[Li-2]1[N6+]3=C(C([H])([H])[H])C([H])=C([H])C4=C3[C1](=C([H])C([H])=C4[H])[Al-]2([C4]([H])([H])C([H])([H])[H])[C3]2=C([H])C([H])=C([H])C3=C2[N7+]1=C(C([H])([H])[H])C([H])=C3[H]	0.139588

Sheet1

2104359	[H]C1=C([H])C([H])=[N+]2C(=C1[H])C1=C([H])C([H])=C([H])C3=[N+]1[Ru@OH23+7]214([N+]2=C([H])C([H])=C([H])C([H])=C2C2=C([H])C([H])=C([H])C(=[N+]21)C1=C([H])C([H])=C([H])C([H])=[N+]14)[N+]1=C([H])C([H])=C([H])C([H])=C31	[H]C1=C([H])C([H])=[N1+]2C(=C1[H])C1=[N5+]3C(=C([H])C([H])=C1[H])C1=C([H])C([H])=C([H])C([H])=[N3+]1[Ru+7]2314[N2+]2=C([H])C([H])=C([H])C([H])=C2C2=[N6+]1C(=C([H])C([H])=C2[H])C1=C([H])C([H])=C([H])C([H])=[N4+]14	0.055472
2150100	[H]C1=C([H])C([H])=C2C(=C1[H])C(=O)C([H])([H])C1=C(C([H])=C([H])C([H])=C1[H])N2C(=O)N([H])[H]	[H]C1=C([H])C([H])=C2C(=C1[H])C(=O)C([H])([H])C1=C(C([H])=C([H])C([H])=C1[H])N2C(=O)N([H])[H]	0.266783
229783	[H]C1=C(C([H])([H])[H])C([H])=C(C([H])([H])[H])C(N2[C]3N(C([H])=C2[H])C2=[N+](C(C([H])([H])[H])(C([H])([H])[H])C([H])([H])O2)[Rh@SP3+5]3([Br])[C]#[O+])=C1C([H])([H])[H]	[H]C1=C(C([H])([H])[H])C([H])=C(C([H])([H])[H])C(N2C([H])=C([H])N3C4=[N4+](C(C([H])([H])[H])(C([H])([H])[H])C([H])([H])O4)[Rh+5]([Br1])([C2]#[O+])[C3]23)=C1C([H])([H])[H]	0.178697
232528	[H]C([H])([H])[P+](C([H])([H])[H])(C([H])([H])[H])[Au@SP3+5]([I])([I])[I]	[H]C([H])([H])[P4+](C([H])([H])[H])(C([H])([H])[H])[Au+5]([I1])([I2])[I3]	0.04741
232536	[H]C1=C([H])C([H])=C([P+]([Au+3][Br])(C2=C([H])C([H])=C([H])C([H])=C2[H])C([H])([H])[P+]([Au+3][Br])(C2=C([H])C([H])=C([H])C([H])=C2[H])C2=C([H])C([H])=C([H])C([H])=C2[H])C([H])=C1[H]	[H]C1=C([H])C([H])=C([P2+]([Au+3][Br1])(C2=C([H])C([H])=C([H])C([H])=C2[H])C([H])([H])[P4+]([Au+3][Br3])(C2=C([H])C([H])=C([H])C([H])=C2[H])C2=C([H])C([H])=C([H])C([H])=C2[H])C([H])=C1[H]	0.710424
232591	[H]C([H])([H])[C]12=[P+]3=[C]4(C([H])([H])[H])=[C]5(C([H])([H])[H])=[C]=1(C([H])([H])[H])~[Fe+]~3~2~4~51678~[P+]2=[C]~1(C([H])([H])[H])=[C]~6(C([H])([H])[H])=[C]~7(C([H])([H])[H])=[C]=2~8C([H])([H])[H]	[H]C([H])([H])[C1]12=[C5]3(C([H])([H])[H])=[C7]4(C([H])([H])[H])=[C3]5(C([H])([H])[H])=[P9+]=1~[Fe+]~2~5~3~41678~[C2]2(C([H])([H])[H])=[C6]~1(C([H])([H])[H])=[C8]~6(C([H])([H])[H])=[C4]~7(C([H])([H])[H])=[P10+]=2~8	0.022815
234033	[H]C1=C(C2=C([H])C([H])=[N+]([Pb+2]3([N-2])([Cl])([Cl])[Cl+][Pb][Cl+]3)C([H])=C2[H])C([H])=C([H])[N+]([Pb-])=C1[H]	[H]C1=C(C2=C([H])C([H])=[N6+]([Pb+2]3([Cl4])([Cl5])([N7-2])[Cl2+][Pb][Cl3+]3)C([H])=C2[H])C([H])=C([H])[N1+]([Pb-])=C1[H]	0.646602
234071	[H]C1=C([H])C([H])=[C]2C(=C1[H])C([H])([H])[N@+]1(C(C([H])([H])[H])(C([H])([H])[H])C([H])([H])[H])C([H])([H])C3=C([H])C([H])=C([H])C([H])=[C]3[Bi@+]21[Cl]	[H]C1=C([H])C([H])=[C1]2C(=C1[H])C([H])([H])[N4+]1(C(C([H])([H])[H])(C([H])([H])[H])C([H])([H])[H])C([H])([H])C3=C([H])C([H])=C([H])C([H])=[C2]3[Bi+]21[Cl3]	0.112779
236914	[H]C([H])([H])C([H])([H])[S@+]1C([H])([H])C([H])([H])[S@@+](C([H])([H])C([H])([H])[H])[Mo@OH22+9]1(=[O])(=[O])([Cl])[Cl]	[H]C([H])([H])C([H])([H])[S5+]1C([H])([H])C([H])([H])[S6+](C([H])([H])C([H])([H])[H])[Mo+9]1([Cl1])([Cl2])(=[O3])=[O4]	0.058951
2378332	[H]C([H])([H])C([H])([H])C([H])([H])N(C1~[S-][Au@SP3+5]2([S-]~1)[S-]~C(N(C([H])([H])C([H])([H])C([H])([H])[H])C([H])([H])C([H])([H])C([H])([H])[H])~[S-]2)C([H])([H])C([H])([H])C([H])([H])[H]	[H]C([H])([H])C([H])([H])C([H])([H])N(C1~[S1-][Au+5]2([S2-]~1)[S3-]~C(N(C([H])([H])C([H])([H])C([H])([H])[H])C([H])([H])C([H])([H])C([H])([H])[H])~[S4-]2)C([H])([H])C([H])([H])C([H])([H])[H]	0.151653
243849	[H]C1=C([H])C2=C([H])C3=C(C([H])=C([H])C([H])=C3[H])C(C([H])([H])[N@@+]3(C([H])([H])[H])C([H])([H])C([H])([H])[N@@+](C([H])([H])[H])(C([H])([H])C4=C5C([H])=C([H])C([H])=C([H])C5=C([H])C5=C4C([H])=C([H])C([H])=C5[H])[Pd@SP1+5]3([Cl])[Cl])=C2C([H])=C1[H]	[H]C1=C([H])C2=C([H])C3=C(C([H])=C([H])C([H])=C3[H])C(C([H])([H])[N3+]3(C([H])([H])[H])C([H])([H])C([H])([H])[N4+](C([H])([H])[H])(C([H])([H])C4=C5C([H])=C([H])C([H])=C([H])C5=C([H])C5=C4C([H])=C([H])C([H])=C5[H])[Pd+5]3([Cl1])[Cl2])=C2C([H])=C1[H]	0.291088

Sheet1

244175	[H]C([H])OC1=C([H])C([H])=C([H])C([H])=C1C([H])([H])[N@@+]1([H])[C@]([H])(C2=C([H])C([H])=C([H])C([H])=C2[H])[C@@]([H])(C2=C([H])C([H])=C([H])C([H])=C2[H])[N@+]([H])(C([H])([H])C2=C([H])C([H])=C([H])C([H])=C2OC([H])([H])[H])[Zn-5]1([Cl])[Cl]	[H]C([H])OC1=C([H])C([H])=C([H])C([H])=C1C([H])([H])[N3+]1([H])C([H])(C2=C([H])C([H])=C([H])C([H])=C2[H])C([H])(C2=C([H])C([H])=C([H])C([H])=C2[H])[N4+]([H])(C([H])([H])C2=C([H])C([H])=C([H])C([H])=C2OC([H])([H])[H])[Zn-5]1([Cl1])[Cl2]	0.991014
251255	[H]C1=NC(C([H])([H])N2[C]([Hg-3][C]3N(C([H])([H])C4=NC([H])=C([H])C([H])=C4[H])C([H])=C([H])N3C([H])([H])C3=NC([H])=C([H])C([H])=C3[H])N(C([H])([H])C3=C([H])C([H])=C([H])C([H])=N3)C([H])=C2[H])=C([H])C([H])=C1[H]	[H]C1=NC(C([H])([H])N2C([H])=C([H])N(C([H])([H])C3=C([H])C([H])=C([H])C([H])=N3)[C1]2[Hg-3][C2]2N(C([H])([H])C3=NC([H])=C([H])C([H])=C3[H])C([H])=C([H])N2C([H])([H])C2=NC([H])=C([H])C([H])=C2[H])=C([H])C([H])=C1[H]	0.692237
2515638	[H]C1([H])C([H])([H])C([H])([H])C([H])([N+]([H])([H])C([H])([H])[H])C([H])([H])C1([H])[H]	[H]C1([H])C([H])([H])C([H])([H])C([H])([N+]([H])([H])C([H])([H])[H])C([H])([H])C1([H])[H]	0.052291
253279	[H]C1=C([H])C([H])=C([P+](C2=C([H])C([H])=C([H])C([H])=C2[H])(C2=C([H])C([H])=C([H])C([H])=C2[H])C2=C([H])C([H])=[C]([Pd@SP2+5]([N+]#CC3=C([H])C([H])=C(C4=C([H])C([H])=C(C#[N+][Pd@SP2+5]([C]5=C([H])C([H])=C([P+](C6=C([H])C([H])=C([H])C([H])=C6[H])(C6=C([H])C([H])=C([H])C([H])=C6[H])C6=C([H])C([H])=C([H])C([H])=C6[H])C([H])=C5[H])([P+](C5=C([H])C([H])=C([H])C([H])=C5[H])(C5=C([H])C([H])=C([H])C([H])=C5[H])C5=C([H])C([H])=C([H])C([H])=C5[H])[P+](C5=C([H])C([H])=C([H])C([H])=C5[H])(C5=C([H])C([H])=C([H])C([H])=C5[H])C5=C([H])C([H])=C([H])C([H])=C5[H])C([H])=C4[H])C([H])=C3[H])([P+](C3=C([H])C([H])=C([H])C([H])=C3[H])(C3=C([H])C([H])=C([H])C([H])=C3[H])C3=C([H])C([H])=C([H])C([H])=C3[H])[P+](C3=C([H])C([H])=C([H])C([H])=C3[H])(C3=C([H])C([H])=C([H])C([H])=C3[H])C3=C([H])C([H])=C([H])C([H])=C3[H])C([H])=C2[H])C([H])=C1[H]	[H]C1=C([H])C([H])=C([P+](C2=C([H])C([H])=C([H])C([H])=C2[H])(C2=C([H])C([H])=C([H])C([H])=C2[H])C2=C([H])C([H])=[C1]([Pd+5]([P2+](C3=C([H])C([H])=C([H])C([H])=C3[H])(C3=C([H])C([H])=C([H])C([H])=C3[H])C3=C([H])C([H])=C([H])C([H])=C3[H])([N3+]#CC3=C([H])C([H])=C(C4=C([H])C([H])=C(C#[N7+][Pd+5]([C5]5=C([H])C([H])=C([P+](C6=C([H])C([H])=C([H])C([H])=C6[H])(C6=C([H])C([H])=C([H])C([H])=C6[H])C6=C([H])C([H])=C([H])C([H])=C6[H])C([H])=C5[H])([P6+](C5=C([H])C([H])=C([H])C([H])=C5[H])(C5=C([H])C([H])=C([H])C([H])=C5[H])C5=C([H])C([H])=C([H])C([H])=C5[H])[P8+](C5=C([H])C([H])=C([H])C([H])=C5[H])(C5=C([H])C([H])=C([H])C([H])=C5[H])C5=C([H])C([H])=C([H])C([H])=C5[H])C([H])=C4[H])C([H])=C3[H])[P4+](C3=C([H])C([H])=C([H])C([H])=C3[H])(C3=C([H])C([H])=C([H])C([H])=C3[H])C3=C([H])C([H])=C([H])C([H])=C3[H])C([H])=C2[H])C([H])=C1[H]	2.531577
253288	[H]C1=C([H])C(C([H])([H])[H])=C([O][Ti+5]([O]C2=C(C([H])([H])[H])C([H])=C([H])C([H])=C2C([H])([H])[H])([O]C2=C(C([H])([H])[H])C([H])=C([H])C([H])=C2C([H])([H])[H])[O]C2=C(C([H])([H])[H])C([H])=C([H])C([H])=C2C([H])([H])[H])C(C([H])([H])[H])=C1[H]	[H]C1=C([H])C(C([H])([H])[H])=C([O1][Ti+5]([O2]C2=C(C([H])([H])[H])C([H])=C([H])C([H])=C2C([H])([H])[H])([O3]C2=C(C([H])([H])[H])C([H])=C([H])C([H])=C2C([H])([H])[H])[O4]C2=C(C([H])([H])[H])C([H])=C([H])C([H])=C2C([H])([H])[H])C(C([H])([H])[H])=C1[H]	0.347849

Sheet1

253291	[H]C1=C([H])C([H])=[N+]([Rb-4]234[O+](C([H])([H])C(C([H])([H])[H])(C([H])([H])[H])C([H])([H])[H])[Ti@+6]5([O]C([H])([H])C(C([H])([H])[H])(C([H])([H])[H])C([H])([H])[H])([O+]2C([H])([H])C(C([H])([H])[H])(C([H])([H])[H])C([H])([H])[H])[O+](C([H])([H])C(C([H])([H])[H])(C([H])([H])[H])C([H])([H])[H])[Rb-4]2([N+]6=C([H])C([H])=C([H])C([H])=C6[H])([O+]5C([H])([H])C(C([H])([H])[H])(C([H])([H])[H])C([H])([H])[H])[O+](C([H])([H])C(C([H])([H])[H])(C([H])([H])[H])C([H])([H])[H])[Ti@+6]([O]C([H])([H])C(C([H])([H])[H])(C([H])([H])[H])C([H])([H])[H])([O+]3C([H])([H])C(C([H])([H])[H])(C([H])([H])[H])C([H])([H])[H])([O+]4C([H])([H])C(C([H])([H])[H])(C([H])([H])[H])C([H])([H])[H])[O+]2C([H])([H])C(C([H])([H])[H])(C([H])([H])[H])C([H])([H])[H])C([H])=C1[H]	[H]C1=C([H])C([H])=[N1+]([Rb-4]234[O2+](C([H])([H])C(C([H])([H])[H])(C([H])([H])[H])C([H])([H])[H])[Ti+6]5([O12]C([H])([H])C(C([H])([H])[H])(C([H])([H])[H])C([H])([H])[H])([O4+]2C([H])([H])C(C([H])([H])[H])(C([H])([H])[H])C([H])([H])[H])[O8+](C([H])([H])C(C([H])([H])[H])(C([H])([H])[H])C([H])([H])[H])[Rb-4]2([N6+]6=C([H])C([H])=C([H])C([H])=C6[H])([O7+](C([H])([H])C(C([H])([H])[H])(C([H])([H])[H])C([H])([H])[H])[Ti+6]([O11]C([H])([H])C(C([H])([H])[H])(C([H])([H])[H])C([H])([H])[H])([O3+]3C([H])([H])C(C([H])([H])[H])(C([H])([H])[H])C([H])([H])[H])([O5+]4C([H])([H])C(C([H])([H])[H])(C([H])([H])[H])C([H])([H])[H])[O9+]2C([H])([H])C(C([H])([H])[H])(C([H])([H])[H])C([H])([H])[H])[O10+]5C([H])([H])C(C([H])([H])[H])(C([H])([H])[H])C([H])([H])[H])C([H])=C1[H]	0.509626
2535798	[H]C1=C([H])C([H])=C(P(C2=C([H])C([H])=C([H])C([H])=C2[H])C([H])([H])C([H])([H])[P+](C2=C([H])C([H])=C([H])C([H])=C2[H])(C2=C([H])C([H])=C([H])C([H])=C2[H])[Mo@OH12+7]2([C]#[O+])([C]#[O+])([C]#[O+])[P+](C3=C([H])C([H])=C([H])C([H])=C3[H])(C3=C([H])C([H])=C([H])C([H])=C3[H])C([H])([H])C([H])([H])[P+]2(C2=C([H])C([H])=C([H])C([H])=C2[H])C2=C([H])C([H])=C([H])C([H])=C2[H])C([H])=C1[H]	[H]C1=C([H])C([H])=C(P(C2=C([H])C([H])=C([H])C([H])=C2[H])C([H])([H])C([H])([H])[P4+](C2=C([H])C([H])=C([H])C([H])=C2[H])(C2=C([H])C([H])=C([H])C([H])=C2[H])[Mo+7]2([C1]#[O+])([C2]#[O+])([C3]#[O+])[P5+](C3=C([H])C([H])=C([H])C([H])=C3[H])(C3=C([H])C([H])=C([H])C([H])=C3[H])C([H])([H])C([H])([H])[P6+]2(C2=C([H])C([H])=C([H])C([H])=C2[H])C2=C([H])C([H])=C([H])C([H])=C2[H])C([H])=C1[H]	0.303471
602743	[H]C1=C([H])C(C([H])([H])[H])=C([H])C([H])=C1C(=O)[S][Sb@TB18]12([O+]=C(C3=C([H])C([H])=C(C([H])([H])[H])C([H])=C3[H])[S]1)[O+]=C(C1=C([H])C([H])=C(C([H])([H])[H])C([H])=C1[H])[S]2	[H]C1=C([H])C(C([H])([H])[H])=C([H])C([H])=C1C(=O)[S5][Sb]12([O1+]=C(C3=C([H])C([H])=C(C([H])([H])[H])C([H])=C3[H])[S3]1)[O2+]=C(C1=C([H])C([H])=C(C([H])([H])[H])C([H])=C1[H])[S4]2	0.395392
613741	[H]C1=C([H])C2=[N+](C([H])=C1[H])[In@OH13-3]1([Br])([Br])([Br])[N+]3=C(C([H])=C([H])C([H])=C3[H])C([H])([H])[N+]1([H])C2([H])[H]	[H]C1=C([H])C2=[N4+](C([H])=C1[H])[In-3]1([Br1])([Br2])([Br3])[N5+]3=C(C([H])=C([H])C([H])=C3[H])C([H])([H])[N6+]1([H])C2([H])[H]	0.13586
716039	[H]C1=C([H])C([H])=C2C(=C1[H])N([H])C1=[N+]2[Ho+8]23([N-2])([N-2])([S]1)[N+]1=C(N([H])C4=C1C([H])=C([H])C([H])=C4[H])[S@@+]2[Ho+8]124([S]C5=[N+]1C1=C([H])C([H])=C([H])C([H])=C1N5[H])([N]1C5=C([H])C([H])=C([H])C([H])=C5[N+]5=C1C1=[N+]([Ho+3]5)C5=C([H])C([H])=C([H])C([H])=C5[N]12)[N+]1=C(N([H])C2=C1C([H])=C([H])C([H])=C2[H])[S@+]34	[H]C1=C([H])C([H])=C2C(=C1[H])N([H])C1=[N3+]2[Ho+8]234([N4+]5=C(N([H])C6=C5C([H])=C([H])C([H])=C6[H])[S8+]2[Ho+8]25([N12-2])([N13-2])([S7+]3C3=[N10+]2C2=C(C([H])=C([H])C([H])=C2[H])N3[H])[N11+]2=C(N([H])C3=C([H])C([H])=C([H])C([H])=C32)[S14]5)([N5]2C3=C([H])C([H])=C([H])C([H])=C3[N2+]3=C2C2=[N1+]([Ho+3]3)C3=C([H])C([H])=C([H])C([H])=C3[N6]24)[S9]1	0.500802
780531	[H]C1=C([H])C([H])=C(C2=N[N+](=C(C([H])([H])[H])C([H])([H])[H])[Re@OH20+8](=[O])([Cl])([Cl])([P+](C3=C([H])C([H])=C([H])C([H])=C3[H])(C3=C([H])C([H])=C([H])C([H])=C3[H])C3=C([H])C([H])=C([H])C([H])=C3[H])[O]2)C([H])=C1[H]	[H]C1=C([H])C([H])=C(C2=N[N3+](=C(C([H])([H])[H])C([H])([H])[H])[Re+8]([Cl1])([Cl2])(=[O4])([P6+](C3=C([H])C([H])=C([H])C([H])=C3[H])(C3=C([H])C([H])=C([H])C([H])=C3[H])C3=C([H])C([H])=C([H])C([H])=C3[H])[O5]2)C([H])=C1[H]	0.31981

Sheet1

791724	[H]C1=C([H])C2=C(C([H])=C1[H])[N+]([Mn@OH2+7]([N]=C=S)([N]=C=S)([N]=C=S)([N]=C=S)[N+]1=C(C([H])([H])[H])N([H])C3=C1C([H])=C([H])C([H])=C3[H])=C(C([H])([H])[H])N2[H]	[H]C1=C([H])C2=C(C([H])=C1[H])[N1+]([Mn+7]([N2+]1=C(C([H])([H])[H])N([H])C3=C1C([H])=C([H])C([H])=C3[H])([N3]=C=S)([N4]=C=S)([N5]=C=S)[N6]=C=S)=C(C([H])([H])[H])N2[H]	0.354161
801553	[H][C]([H])([H])[Al-]1([C]([H])([H])[H])[C]([H])([H])([H])[Tm@OH9+7]23([C]1([H])([H])[H])([C]([H])([H])([H])[Al-]([C]([H])([H])[H])([C]([H])([H])[H])[C]2([H])([H])[H])[C]([H])([H])([H])[Al-]([C]([H])([H])[H])([C]([H])([H])[H])[C]3([H])([H])[H]	[H][C1]([H])([H])[Al-]1([C2]([H])([H])[H])[C3]([H])([H])([H])[Tm+7]23([C4]1([H])([H])[H])([C7]([H])([H])([H])[Al-]([C5]([H])([H])[H])([C6]([H])([H])[H])[C8]2([H])([H])[H])[C11]([H])([H])([H])[Al-]([C9]([H])([H])[H])([C10]([H])([H])[H])[C12]3([H])([H])[H]	0.101246
804893	[H]C1=C([H])C([H])=[N+]([Ce+9]2([Br])([Br])([Br])([N+]3=C([H])C([H])=C([H])C([H])=C3[H])([N+]3=C([H])C([H])=C([H])C([H])=C3[H])[N+]3=C(C([H])=C([H])C([H])=C3[H])C3=[N+]2C([H])=C([H])C([H])=C3[H])C([H])=C1[H]	[H]C1=C([H])C2=[N4+](C([H])=C1[H])[Ce+9]([Br1])([Br2])([Br3])([N6+]1=C([H])C([H])=C([H])C([H])=C1[H])([N7+]1=C([H])C([H])=C([H])C([H])=C1[H])([N8+]1=C([H])C([H])=C([H])C([H])=C1[H])[N5+]1=C2C([H])=C([H])C([H])=C1[H]	0.432575
804894	[H]C1([H])[O+]([Ce+8]([Br])([Br])([Br])([O+]2C([H])([H])C([H])([H])C([H])([H])[C@]2([H])C([H])([H])[H])([O+]2C([H])([H])C([H])([H])C([H])([H])[C@@]2([H])C([H])([H])[H])[O+]2C([H])([H])C([H])([H])C([H])([H])[C@@]2([H])C([H])([H])[H])[C@@]([H])(C([H])([H])[H])C([H])([H])C1([H])[H]	[H]C([H])([H])C1([H])C([H])([H])C([H])([H])C([H])([H])[O4+]1[Ce+8]([Br1])([Br2])([Br3])([O5+]1C([H])([H])C([H])([H])C([H])([H])C1([H])C([H])([H])[H])([O6+]1C([H])([H])C([H])([H])C([H])([H])C1([H])C([H])([H])[H])[O7+]1C([H])([H])C([H])([H])C([H])([H])C1([H])C([H])([H])[H]	0.217225
804896	[H]C([H])([H])C#[N+][La-9]([Br])([Br])([Br])([N+]#CC([H])([H])[H])([N+]#CC([H])([H])[H])([N+]#CC([H])([H])[H])[N+]#CC([H])([H])[H]	[H]C([H])([H])C#[N4+][La-9]([Br1])([Br2])([Br3])([N5+]#CC([H])([H])[H])([N6+]#CC([H])([H])[H])([N7+]#CC([H])([H])[H])[N8+]#CC([H])([H])[H]	1.116587
849384	[H]C1=C([H])C2=C(C([H])=C1[H])[N]([H])[Os+7]([H])([H])([P+](C([H])(C([H])([H])[H])C([H])([H])[H])(C([H])(C([H])([H])[H])C([H])([H])[H])C([H])(C([H])([H])[H])C([H])([H])[H])([P+](C([H])(C([H])([H])[H])C([H])([H])[H])(C([H])(C([H])([H])[H])C([H])([H])[H])C([H])(C([H])([H])[H])C([H])([H])[H])[N]2[H]	[H]C1=C([H])C2=C(C([H])=C1[H])[N2]([H])[Os+7]([H])([H])([P3+](C([H])(C([H])([H])[H])C([H])([H])[H])(C([H])(C([H])([H])[H])C([H])([H])[H])C([H])(C([H])([H])[H])C([H])([H])[H])([P4+](C([H])(C([H])([H])[H])C([H])([H])[H])(C([H])(C([H])([H])[H])C([H])([H])[H])C([H])(C([H])([H])[H])C([H])([H])[H])[N1]2[H]	0.27553
850911	O=[P@@]1([O][U+2])[O][U+3][O][P@@](=O)([O][U+2])[O][U@OH23+7]([O-])([O-])([O-])([O-])[O]1	O=P1([O2][U+2])[O4][U+3][O3]P(=O)([O1][U+2])[O6][U+7]([O7-])([O8-])([O9-])([O10-])[O5]1	1.131383
850947	[H]C1=C([H])C([H])=C([Te][Cu+5]([P+](C([H])([H])C([H])([H])[H])(C([H])([H])C([H])([H])[H])C([H])([H])C([H])([H])[H])([P+](C([H])([H])C([H])([H])[H])(C([H])([H])C([H])([H])[H])C([H])([H])C([H])([H])[H])[P+](C([H])([H])C([H])([H])[H])(C([H])([H])C([H])([H])[H])C([H])([H])C([H])([H])[H])C([H])=C1[H]	[H]C1=C([H])C([H])=[C4]([Te][Cu+5]([P1+](C([H])([H])C([H])([H])[H])(C([H])([H])C([H])([H])[H])C([H])([H])C([H])([H])[H])([P2+](C([H])([H])C([H])([H])[H])(C([H])([H])C([H])([H])[H])C([H])([H])C([H])([H])[H])[P3+](C([H])([H])C([H])([H])[H])(C([H])([H])C([H])([H])[H])C([H])([H])C([H])([H])[H])C([H])=C1[H]	0.498314
851092	[H]C1([H])[O+]2C([H])([H])C([H])([H])[N+]34C([H])([H])C([H])([H])[O+]5C([H])([H])C([H])([H])[O+]6C([H])([H])C([H])([H])[N+]78C([H])([H])C([H])([H])[O+](C1([H])[H])[K-7]256371[O+](C([H])([H])C([H])([H])[O+]1C([H])([H])C8([H])[H])C([H])([H])C4([H])[H]	[H]C1([H])C([H])([H])[O3+]2C([H])([H])C([H])([H])[O8+]3C([H])([H])C([H])([H])[N2+]45C([H])([H])C([H])([H])[O6+]6C([H])([H])C([H])([H])[O4+]7C([H])([H])C([H])([H])[N1+]18C([H])([H])C([H])([H])[O5+]1C([H])([H])C([H])([H])[O7+](C([H])([H])C4([H])[H])[K-7]8527163	0.242127

Sheet1

876252	[H]C1=C([H])C([H])=C(C2=C(OC([H])([H])[H])O[Si+2]345(OC(OC([H])([H])[H])=C(C6=C([H])C([H])=C([H])C([H])=C6[H])[N+]3=C([H])C3=C(O4)C([H])=C([H])C([H])=C3[H])OC3=C(C([H])=C([H])C([H])=C3[H])C([H])=[N+]25)C([H])=C1[H]	[H]C1=C([H])C([H])=C(C2=C(OC([H])([H])[H])[O4][Si+2]345([N1+]2=C([H])C2=C(C([H])=C([H])C([H])=C2[H])[O5]3)[N2+](=C([H])C2=C(C([H])=C([H])C([H])=C2[H])[O6]4)C(C2=C([H])C([H])=C([H])C([H])=C2[H])=C(OC([H])([H])[H])[O3]5)C([H])=C1[H]	0.237206
902738	[H]C([H])(C(=O)[O-])[N+]12C([H])([H])C(=O)[O][Mo@OH16+10]1(=[O])(=[O])(=[O])[O]C(=O)C2([H])[H]	[H]C([H])(C(=O)[O-])[N1+]12C([H])([H])C(=O)[O5][Mo+10]1(=[O2])(=[O3])(=[O4])[O6]C(=O)C2([H])[H]	0.332113
942961	[H]C1=C([H])C(C2=NC3=C(C4=C5C6=C3C([H])=C([H])C([H])=[N+]6[Sr-6]3([O-])([O-])([O-])([O-])([N+]6=C([H])C([H])=C([H])C7=C6C6=C(C([H])=C([H])C([H])=[N+]63)C3=C7N=C(C6=C([H])C([H])=C(C7[O][Sr][O]7)C([H])=C6[H])N3[H])[N+]5=C([H])C([H])=C4[H])N2[H])=C([H])C([H])=C1C1[O][Sr][O]1	[H]C1=C([H])C(C2=NC3=C(C4=C5C6=C3C([H])=C([H])C([H])=[N5+]6[Sr-6]3([O9-])([O10-])([O11-])([O12-])([N6+]6=C([H])C([H])=C([H])C7=C6C6=C(C([H])=C([H])C([H])=[N8+]63)C3=C7N=C(C6=C([H])C([H])=C(C7[O3][Sr][O4]7)C([H])=C6[H])N3[H])[N7+]5=C([H])C([H])=C4[H])N2[H])=C([H])C([H])=C1C1[O1][Sr][O2]1	0.236912
993277	[H]C1=C([H])C2=C(C3=C([H])C([H])=C([S+](=O)(~[O-][Tb+2])~[O-][Tb+2])C([H])=C3[H])C3=C([H])C([H])=C([N-]3)C(C3=C([H])C([H])=C([S+](=O)(~[O-][Tb+2])~[O-][Tb+9]([O-])([O-])([O-])([O-])([O-])([O-])[O-])C([H])=C3[H])=C3N=C(C([H])=C3[H])C(C3=C([H])C([H])=C([S+](=O)(~[O-][Tb+2])~[O-][Tb+2])C([H])=C3[H])=C3[N-]C(=C(C4=C([H])C([H])=C([S+](=O)(~[O-][Tb+2])~[O-][Tb+2])C([H])=C4[H])C1=N2)C([H])=C3[H]	[H]C1=C([H])C2=C(C3=C([H])C([H])=C([S+](=O)(~[O3-][Tb+2])~[O4-][Tb+2])C([H])=C3[H])C3=C([H])C([H])=C([N-]3)C(C3=C([H])C([H])=C([S+](=O)(~[O7-][Tb+2])~[O8-][Tb+9]([O9-])([O10-])([O11-])([O12-])([O13-])([O14-])[O15-])C([H])=C3[H])=C3N=C(C([H])=C3[H])C(C3=C([H])C([H])=C([S+](=O)(~[O2-][Tb+2])~[O6-][Tb+2])C([H])=C3[H])=C3[N-]C(=C(C4=C([H])C([H])=C([S+](=O)(~[O1-][Tb+2])~[O5-][Tb+2])C([H])=C4[H])C1=N2)C([H])=C3[H]	1.441013

Sheet1

OrbGlobalRMSD_A	HybridGlobalRMSD_A	OrbMetalSiteRMSD_A	HybridMetalSiteRMSD_A	OptimizedTrustRMSD_A
0.206949	0.409687	0.114345	0.142645	0.2184065
0.271902	0.216644	0.056202	0.066749	0.15287425
0.50192	0.516271	0.417945	0.417201	0.46333425
0.110136	0.253447	0.080256	0.110347	0.1385465
0.488638	0.450565	0.016858	0.028809	0.2462175
0.334208	0.099716	0.008253	0.016917	0.1147735
0.98317	1.008366	1.099721	1.13902	1.05756925
0.66273	0.800492	0.433525	0.239406	0.53403825
0.354938	0.376753	0.08143	0.09246	0.22639525
0.460248	0.39809	0.01492	0.055889	0.23228675
0.116288	0.557241	0.042725	0.048947	0.19130025
0.210106	0.176128	0.146368	0.138366	0.167742
0.550371	0.647002	0.021456	0.026062	0.31122275
0.397309	0.180287	0.293577	0.150681	0.2554635
0.919494	0.928946	N/A	N/A	N/A
0.321722	0.364508	0.113731	0.136251	0.234053
0.264769	0.504725	0.137849	0.116872	0.25605374999999997
0.71909	0.713992	0.022179	0.019966	0.36880675
0.857992	1.097697	0.027999	0.017087	0.50019375
0.82145	0.83304	0.258904	0.279675	0.54826725
0.58807	0.223269	0.0558	0.076568	0.23592675

Sheet1

0.840306	1.15228	0.393646	0.349674	0.6839765
0.898779	0.905764	0.066082	0.065491	0.484029
0.201781	0.324295	0.021019	0.035498	0.14564825
0.890965	0.973123	0.124148	0.13416	0.530599
0.110541	0.600744	N/A	N/A	N/A
0.551156	0.263987	0.153054	0.148503	0.279175
0.435738	0.303745	0.100546	0.126702	0.24168275
0.131449	0.177368	0.059623	0.096248	0.116172
0.143691	0.134476	0.077557	0.074595	0.10757975
0.046071	0.063398	N/A	N/A	N/A
0.575457	0.133219	0.023566	0.02345	0.188923
0.424706	0.114889	0.298091	0.048041	0.22143174999999998
0.391746	0.61747	0.062561	0.109907	0.295421
1.198259	0.77861	0.595934	0.442095	0.7537245
1.469257	0.538792	0.032102	0.069257	0.527352
0.13496	0.106773	N/A	N/A	N/A
1.330029	1.312791	N/A	N/A	N/A
0.372465	0.086838	N/A	N/A	N/A
0.370889	0.086817	N/A	N/A	N/A
0.774905	0.281112	0.119406	0.096854	0.31806924999999997
0.487136	0.486326	0.414305	0.413082	0.45021225
0.61504	0.452351	0.103953	0.153276	0.33115500000000003

Sheet1

0.421118	0.285782	0.057639	0.065518	0.20751424999999998
0.222267	0.307875	0.066906	0.089521	0.17164225
0.755295	0.401177	0.193791	0.191994	0.38556425
0.219963	0.333134	0.15407	0.141228	0.21209875
0.072338	0.135103	0.054276	0.091022	0.08818475
0.08223	0.19891	N/A	N/A	N/A
1.003311	0.494343	0.067413	0.05014	0.40380175
0.065774	0.054017	0.047925	0.029011	0.04918175
0.2823	0.482076	0.082182	0.456195	0.32568825
0.13012	0.114449	0.013037	0.018702	0.069077
0.227377	0.460571	0.255731	0.386047	0.3324315
0.130941	0.098983	0.022343	0.03144	0.07092675
0.087179	0.06204	0.047293	0.04634	0.060713
0.828676	0.80772	0.015691	0.013375	0.4163655
1.035893	0.273999	0.031533	0.022669	0.3410235
0.713249	0.590148	0.614939	0.635163	0.63837475
0.51787	1.007088	0.316537	0.371341	0.5532090000000001
0.169526	0.240927	N/A	N/A	N/A
0.965201	1.220397	0.501183	0.629853	0.8291585
0.146936	0.428502	0.00575	0.016222	0.1493525
0.245716	0.377573	0.065542	0.113076	0.20047674999999998
0.313332	0.836764	0.068542	0.103429	0.33051674999999997

Sheet1

1.039852	0.386059	0.035994	0.035977	0.3744705
0.113572	0.117375	0.068374	0.08279	0.09552775
1.316818	1.596181	1.429434	1.969738	1.57804275
0.473334	0.611166	0.037453	0.037312	0.28981625
0.260051	0.298683	0.110548	0.11474	0.1960055
0.289743	0.289349	0.289743	0.289349	0.28954599999999997
0.15219	0.193774	0.073433	0.060158	0.11988875
0.287751	0.54009	0.063359	0.11624	0.25186
0.350377	0.378574	0.022333	0.030536	0.195455
0.801294	0.855547	0.389065	0.539743	0.64641225
1.516642	1.536295	1.41807	1.438666	1.47741825
0.443561	0.420125	0.068345	0.069879	0.2504775
1.174933	1.128186	0.711848	0.635712	0.9126697500000001
0.441787	0.624568	N/A	N/A	N/A
0.537779	0.553893	0.109101	0.11719	0.32949075
1.788019	3.742331	2.226525	4.837785	3.1486650000000003
1.260037	1.773973	1.982361	3.13192	2.03707275

Sheet1

MetalSiteAtomCount	Status
9	OK
7	OK
12	OK
7	OK
4	OK
7	OK
18	OK
10	OK
5	OK
7	OK
5	OK
7	OK
7	OK
14	OK
N/A	OK_NO_METAL
13	OK
4	OK
7	OK
6	OK
9	OK
9	OK

Sheet1

9	OK
5	OK
6	OK
17	OK
N/A	OK_NO_METAL
10	OK
7	OK
8	OK
5	OK
N/A	OK_NO_METAL
5	OK
8	OK
11	OK
14	OK
5	OK
N/A	OK_NO_METAL
N/A	OK_NO_METAL
N/A	OK_NO_METAL
N/A	OK_NO_METAL
13	OK
14	OK
8	OK

Sheet1

9	OK
6	OK
13	OK
9	OK
7	OK
N/A	OK_NO_METAL
5	OK
5	OK
6	OK
11	OK
10	OK
5	OK
7	OK
5	OK
5	OK
5	OK
3	OK
N/A	OK_NO_METAL
10	OK
5	OK
16	OK
7	OK

Sheet1

6	OK
7	OK
17	OK
7	OK
7	OK
16	OK
9	OK
8	OK
9	OK
7	OK
14	OK
6	OK
9	OK
N/A	OK_NO_METAL
7	OK
15	OK
23	OK
