# Supplementary material for: RapidMetalFF-AI: An AI-Assisted Web Server for Minute-Scale Bonded Force-Field Parameterization and Structural Validation of Metal-Containing Molecules with Validated AMBER and Converted GROMACS/CHARMM Outputs
Source: Molecules. 2026 Oct 17;31(20):3724. doi: 10.3390/molecules31203724 (PMC13707471; doi:10.3390/molecules31203724)
Supplement: Supplementary file 1 [file molecules-31-03724-s001.zip › pdf_versions_of_data/Supplementary_Table_S4_Exact82_Molecule_Resolved_Runtime.pdf]

Sheet1

Molecule	runtime_seconds	queue_seconds	mol2_preflight_s	orb_opt_s	structure_integrity_post_orbmol_s
145192	80.187329	0.0	0.141948	54.87542724609375	0.3351399898529053
152002	45.758577	0.0	0.094113	20.32119059562683	0.24333524703979492
154031	27.183664	0.0	0.118515	11.978055715560913	0.21673583984375
154784	47.060131	0.0	0.118507	30.048702239990234	0.19508814811706543
155447	41.011334	0.0	0.085482	21.49534559249878	0.1944580078125
156313	38.690866	0.0	0.112962	19.032079219818115	0.22761011123657227
158148	87.025174	0.0	0.090454	49.19846057891846	0.18185019493103027
162486	43.40095	0.0	0.111913	20.63136863708496	0.16690468788146973
163152	33.013751	0.0	0.073601	15.653989791870117	0.1550917625427246
175478	29.283958	0.0	0.115103	12.757044553756714	0.20558929443359375
179921	46.817214	0.0	0.071107	27.514269590377808	0.16650056838989258
182741	44.788092	0.0	0.113022	24.016706466674805	0.1783754825592041
183305	45.848816	0.0	0.110302	25.61381483078003	0.16927504539489746
229783	36.959576	0.0	0.075115	16.64805293083191	0.15192675590515137
232528	27.774824	0.0	0.109963	14.03566575050354	0.15988707542419434
232536	42.033382	0.0	0.103677	20.566413402557373	0.18383479118347168
232591	31.997509	0.0	0.073494	11.894618034362793	0.15593743324279785
234033	35.65498	0.0	0.111353	19.0119411945343	0.15972304344177246
234071	38.288637	0.0	0.104989	16.85975742340088	0.1638791561126709
236914	31.954319	0.0	0.104222	15.862126111984253	0.18387866020202637
243849	58.703925	0.0	0.107272	30.205947637557983	0.1870408058166504

Sheet1

244175	55.901747	0.0	0.071398	29.958688735961914	0.18698787689208984
251255	51.651606	0.0	0.070211	25.879273414611816	0.1500532627105713
253279	234.284824	0.0	0.083729	105.66485524177551	0.21259498596191406
253288	55.942962	0.0	0.069039	23.535308361053467	0.16696858406066895
253291	203.1473	0.0	0.140431	72.61998963356018	0.25133490562438965
602743	55.078549	0.0	0.108678	27.19123673439026	0.19975662231445312
613741	42.909307	0.0	0.102867	20.299962043762207	0.1911458969116211
716039	90.696261	0.0	0.094602	44.497995138168335	0.20581984519958496
780531	80.360894	0.0	0.163157	45.42626476287842	0.2082068920135498
791724	51.498296	0.0	0.258608	23.296329736709595	0.19416522979736328
801553	56.742296	0.0	0.101584	27.35238742828369	0.19239234924316406
804893	70.019908	0.0	0.105599	34.81090235710144	0.2058868408203125
804894	84.918277	0.0	0.134876	47.4442253112793	0.1735985279083252
804896	98.635013	0.0	0.124846	43.01482844352722	0.31320667266845703
849384	120.751505	0.0	0.244293	36.75646471977234	0.34938716888427734
850911	216.720509	0.0	0.105815	23.86200714111328	0.16650795936584473
850947	101.406242	0.0	0.157857	52.69049048423767	0.194291353225708
851092	58.078523	0.0	0.101816	26.47098398208618	0.18849611282348633
876252	68.013216	0.0	0.117804	30.624968767166138	0.22876453399658203
902738	69.978231	0.0	0.143087	48.68304252624512	0.21815276145935059
942961	77.79706	0.0	0.103062	30.93456268310547	0.20739245414733887
993277	170.900842	0.0	0.2378	118.76608419418335	0.1916825771331787

Sheet1

1012591	103.954096	0.0	0.094917	44.342621088027954	0.2771003246307373
1033814	83.833783	0.0	0.183228	33.30150365829468	0.3200409412384033
1100009	61.66779	0.0	0.266028	31.770158767700195	0.27466273307800293
1100100	51.608867	0.0	0.128817	24.99704647064209	0.23645973205566406
1100131	35.58575	0.0	0.109197	15.592461824417114	0.20513105392456055
1100134	37.352633	0.0	0.068577	19.86297583580017	0.206695556640625
1100181	91.046592	0.0	0.112874	41.132728815078735	0.18185663223266602
1100271	60.919511	0.0	0.081008	21.994730234146118	0.19833683967590332
1100319	31.457777	0.0	0.062855	14.20275354385376	0.15333342552185059
1100364	39.637207	0.0	0.068548	18.26955485343933	0.17519736289978027
1100416	45.228263	0.0	0.087183	26.975515604019165	0.17519068717956543
1101146	28.609688	0.0	0.115571	16.146807432174683	0.18197107315063477
1101247	29.578532	0.0	0.107586	12.578008890151978	0.14031744003295898
1493407	38.612514	0.0	0.069644	17.647602796554565	0.17118406295776367
1506292	77.3718	0.0	0.109065	22.57295823097229	0.17737483978271484
1518810	67.28512	0.0	0.108431	44.9677574634552	0.16144156455993652
1519012	38.136531	0.0	0.109327	20.71192216873169	0.13115286827087402
1530857	53.720163	0.0	0.069899	23.482402086257935	0.16150784492492676
1563993	266.071695	0.0	0.091254	53.760791540145874	0.18854188919067383
1574551	39.691187	0.0	0.063245	20.440504550933838	0.1569070816040039
1582689	39.124409	0.0	0.115644	19.672839879989624	0.15397381782531738
2051215	103.079177	0.0	0.069915	50.927796840667725	0.42978954315185547

Sheet1

2074417	80.916117	0.0	0.066262	34.360806465148926	0.17010927200317383
2075115	34.318371	0.0	0.064816	13.337920904159546	0.14245319366455078
2075193_as	40.177589	0.0	0.074652	15.791971683502197	0.1542201042175293
2076769	30.029766	0.0	0.109266	11.99721622467041	0.14391756057739258
2076771	31.002043	0.0	0.128561	12.579710721969604	0.13828730583190918
2083565	39.634249	0.0	0.121193	22.269521951675415	0.18895602226257324
2083570	39.809549	0.0	0.110441	20.86074185371399	0.14596080780029297
2087213	159.688864	0.0	0.067674	52.29557490348816	0.23610234260559082
2087214	74.442852	0.0	0.116781	30.813339948654175	0.18667030334472656
2087215	82.282309	0.0	0.087562	38.85946273803711	0.2080376148223877
2091598	50.786464	0.0	0.125498	14.459749937057495	0.14606261253356934
2096425	45.510224	0.0	0.060709	21.258536100387573	0.21703648567199707
2104359	42.034647	0.0	0.069567	13.486050128936768	0.16093683242797852
2150100	32.320536	0.0	0.144529	16.524497985839844	0.2220611572265625
2378332	35.747552	0.0	0.066196	16.019132137298584	0.13085103034973145
2515638	31.092048	0.0	0.070448	12.497260570526123	0.17812466621398926
2535798	69.524773	0.0	0.090008	26.36311960220337	0.19667363166809082

Sheet1

orb_fd_hessian_s	metal_ff_s	xtb_charge_s	pipeline_ff_openmm_s	export_ff_s	sum_listed_stage_times_s
8.946659803390503	0.30246	0.671128	3.361406	0.56567	69.19983903933716
10.838517427444458	0.314848	0.75574	2.697283	0.548266	35.81329327011108
3.535287857055664	0.317838	0.639707	2.389358	0.405096	19.600593412460327
5.098119497299194	0.244376	0.974589	2.141423	0.488153	39.30895788540649
6.653665542602539	0.244983	0.675002	2.514208	0.536533	32.399677142913816
8.085772037506104	0.262997	0.432377	2.174756	0.475149	30.803702368560792
25.536100387573242	0.266012	0.767834	2.483009	0.561288	79.08500816142273
11.396720886230469	0.265726	0.854543	1.963983	0.320309	35.7114682111969
6.235177040100098	0.229092	0.421269	2.072697	0.509629	25.350546594512938
4.5728724002838135	0.256803	0.464995	1.999852	0.534176	20.90643524847412
7.819249153137207	0.243442	0.471927	2.094659	0.516605	38.897759311904906
9.806386232376099	0.367959	0.587365	2.14823	0.529254	37.747298181610105
8.19708800315857	0.251245	0.613287	2.896077	0.483755	38.3348438793335
8.588089227676392	0.27417	0.440199	2.318437	0.598403	29.09439291441345
2.634183883666992	0.227988	0.440051	2.324619	0.484352	20.416709709594727
9.728827953338623	0.263961	0.535545	2.427498	0.481445	34.29120214707947
7.652186870574951	0.345716	0.547387	2.841089	0.52855	24.03897833818054
4.009293556213379	0.269322	0.707346	2.47244	0.48534	27.226758794189454
9.655545473098755	0.269758	0.812332	2.364356	0.489051	30.719668052612306
4.395708084106445	0.254711	0.644241	2.63381	0.47717	24.555866856292724
13.889711141586304	0.539968	1.880292	3.155013	0.475512	50.44075658496094

Sheet1

13.684912204742432	0.278821	0.692599	2.725387	0.523652	48.122445817596436
12.703514814376831	0.248718	1.719891	2.831416	0.528105	44.13118249169922
108.2559142112732	0.311094	7.70579	4.003142	0.512006	226.7491254390106
16.67290186882019	0.421173	1.018989	3.738524	1.902781	47.525684813934326
107.40153169631958	0.461031	7.487604	5.41232	0.566272	194.34051423550414
11.310224771499634	0.347712	1.632097	3.865822	0.873611	45.529138128204345
6.538839340209961	0.367021	0.660723	3.374951	0.740222	32.27573128088379
27.9074547290802	0.409232	1.656057	4.58329	0.849607	80.20405771244812
14.129272699356079	0.357444	1.610029	3.848188	1.170856	66.91341835424805
10.700595378875732	0.381658	1.036198	3.48002	1.180731	40.52830534538269
13.219677686691284	0.400557	0.798516	3.942051	1.080533	47.08769846421814
13.3980872631073	0.415833	3.738812	6.013819	1.320224	60.009163461029054
19.432164907455444	0.417301	2.133968	3.802458	0.566271	74.10486274664306
9.875064373016357	0.400347	2.329879	32.728556	0.468853	89.25558048921204
64.60686993598938	0.411246	1.067925	4.680665	1.143707	109.260557824646
3.779292345046997	0.389524	172.861659	5.218569	0.522713	206.90608744552614
27.663307428359985	0.492866	2.477511	5.139392	1.309978	90.12569326582336
15.516210079193115	0.406518	0.976914	3.423206	1.150525	48.234669174102784
17.43259859085083	0.407551	3.494239	5.333544	0.939691	58.57916089201355
4.79185938835144	0.347273	0.825778	3.707696	1.056679	59.773567676055904
21.31043314933777	0.551389	6.90953	5.886618	0.690488	66.59347528659058
27.627832174301147	0.38541	7.111068	5.303671	0.497177	160.12072494561767

Sheet1

35.798166036605835	0.518902	4.92216	6.441056	0.719377	93.11429944926452
25.348991870880127	0.485418	2.40386	5.731533	1.432944	69.2075194704132
6.431509971618652	0.592369	1.016301	6.156089	1.326909	47.83402747239685
12.107835292816162	0.241173	1.031881	3.293431	0.476973	42.513616495513915
6.201700925827026	0.22651	0.643569	2.519134	0.49102	25.9887238041687
2.4781858921051025	0.265833	3.299758	2.941353	0.573517	29.6968952845459
37.41764450073242	0.311211	1.428616	2.85887	0.533304	83.97710494804382
26.551511526107788	0.275165	1.104923	3.129345	0.547876	53.88289559992981
6.087527513504028	0.271293	0.512594	2.586429	0.526901	24.40368648287964
5.022277355194092	0.249032	0.580667	4.842418	0.522457	29.730151571533202
6.219431161880493	0.229923	0.519267	2.904703	0.545835	37.65704845307923
1.4904470443725586	0.266673	0.612976	2.280978	0.438834	21.534257549697877
5.705265998840332	0.330568	0.61936	2.749191	0.524484	22.754781329025267
9.845040559768677	0.27852	0.440281	2.498358	0.510399	31.461029419281004
43.55674457550049	0.255368	0.659376	2.759995	0.493599	70.5844806462555
11.314681768417358	0.257543	0.729547	2.384927	0.554546	60.47887479643249
6.8558290004730225	0.296354	0.507983	2.229649	0.519079	31.361296037475586
18.923080444335938	0.298022	0.687235	2.508516	0.524217	46.654879375518796
42.66123986244202	0.247554	158.13984	3.797012	0.429885	259.31611829177854
7.738306999206543	0.261031	0.754423	2.552752	0.466951	32.434120631744385
8.025215148925781	0.244963	0.535397	2.556514	0.490878	31.795424846740723
36.1527304649353	3.145769	1.659095	2.537661	0.513929	95.43668584875488

Sheet1

32.64429426193237	0.257876	2.040102	2.836813	0.478407	72.85466999908448
6.491785526275635	0.256802	0.500292	5.495802	0.469439	26.759310624099733
12.592246532440186	0.254989	0.528268	2.983794	0.538815	32.918956320159914
6.632784605026245	0.292799	0.697502	2.43442	0.511095	22.81900039027405
6.662773132324219	0.28207	0.675942	2.552897	0.514838	23.535079160125733
5.627129316329956	0.259971	0.670993	2.744086	0.515848	32.39769829026795
6.661670684814453	0.263943	0.931141	2.765857	0.460428	32.200183346328735
93.29614281654358	0.300649	1.236363	2.981476	0.631741	151.04572306263734
30.61601710319519	0.283185	1.816684	2.597677	0.526457	66.95681135519409
31.990626335144043	0.270075	0.591497	2.513098	0.544692	75.06505068800354
20.879725694656372	0.249564	1.262641	2.635675	0.419725	40.17864124424744
11.789644241333008	0.272358	1.043916	2.826632	0.523967	37.992798827392576
9.907672643661499	0.318239	1.319151	5.776522	0.474039	31.512177605026245
3.6342861652374268	0.350496	0.70612	2.706151	0.493318	24.781459308303834
6.9942638874053955	0.238044	0.594197	3.105429	0.563887	27.71200005505371
4.264362812042236	2.284043	0.466763	2.663357	0.513427	22.93778604878235
27.831724166870117	0.26477	0.705417	2.978425	0.567689	58.99782640074158

Sheet1

runtime_minus_listed_stages_s	orb_stage_runtime_s	gpu_name
10.987489960662842	72.42884659767151	NVIDIA GeForce RTX 4060 Laptop GPU
9.94528372988892	39.18366765975952	NVIDIA GeForce RTX 4060 Laptop GPU
7.583070587539673	21.1428325176239	NVIDIA GeForce RTX 4060 Laptop GPU
7.751173114593506	40.987449407577515	NVIDIA GeForce RTX 4060 Laptop GPU
8.611656857086182	34.51844143867493	NVIDIA GeForce RTX 4060 Laptop GPU
7.887163631439208	32.86143445968628	NVIDIA GeForce RTX 4060 Laptop GPU
7.940165838577272	80.5370728969574	NVIDIA GeForce RTX 4060 Laptop GPU
7.6894817888031	37.925280809402466	NVIDIA GeForce RTX 4060 Laptop GPU
7.663204405487061	27.58781123161316	NVIDIA GeForce RTX 4060 Laptop GPU
8.377522751525877	23.803311824798584	NVIDIA GeForce RTX 4060 Laptop GPU
7.919454688095094	41.22845220565796	NVIDIA GeForce RTX 4060 Laptop GPU
7.0407938183898935	38.89366006851196	NVIDIA GeForce RTX 4060 Laptop GPU
7.5139721206665016	39.413026094436646	NVIDIA GeForce RTX 4060 Laptop GPU
7.865183085586548	31.133440494537354	NVIDIA GeForce RTX 4060 Laptop GPU
7.358114290405272	22.100952863693237	NVIDIA GeForce RTX 4060 Laptop GPU
7.742179852920536	36.31286883354187	NVIDIA GeForce RTX 4060 Laptop GPU
7.958530661819459	25.613747119903564	NVIDIA GeForce RTX 4060 Laptop GPU
8.428221205810548	28.61974334716797	NVIDIA GeForce RTX 4060 Laptop GPU
7.568968947387695	32.09525966644287	NVIDIA GeForce RTX 4060 Laptop GPU
7.398452143707278	25.89375329017639	NVIDIA GeForce RTX 4060 Laptop GPU
8.263168415039061	50.56275677680969	NVIDIA GeForce RTX 4060 Laptop GPU

Sheet1

7.7793011824035645	49.42604637145996	NVIDIA GeForce RTX 4060 Laptop GPU
7.52042350830078	44.070130348205566	NVIDIA GeForce RTX 4060 Laptop GPU
7.535698560989374	219.41643357276917	NVIDIA GeForce RTX 4060 Laptop GPU
8.417277186065675	46.128262519836426	NVIDIA GeForce RTX 4060 Laptop GPU
8.806785764495856	186.6340320110321	NVIDIA GeForce RTX 4060 Laptop GPU
9.549410871795658	45.526360750198364	NVIDIA GeForce RTX 4060 Laptop GPU
10.633575719116209	34.803982734680176	NVIDIA GeForce RTX 4060 Laptop GPU
10.492203287551888	79.47575187683105	NVIDIA GeForce RTX 4060 Laptop GPU
13.44747564575195	69.37326502799988	NVIDIA GeForce RTX 4060 Laptop GPU
10.969990654617312	42.094923973083496	NVIDIA GeForce RTX 4060 Laptop GPU
9.65459753578186	47.60223126411438	NVIDIA GeForce RTX 4060 Laptop GPU
10.010744538970947	55.33755826950073	NVIDIA GeForce RTX 4060 Laptop GPU
10.813414253356939	75.4321231842041	NVIDIA GeForce RTX 4060 Laptop GPU
9.379432510787964	60.14632821083069	NVIDIA GeForce RTX 4060 Laptop GPU
11.490947175353995	110.34332752227783	NVIDIA GeForce RTX 4060 Laptop GPU
9.814421554473853	35.080779790878296	NVIDIA GeForce RTX 4060 Laptop GPU
11.280548734176648	87.99819111824036	NVIDIA GeForce RTX 4060 Laptop GPU
9.843853825897213	48.961328983306885	NVIDIA GeForce RTX 4060 Laptop GPU
9.434055107986453	55.03130292892456	NVIDIA GeForce RTX 4060 Laptop GPU
10.20466332394409	60.96354556083679	NVIDIA GeForce RTX 4060 Laptop GPU
11.203584713409427	61.00980019569397	NVIDIA GeForce RTX 4060 Laptop GPU
10.780117054382345	154.93593883514404	NVIDIA GeForce RTX 4060 Laptop GPU

Sheet1

10.839796550735485	87.17419481277466	NVIDIA GeForce RTX 4060 Laptop GPU
14.626263529586794	68.96172428131104	NVIDIA GeForce RTX 4060 Laptop GPU
13.833762527603149	48.16486191749573	NVIDIA GeForce RTX 4060 Laptop GPU
9.095250504486081	44.43659448623657	NVIDIA GeForce RTX 4060 Laptop GPU
9.597026195831297	29.713731288909912	NVIDIA GeForce RTX 4060 Laptop GPU
7.655737715454098	28.025158882141113	NVIDIA GeForce RTX 4060 Laptop GPU
7.069487051956187	83.81965684890747	NVIDIA GeForce RTX 4060 Laptop GPU
7.03661540007019	53.73088026046753	NVIDIA GeForce RTX 4060 Laptop GPU
7.054090517120361	25.37844443321228	NVIDIA GeForce RTX 4060 Laptop GPU
9.907055428466794	31.35646152496338	NVIDIA GeForce RTX 4060 Laptop GPU
7.571214546920771	38.96488356590271	NVIDIA GeForce RTX 4060 Laptop GPU
7.075430450302122	22.993196725845337	NVIDIA GeForce RTX 4060 Laptop GPU
6.823750670974732	23.25272846221924	NVIDIA GeForce RTX 4060 Laptop GPU
7.151484580718993	32.836891174316406	NVIDIA GeForce RTX 4060 Laptop GPU
6.787319353744493	71.14562368392944	NVIDIA GeForce RTX 4060 Laptop GPU
6.806245203567514	61.329081535339355	NVIDIA GeForce RTX 4060 Laptop GPU
6.7752349625244115	32.555705547332764	NVIDIA GeForce RTX 4060 Laptop GPU
7.065283624481204	47.59873390197754	NVIDIA GeForce RTX 4060 Laptop GPU
6.75557670822144	101.52229619026184	NVIDIA GeForce RTX 4060 Laptop GPU
7.2570663682556145	33.65750813484192	NVIDIA GeForce RTX 4060 Laptop GPU
7.328984153259277	33.25715708732605	NVIDIA GeForce RTX 4060 Laptop GPU
7.642491151245125	93.09295010566711	NVIDIA GeForce RTX 4060 Laptop GPU

Sheet1

8.061447000915521	73.26566481590271	NVIDIA GeForce RTX 4060 Laptop GPU
7.559060375900266	25.58116888999939	NVIDIA GeForce RTX 4060 Laptop GPU
7.258632679840083	33.7640540599823	NVIDIA GeForce RTX 4060 Laptop GPU
7.21076560972595	24.061741590499878	NVIDIA GeForce RTX 4060 Laptop GPU
7.466963839874268	24.65128803253174	NVIDIA GeForce RTX 4060 Laptop GPU
7.23655070973205	33.42452120780945	NVIDIA GeForce RTX 4060 Laptop GPU
7.6093656536712615	33.399513244628906	NVIDIA GeForce RTX 4060 Laptop GPU
8.64314093736266	152.48956894874573	NVIDIA GeForce RTX 4060 Laptop GPU
7.486040644805911	66.96274304389954	NVIDIA GeForce RTX 4060 Laptop GPU
7.21725831199646	76.38408422470093	NVIDIA GeForce RTX 4060 Laptop GPU
10.607822755752565	44.24989461898804	NVIDIA GeForce RTX 4060 Laptop GPU
7.517425172607425	38.89458203315735	NVIDIA GeForce RTX 4060 Laptop GPU
10.522469394973754	32.160276889801025	NVIDIA GeForce RTX 4060 Laptop GPU
7.539076691696163	25.924078226089478	NVIDIA GeForce RTX 4060 Laptop GPU
8.035551944946288	29.144618034362793	NVIDIA GeForce RTX 4060 Laptop GPU
8.154261951217649	23.08762288093567	NVIDIA GeForce RTX 4060 Laptop GPU
10.526946599258416	62.81657266616821	NVIDIA GeForce RTX 4060 Laptop GPU

Sheet1

metadata_file
145192/rapidmetalff_metadata.json
152002/rapidmetalff_metadata.json
154031/rapidmetalff_metadata.json
154784/rapidmetalff_metadata.json
155447/rapidmetalff_metadata.json
156313/rapidmetalff_metadata.json
158148/rapidmetalff_metadata.json
162486/rapidmetalff_metadata.json
163152/rapidmetalff_metadata.json
175478/rapidmetalff_metadata.json
179921/rapidmetalff_metadata.json
182741/rapidmetalff_metadata.json
183305/rapidmetalff_metadata.json
229783/rapidmetalff_metadata.json
232528/rapidmetalff_metadata.json
232536/rapidmetalff_metadata.json
232591/rapidmetalff_metadata.json
234033/rapidmetalff_metadata.json
234071/rapidmetalff_metadata.json
236914/rapidmetalff_metadata.json
243849/rapidmetalff_metadata.json

Sheet1

244175/rapidmetalff_metadata.json
251255/rapidmetalff_metadata.json
253279/rapidmetalff_metadata.json
253288/rapidmetalff_metadata.json
253291/rapidmetalff_metadata.json
602743/rapidmetalff_metadata.json
613741/rapidmetalff_metadata.json
716039/rapidmetalff_metadata.json
780531/rapidmetalff_metadata.json
791724/rapidmetalff_metadata.json
801553/rapidmetalff_metadata.json
804893/rapidmetalff_metadata.json
804894/rapidmetalff_metadata.json
804896/rapidmetalff_metadata.json
849384/rapidmetalff_metadata.json
850911/rapidmetalff_metadata.json
850947/rapidmetalff_metadata.json
851092/rapidmetalff_metadata.json
876252/rapidmetalff_metadata.json
902738/rapidmetalff_metadata.json
942961/rapidmetalff_metadata.json
993277/rapidmetalff_metadata.json

Sheet1

1012591/rapidmetalff_metadata.json
1033814/rapidmetalff_metadata.json
1100009/rapidmetalff_metadata.json
1100100/rapidmetalff_metadata.json
1100131/rapidmetalff_metadata.json
1100134/rapidmetalff_metadata.json
1100181/rapidmetalff_metadata.json
1100271/rapidmetalff_metadata.json
1100319/rapidmetalff_metadata.json
1100364/rapidmetalff_metadata.json
1100416/rapidmetalff_metadata.json
1101146/rapidmetalff_metadata.json
1101247/rapidmetalff_metadata.json
1493407/rapidmetalff_metadata.json
1506292/rapidmetalff_metadata.json
1518810/rapidmetalff_metadata.json
1519012/rapidmetalff_metadata.json
1530857/rapidmetalff_metadata.json
1563993/rapidmetalff_metadata.json
1574551/rapidmetalff_metadata.json
1582689/rapidmetalff_metadata.json
2051215/rapidmetalff_metadata.json

Sheet1

2074417/rapidmetalff_metadata.json
2075115/rapidmetalff_metadata.json
2075193_as/rapidmetalff_metadata.json
2076769/rapidmetalff_metadata.json
2076771/rapidmetalff_metadata.json
2083565/rapidmetalff_metadata.json
2083570/rapidmetalff_metadata.json
2087213/rapidmetalff_metadata.json
2087214/rapidmetalff_metadata.json
2087215/rapidmetalff_metadata.json
2091598/rapidmetalff_metadata.json
2096425/rapidmetalff_metadata.json
2104359/rapidmetalff_metadata.json
2150100/rapidmetalff_metadata.json
2378332/rapidmetalff_metadata.json
2515638/rapidmetalff_metadata.json
2535798/rapidmetalff_metadata.json
