# Supplementary material for: RapidMetalFF-AI: An AI-Assisted Web Server for Minute-Scale Bonded Force-Field Parameterization and Structural Validation of Metal-Containing Molecules with Validated AMBER and Converted GROMACS/CHARMM Outputs
Source: Molecules. 2026 Oct 17;31(20):3724. doi: 10.3390/molecules31203724 (PMC13707471; doi:10.3390/molecules31203724)
Supplement: Supplementary file 1 [file molecules-31-03724-s001.zip › pdf_versions_of_data/Supplementary_Table_S5_Exact82_Runtime_Summary.pdf]

Sheet1

Metric	N	Mean_s	Median_s	SD_s	Q1_s	Q3_s	Min_s
End-to-end runtime	82	65.509286	48.923298	46.848647	38.369606	77.690745	27.183664
MOL2 preflight	82	0.107991	0.105707	0.041607	0.074768	0.116497	0.060709
OrbMol-v2 optimization	82	29.111316	23.508855	18.458367	17.056719	34.095981	11.894618
Post-OrbMol structural integrity	82	0.195164	0.186829	0.050833	0.162101	0.207218	0.130851
Orb finite-difference Hessian	82	17.386393	9.891369	20.394957	6.503549	20.517835	1.490447
RapidMetalFF parameter construction	82	0.376490	0.280446	0.387270	0.257626	0.378233	0.226510
GFN1-xTB / CM5 charge	82	5.400964	0.761787	25.544571	0.613054	1.626580	0.421269
FF assembly + OpenMM	82	3.650921	2.834115	3.450909	2.513375	3.801097	1.963983
Multi-format export	82	0.634148	0.524350	0.283719	0.489508	0.567335	0.320309
Sum of listed stages	82	56.863386	39.743800	46.539680	30.740677	66.945963	19.600593
Unlisted/orchestration runtime	82	8.645899	7.903309	1.719318	7.471733	9.836496	6.755577
Complete Orb stage	82	52.998365	41.107951	35.703652	32.111514	62.444700	21.142833

Sheet1

Max_s
266.071695
0.266028
118.766084
0.429790
108.255914
3.145769
172.861659
32.728556
1.902781
259.316118
14.626264
219.416434
