# Supplementary material for: RapidMetalFF-AI: An AI-Assisted Web Server for Minute-Scale Bonded Force-Field Parameterization and Structural Validation of Metal-Containing Molecules with Validated AMBER and Converted GROMACS/CHARMM Outputs
Source: Molecules. 2026 Oct 17;31(20):3724. doi: 10.3390/molecules31203724 (PMC13707471; doi:10.3390/molecules31203724)
Supplement: Supplementary file 1 [file molecules-31-03724-s001.zip › pdf_versions_of_data/Supplementary_Table_S7_Independent_QM_Geometry_Bond_Angle.pdf]

Sheet1

Molecule	Metal	Charge	Multiplicity	QMLevel	LowestRealFrequency_cm-1	ImaginaryFrequencyCount
1101146	Nb	0	1	HF/SDD	18.938	0
1101146	Nb	0	1	HF/SDD	18.938	0
1101146	Nb	0	1	HF/SDD	18.938	0
155447	Pt	0	1	HF/SDD	25.982	0
155447	Pt	0	1	HF/SDD	25.982	0
155447	Pt	0	1	HF/SDD	25.982	0
902738	Mo	-3	1	HF/SDD	33.632	0
902738	Mo	-3	1	HF/SDD	33.632	0
902738	Mo	-3	1	HF/SDD	33.632	0
1100364	Sc	0	1	HF/SDD	31.125	0
1100364	Sc	0	1	HF/SDD	31.125	0
1100364	Sc	0	1	HF/SDD	31.125	0
232528	Au	0	1	HF/SDD	25.185	0
232528	Au	0	1	HF/SDD	25.185	0
232528	Au	0	1	HF/SDD	25.185	0
613741	In	0	1	HF/SDD	37.913	0
613741	In	0	1	HF/SDD	37.913	0
613741	In	0	1	HF/SDD	37.913	0
163152	Ga	0	1	HF/SDD	25.548	0
163152	Ga	0	1	HF/SDD	25.548	0
163152	Ga	0	1	HF/SDD	25.548	0

Sheet1

234071	Bi	0	1	HF/SDD	38.968	0
234071	Bi	0	1	HF/SDD	38.968	0
234071	Bi	0	1	HF/SDD	38.968	0
179921	Sn	0	1	HF/SDD	16.979	0
179921	Sn	0	1	HF/SDD	16.979	0
179921	Sn	0	1	HF/SDD	16.979	0

Sheet1

ComparisonStage	GlobalHeavyAtomRMSD_A	MetalSiteRMSD_A	MetalLigandBondMAE_A	CoordinationAngleMAE_deg
OrbMol-v2	0.206011	0.120219	0.066535	3.828806
Native RapidMetalFF	0.342653	0.198000	0.121847	4.588060
Hybrid RapidMetalFF/GAFF2	0.308182	0.190881	0.117438	4.535400
OrbMol-v2	0.104660	0.078811	0.071446	0.771345
Native RapidMetalFF	0.910308	0.120030	0.130554	0.538157
Hybrid RapidMetalFF/GAFF2	0.886179	0.120479	0.130438	0.493640
OrbMol-v2	0.076846	0.080784	0.053586	1.849521
Native RapidMetalFF	0.610234	0.177406	0.148516	2.865368
Hybrid RapidMetalFF/GAFF2	0.555419	0.187960	0.150346	3.310803
OrbMol-v2	0.071238	0.053647	0.007189	1.778781
Native RapidMetalFF	0.333174	0.058123	0.003018	1.926154
Hybrid RapidMetalFF/GAFF2	0.084380	0.053572	0.030312	1.536173
OrbMol-v2	0.119506	0.104466	0.106984	0.548009
Native RapidMetalFF	0.097887	0.090905	0.097888	0.932876
Hybrid RapidMetalFF/GAFF2	0.103960	0.093422	0.098673	0.836601
OrbMol-v2	0.154324	0.052218	0.055826	2.752675
Native RapidMetalFF	0.178317	0.094351	0.042642	3.529105
Hybrid RapidMetalFF/GAFF2	0.191270	0.111485	0.034671	3.913896
OrbMol-v2	0.204774	0.049398	0.053807	0.899475
Native RapidMetalFF	0.278110	0.105137	0.073518	3.769435
Hybrid RapidMetalFF/GAFF2	0.267913	0.101189	0.071251	3.799927

Sheet1

OrbMol-v2	0.081810	0.049956	0.038279	1.370536
Native RapidMetalFF	0.120482	0.044574	0.030630	1.303031
Hybrid RapidMetalFF/GAFF2	0.123864	0.053970	0.032268	1.611878
OrbMol-v2	0.582829	0.080556	0.018256	3.403931
Native RapidMetalFF	0.850823	0.101407	0.011274	4.509323
Hybrid RapidMetalFF/GAFF2	0.605450	0.100442	0.009521	4.502876

Sheet1

Status	Notes
INCLUDED	Atom order/connectivity verified; HF/SDD frequency analysis had no imaginary modes
INCLUDED	Atom order/connectivity verified; HF/SDD frequency analysis had no imaginary modes
INCLUDED	Atom order/connectivity verified; HF/SDD frequency analysis had no imaginary modes
INCLUDED	Atom order/connectivity verified; HF/SDD frequency analysis had no imaginary modes
INCLUDED	Atom order/connectivity verified; HF/SDD frequency analysis had no imaginary modes
INCLUDED	Atom order/connectivity verified; HF/SDD frequency analysis had no imaginary modes
INCLUDED	Atom order/connectivity verified; HF/SDD frequency analysis had no imaginary modes
INCLUDED	Atom order/connectivity verified; HF/SDD frequency analysis had no imaginary modes
INCLUDED	Atom order/connectivity verified; HF/SDD frequency analysis had no imaginary modes
INCLUDED	Atom order/connectivity verified; HF/SDD frequency analysis had no imaginary modes
INCLUDED	Atom order/connectivity verified; HF/SDD frequency analysis had no imaginary modes
INCLUDED	Atom order/connectivity verified; HF/SDD frequency analysis had no imaginary modes
INCLUDED	Atom order/connectivity verified; HF/SDD frequency analysis had no imaginary modes
INCLUDED	Atom order/connectivity verified; HF/SDD frequency analysis had no imaginary modes
INCLUDED	Atom order/connectivity verified; HF/SDD frequency analysis had no imaginary modes

Sheet1

INCLUDED	Atom order/connectivity verified; HF/SDD frequency analysis had no imaginary modes
INCLUDED	Atom order/connectivity verified; HF/SDD frequency analysis had no imaginary modes
INCLUDED	Atom order/connectivity verified; HF/SDD frequency analysis had no imaginary modes
INCLUDED	Atom order/connectivity verified; HF/SDD frequency analysis had no imaginary modes
INCLUDED	Atom order/connectivity verified; HF/SDD frequency analysis had no imaginary modes
INCLUDED	Atom order/connectivity verified; HF/SDD frequency analysis had no imaginary modes
INCLUDED	Atom order/connectivity verified; HF/SDD frequency analysis had no imaginary modes
INCLUDED	Atom order/connectivity verified; HF/SDD frequency analysis had no imaginary modes
INCLUDED	Atom order/connectivity verified; HF/SDD frequency analysis had no imaginary modes
INCLUDED	Atom order/connectivity verified; HF/SDD frequency analysis had no imaginary modes
INCLUDED	Atom order/connectivity verified; HF/SDD frequency analysis had no imaginary modes
INCLUDED	Atom order/connectivity verified; HF/SDD frequency analysis had no imaginary modes
