# Supplementary material for: RapidMetalFF-AI: An AI-Assisted Web Server for Minute-Scale Bonded Force-Field Parameterization and Structural Validation of Metal-Containing Molecules with Validated AMBER and Converted GROMACS/CHARMM Outputs
Source: Molecules. 2026 Oct 17;31(20):3724. doi: 10.3390/molecules31203724 (PMC13707471; doi:10.3390/molecules31203724)
Supplement: Supplementary file 1 [file molecules-31-03724-s001.zip › pdf_versions_of_data/Supplementary_Table_S8_Explicit_Water_MD_Summary.pdf]

Sheet1

Molecule	Metal	Charge	Multiplicity	Electrostatics	Water	Padding_A	Heating_ps
1101146	Nb	0	1	GFN1-xTB/CM5	TIP3P	10	50
155447	Pt	0	1	GFN1-xTB/CM5	TIP3P	10	50
902738	Mo	-3	1	GFN1-xTB/CM5	TIP3P	10	50
1100319	Be	0	1	GFN1-xTB/CM5	TIP3P	10	50
1100364	Sc	0	1	GFN2-xTB follow-up substitution	TIP3P	10	50
232528	Au	0	1	GFN1-xTB/CM5	TIP3P	10	50
613741	In	0	1	GFN1-xTB/CM5	TIP3P	10	50
163152	Ga	0	1	GFN1-xTB/CM5	TIP3P	10	50
234071	Bi	0	1	GFN1-xTB/CM5	TIP3P	10	50
179921	Sn	0	1	GFN1-xTB/CM5	TIP3P	10	50

Sheet1

Equilibration_ps	Production_ps	Timestep_fs	Frames	Last1nsFrames	GlobalRMSDMean_A	GlobalRMSDLast1nsMean_A
100	5000	0.5	2500	501	0.553900	0.560470
100	5000	0.5	2500	501	0.569976	0.572404
100	5000	0.5	2500	501	0.774768	0.750235
100	5000	0.5	2500	501	0.560486	0.557426
100	5000	0.5	2500	501	0.571887	0.585195
100	5000	0.5	2500	501	1.166380	1.199979
100	5000	0.5	2500	501	0.370944	0.368657
100	5000	0.5	2500	501	1.126125	1.153650
100	5000	0.5	2500	501	0.522633	0.682971
100	5000	0.5	2500	501	1.761390	1.585656

Sheet1

GlobalRMSDSD_A	GlobalRMSDMax_A	MetalSiteRMSDMean_A	MetalSiteRMSDLast1nsMean_A	MetalSiteRMSDSD_A
0.168121	0.929397	0.191291	0.189993	0.037040
0.138476	1.046610	0.083192	0.082919	0.019431
0.289441	1.157500	0.104633	0.104328	0.021343
0.138702	1.082986	0.088707	0.088256	0.021572
0.126995	0.984237	0.114168	0.113369	0.023431
0.597986	2.046945	0.137624	0.137706	0.039896
0.111818	0.812173	0.182052	0.183172	0.039573
0.353380	1.854843	0.109113	0.111840	0.029577
0.289634	1.145337	0.105472	0.106742	0.027809
0.375688	2.704414	0.117437	0.117017	0.028330

Sheet1

MetalSiteRMSDMax_A	MeanTemperature_K	MeanDensity_g_cm3	Notes
0.339103	299.989	1.035341	RapidMetalFF parameter set; CUDA mixed precision; PME; 300 K; 1 bar
0.180614	299.931	1.037640	RapidMetalFF parameter set; CUDA mixed precision; PME; 300 K; 1 bar
0.177938	299.988	1.051955	RapidMetalFF parameter set; CUDA mixed precision; PME; 300 K; 1 bar; neutralized with 3 Na+
0.180621	300.052	1.026625	RapidMetalFF parameter set; CUDA mixed precision; PME; 300 K; 1 bar; input metadata warned all-zero charges; final xTB charges were nonzero
0.205616	300.097	1.027673	RapidMetalFF parameter set; CUDA mixed precision; PME; 300 K; 1 bar; GFN1 charge step failed; RapidMetalFF bonded/torsion/LJ retained
0.323910	299.829	1.061121	RapidMetalFF parameter set; CUDA mixed precision; PME; 300 K; 1 bar
0.350428	300.038	1.047760	RapidMetalFF parameter set; CUDA mixed precision; PME; 300 K; 1 bar
0.235025	300.138	1.030108	RapidMetalFF parameter set; CUDA mixed precision; PME; 300 K; 1 bar
0.211660	300.067	1.040005	RapidMetalFF parameter set; CUDA mixed precision; PME; 300 K; 1 bar
0.228450	299.848	1.032240	RapidMetalFF parameter set; CUDA mixed precision; PME; 300 K; 1 bar
