# Supplementary material for: RapidMetalFF-AI: An AI-Assisted Web Server for Minute-Scale Bonded Force-Field Parameterization and Structural Validation of Metal-Containing Molecules with Validated AMBER and Converted GROMACS/CHARMM Outputs
Source: Molecules. 2026 Oct 17;31(20):3724. doi: 10.3390/molecules31203724 (PMC13707471; doi:10.3390/molecules31203724)
Supplement: Supplementary file 1 [file molecules-31-03724-s001.zip › pdf_versions_of_data/Supplementary_Table_S9a_Flagged_Case_Resolution.pdf]

Sheet1

Record	HistoricalAtoms	HistoricalQ	HistoricalM	HistoricalChargeSum	Diagnosis
145192	42	0	1	~0	Incomplete C18H23Yb input with 201 electrons; Q0 M1 parity invalid
850911	18	0	1	-3	Periodic U phosphate fragment; repeated U and O images; xTB process failed and source charges propagated to topologies
993277	103	0	1	-1	Periodic Tb network fragment; repeated sites and disconnected O components; incomplete CM5 table; Orb not converged

Sheet1

Resolution	CorrectedAtoms	CorrectedSourceEdges	CorrectedTopologyQ
Separate 81 atom C32H46OYb2 Q0 M1 follow-up; native and hybrid rerun with source connectivity and elements preserved	81	106	-5.487778e-10
Classify supplied input as unsupported periodic representation; no arbitrary isolated charge or multiplicity assigned	NA	NA	NA
Classify supplied input as unsupported periodic representation; no arbitrary capping or spin assignment	NA	NA	NA

Sheet1

Status
Corrected follow-up verified; historical 42 atom record superseded for chemical interpretation
Historical topology not suitable for molecular validation or production use
Historical topology not suitable for molecular validation or production use
