# Supplementary material for: RapidMetalFF-AI: An AI-Assisted Web Server for Minute-Scale Bonded Force-Field Parameterization and Structural Validation of Metal-Containing Molecules with Validated AMBER and Converted GROMACS/CHARMM Outputs
Source: Molecules. 2026 Oct 17;31(20):3724. doi: 10.3390/molecules31203724 (PMC13707471; doi:10.3390/molecules31203724)
Supplement: Supplementary file 1 [file molecules-31-03724-s001.zip › pdf_versions_of_data/Supplementary_Table_S9b_Corrected_Topologies.pdf]

Sheet1

Molecule	atoms	source_edges	native_RMSD_vs_Orb_A	hybrid_RMSD_vs_Orb_A	all_checks_pass
158148	102	113	0.6388571053841516	0.35400048474044243	True
232591	43	52	0.059674143755764776	0.0378535357018687	True
251255	67	72	0.45400495114199374	0.49642101981509534	True
716039	91	109	0.778602035446825	0.8825815471628595	True
849384	79	80	0.40342583340804866	0.28561301789764626	True
