# Supplementary material for: RapidMetalFF-AI: An AI-Assisted Web Server for Minute-Scale Bonded Force-Field Parameterization and Structural Validation of Metal-Containing Molecules with Validated AMBER and Converted GROMACS/CHARMM Outputs
Source: Molecules. 2026 Oct 17;31(20):3724. doi: 10.3390/molecules31203724 (PMC13707471; doi:10.3390/molecules31203724)
Supplement: Supplementary file 1 [file molecules-31-03724-s001.zip › pdf_versions_of_data/Supplementary_Table_S9c_Archive_Recheck.pdf]

Sheet1

Molecule	ArchiveSHA256	ActualInputAtoms	Composition	StoredQ
1012591	1ace82ad4cee6193886bacf09085f8bf282e312f71b2b0a5a9bb7be09e12b266	89	{'C': 40, 'H': 32, 'Cl': 8, 'Er': 1, 'N': 4, 'O': 4}	0
1033814	140a12a54a13291c93bf95fd79ef8a301d04326d4580ed11597b2d46082645dc	72	{'C': 37, 'H': 26, 'F': 4, 'Ir': 1, 'N': 3, 'O': 1}	0
1100009	1224e407df722c173f1ed2d55ff55d9296bec114b895ba5de2720aa514734e6b	20	{'Ru': 2, 'O': 10, 'C': 8}	0
1100100	3db25854a6d7c769b28211e0df9295e0c8717f5976c493a0cbd57a50a17390ab	62	{'Zr': 1, 'Cl': 2, 'O': 2, 'N': 2, 'C': 23, 'H': 32}	0
1100131	f8dfa2fe0e8f515d82400c293ad5182b37b7616eb88899758bfe969cc3c0198b	38	{'Ge': 1, 'N': 5, 'C': 13, 'H': 19}	0
1100134	b81f701ecb47fc7dff8cb067e3973c38abf554d43cfa87382918d3533d4e9f08	15	{'Br': 4, 'C': 2, 'H': 5, 'N': 1, 'O': 2, 'Re': 1}	0
1100181	94851ed438a78da89d7a36c54306bf0ae3117ae3aa785d2f187afff2b30708f3	112	{'Y': 1, 'Si': 2, 'N': 6, 'C': 42, 'Li': 1, 'H': 60}	0
1100271	5af869cb210004009f87610cb7b074068cc18daf974abc8a16e22d5e5fde1420	98	{'C': 40, 'H': 50, 'O': 4, 'Pd': 2, 'P': 2}	0
1100319	cc6bc85e37f077393e96f8df563572a4f4dc5a9208810ebdc3e5a9b9f1d7fbfc	29	{'Be': 1, 'C': 10, 'H': 14, 'O': 4}	0
1100364	92eb38cedb30cbf4f37c23ad67370d2a91490c4a6601c057674be3649a9d7b6e	36	{'C': 15, 'H': 14, 'O': 6, 'Sc': 1}	0
1100416	f00e94c30e084da6e79c975fc58ed03b8ad904ed8602339a49df2a536bb4a9a6	37	{'Pt': 1, 'Cl': 2, 'O': 2, 'N': 2, 'C': 10, 'H': 20}	-2
1101146	128589110bd7aa500d19d089d9ed36f6c6532f7036c94e87c216c8c9b158299e	11	{'Nb': 1, 'Cl': 3, 'O': 1, 'N': 2, 'C': 4}	0
1101247	b192173073b04134bb3c0744d27e0d5f4c65ef5a1be415d5defd053fb1a83e28	23	{'Ta': 1, 'Cl': 7, 'P': 1, 'O': 1, 'N': 1, 'C': 4, 'H': 8}	0
145192	12a678263873c516c6de360f78a2b8c278ef365276996b7e1fe90b23ddf6cee0	42	{'Yb': 1, 'C': 18, 'H': 23}	0
1493407	7fa31340abd7eba90c4355729f9e1274afd8814bd97c4980b96e874e36dede58	41	{'Se': 2, 'Cl': 1, 'O': 1, 'C': 20, 'H': 17}	0

Sheet1

1506292	02caaf5c275ccdb35df0571e2041f92dda4eca9c7a620454898d73aeabef69be	123	{'Si': 2, 'Mg': 1, 'O': 1, 'N': 4, 'C': 41, 'H': 74}	0
1518810	1cc18d25ce780ac7197794d3b0bb24f63c0fb4cd9b7a4f90d888798ce4f4bc79	70	{'N': 3, 'S': 2, 'O': 4, 'C': 28, 'H': 30, 'Br': 2, 'Ag': 1}	0
1519012	7ed63fc91a65376b1f35429979b41aeaa580565dc4ae7232efc0b7188d910053	41	{'Sc': 1, 'S': 2, 'Cl': 2, 'N': 1, 'C': 11, 'H': 22, 'O': 2}	0
152002	ee59b7382b2cd41fbc1968c27b3361f03d28d9b1f18d67c365fd881de8d16ea1	56	{'Tc': 1, 'O': 3, 'S': 3, 'N': 1, 'C': 20, 'H': 28}	0
1530857	fa2469e6d66bbfb46d07f31cd23985417b5a9bea4217bd8742393e625e66bdc1	83	{'Tm': 1, 'S': 3, 'O': 6, 'F': 9, 'N': 4, 'C': 38, 'H': 22}	0
154031	e61371e71633aff3f0d5465c1276f153f8658e13655a8dd8fe71a1fd8f40f767	19	{'Pt': 1, 'Se': 2, 'O': 8, 'N': 2, 'C': 6}	0
154784	111ccf10d33f880c699b7f1d97e6cc1628816dc9b6a0b9cad02ce3990dbc4187	33	{'Pb': 3, 'C': 10, 'N': 5, 'S': 4, 'H': 11}	0
155447	a9c7bb7bc06be4f61b43db5501d2fd769b1cf7997eab69bab34ce4d1e882f337	35	{'Pt': 1, 'Cl': 2, 'N': 4, 'H': 20, 'C': 8}	0
156313	58140453490212716486f0c846c8ed2e3644e76c47b49dec0b86da4fa7ee59dc	46	{'Tc': 1, 'S': 4, 'P': 1, 'C': 18, 'H': 22}	0
1563993	89337fbee88c51e7a1bba06c6ef5580ad4eb764fbba5230e062650170890a0c7	137	{'C': 60, 'H': 65, 'B': 2, 'N': 8, 'P': 1, 'Th': 1}	0
1574551	d9e89ef8ef26587ac14a25b755348d401804609f42d558e3feb89e7baa206fe2	45	{'P': 1, 'C': 24, 'H': 20}	0
158148	c7d2f312a587dccb9ce1e4de1fcc93d59023f4b0cf5e2b0414521d5757d6ca3f	102	{'Eu': 1, 'N': 8, 'C': 40, 'H': 48, 'O': 5}	0
1582689	747f7d2d27b5fb0d183736266e797aee12df53e22614a19f4e4e11634dee0b39	43	{'Ga': 1, 'O': 6, 'C': 15, 'H': 21}	0
162486	66c9093b93a4a5c219a2dfd4a79b90325bd001d9b5936eb42923682e7efa565c	59	{'Sn': 1, 'In': 1, 'Cl': 2, 'O': 4, 'C': 16, 'H': 35}	0
163152	9ec6f15e2454e0425c56e854a4444a52eea06b56277db532f3a415a3cef0c0bc	38	{'Ga': 1, 'Cl': 2, 'N': 2, 'H': 23, 'C': 10}	0
175478	2233033d3dd4efa69e058c46dc8b8cd5fa1299c2ea32bfe4461c4527f0354706	29	{'Te': 1, 'C': 13, 'H': 15}	0

Sheet1

179921	8dde5fb43a760e7880c47d7a428846cf424fac7fe3671661394e4bb34288d7e8	44	{'Sn': 1, 'N': 1, 'O': 2, 'S': 1, 'C': 22, 'H': 17}	0
182741	63612e3a67002da20ee8f9ba357637a1acdec664d67b4fb9c256613e060241be	52	{'Tl': 1, 'S': 1, 'P': 1, 'Si': 1, 'N': 3, 'C': 16, 'H': 29}	0
183305	929422417393ca26e1a8cf7d17e88e124cf23233b00f26b1e656908453e1b96a	47	{'Cu': 3, 'O': 2, 'N': 6, 'C': 20, 'H': 16}	3
2051215	ee582e80f9b209319831d2e4416a5a867eb71fea925fd46d4d0641cee9e760fa	134	{'Sn': 2, 'C': 56, 'H': 70, 'Ba': 1, 'O': 5}	0
2074417	2f52b2848118963d6d5d1f007e3aeb2e4a46ccfadb3f17113b77f9e0f7f86481	115	{'Fe': 1, 'N': 3, 'C': 48, 'H': 63}	0
2075115	c21276d25efbb75c5ca9cf6a28ff8c2e814be563d03abeee28e903830e5823e8	39	{'Si': 1, 'N': 1, 'C': 11, 'H': 22, 'Cl': 1, 'O': 3}	1
2075193_as	44b268405ad1f94b162531da551fed7f7be65fbbaedc4b80bc65324e880ab65d	64	{'O': 8, 'C': 24, 'H': 32}	0
2076769	a8e9b4f6c35d2755699be125ced5b11af1a977db0b43decb9ec0aba18d889325	27	{'Cl': 1, 'F': 2, 'N': 2, 'C': 13, 'H': 8, 'B': 1}	0
2076771	ddc8af9e13fce8e169452f1a7619f7492c0cb7e2b8bf33d522309f6eef2d439a	27	{'I': 1, 'F': 2, 'N': 2, 'C': 13, 'H': 8, 'B': 1}	0
2083565	db267b7f191b07f839b713eac12ad1d4ddb9e7113afa46596624ad45a87601ea	35	{'W': 2, 'P': 1, 'S': 1, 'O': 10, 'C': 16, 'H': 5}	0
2083570	83db087c3a3cfe8031d1936654854b5e5986ffda2f2e3afff04750937598593e	42	{'W': 2, 'Se': 2, 'O': 8, 'C': 20, 'H': 10}	0
2087213	f861b1660e1a709562f58d9bfdc8d9e986494ae359d32bc5a145c7a285ac4ee9	218	{'C': 92, 'H': 116, 'Ga': 2, 'N': 6, 'Se': 2}	0
2087214	d64d343891d989c8cf3cb8526889dd170217fce0966642e6172eea23675fc362	119	{'Cr': 1, 'Ga': 1, 'N': 3, 'O': 5, 'C': 51, 'H': 58}	0
2087215	bbbe6645879efe84d2d1ab3354b19274fa217795d54911eaad242b2603a71bb9	116	{'Al': 1, 'C': 48, 'H': 64, 'N': 3}	0
2091598	440e9bb5e29de5e275077d8750915a1ad16577bed8c057218d8900b880d9956e	83	{'Mg': 1, 'O': 10, 'F': 24, 'C': 20, 'H': 26, 'B': 2}	0
2096425	4d784a1aa5a945539dc75da255a2aa1bd4d17f9e2d93d920632f154ec9a99a91	66	{'Al': 1, 'Li': 1, 'N': 3, 'C': 32, 'H': 29}	0

Sheet1

2104359	8dea4a87c15edf2620a86b2678722a5ae48d87120f5e0654a8ec4c95400c98eb	59	{'Ru': 1, 'N': 6, 'C': 30, 'H': 22}	0
2150100	4d28ab75d1e8ea8fb74f432fb950750a7147620ab8e9de6e434ab227820e630f	31	{'N': 2, 'C': 15, 'O': 2, 'H': 12}	0
229783	6d60bdbb5b0564ec8cdc40d8f5a0dfe8745291651a1ad800672a3abbba9c84d9	46	{'Rh': 1, 'Br': 1, 'C': 18, 'O': 2, 'N': 3, 'H': 21}	0
232528	0eec99077535d98b59cf7962afed7db78f5fbb635d1cfc00f11e673b04672f29	17	{'Au': 1, 'I': 3, 'P': 1, 'C': 3, 'H': 9}	0
232536	e8fe89bde252b1b60f7c6c3950785000348675b3ead08f85ff3de4e933b520fe	53	{'Au': 2, 'Br': 2, 'P': 2, 'C': 25, 'H': 22}	0
232591	ae285181751506c2e07c9726d6ba9bbc388c486a59eee34df76749f45088fe27	43	{'Fe': 1, 'P': 2, 'C': 16, 'H': 24}	0
234033	a77ec7eb63711819059ee50022cdc2bdf713cd8c645584e735b006f48f9b024c	28	{'Pb': 3, 'Cl': 4, 'C': 10, 'H': 8, 'N': 3}	0
234071	b2b1709c7a5baec92f75e76f81b859c6c11f938eb8af675bac21293e2e6a7e6c	42	{'Bi': 1, 'Cl': 1, 'N': 1, 'C': 18, 'H': 21}	0
236914	c1ee7a236123489f747d83ceed40d65fd236f374d2fb32f95ba42dc522248ee7	27	{'Mo': 1, 'Cl': 2, 'S': 2, 'C': 6, 'H': 14, 'O': 2}	0
2378332	5e7761a7bc023f571f057dbda1f134b2a1e82a5ef6a2f25bddd56ea301b7faea	49	{'Au': 1, 'N': 2, 'C': 14, 'H': 28, 'S': 4}	0
243849	f137ee785fa5e5c860e2229e91f8d758b6ab18591c80707b8c5c94819277cf78	71	{'Pd': 1, 'Cl': 2, 'C': 34, 'N': 2, 'H': 32}	0
244175	ff73aeb418fc72d75ca736bec109daca61bd6761eee0b8eea0e0fa5bf278e8d6	68	{'Zn': 1, 'Cl': 2, 'O': 2, 'N': 2, 'C': 30, 'H': 31}	0
251255	9362f09ebb56eba5de0cc53e13cf6ed777a742368886ef1a20ac3bd47602a8d6	67	{'Hg': 1, 'N': 8, 'C': 30, 'H': 28}	0
2515638	6de15104974923ae39dc6605f49a273f55a92c6a0a386f2f3ae10cd9bf85ea93	24	{'N': 1, 'H': 16, 'C': 7}	1
253279	93f1edcbfc5742c1e4e7ff6505314940289d7c468e867002f7d951bd20e6bc7f	250	{'Pd': 2, 'P': 6, 'C': 134, 'H': 106, 'N': 2}	0
253288	e7e7dbd7b021e2a0a32e461b6bda4def80bd0c51c5d37651ed683598d6493fa4	73	{'Ti': 1, 'O': 4, 'C': 32, 'H': 36}	0

Sheet1

253291	dc1f615544da5995e0d3386e22f08c0b7f9d589bbc98293654fb6d99a04cf274	196	{'Ti': 2, 'Rb': 2, 'N': 2, 'O': 10, 'C': 60, 'H': 120}	0
2535798	e4b79a1a0e04015a99efdc8536b8d99a1541c977280ca4dd7ee55f4052bbde1b	111	{'Mo': 1, 'P': 4, 'O': 3, 'C': 55, 'H': 48}	0
602743	22c6b648116868cb18b03b0e86b62dc3639157a550a83eac1285715aefab1e93	52	{'Sb': 1, 'S': 3, 'O': 3, 'C': 24, 'H': 21}	0
613741	f38b55ced8d0bb2970fd6098707f7ccc8cf8a1886e47d8585c1dc71f9c31eb80	32	{'In': 1, 'Br': 3, 'N': 3, 'H': 13, 'C': 12}	0
716039	f37e457891fbbcf1c6a31811f2953bc2e0cc53d6be26e543aa9068a1119d87b1	91	{'Ho': 3, 'S': 4, 'N': 14, 'H': 28, 'C': 42}	0
780531	c575eb479b1d4e8c5a7132ddd8676045af73bb194e2de99cbb323deeecf96e3f	62	{'Re': 1, 'Cl': 2, 'P': 1, 'O': 2, 'N': 2, 'C': 28, 'H': 26}	0
791724	c716a160095059d16ba2930ea8195fe79a093bf7d3a01cd8aa8ef43e1c3f914e	49	{'Mn': 1, 'S': 4, 'N': 8, 'H': 16, 'C': 20}	0
801553	6209fba55847daba57553697117afa988c80137fb8557c0d40115594da6f464f	52	{'Tm': 1, 'Al': 3, 'C': 12, 'H': 36}	0
804893	2c68a1ef62de53da621ecf9dd421585817fdb4b03884be18b6006061ec6d1c22	57	{'Ce': 1, 'Br': 3, 'N': 5, 'C': 25, 'H': 23}	0
804894	f049b38226816ef240ac8bda4333bc8feac61569468b7844842f65dbf1e7693b	68	{'Ce': 1, 'Br': 3, 'O': 4, 'C': 20, 'H': 40}	0
804896	2729c79d34aa3c076b3e934d177a709845f107d740d519ff51fcf22ec259385f	34	{'La': 1, 'Br': 3, 'N': 5, 'C': 10, 'H': 15}	0
849384	9e14cf36dc9fdf26f1f36b6266096d4e9067cdcf8ee613779f1b8b0bc86a5475	79	{'Os': 1, 'H': 50, 'P': 2, 'N': 2, 'C': 24}	0
850911	2af91a8f9a21b8012ba461b36a26038bc62390734467be61c4bc627c10fa4fc6	18	{'U': 4, 'P': 2, 'O': 12}	0
850947	ca6928a079c93fe8ec8bc3b1e56a0a392b785aa150735153d4da77ea20c2b6fb	79	{'Cu': 1, 'Te': 1, 'P': 3, 'C': 24, 'H': 50}	0
851092	98f4f23cd077d904f6ae61fe24a95503398661593793580482cda29aadfc7ac2	63	{'K': 1, 'N': 2, 'O': 6, 'C': 18, 'H': 36}	0
876252	cbc9ef90d486ca33de5021d018d46bbaf201880055cbef6d473d7486b5bac964	67	{'Si': 1, 'O': 6, 'N': 2, 'C': 32, 'H': 26}	0

Sheet1

902738	28d0140a1e97f90e171b48e7bfa8f95c5ea70c555804b4f9d758cdae88dc99b8	23	{'C': 6, 'N': 1, 'O': 9, 'Mo': 1, 'H': 6}	0
942961	5d3b6363ea92765ba03fb1a4c2077b5b6513bd64fa7970fdc5e98671d5d5f157	81	{'Sr': 3, 'N': 8, 'H': 22, 'O': 8, 'C': 40}	0
993277	db4e54361146489d39df10b4bc08570cc1ee89becb6fd538db4433e83af52b89	103	{'Tb': 8, 'S': 4, 'N': 4, 'C': 44, 'H': 24, 'O': 19}	0

Sheet1

StoredM	AllElectronCount	ParityPass	OrbConverged	OrbFinalFmax	FinalMOL2ChargeSum
1	536	True	True	0.00852158386260271	3.999999999981796e-05
1	390	True	True	0.00831322930753231	2.000000000035307e-05
1	216	True	True	0.008367963135242462	1.0000000000426335e-05
1	274	True	True	0.006311144679784775	4.996003610813204e-16
1	164	True	True	0.008304968476295471	2.000000000008939e-05
2	255	True	True	0.008091330528259277	2.220446049250313e-16
1	424	True	True	0.0089228181168437	0.0
1	444	True	True	0.00669444864615798	2.9999999999141824e-05
1	110	True	True	0.009076236747205257	9.99999999995449e-06
2	173	True	True	0.007911443710327148	0.0
1	224	True	True	0.008818045258522034	-1.9999999999999982
1	138	True	True	0.00906662829220295	-4.0592529337857286e-16
1	254	True	True	0.008256085216999054	-8.326672684688674e-17
1	201	False	True	0.007971815764904022	1.0000000000259801e-05
1	230	True	True	0.006398782134056091	-3.999999999995674e-05
1	396	True	True	0.00889231264591217	-4.000000000062287e-05
1	400	True	False	0.018970362842082977	3.999999999998449e-05
1	198	True	True	0.007401816546916962	0.0
1	270	True	True	0.008651070296764374	3.0000000000265925e-05
1	524	True	True	0.009218275547027588	-3.999999999994286e-05
1	260	True	True	0.006147474050521851	1.9999999999742446e-05

Sheet1

1	416	True	True	0.009095308370888233	3.000000000030756e-05
1	208	True	True	0.006715286523103714	5.551115123125783e-17
1	252	True	True	0.007807175163179636	-1.0000000000010001e-05
1	596	True	False	0.01202262844890356	0.0
2	179	True	True	0.006636627018451691	4.163336342344337e-17
2	447	True	True	0.007511302828788757	-9.999999999399378e-06
1	190	True	True	0.007692188024520874	-3.000000000023817e-05
1	296	True	True	0.008196461945772171	1.0000000000121023e-05
1	162	True	True	0.007168348878622055	2.000000000003388e-05
2	145	True	True	0.0085934828966856	-1.3877787807814457e-16
1	238	True	True	0.006206538528203964	2.220446049250313e-16
1	272	True	True	0.008950348012149334	1.0000000000107145e-05
1	278	True	True	0.00905386172235012	3.000020000000001
1	602	True	False	0.013392344117164612	-8.326672684688674e-17
1	398	True	True	0.008581750094890594	2.0000000000436335e-05
2	149	True	True	0.008495933376252651	0.9999999999999998
1	240	True	True	0.0071667879819869995	-9.9999999988859e-06
1	140	True	True	0.009211922064423561	9.999999999732445e-06
1	176	True	True	0.008496996946632862	-1.0000000000259801e-05
1	360	True	True	0.006129149347543716	-1.1102230246251565e-16
1	410	True	True	0.007285088300704956	2.000000000021429e-05
1	840	True	True	0.009527409449219704	-1.2490009027033011e-15

Sheet1

1	480	True	True	0.008536167442798615	-4.9999999997857314e-05
1	386	True	True	0.009546257555484772	3.0000000000640625e-05
1	464	True	True	0.009182948619127274	-2.000000000046409e-05
1	258	True	True	0.007488163188099861	4.579669976578771e-16
1	288	True	True	0.009655892848968506	6.000000000051797e-05
1	132	True	True	0.008615279570221901	-2.0000000000117146e-05
1	246	True	True	0.008217133581638336	-2.7755575615628914e-16
1	280	True	True	0.006745949387550354	-1.0000000000065512e-05
1	430	True	True	0.008495844900608063	-1.0000000000107145e-05
1	176	True	True	0.006533516570925713	2.9999999999932858e-05
2	403	True	True	0.008137737400829792	-1.0000000000065512e-05
1	236	True	True	0.007406294345855713	-9.999999999843467e-06
1	174	True	True	0.006870415061712265	9.9999999997602e-06
2	269	True	True	0.008613340556621552	-3.999999999998449e-05
1	330	True	True	0.006997436285018921	1.0000000000259801e-05
2	305	True	True	0.008846668526530266	2.9999999998947535e-05
1	344	True	True	0.008436385542154312	-3.000000000061287e-05
1	64	True	True	0.006732091307640076	0.9999999999999999
1	1106	True	False	0.04714753106236458	-5.000000000000837e-05
1	282	True	True	0.00952569767832756	0.0
1	692	True	True	0.008896652609109879	0.0
1	504	True	True	0.009627997875213623	0.0

Sheet1

1	288	True	True	0.008586272597312927	-1.0000000000537357e-05
1	260	True	True	0.008190438151359558	1.0000000000107145e-05
2	643	True	True	0.0075628384947776794	0.0
1	348	True	True	0.007845742627978325	1.0000000000065512e-05
2	281	True	True	0.007596650160849094	2.9999999999752447e-05
1	216	True	True	0.0076830387115478516	-3.0000000000016125e-05
2	371	True	True	0.007756710052490234	0.0
2	355	True	True	0.008938070386648178	0.0
1	272	True	True	0.006653603166341782	0.0
1	314	True	True	0.009609218686819077	1.999999999990898e-05
1	494	True	True	0.007400408387184143	-3.0
1	320	True	True	0.008950889110565186	0.0
2	225	True	True	0.007705681957304478	1.0000000000148779e-05
1	294	True	True	0.007957793772220612	-4.999999999995286e-05
2	163	True	True	0.006948292255401611	-2.7755575615628914e-16
1	496	True	True	0.00850754976272583	-9.999999999898979e-06
1	1052	True	False	0.01613938808441162	-1.0

Sheet1

Warnings	ElectronicGroundStateValidated	NativeTopologyChargeSum	HybridTopologyChargeSum
PRINCIPAL_COMPONENT_SELECTED	False	4.000757313826764e-05	4.000757313826764e-05
PRINCIPAL_COMPONENT_SELECTED	False	2.0003676814095428e-05	2.0003676814095428e-05
PRINCIPAL_COMPONENT_SELECTED	False	9.995664652234781e-06	9.995664652234781e-06
	False	-3.2926697479673805e-10	-3.2926697479673805e-10
	False	2.0001481701559243e-05	2.0001481701559243e-05
PRINCIPAL_COMPONENT_SELECTED	False	2.743890670231508e-09	2.743890670231508e-09
PRINCIPAL_COMPONENT_SELECTED;XTB_CHARGE_FAILED	False	0.0	0.0
PRINCIPAL_COMPONENT_SELECTED	False	3.000109755646463e-05	3.000109755646463e-05
PRINCIPAL_COMPONENT_SELECTED	False	1.0002030479516755e-05	1.0002030479516755e-05
XTB_CHARGE_FAILED	False	0.0	0.0
PRINCIPAL_COMPONENT_SELECTED	False	-1.9999999969817202	-1.9999999969817202
	False	-8.231669963720822e-10	-8.231669963720822e-10
	False	-5.487781340463016e-10	-5.487781340463016e-10
	False	1.000148170111903e-05	1.000148170111903e-05
	False	-3.9999615855268456e-05	-3.9999615855268456e-05
	False	-3.99955000193325e-05	-3.99955000193325e-05
	False	4.000801216058614e-05	4.000801216058614e-05
XTB_CHARGE_FAILED	False	0.0	0.0
PRINCIPAL_COMPONENT_SELECTED	False	2.999681708761026e-05	2.999681708761026e-05
PRINCIPAL_COMPONENT_SELECTED	False	-4.0003073159919356e-05	-4.0003073159919356e-05
PRINCIPAL_COMPONENT_SELECTED	False	1.9999670733128472e-05	1.9999670733128472e-05

Sheet1

	False	2.9999945122094474e-05	2.9999945122094474e-05
PRINCIPAL_COMPONENT_SELECTED	False	4.664613723059929e-09	4.664613723059929e-09
PRINCIPAL_COMPONENT_SELECTED	False	-9.999396343940425e-06	-9.999396343940425e-06
XTB_CHARGE_FAILED	False	0.0	0.0
PRINCIPAL_COMPONENT_SELECTED	False	2.1951118700513916e-10	2.1951118700513916e-10
PRINCIPAL_COMPONENT_SELECTED	False	-9.994951241543149e-06	-9.994951241543149e-06
	False	-3.0001152434094847e-05	-3.0001152434094847e-05
	False	1.0006420703811969e-05	1.0006420703811969e-05
	False	1.999341466225879e-05	1.999341466225879e-05
PRINCIPAL_COMPONENT_SELECTED	False	-8.231671871916646e-10	-8.231671871916646e-10
	False	7.68289667996136e-10	7.68289667996136e-10
	False	9.998189031815152e-06	9.998189031815152e-06
PRINCIPAL_COMPONENT_SELECTED	False	3.000020005158515	3.000020005158515
PRINCIPAL_COMPONENT_SELECTED	False	7.957283748583066e-09	7.957283748583066e-09
PRINCIPAL_COMPONENT_SELECTED	False	1.9999286588592846e-05	1.9999286588592846e-05
	False	0.9999999996158554	0.9999999996158554
PRINCIPAL_COMPONENT_SELECTED	False	-9.999835365967491e-06	-9.999835365967491e-06
	False	9.999067076882362e-06	9.999067076882362e-06
PRINCIPAL_COMPONENT_SELECTED	False	-1.000104267837032e-05	-1.000104267837032e-05
PRINCIPAL_COMPONENT_SELECTED	False	1.3719454322602687e-09	1.3719454322602687e-09
	False	1.9998573176902013e-05	1.9998573176902013e-05
PRINCIPAL_COMPONENT_SELECTED	False	5.487793830472043e-10	5.487793830472043e-10

Sheet1

PRINCIPAL_COMPONENT_SELECTED	False	-5.000345730205025e-05	-5.000345730205025e-05
PRINCIPAL_COMPONENT_SELECTED	False	3.0001920722944053e-05	3.0001920722944053e-05
	False	-2.000762801501743e-05	-2.000762801501743e-05
	False	4.225591807016649e-09	4.225591807016649e-09
PRINCIPAL_COMPONENT_SELECTED	False	6.000230486809255e-05	6.000230486809255e-05
	False	-1.9998079276328795e-05	-1.9998079276328795e-05
	False	-9.71445146547012e-16	-9.71445146547012e-16
	False	-9.999286588277534e-06	-9.999286588277534e-06
	False	-1.0000878045160477e-05	-1.0000878045160477e-05
	False	3.0000329266838266e-05	3.0000329266838266e-05
	False	-1.0002634135142241e-05	-1.0002634135142241e-05
	False	-9.998518298817705e-06	-9.998518298817705e-06
	False	1.0002579257692434e-05	1.0002579257692434e-05
PRINCIPAL_COMPONENT_SELECTED	False	-3.999983536645546e-05	-3.999983536645546e-05
PRINCIPAL_COMPONENT_SELECTED	False	9.999341466809808e-06	9.999341466809808e-06
PRINCIPAL_COMPONENT_SELECTED	False	3.00038963246263e-05	3.00038963246263e-05
PRINCIPAL_COMPONENT_SELECTED	False	-3.0003512180007408e-05	-3.0003512180007408e-05
PRINCIPAL_COMPONENT_SELECTED	False	1.0000000005487777	1.0000000005487777
PRINCIPAL_COMPONENT_SELECTED	False	-4.999868293263654e-05	-4.999868293263654e-05
XTB_CHARGE_FAILED	False	0.0	0.0
XTB_CHARGE_FAILED	False	0.0	0.0
PRINCIPAL_COMPONENT_SELECTED	False	-4.939000458614728e-10	-4.939000458614728e-10

Sheet1

	False	-1.0000384144351337e-05	-1.0000384144351337e-05
	False	9.997091475935638e-06	9.997091475935638e-06
XTB_CHARGE_FAILED	False	0.0	0.0
	False	1.0000439022953e-05	1.0000439022953e-05
PRINCIPAL_COMPONENT_SELECTED	False	3.000170121225665e-05	3.000170121225665e-05
	False	-2.99973109871704e-05	-2.99973109871704e-05
XTB_CHARGE_FAILED	False	0.0	0.0
XTB_CHARGE_FAILED	False	0.0	0.0
PRINCIPAL_COMPONENT_SELECTED;XTB_CHARGE_FAILED	False	0.0	0.0
	False	1.9998024398962255e-05	1.9998024398962255e-05
PRINCIPAL_COMPONENT_SELECTED;XTB_CHARGE_FAILED	False	-3.0	-3.0
	False	2.085356998193788e-09	2.085356998193788e-09
PRINCIPAL_COMPONENT_SELECTED	False	9.998079277373506e-06	9.998079277373506e-06
	False	-5.0000329267135823e-05	-5.0000329267135823e-05
PRINCIPAL_COMPONENT_SELECTED	False	-3.896324596297518e-09	-3.896324596297518e-09
	False	-9.999560977247413e-06	-9.999560977247413e-06
PRINCIPAL_COMPONENT_SELECTED;XTB_CHARGE_FAILED	False	-1.0	-1.0
