# Supplementary material for: RapidMetalFF-AI: An AI-Assisted Web Server for Minute-Scale Bonded Force-Field Parameterization and Structural Validation of Metal-Containing Molecules with Validated AMBER and Converted GROMACS/CHARMM Outputs
Source: Molecules. 2026 Oct 17;31(20):3724. doi: 10.3390/molecules31203724 (PMC13707471; doi:10.3390/molecules31203724)
Supplement: Supplementary file 1 [file molecules-31-03724-s001.zip › pdf_versions_of_data/Supplementary_Table_S9d_Sensitivity.pdf]

Sheet1

Source	Metric	Population	N
Supplementary_Table_S2_Experimental_RMSD_Exact82_Strict72_CORRECTED.csv	ExperimentalToOrbOptimizedRMSD_A	original_82	82
Supplementary_Table_S2_Experimental_RMSD_Exact82_Strict72_CORRECTED.csv	ExperimentalToOrbOptimizedRMSD_A	sensitivity_without_flagged_three	79
Supplementary_Table_S2_Experimental_RMSD_Exact82_Strict72_CORRECTED.csv	ExperimentalToRapidMetalFFMinimizedRMSD_A	original_82	82
Supplementary_Table_S2_Experimental_RMSD_Exact82_Strict72_CORRECTED.csv	ExperimentalToRapidMetalFFMinimizedRMSD_A	sensitivity_without_flagged_three	79
Supplementary_Table_S2_Experimental_RMSD_Exact82_Strict72_CORRECTED.csv	ExperimentalToHybridMinimizedRMSD_A	original_82	82
Supplementary_Table_S2_Experimental_RMSD_Exact82_Strict72_CORRECTED.csv	ExperimentalToHybridMinimizedRMSD_A	sensitivity_without_flagged_three	79
Supplementary_Table_S2_Experimental_RMSD_Exact82_Strict72_CORRECTED.csv	ExperimentalToRapidMetalFFMinimizedMetalSiteRMSD_A	original_82	72
Supplementary_Table_S2_Experimental_RMSD_Exact82_Strict72_CORRECTED.csv	ExperimentalToRapidMetalFFMinimizedMetalSiteRMSD_A	sensitivity_without_flagged_three	69
Supplementary_Table_S2_Experimental_RMSD_Exact82_Strict72_CORRECTED.csv	ExperimentalToHybridMinimizedMetalSiteRMSD_A	original_82	72
Supplementary_Table_S2_Experimental_RMSD_Exact82_Strict72_CORRECTED.csv	ExperimentalToHybridMinimizedMetalSiteRMSD_A	sensitivity_without_flagged_three	69
Supplementary_Table_S3_AI_Supplementary_Table_S1_Exact82_CORRECTED.csv	ExperimentalGeometryRMSD_A	original_82	82
Supplementary_Table_S3_AI_Supplementary_Table_S1_Exact82_CORRECTED.csv	ExperimentalGeometryRMSD_A	sensitivity_without_flagged_three	79
Supplementary_Table_S3_AI_Supplementary_Table_S1_Exact82_CORRECTED.csv	OrbGlobalRMSD_A	original_82	82
Supplementary_Table_S3_AI_Supplementary_Table_S1_Exact82_CORRECTED.csv	OrbGlobalRMSD_A	sensitivity_without_flagged_three	79
Supplementary_Table_S3_AI_Supplementary_Table_S1_Exact82_CORRECTED.csv	HybridGlobalRMSD_A	original_82	82

Sheet1

Supplementary_Table_S3_AI_Supplementary_Table_S1_Exact82_CORRECTED.csv	HybridGlobalRMSD_A	sensitivity_without_flagged_three	79
Supplementary_Table_S3_AI_Supplementary_Table_S1_Exact82_CORRECTED.csv	OrbMetalSiteRMSD_A	original_82	72
Supplementary_Table_S3_AI_Supplementary_Table_S1_Exact82_CORRECTED.csv	OrbMetalSiteRMSD_A	sensitivity_without_flagged_three	69
Supplementary_Table_S3_AI_Supplementary_Table_S1_Exact82_CORRECTED.csv	HybridMetalSiteRMSD_A	original_82	72
Supplementary_Table_S3_AI_Supplementary_Table_S1_Exact82_CORRECTED.csv	HybridMetalSiteRMSD_A	sensitivity_without_flagged_three	69
Supplementary_Table_S3_AI_Supplementary_Table_S1_Exact82_CORRECTED.csv	OptimizedTrustRMSD_A	original_82	72
Supplementary_Table_S3_AI_Supplementary_Table_S1_Exact82_CORRECTED.csv	OptimizedTrustRMSD_A	sensitivity_without_flagged_three	69

Sheet1

Mean	Median	Max
0.4122021097560975	0.2779555	2.531577
0.3942576329113924	0.27553	2.531577
0.6918508780487805	0.6262099999999999	2.580833
0.6743999620253165	0.616513	2.580833
0.6748690731707316	0.6226754999999999	3.792733
0.6630436835443038	0.622216	3.792733
0.36229843055555555	0.176027	2.764377
0.3179257391304347	0.170487	2.301505
0.40135288888888887	0.16576950000000001	4.913271
0.36512915942028984	0.153232	4.913271
0.4122021097560975	0.2779555	2.531577
0.3942576329113924	0.27553	2.531577
0.5295265609756097	0.430222	1.788019
0.5094581012658228	0.424706	1.788019
0.547680256097561	0.414906	3.742331
0.524294	0.409687	3.742331
0.24733256944444446	0.07890649999999999	2.226525
0.20454981159420293	0.073433	2.226525
0.3155107222222222	0.106668	4.837785
0.26080442028985507	0.096854	4.837785
0.41884365277777774	0.2843605	3.1486650000000003

Sheet1

0.3824172246376812	0.279175	3.1486650000000003
