# Supplementary material for: RapidMetalFF-AI: An AI-Assisted Web Server for Minute-Scale Bonded Force-Field Parameterization and Structural Validation of Metal-Containing Molecules with Validated AMBER and Converted GROMACS/CHARMM Outputs
Source: Molecules. 2026 Oct 17;31(20):3724. doi: 10.3390/molecules31203724 (PMC13707471; doi:10.3390/molecules31203724)
Supplement: Supplementary file 1 [file molecules-31-03724-s001.zip › pdf_versions_of_data/Supplementary_Table_S9_Charge_Multiplicity_Audit_Exact90.pdf]

Sheet1

Molecule	FinalOutcome	Charge	Multiplicity	ChargeSpinSource	MetadataVsOrbStage
1012591	COMPLETED_RMSD_REPORT	0	1	auto	PASS
1033814	COMPLETED_RMSD_REPORT	0	1	auto	PASS
1100009	COMPLETED_RMSD_REPORT	0	1	auto	PASS
1100100	COMPLETED_RMSD_REPORT	0	1	auto	PASS
1100131	COMPLETED_RMSD_REPORT	0	1	auto	PASS
1100134	COMPLETED_RMSD_REPORT	0	2	auto	PASS
1100181	COMPLETED_RMSD_REPORT	0	1	auto	PASS
1100271	COMPLETED_RMSD_REPORT	0	1	auto	PASS
1100319	COMPLETED_RMSD_REPORT	0	1	auto	PASS
1100364	COMPLETED_RMSD_REPORT	0	2	auto	PASS
1100369	SCREENED_IN_NO_COMPLETE_RMSD_IN_SUPPLIED_REPORT	N/A	N/A	N/A	N/A
1100416	COMPLETED_RMSD_REPORT	-2	1	auto	PASS
1101146	COMPLETED_RMSD_REPORT	0	1	auto	PASS
1101247	COMPLETED_RMSD_REPORT	0	1	auto	PASS
1107459	SCREENED_IN_NO_COMPLETE_RMSD_IN_SUPPLIED_REPORT	N/A	N/A	N/A	N/A
145192	COMPLETED_RMSD_REPORT	0	1	manual	PASS
1493407	COMPLETED_RMSD_REPORT	0	1	auto	PASS
1506292	COMPLETED_RMSD_REPORT	0	1	auto	PASS
1518810	COMPLETED_RMSD_REPORT	0	1	auto	PASS
1519012	COMPLETED_RMSD_REPORT	0	1	auto	PASS

Sheet1

152002	COMPLETED_RMSD_REPORT	0	1	auto	PASS
1530857	COMPLETED_RMSD_REPORT	0	1	auto	PASS
154031	COMPLETED_RMSD_REPORT	0	1	auto	PASS
154784	COMPLETED_RMSD_REPORT	0	1	auto	PASS
155447	COMPLETED_RMSD_REPORT	0	1	auto	PASS
156313	COMPLETED_RMSD_REPORT	0	1	auto	PASS
1563993	COMPLETED_RMSD_REPORT	0	1	auto	PASS
1574551	COMPLETED_RMSD_REPORT	0	2	auto	PASS
158148	COMPLETED_RMSD_REPORT	0	2	auto	PASS
1582689	COMPLETED_RMSD_REPORT	0	1	auto	PASS
162486	COMPLETED_RMSD_REPORT	0	1	auto	PASS
163152	COMPLETED_RMSD_REPORT	0	1	auto	PASS
175478	COMPLETED_RMSD_REPORT	0	2	auto	PASS
179921	COMPLETED_RMSD_REPORT	0	1	auto	PASS
1822165	SCREENED_IN_NO_COMPLETE_RMSD_IN_SUPPLIED_REPORT	N/A	N/A	N/A	N/A
182741	COMPLETED_RMSD_REPORT	0	1	auto	PASS
183305	COMPLETED_RMSD_REPORT	3	1	auto	PASS
2051215	COMPLETED_RMSD_REPORT	0	1	auto	PASS
2074417	COMPLETED_RMSD_REPORT	0	1	auto	PASS
2075115	COMPLETED_RMSD_REPORT	1	2	auto	PASS
2075193_as	COMPLETED_RMSD_REPORT	0	1	auto	PASS
2076769	COMPLETED_RMSD_REPORT	0	1	auto	PASS

Sheet1

2076771	COMPLETED_RMSD_REPORT	0	1	auto	PASS
2083565	COMPLETED_RMSD_REPORT	0	1	auto	PASS
2083570	COMPLETED_RMSD_REPORT	0	1	auto	PASS
2083695	SCREENED_IN_NO_COMPLETE_RMSD_IN_SUPPLIED_REPORT	N/A	N/A	N/A	N/A
2087213	COMPLETED_RMSD_REPORT	0	1	auto	PASS
2087214	COMPLETED_RMSD_REPORT	0	1	auto	PASS
2087215	COMPLETED_RMSD_REPORT	0	1	auto	PASS
2091598	COMPLETED_RMSD_REPORT	0	1	auto	PASS
2096425	COMPLETED_RMSD_REPORT	0	1	auto	PASS
2104359	COMPLETED_RMSD_REPORT	0	1	auto	PASS
2150100	COMPLETED_RMSD_REPORT	0	1	auto	PASS
229783	COMPLETED_RMSD_REPORT	0	1	auto	PASS
232528	COMPLETED_RMSD_REPORT	0	1	auto	PASS
232536	COMPLETED_RMSD_REPORT	0	1	auto	PASS
232591	COMPLETED_RMSD_REPORT	0	1	auto	PASS
234033	COMPLETED_RMSD_REPORT	0	2	auto	PASS
234071	COMPLETED_RMSD_REPORT	0	1	auto	PASS
236914	COMPLETED_RMSD_REPORT	0	1	auto	PASS
2378332	COMPLETED_RMSD_REPORT	0	2	auto	PASS
241977	SCREENED_IN_NO_COMPLETE_RMSD_IN_SUPPLIED_REPORT	N/A	N/A	N/A	N/A
243849	COMPLETED_RMSD_REPORT	0	1	auto	PASS

Sheet1

244158	SCREENED_IN_NO_COMPLETE_RMSD_IN_SUPPLIED_REPORT	N/A	N/A	N/A	N/A
244175	COMPLETED_RMSD_REPORT	0	2	auto	PASS
2454589	SCREENED_IN_NO_COMPLETE_RMSD_IN_SUPPLIED_REPORT	N/A	N/A	N/A	N/A
2502801	SCREENED_IN_NO_COMPLETE_RMSD_IN_SUPPLIED_REPORT	N/A	N/A	N/A	N/A
251255	COMPLETED_RMSD_REPORT	0	1	auto	PASS
2515638	COMPLETED_RMSD_REPORT	1	1	auto	PASS
253279	COMPLETED_RMSD_REPORT	0	1	auto	PASS
253288	COMPLETED_RMSD_REPORT	0	1	auto	PASS
253291	COMPLETED_RMSD_REPORT	0	1	auto	PASS
2535798	COMPLETED_RMSD_REPORT	0	1	auto	PASS
602743	COMPLETED_RMSD_REPORT	0	1	auto	PASS
613741	COMPLETED_RMSD_REPORT	0	1	auto	PASS
716039	COMPLETED_RMSD_REPORT	0	2	auto	PASS
780531	COMPLETED_RMSD_REPORT	0	1	auto	PASS
791724	COMPLETED_RMSD_REPORT	0	2	auto	PASS
801553	COMPLETED_RMSD_REPORT	0	1	auto	PASS
804893	COMPLETED_RMSD_REPORT	0	2	auto	PASS
804894	COMPLETED_RMSD_REPORT	0	2	auto	PASS
804896	COMPLETED_RMSD_REPORT	0	1	auto	PASS
849384	COMPLETED_RMSD_REPORT	0	1	auto	PASS
850911	COMPLETED_RMSD_REPORT	0	1	auto	PASS

Sheet1

850947	COMPLETED_RMSD_REPORT	0	1	auto	PASS
851092	COMPLETED_RMSD_REPORT	0	2	auto	PASS
876252	COMPLETED_RMSD_REPORT	0	1	auto	PASS
902738	COMPLETED_RMSD_REPORT	0	2	auto	PASS
942961	COMPLETED_RMSD_REPORT	0	1	auto	PASS
993277	COMPLETED_RMSD_REPORT	0	1	auto	PASS

Sheet1

ElectronParityCheck	FinalNumberedMOL2ChargeSum	ChargeSumMatchesStoredQ_0.01e	Warnings
PASS	0.000040	PASS	PRINCIPAL_COMPONENT_SELECTED
PASS	0.000020	PASS	PRINCIPAL_COMPONENT_SELECTED
PASS	0.000010	PASS	PRINCIPAL_COMPONENT_SELECTED
PASS	0.000000	PASS	
PASS	0.000020	PASS	
PASS	0.000000	PASS	PRINCIPAL_COMPONENT_SELECTED
PASS	0.000000	PASS	PRINCIPAL_COMPONENT_SELECTED;XTB_CHARGE_FAILED
PASS	0.000030	PASS	PRINCIPAL_COMPONENT_SELECTED
PASS	0.000010	PASS	PRINCIPAL_COMPONENT_SELECTED
PASS	0.000000	PASS	XTB_CHARGE_FAILED
N/A	N/A	N/A	No completed run archive
PASS	-2.000000	PASS	PRINCIPAL_COMPONENT_SELECTED
PASS	-0.000000	PASS	
PASS	-0.000000	PASS	
N/A	N/A	N/A	No completed run archive
WARN	0.000010	PASS	
PASS	-0.000040	PASS	
PASS	-0.000040	PASS	
PASS	0.000040	PASS	
PASS	0.000000	PASS	XTB_CHARGE_FAILED
PASS	0.000030	PASS	PRINCIPAL_COMPONENT_SELECTED

Sheet1

PASS	-0.000040	PASS	PRINCIPAL_COMPONENT_SELECTED
PASS	0.000020	PASS	PRINCIPAL_COMPONENT_SELECTED
PASS	0.000030	PASS	
PASS	0.000000	PASS	PRINCIPAL_COMPONENT_SELECTED
PASS	-0.000010	PASS	PRINCIPAL_COMPONENT_SELECTED
PASS	0.000000	PASS	XTB_CHARGE_FAILED
PASS	0.000000	PASS	PRINCIPAL_COMPONENT_SELECTED
PASS	-0.000010	PASS	PRINCIPAL_COMPONENT_SELECTED
PASS	-0.000030	PASS	
PASS	0.000010	PASS	
PASS	0.000020	PASS	
PASS	-0.000000	PASS	PRINCIPAL_COMPONENT_SELECTED
PASS	0.000000	PASS	
N/A	N/A	N/A	No completed run archive
PASS	0.000010	PASS	
PASS	3.000020	PASS	PRINCIPAL_COMPONENT_SELECTED
PASS	-0.000000	PASS	PRINCIPAL_COMPONENT_SELECTED
PASS	0.000020	PASS	PRINCIPAL_COMPONENT_SELECTED
PASS	1.000000	PASS	
PASS	-0.000010	PASS	PRINCIPAL_COMPONENT_SELECTED
PASS	0.000010	PASS	
PASS	-0.000010	PASS	PRINCIPAL_COMPONENT_SELECTED

Sheet1

PASS	-0.000000	PASS	PRINCIPAL_COMPONENT_SELECTED
PASS	0.000020	PASS	
N/A	N/A	N/A	No completed run archive
PASS	-0.000000	PASS	PRINCIPAL_COMPONENT_SELECTED
PASS	-0.000050	PASS	PRINCIPAL_COMPONENT_SELECTED
PASS	0.000030	PASS	PRINCIPAL_COMPONENT_SELECTED
PASS	-0.000020	PASS	
PASS	0.000000	PASS	
PASS	0.000060	PASS	PRINCIPAL_COMPONENT_SELECTED
PASS	-0.000020	PASS	
PASS	-0.000000	PASS	
PASS	-0.000010	PASS	
PASS	-0.000010	PASS	
PASS	0.000030	PASS	
PASS	-0.000010	PASS	
PASS	-0.000010	PASS	
PASS	0.000010	PASS	
PASS	-0.000040	PASS	PRINCIPAL_COMPONENT_SELECTED
N/A	N/A	N/A	No completed run archive
PASS	0.000010	PASS	PRINCIPAL_COMPONENT_SELECTED
N/A	N/A	N/A	No completed run archive
PASS	0.000030	PASS	PRINCIPAL_COMPONENT_SELECTED

Sheet1

N/A	N/A	N/A	No completed run archive
N/A	N/A	N/A	No completed run archive
PASS	-0.000030	PASS	PRINCIPAL_COMPONENT_SELECTED
PASS	1.000000	PASS	PRINCIPAL_COMPONENT_SELECTED
PASS	-0.000050	PASS	PRINCIPAL_COMPONENT_SELECTED
PASS	0.000000	PASS	XTB_CHARGE_FAILED
PASS	0.000000	PASS	XTB_CHARGE_FAILED
PASS	0.000000	PASS	PRINCIPAL_COMPONENT_SELECTED
PASS	-0.000010	PASS	
PASS	0.000010	PASS	
PASS	0.000000	PASS	XTB_CHARGE_FAILED
PASS	0.000010	PASS	
PASS	0.000030	PASS	PRINCIPAL_COMPONENT_SELECTED
PASS	-0.000030	PASS	
PASS	0.000000	PASS	XTB_CHARGE_FAILED
PASS	0.000000	PASS	XTB_CHARGE_FAILED
PASS	0.000000	PASS	PRINCIPAL_COMPONENT_SELECTED;XTB_CHARGE_FAILED
PASS	0.000020	PASS	
PASS	-3.000000	WARN	PRINCIPAL_COMPONENT_SELECTED;XTB_CHARGE_FAILED
PASS	0.000000	PASS	
PASS	0.000010	PASS	PRINCIPAL_COMPONENT_SELECTED
PASS	-0.000050	PASS	

Sheet1

PASS	-0.000000	PASS	PRINCIPAL_COMPONENT_SELECTED
PASS	-0.000010	PASS	
PASS	-1.000000	WARN	PRINCIPAL_COMPONENT_SELECTED;XTB_CHARGE_FAILED

Sheet1

FollowUpStateRevision	AuditScope
	Internal bookkeeping/parity audit; not an oxidation-state or ground-state determination
	Internal bookkeeping/parity audit; not an oxidation-state or ground-state determination
	Internal bookkeeping/parity audit; not an oxidation-state or ground-state determination
	Internal bookkeeping/parity audit; not an oxidation-state or ground-state determination
	Internal bookkeeping/parity audit; not an oxidation-state or ground-state determination
	Internal bookkeeping/parity audit; not an oxidation-state or ground-state determination
	Internal bookkeeping/parity audit; not an oxidation-state or ground-state determination
	Internal bookkeeping/parity audit; not an oxidation-state or ground-state determination
Follow-up used Q=0, M=1 (unchanged)	Internal bookkeeping/parity audit; not an oxidation-state or ground-state determination
QM/MD follow-up used Q=0, M=1	Internal bookkeeping/parity audit; not an oxidation-state or ground-state determination
	Q/M unavailable because no completed archive was supplied
	Internal bookkeeping/parity audit; not an oxidation-state or ground-state determination
	Internal bookkeeping/parity audit; not an oxidation-state or ground-state determination
	Internal bookkeeping/parity audit; not an oxidation-state or ground-state determination
	Q/M unavailable because no completed archive was supplied

Sheet1

	Internal bookkeeping/parity audit; not an oxidation-state or ground-state determination
	Internal bookkeeping/parity audit; not an oxidation-state or ground-state determination
	Internal bookkeeping/parity audit; not an oxidation-state or ground-state determination
	Internal bookkeeping/parity audit; not an oxidation-state or ground-state determination
	Internal bookkeeping/parity audit; not an oxidation-state or ground-state determination
	Internal bookkeeping/parity audit; not an oxidation-state or ground-state determination
	Internal bookkeeping/parity audit; not an oxidation-state or ground-state determination
	Internal bookkeeping/parity audit; not an oxidation-state or ground-state determination
	Internal bookkeeping/parity audit; not an oxidation-state or ground-state determination
	Internal bookkeeping/parity audit; not an oxidation-state or ground-state determination
	Internal bookkeeping/parity audit; not an oxidation-state or ground-state determination
	Internal bookkeeping/parity audit; not an oxidation-state or ground-state determination
	Internal bookkeeping/parity audit; not an oxidation-state or ground-state determination
	Internal bookkeeping/parity audit; not an oxidation-state or ground-state determination
	Internal bookkeeping/parity audit; not an oxidation-state or ground-state determination
	Internal bookkeeping/parity audit; not an oxidation-state or ground-state determination

Sheet1

	Internal bookkeeping/parity audit; not an oxidation-state or ground-state determination
	Internal bookkeeping/parity audit; not an oxidation-state or ground-state determination
	Internal bookkeeping/parity audit; not an oxidation-state or ground-state determination
	Q/M unavailable because no completed archive was supplied
	Internal bookkeeping/parity audit; not an oxidation-state or ground-state determination
	Internal bookkeeping/parity audit; not an oxidation-state or ground-state determination
	Internal bookkeeping/parity audit; not an oxidation-state or ground-state determination
	Internal bookkeeping/parity audit; not an oxidation-state or ground-state determination
	Internal bookkeeping/parity audit; not an oxidation-state or ground-state determination
	Internal bookkeeping/parity audit; not an oxidation-state or ground-state determination
	Internal bookkeeping/parity audit; not an oxidation-state or ground-state determination
	Internal bookkeeping/parity audit; not an oxidation-state or ground-state determination
	Internal bookkeeping/parity audit; not an oxidation-state or ground-state determination
	Internal bookkeeping/parity audit; not an oxidation-state or ground-state determination
	Q/M unavailable because no completed archive was supplied
	Internal bookkeeping/parity audit; not an oxidation-state or ground-state determination

Sheet1

	Internal bookkeeping/parity audit; not an oxidation-state or ground-state determination
	Internal bookkeeping/parity audit; not an oxidation-state or ground-state determination
	Internal bookkeeping/parity audit; not an oxidation-state or ground-state determination
	Internal bookkeeping/parity audit; not an oxidation-state or ground-state determination
	Internal bookkeeping/parity audit; not an oxidation-state or ground-state determination
	Internal bookkeeping/parity audit; not an oxidation-state or ground-state determination
	Internal bookkeeping/parity audit; not an oxidation-state or ground-state determination
	Internal bookkeeping/parity audit; not an oxidation-state or ground-state determination
	Internal bookkeeping/parity audit; not an oxidation-state or ground-state determination
	Internal bookkeeping/parity audit; not an oxidation-state or ground-state determination
	Internal bookkeeping/parity audit; not an oxidation-state or ground-state determination
	Internal bookkeeping/parity audit; not an oxidation-state or ground-state determination
	Internal bookkeeping/parity audit; not an oxidation-state or ground-state determination
	Internal bookkeeping/parity audit; not an oxidation-state or ground-state determination
	Q/M unavailable because no completed archive was supplied
	Internal bookkeeping/parity audit; not an oxidation-state or ground-state determination

Sheet1

	Q/M unavailable because no completed archive was supplied
	Internal bookkeeping/parity audit; not an oxidation-state or ground-state determination
	Q/M unavailable because no completed archive was supplied
	Q/M unavailable because no completed archive was supplied
	Internal bookkeeping/parity audit; not an oxidation-state or ground-state determination
	Internal bookkeeping/parity audit; not an oxidation-state or ground-state determination
	Internal bookkeeping/parity audit; not an oxidation-state or ground-state determination
	Internal bookkeeping/parity audit; not an oxidation-state or ground-state determination
	Internal bookkeeping/parity audit; not an oxidation-state or ground-state determination
	Internal bookkeeping/parity audit; not an oxidation-state or ground-state determination
	Internal bookkeeping/parity audit; not an oxidation-state or ground-state determination
	Internal bookkeeping/parity audit; not an oxidation-state or ground-state determination
	Internal bookkeeping/parity audit; not an oxidation-state or ground-state determination
	Internal bookkeeping/parity audit; not an oxidation-state or ground-state determination
	Internal bookkeeping/parity audit; not an oxidation-state or ground-state determination
	Internal bookkeeping/parity audit; not an oxidation-state or ground-state determination

Sheet1

	Internal bookkeeping/parity audit; not an oxidation-state or ground-state determination
	Internal bookkeeping/parity audit; not an oxidation-state or ground-state determination
	Internal bookkeeping/parity audit; not an oxidation-state or ground-state determination
	Internal bookkeeping/parity audit; not an oxidation-state or ground-state determination
	Internal bookkeeping/parity audit; not an oxidation-state or ground-state determination
	Internal bookkeeping/parity audit; not an oxidation-state or ground-state determination
	Internal bookkeeping/parity audit; not an oxidation-state or ground-state determination
	Internal bookkeeping/parity audit; not an oxidation-state or ground-state determination
QM/MD follow-up used Q=-3, M=1	Internal bookkeeping/parity audit; not an oxidation-state or ground-state determination
	Internal bookkeeping/parity audit; not an oxidation-state or ground-state determination
	Internal bookkeeping/parity audit; not an oxidation-state or ground-state determination
