# Supplementary material for: RapidMetalFF-AI: An AI-Assisted Web Server for Minute-Scale Bonded Force-Field Parameterization and Structural Validation of Metal-Containing Molecules with Validated AMBER and Converted GROMACS/CHARMM Outputs
Source: Molecules. 2026 Oct 17;31(20):3724. doi: 10.3390/molecules31203724 (PMC13707471; doi:10.3390/molecules31203724)
Supplement: Supplementary file 1 [file molecules-31-03724-s001.zip › S9_Atom_Maps/pdf_versions_of_excels/145192_historical_to_raw.pdf]

Sheet1

historical_id	nearest_raw_id	distance_A
1	2	0.0
2	29	0.0
3	30	0.0
4	31	0.0
5	32	0.0
6	33	0.0
7	34	0.0
8	35	0.0
9	36	0.0
10	37	0.0
11	38	0.0
12	39	0.0
13	40	0.0
14	41	0.0
15	42	0.0
16	43	0.0
17	44	0.0
18	45	0.0
19	46	0.0
20	47	0.0
21	48	0.0

Sheet1

22	49	0.0
23	50	0.0
24	51	0.0
25	52	0.0
26	53	0.0
27	54	0.0
28	55	0.0
29	56	0.0
30	57	0.0
31	58	0.0
32	59	0.0
33	60	0.0
34	61	0.0
35	62	0.0
36	63	0.0
37	64	0.0
38	65	0.0
39	66	0.0
40	67	0.0
41	68	0.0
42	69	0.0
