# Supplementary material for: RapidMetalFF-AI: An AI-Assisted Web Server for Minute-Scale Bonded Force-Field Parameterization and Structural Validation of Metal-Containing Molecules with Validated AMBER and Converted GROMACS/CHARMM Outputs
Source: Molecules. 2026 Oct 17;31(20):3724. doi: 10.3390/molecules31203724 (PMC13707471; doi:10.3390/molecules31203724)
Supplement: Supplementary file 1 [file molecules-31-03724-s001.zip › S9_Atom_Maps/pdf_versions_of_excels/850911_atom_mapping.pdf]

Sheet1

raw_id	name	element	residue	representative	na	nb	nc	residual_A
1	U1	U	1	1	0	0	0	0.0
2	P1	P	1	2	0	0	0	0.0
3	O1	O	1	3	0	0	0	0.0
4	O2	O	1	4	0	0	0	0.0
5	O3	O	1	5	0	0	0	0.0
6	O4	O	1	6	0	0	0	0.0
7	U1	U	1	1	-1	1	0	7.224796929169242e-05
8	U1	U	1	1	-1	0	0	0.0
9	U1	U	1	1	0	1	0	7.224796929169242e-05
10	P1A	P	1	10	0	0	0	0.0
11	O1A	O	1	11	0	0	0	0.0
12	O2A	O	1	12	0	0	0	0.0
13	O2A	O	1	12	-1	1	0	7.338823166759083e-05
14	O3	O	1	5	1	-1	0	5.3322645692248925e-05
15	O3A	O	1	15	0	0	0	0.0
16	O4	O	1	6	1	0	0	0.0
17	O4A	O	1	17	0	0	0	0.0
18	O4A	O	1	17	0	1	0	5.332264569162784e-05
19	C1	C	2	19	0	0	0	0.0
20	N1	N	2	20	0	0	0	0.0
21	H1	H	2	21	0	0	0	0.0

Sheet1

22	H2	H	2	22	0	0	0	0.0
23	H3	H	2	23	0	0	0	0.0
24	H4	H	2	24	0	0	0	0.0
25	H5	H	2	25	0	0	0	0.0
26	C1A	C	2	26	0	0	0	0.0
27	N1A	N	2	27	0	0	0	0.0
28	H1A	H	2	28	0	0	0	0.0
29	H2A	H	2	29	0	0	0	0.0
30	H3A	H	2	30	0	0	0	0.0
31	H4A	H	2	31	0	0	0	0.0
32	H5A	H	2	32	0	0	0	0.0
33	H6?	H	3	33	0	0	0	0.0
34	H7?	H	3	34	0	0	0	0.0
35	H8?	H	3	35	0	0	0	0.0
36	H9?	H	3	36	0	0	0	0.0
37	N2?	N	3	37	0	0	0	0.0

Sheet1

raw_charge
0.0
0.0
0.0
0.0
-1.0
0.0
0.0
0.0
0.0
0.0
0.0
0.0
0.0
-1.0
-1.0
0.0
0.0
0.0
0.0
1.0
0.0

Sheet1

0.0
0.0
0.0
0.0
0.0
1.0
0.0
0.0
0.0
0.0
0.0
0.0
0.0
0.0
0.0
0.0
