# Supplementary material for: RapidMetalFF-AI: An AI-Assisted Web Server for Minute-Scale Bonded Force-Field Parameterization and Structural Validation of Metal-Containing Molecules with Validated AMBER and Converted GROMACS/CHARMM Outputs
Source: Molecules. 2026 Oct 17;31(20):3724. doi: 10.3390/molecules31203724 (PMC13707471; doi:10.3390/molecules31203724)
Supplement: Supplementary file 1 [file molecules-31-03724-s001.zip › S9_Atom_Maps/pdf_versions_of_excels/850911_UP6O24_ligand_complete_NOT_QM_READY_boundary.pdf]

Sheet1

inside_new_id	inside_rep	inside_na	inside_nb	inside_nc	outside_rep	outside_na	outside_nb	outside_nc
12	4	1	-1	0	1	1	-1	0
13	4	1	0	0	1	1	0	0
14	5	0	0	0	1	-1	1	0
16	5	1	0	0	1	0	1	0
17	6	0	0	0	1	-1	0	0
18	6	1	-1	0	1	0	-1	0
23	12	-1	0	0	1	-1	0	0
24	12	-1	1	0	1	-1	1	0
26	15	0	-1	0	1	0	-1	0
28	15	1	-1	0	1	1	-1	0
30	17	0	1	0	1	0	1	0
31	17	1	0	0	1	1	0	0

Sheet1

source_bond_id	source_order	distance_A
1	1	2.2791709918301435
1	1	2.2791709918301426
12	1	2.2259217416611934
12	1	2.2259217416611934
14	1	2.2096785512829693
14	1	2.209678551282969
11	1	2.2791485493490766
11	1	2.279170991830143
13	1	2.2259138190864443
13	1	2.225913819086444
15	1	2.2096015998364953
15	1	2.2096785512829675
