# Supplementary material for: RapidMetalFF-AI: An AI-Assisted Web Server for Minute-Scale Bonded Force-Field Parameterization and Structural Validation of Metal-Containing Molecules with Validated AMBER and Converted GROMACS/CHARMM Outputs
Source: Molecules. 2026 Oct 17;31(20):3724. doi: 10.3390/molecules31203724 (PMC13707471; doi:10.3390/molecules31203724)
Supplement: Supplementary file 1 [file molecules-31-03724-s001.zip › S9_Atom_Maps/pdf_versions_of_excels/850911_UP6O24_ligand_complete_NOT_QM_READY_mapping.pdf]

Sheet1

new_id	source_raw_id	translation_representative	na	nb	nc	matching_raw_ids	raw_name	raw_type
1	1	1	0	0	0	1	U1	U
2	2	2	0	0	0	2	P1	P.3
3	2	2	1	-1	0		P1	P.3
4	2	2	1	0	0		P1	P.3
5	10	10	0	-1	0		P1A	P.3
6	10	10	0	0	0	10	P1A	P.3
7	10	10	1	-1	0		P1A	P.3
8	3	3	0	0	0	3	O1	O.co2
9	3	3	1	-1	0		O1	O.co2
10	3	3	1	0	0		O1	O.co2
11	4	4	0	0	0	4	O2	O.co2
12	4	4	1	-1	0		O2	O.co2
13	4	4	1	0	0		O2	O.co2
14	5	5	0	0	0	5	O3	O.co2
15	14	5	1	-1	0	14	O3	O.3
16	5	5	1	0	0		O3	O.co2
17	6	6	0	0	0	6	O4	O.co2
18	6	6	1	-1	0		O4	O.co2
19	16	6	1	0	0	16	O4	O.3
20	11	11	0	-1	0		O1A	O.co2
21	11	11	0	0	0	11	O1A	O.co2

Sheet1

22	11	11	1	-1	0		O1A	O.co2
23	12	12	-1	0	0		O2A	O.3
24	13	12	-1	1	0	13	O2A	O.co2
25	12	12	0	0	0	12	O2A	O.3
26	15	15	0	-1	0		O3A	O.co2
27	15	15	0	0	0	15	O3A	O.co2
28	15	15	1	-1	0		O3A	O.co2
29	17	17	0	0	0	17	O4A	O.3
30	18	17	0	1	0	18	O4A	O.co2
31	17	17	1	0	0		O4A	O.3

Sheet1

raw_charge_field	coordinate_origin
0.0	raw_exact
0.0	raw_exact
0.0	lattice_translation
0.0	lattice_translation
0.0	lattice_translation
0.0	raw_exact
0.0	lattice_translation
0.0	raw_exact
0.0	lattice_translation
0.0	lattice_translation
0.0	raw_exact
0.0	lattice_translation
0.0	lattice_translation
-1.0	raw_exact
-1.0	raw_exact
-1.0	lattice_translation
0.0	raw_exact
0.0	lattice_translation
0.0	raw_exact
0.0	lattice_translation
0.0	raw_exact

Sheet1

0.0	lattice_translation
0.0	lattice_translation
0.0	raw_exact
0.0	raw_exact
-1.0	lattice_translation
-1.0	raw_exact
-1.0	lattice_translation
0.0	raw_exact
0.0	raw_exact
0.0	lattice_translation
