# Supplementary material for: RapidMetalFF-AI: An AI-Assisted Web Server for Minute-Scale Bonded Force-Field Parameterization and Structural Validation of Metal-Containing Molecules with Validated AMBER and Converted GROMACS/CHARMM Outputs
Source: Molecules. 2026 Oct 17;31(20):3724. doi: 10.3390/molecules31203724 (PMC13707471; doi:10.3390/molecules31203724)
Supplement: Supplementary file 1 [file molecules-31-03724-s001.zip › S9_Atom_Maps/pdf_versions_of_excels/993277_atom_mapping.pdf]

Sheet1

raw_id	element	residue	representative	na	nb	nc	residual_A
1	Tb	1	1	0	0	0	0.0
2	S	1	2	0	0	0	0.0
3	N	1	3	0	0	0	0.0
4	C	1	4	0	0	0	0.0
5	C	1	5	0	0	0	0.0
6	H	1	6	0	0	0	0.0
7	C	1	7	0	0	0	0.0
8	H	1	8	0	0	0	0.0
9	C	1	9	0	0	0	0.0
10	C	1	10	0	0	0	0.0
11	C	1	11	0	0	0	0.0
12	C	1	12	0	0	0	0.0
13	H	1	13	0	0	0	0.0
14	C	1	14	0	0	0	0.0
15	H	1	15	0	0	0	0.0
16	C	1	16	0	0	0	0.0
17	O	1	17	0	0	0	0.0
18	O	1	18	0	0	0	0.0
19	S	1	19	0	0	0	0.0
20	N	1	20	0	0	0	0.0
21	C	1	21	0	0	0	0.0

Sheet1

22	C	1	22	0	0	0	0.0
23	H	1	23	0	0	0	0.0
24	C	1	24	0	0	0	0.0
25	H	1	25	0	0	0	0.0
26	C	1	26	0	0	0	0.0
27	C	1	27	0	0	0	0.0
28	C	1	28	0	0	0	0.0
29	C	1	29	0	0	0	0.0
30	H	1	30	0	0	0	0.0
31	C	1	31	0	0	0	0.0
32	H	1	32	0	0	0	0.0
33	C	1	33	0	0	0	0.0
34	O	1	34	0	0	0	0.0
35	O	1	35	0	0	0	0.0
36	S	1	36	0	0	0	0.0
37	N	1	37	0	0	0	0.0
38	C	1	38	0	0	0	0.0
39	C	1	39	0	0	0	0.0
40	H	1	40	0	0	0	0.0
41	C	1	41	0	0	0	0.0
42	H	1	42	0	0	0	0.0
43	C	1	43	0	0	0	0.0

Sheet1

44	C	1	44	0	0	0	0.0
45	C	1	45	0	0	0	0.0
46	C	1	46	0	0	0	0.0
47	H	1	47	0	0	0	0.0
48	C	1	48	0	0	0	0.0
49	H	1	49	0	0	0	0.0
50	C	1	50	0	0	0	0.0
51	O	1	51	0	0	0	0.0
52	O	1	52	0	0	0	0.0
53	C	1	53	0	0	0	0.0
54	H	1	54	0	0	0	0.0
55	C	1	55	0	0	0	0.0
56	H	1	56	0	0	0	0.0
57	O	1	57	0	0	0	0.0
58	C	1	58	0	0	0	0.0
59	H	1	59	0	0	0	0.0
60	C	1	60	0	0	0	0.0
61	H	1	61	0	0	0	0.0
62	O	1	62	0	0	0	0.0
63	C	1	63	0	0	0	0.0
64	H	1	64	0	0	0	0.0
65	C	1	65	0	0	0	0.0

Sheet1

66	H	1	66	0	0	0	0.0
67	O	1	67	0	0	0	0.0
68	S	1	68	0	0	0	0.0
69	N	1	69	0	0	0	0.0
70	C	1	70	0	0	0	0.0
71	C	1	71	0	0	0	0.0
72	H	1	72	0	0	0	0.0
73	C	1	73	0	0	0	0.0
74	H	1	74	0	0	0	0.0
75	C	1	75	0	0	0	0.0
76	C	1	76	0	0	0	0.0
77	C	1	77	0	0	0	0.0
78	C	1	78	0	0	0	0.0
79	H	1	79	0	0	0	0.0
80	C	1	80	0	0	0	0.0
81	H	1	81	0	0	0	0.0
82	C	1	82	0	0	0	0.0
83	O	1	83	0	0	0	0.0
84	O	1	84	0	0	0	0.0
85	C	1	85	0	0	0	0.0
86	H	1	86	0	0	0	0.0
87	C	1	87	0	0	0	0.0

Sheet1

88	H	1	88	0	0	0	0.0
89	O	1	89	0	0	0	0.0
90	Tb	1	1	-1	-1	0	0.0001414213562369799
91	Tb	1	1	-1	0	0	0.0
92	Tb	1	1	0	-1	0	0.0001414213562369799
93	Tb	1	93	0	0	0	0.0
94	Tb	1	93	0	1	0	0.0001414213562369799
95	Tb	1	93	1	0	0	0.0
96	Tb	1	93	1	1	0	0.0001414213562369799
97	O	1	83	1	1	0	0.0
98	O	1	34	1	0	0	0.0
99	O	1	51	0	1	0	0.0
100	O	1	100	0	0	0	0.0
101	O	1	101	0	0	0	0.0
102	O	1	102	0	0	0	0.0
103	O	1	103	0	0	0	0.0
104	O	2	104	0	0	0	0.0
105	O	3	105	0	0	0	0.0

Sheet1

raw_charge
0.0
0.0
0.0
0.0
0.0
0.0
0.0
0.0
0.0
0.0
0.0
0.0
0.0
0.0
0.0
0.0
0.0
0.0
0.0
0.0
0.0

Sheet1

0.0
0.0
0.0
0.0
0.0
0.0
0.0
0.0
0.0
0.0
0.0
0.0
0.0
0.0
0.0
0.0
0.0
0.0
0.0
0.0
0.0
0.0

Sheet1

0.0
0.0
0.0
0.0
0.0
0.0
0.0
0.0
0.0
0.0
0.0
0.0
0.0
0.0
0.0
0.0
0.0
0.0
-1.0
0.0
0.0
0.0

Sheet1

0.0
0.0
0.0
0.0
0.0
0.0
0.0
0.0
0.0
0.0
0.0
0.0
0.0
0.0
0.0
0.0
0.0
0.0
0.0
0.0
0.0
0.0

Sheet1

0.0
0.0
0.0
0.0
0.0
0.0
0.0
0.0
0.0
0.0
0.0
0.0
0.0
0.0
0.0
0.0
0.0
1.0
