# Supplementary material for: RapidMetalFF-AI: An AI-Assisted Web Server for Minute-Scale Bonded Force-Field Parameterization and Structural Validation of Metal-Containing Molecules with Validated AMBER and Converted GROMACS/CHARMM Outputs
Source: Molecules. 2026 Oct 17;31(20):3724. doi: 10.3390/molecules31203724 (PMC13707471; doi:10.3390/molecules31203724)
Supplement: Supplementary file 1 [file molecules-31-03724-s001.zip › Supplementary_Table_S6.pdf]

1. Top-level input, preprocessing, metadata, and identity files

The top level of the result directory preserves both the molecular structure entering the scientific workflow and the records describing preprocessing and calculation status.

2087213_input.mol2 contains the principal molecular component selected for parameterization. When the submitted MOL2 contains multiple molecular records, disconnected components, solvent molecules, counterions, or other auxiliary species, the structure retained for calculation is written to this file.

When applicable, 2087213_input_original.mol2 preserves the original uploaded structure before principal-component selection, while 2087213_input_before_principal_filter.mol2 records the normalized MOL2 immediately before principal-component filtering.

The corresponding preprocessing operations are documented in machine-readable form through:

- mol2_multirecord_normalization.json
- mol2_preflight.json
- mol2_principal_component_filter.json

These records allow the submitted and retained molecular entities to be compared retrospectively and preserve the structural admission-control decisions made before parameterization.

General calculation information is stored in rapidmetalff_metadata.json, including the charge and spin state used by the workflow, calculation status, stage information, runtime data, and method/version information. orb_stage_metadata.json contains metadata specifically associated with the OrbMol-v2 stage.

Molecular-identity information is supplied in both machine-readable and human-readable formats through:

- molecule_identity.json
- molecule_identity.txt

These files contain the molecular identifiers generated by the RapidMetalFF-AI identity layer and support subsequent library searching and verified result reuse.

haptic_detection.json records whether a haptic metal-ligand coordination pattern was detected. The root-level structure_integrity_post_orbmol.json records the structural-integrity assessment carried out after OrbMol-v2 geometry optimization and before subsequent parameterization stages.

2. orb_output/: OrbMol-v2 geometry and Hessian data

The orb_output/ directory contains the primary scientific information generated during the machine-learning stage.

2087213_optimized.xyz contains the OrbMol-v2 optimized molecular geometry. This structure serves as the reference geometry for Hessian generation, bonded-parameter derivation, and optimized-reference structural-retention analysis.

2087213_hessian.npy contains the Cartesian finite-difference Hessian calculated from OrbMol-v2 force predictions. This Hessian provides the curvature information used by the Seminario projection to derive bond and angle force constants.

Retention of both files allows the geometry and curvature data underlying the RapidMetalFF bonded parameters to be inspected independently of the final force-field files.

3. orb_metal_ff_output/: Native RapidMetalFF parameterization

The orb_metal_ff_output/ directory contains the principal outputs of native RapidMetalFF force-field generation.

2087213.frcmod contains the generated RapidMetalFF parameters, including the bonded terms constructed for the processed molecular system.

2087213_numbered.mol2 contains the corresponding molecular structure with the RapidMetalFF/ANS atom types and position-specific coordination-site numbering used during parameterization.

2087213_atom_mapping.json preserves atom correspondence across the original, optimized, numbered, and subsequently minimized structures. This mapping allows atom identities to be followed across the complete workflow and supports molecule-resolved structural comparison.

For systems in which haptic coordination is detected, additional records such as haptic_detection.json and haptic_parameter_symmetry.json describe the detected coordination pattern and the corresponding symmetry treatment applied during parameter generation.

4. openmm/: Native force-field assembly, minimization, and structural validation

The openmm/ directory is particularly important because the generated RapidMetalFF FRCMOD file is not delivered without further testing. The generated parameters are first assembled into an AMBER-compatible molecular system and are then applied to the molecule in an OpenMM energy minimization.

The simulation-ready AMBER files are:

- 2087213.prmtop
- 2087213.inpcrd

These contain the topology, parameter assignments, and starting coordinates used for native RapidMetalFF minimization.

The final minimized coordinates are retained in several representations:

- 2087213_min.rst7
- 2087213_min.pdb
- 2087213_numbered_min.mol2
- 2087213_min_for_view_no_numbering.mol2

2087213_numbered_min.mol2 preserves the RapidMetalFF/ANS atom numbering in the final minimized geometry, making it possible to relate the structural result directly to the atom types and bonded parameters used during force-field generation.

Because MOL2 structures containing the internal RapidMetalFF numbering and atom types are not interpreted correctly by all molecular-visualization programs, 2087213_min_for_view_no_numbering.mol2 provides an additional Sybyl-compatible visualization copy without the internal ANS numbering. The corresponding conventional PDB representation is provided as 2087213_min.pdb.

Thus, the result archive supplies both the internally numbered structure required for parameter tracing and conventional visualization-oriented structures that can be opened directly in widely used molecular-viewing software.

2087213_min.out retains the OpenMM minimization output record.

Structural validation is performed on the minimized structure. 2087213_rmsd.dat contains the automatically calculated structural deviation between the OrbMol-v2 optimized reference and the native RapidMetalFF-minimized geometry.

structure_integrity_post_openmm.json records the post-minimization structural-integrity assessment and allows possible connectivity disruption, atom displacement, clashes, or other major structural abnormalities to be identified independently of the RMSD value.

Supporting records retained in this directory include:

- cpptraj.log
- leap.log
- min.in

These document the structural-analysis operation, AMBER system assembly, and minimization configuration, respectively.

5. hybrid/: Hybrid RapidMetalFF/GAFF2 construction and validation

The hybrid/ directory contains the complete alternative Hybrid RapidMetalFF/GAFF2 realization.

In this branch, the RapidMetalFF description is retained for the protected metal-coordination environment, whereas suitable ligand-region atom types and bonded terms outside the protected region are supplied by GAFF2.

The principal Hybrid parameter files are:

- 2087213_hybrid.frcmod
- 2087213_hybrid.mol2

The corresponding simulation-ready AMBER files are:

- 2087213_hybrid.prmtop
- 2087213_hybrid.inpcrd

As in the native branch, the Hybrid parameterization is actually applied to the molecular system and subjected to an independent OpenMM minimization rather than being delivered as an untested FRCMOD file.

The resulting minimized Hybrid structures are provided as:

- 2087213_hybrid_min.rst7
- 2087213_hybrid_min.pdb
- 2087213_hybrid_numbered_min.mol2
- 2087213_hybrid_min_for_view_no_numbering.mol2

The numbered MOL2 retains the internal atom-type identities used by the Hybrid force field, whereas the non-numbered MOL2 provides a Sybyl-compatible visualization representation for programs that cannot correctly display the internally numbered atom types. The PDB representation provides an additional broadly compatible visualization and structural-analysis format.

2087213_hybrid_rmsd.dat contains the automatically calculated RMSD between the OrbMol-v2 optimized reference structure and the final Hybrid minimized geometry.

2087213_hybrid_vs_rapidmetalff_rmsd.txt provides an additional direct structural comparison between the minimized native RapidMetalFF and Hybrid RapidMetalFF/GAFF2 realizations.

The Hybrid directory also retains detailed records describing how the combined force field was constructed:

- 2087213_hybrid_typing.json
- 2087213_hybrid_replacement_report.txt
- 2087213_hybrid_parmed_status.txt

2087213_hybrid_typing.json provides a machine-readable description of atom typing and parameter assignment during Hybrid construction.

2087213_hybrid_replacement_report.txt provides a human-readable record of the RapidMetalFF/GAFF2 parameter or atom-type replacements used to construct the Hybrid representation.

2087213_hybrid_parmed_status.txt records the status of subsequent ParmEd-based topology conversion.

Branch-specific processing logs are retained directly within hybrid/, including:

- tleap.log
- openmm.log
- parmed.log
- mol2_view_copy.log
- cpptraj.log
- leap.log

These files allow AMBER assembly, OpenMM minimization, ParmEd conversion, visualization-MOL2 generation, and Hybrid structural-analysis operations to be inspected separately from the native branch.

6. hybrid/hybrid_parmed/: Converted Hybrid simulation formats

The hybrid_parmed/ subdirectory contains additional force-field and coordinate representations generated from the Hybrid AMBER system using ParmEd.

Representative outputs include:

- .pdb
- .gro
- .top
- .psf
- .str
- .cif
- .crd
- .prmtop
- .rst7

These conversions allow the same Hybrid parameterization to be transferred to AMBER-, GROMACS-, CHARMM-, and other compatible molecular-simulation or visualization environments without reconstructing the force field from the original FRCMOD file.

7. exports/: Native RapidMetalFF multi-format export

The top-level exports/ directory provides corresponding multi-format conversions for the native RapidMetalFF branch.

Supported exported representations include:

- .cif
- .crd
- .gro
- .mol2
- .pdb
- .prmtop
- .psf
- .rst7
- .str
- .top

Together with the primary native AMBER files, these outputs provide direct access to commonly used AMBER-, GROMACS-, and CHARMM-compatible molecular representations and broadly supported structural formats.

8. logs/: Stage-specific calculation audit trail

The logs/ directory collects diagnostic information from the complete automated workflow.

The principal log files include:

- preflight.log - structural admission-control screening
- mol2_multirecord_normalization.log - processing of multirecord MOL2 input
- mol2_principal_component_filter.log - deterministic principal-component selection
- orb.log - OrbMol-v2 optimization and Hessian generation
- metal_ff.log - native RapidMetalFF parameter generation
- xtb_charge.log - GFN1-xTB/CM5 charge assignment
- pipeline_ff.log - AMBER/OpenMM force-field assembly and validation
- hybrid_gaff2.log - Hybrid RapidMetalFF/GAFF2 construction
- identity.log - molecular-identity generation
- export_ff.log - final multi-format export

These logs provide a stage-resolved audit trail and allow an unsuccessful or unexpected result to be localized to a specific part of the workflow.

9. Functional interpretation of the result package

The RapidMetalFF-AI result package is therefore designed to contain substantially more than the generated parameter file alone.

For both the native and Hybrid branches, the complete sequence retained in the archive is:

generated FRCMOD -> AMBER system assembly -> OpenMM minimization -> final minimized structure -> RMSD and structural-integrity assessment -> simulation/visualization export

This organization allows users to determine not only which parameters were generated but also the structural result obtained when those parameters were actually applied to the submitted molecular system.

The simultaneous availability of internally numbered MOL2 files and conventional Sybyl MOL2/PDB representations serves two complementary purposes. The numbered files preserve the precise RapidMetalFF/ANS atom identities required for parameter tracing and reproducibility, whereas the non-numbered Sybyl MOL2 and PDB files provide immediately viewable representations for molecular-visualization software that does not support the internal RapidMetalFF atom-type notation.

The ZIP archive consequently serves both as a simulation-ready force-field deliverable and as an inspectable record of parameter generation and structural validation.

Supplementary Table S6. RapidMetalFF-AI result-package file inventory

Location	Representative file(s)	Function
Root	2087213_input.mol2	Principal molecular component entering parameterization
Root	2087213_input_original.mol2	Original uploaded MOL2 where preservation is required
Root	2087213_input_before_principal_filter.mol2	Normalized input immediately before principal-component filtering
Root	mol2_multirecord_normalization.json	Machine-readable record of MOL2 multirecord normalization
Root	mol2_preflight.json	Preflight screening result and structural warnings/failures
Root	mol2_principal_component_filter.json	Principal-component selection record
Root	rapidmetalff_metadata.json	Overall calculation status, Q/M, versions, stages, and runtime metadata
Root	orb_stage_metadata.json	OrbMol-v2-specific metadata
Root	molecule_identity.json; molecule_identity.txt	Machine- and human-readable molecular-identity records
Root	haptic_detection.json	Haptic coordination detection record
Root	structure_integrity_post_orbmol.json	Post-OrbMol-v2 structural-integrity assessment
orb_output/	2087213_optimized.xyz	OrbMol-v2 optimized reference geometry
orb_output/	2087213_hessian.npy	Cartesian Hessian used for bond/angle parameter derivation
orb_metal_ff_output/	2087213.frcmod	Native RapidMetalFF force-field parameters
orb_metal_ff_output/	2087213_numbered.mol2	ANS-numbered/typed structure used by native RapidMetalFF
orb_metal_ff_output/	2087213_atom_mapping.json	Atom correspondence across workflow stages
orb_metal_ff_output/	haptic_parameter_symmetry.json	Haptic parameter-symmetry treatment when applicable
openmm/	2087213.prmtop; 2087213.inpcrd	Simulation-ready native AMBER topology and coordinates
openmm/	2087213_min.rst7	Native minimized AMBER restart coordinates
openmm/	2087213_min.pdb	Conventional PDB representation of native minimized geometry
openmm/	2087213_numbered_min.mol2	Native minimized geometry retaining internal ANS/RapidMetalFF numbering
openmm/	2087213_min_for_view_no_numbering.mol2	Sybyl-compatible visualization MOL2 without internal numbering
openmm/	2087213_min.out	Native OpenMM minimization output
openmm/	2087213_rmsd.dat	Native optimized-reference RMSD validation result
openmm/	structure_integrity_post_openmm.json	Post-minimization structural-integrity assessment
openmm/	cpptraj.log; leap.log; min.in	Structural analysis, AMBER assembly, and minimization records
hybrid/	2087213_hybrid.frcmod	Hybrid RapidMetalFF/GAFF2 parameter set
hybrid/	2087213_hybrid.mol2	Hybrid atom-typed molecular structure
hybrid/	2087213_hybrid.prmtop; 2087213_hybrid.inpcrd	Simulation-ready Hybrid AMBER topology and coordinates
hybrid/	2087213_hybrid_min.rst7	Hybrid minimized AMBER restart coordinates
hybrid/	2087213_hybrid_min.pdb	Conventional PDB representation of Hybrid minimized geometry
hybrid/	2087213_hybrid_numbered_min.mol2	Hybrid minimized structure retaining internal atom types
hybrid/	2087213_hybrid_min_for_view_no_numbering.mol2	Sybyl-compatible visualization copy of Hybrid minimized structure
hybrid/	2087213_hybrid_rmsd.dat	Hybrid optimized-reference RMSD validation result
hybrid/	2087213_hybrid_vs_rapidmetalff_rmsd.txt	Direct native-versus-Hybrid minimized-geometry comparison
hybrid/	2087213_hybrid_typing.json	Machine-readable Hybrid typing and parameter-assignment record
hybrid/	2087213_hybrid_replacement_report.txt	Human-readable Hybrid replacement report
hybrid/	2087213_hybrid_parmed_status.txt	ParmEd conversion-stage status
hybrid/	tleap.log; openmm.log; parmed.log	Hybrid AMBER assembly, minimization, and conversion logs
hybrid/	mol2_view_copy.log	Hybrid visualization-MOL2 generation record
hybrid/	cpptraj.log; leap.log	Hybrid-specific structural-analysis and assembly records
hybrid/hybrid_parmed/	.pdb; .gro; .top; .psf; .str; .cif; .crd; .prmtop; .rst7	Converted Hybrid simulation and structural formats
exports/	.cif; .crd; .gro; .mol2; .pdb; .prmtop; .psf; .rst7; .str; .top	Multi-format native RapidMetalFF exports
logs/	preflight.log	Preflight screening log
logs/	mol2_multirecord_normalization.log	MOL2 normalization log
logs/	mol2_principal_component_filter.log	Principal-component filtering log
logs/	orb.log	OrbMol-v2 optimization/Hessian log
logs/	metal_ff.log	Native force-field generation log
logs/	xtb_charge.log	GFN1-xTB/CM5 charge-assignment log
logs/	pipeline_ff.log	Force-field assembly/OpenMM validation log
logs/	hybrid_gaff2.log	Hybrid construction log
logs/	identity.log	Molecular-identity generation log
logs/	export_ff.log	Multi-format export log

Note: File availability may depend on the molecular topology and workflow path. For example, haptic-specific records are generated only when the corresponding coordination pattern is detected, and preservation of original/pre-filter structures is relevant primarily to multirecord or multicomponent inputs.
